# Do patients with different craniofacial patterns have differences in upper airway volume? A systematic review with network meta-analysis

**DOI:** 10.1093/ejo/cjae010

**Published:** 2024-03-25

**Authors:** Charlotte Altheer, Spyridon N Papageorgiou, Gregory S Antonarakis, Alexandra K Papadopoulou

**Affiliations:** Division of Orthodontics, University Clinics of Dental Medicine, Faculty of Medicine, University of Geneva, Geneva, Switzerland; Clinic of Orthodontics and Pediatric Dentistry, Center for Dental Medicine, University Zurich, Zurich, Switzerland; Division of Orthodontics, University Clinics of Dental Medicine, Faculty of Medicine, University of Geneva, Geneva, Switzerland; Division of Orthodontics, University Clinics of Dental Medicine, Faculty of Medicine, University of Geneva, Geneva, Switzerland; Discipline of Orthodontics, Sydney Dental School, Faculty of Medicine and Health, The University of Sydney, Sydney, Australia

## Abstract

**Background:**

Craniofacial skeletal discrepancies have been associated with upper airway dimensions.

**Objective:**

To identify differences in upper airway volume across different sagittal and vertical skeletal patterns.

**Search methods:**

Unrestricted literature searches in eight databases/registers for human studies until May 2023.

**Selection criteria:**

Cross-sectional studies measuring upper airway volumes using three-dimensional imaging in healthy patients of different sagittal (Class I, Class II, and Class III) or vertical (normodivergent, hypodivergent, and hyperdivergent) craniofacial morphology.

**Data collection and analysis:**

Duplicate independent study selection, data extraction, and risk of bias assessment. Random-effects frequentist network meta-analysis was performed followed by subgroup-analyses and assessment of the quality of clinical recommendations (confidence in effect estimates) with the CINeMA (Confidence in Network Meta-Analysis) approach.

**Results:**

Seventy publications pertaining to 66 unique studies were included with 56 studies (5734 patients) contributing to meta-analyses. Statistically significant differences were found for total  pharyngeal airway volume, with Class II having decreased airway volume (−2256.06 mm^3^; 95% Confidence Interval [CI] −3201.61 to −1310.51 mm^3^) and Class III increased airway volume (1098.93 mm^3^; 95% CI 25.41 to 2172.45 mm^3^) compared to Class I. Significant airway volume reductions for Class II were localized mostly at the oropharynx, followed by the palatopharynx, and the glossopharynx. Significant airway volume increases for Class III were localized mostly at the oropharynx, followed by the intraoral cavity, and hypopharynx. Statistically significant differences according to vertical skeletal configuration were seen only for the oropharynx, where hyperdivergent patients had reduced volumes compared to normodivergent patients (−1716.77 mm^3^; 95% CI −3296.42 to −137.12 mm^3^). Airway differences for Class II and Class III configurations (compared to Class I) were more pronounced in adults than in children and the confidence for all estimates was very low according to CINeMA.

**Conclusions:**

Considerable differences in upper airway volume were found between sagittal and vertical skeletal configurations. However, results should be interpreted with caution due to the high risk of bias, owing to the retrospective study design, inconsistencies in anatomic compartment boundaries used, samples of mixed children–adult patients, and incomplete reporting.

**Clinical Trial Registration:**

PROSPERO (CRD42022366928).

## Introduction

### Rationale

The use of three-dimensional (3D) imaging, in terms of Computerized Tomography (CT) or Cone Beam Computerized Tomography (CBCT), has enabled the accurate estimation of the volume of craniofacial bones and airways [1, 2] and the assessment of the effect of various orthodontic or orthopedic treatments on airway volumes, including mandibular advancement [3], and maxillary expansion [4–6].

The use of 3D imaging can also in some instances aid in the diagnosis and treatment of breathing disorders [7–9]. Formally, the definitive diagnosis of sleep-related breathing disorders necessitates dedicated diagnostics in a laboratory-based setting such as the use of polysomnography for the diagnosis of obstructive sleep apnea. However, craniofacial morphology tends to differ between disease-free and apneic patients, with the latter having significantly reduced pharyngeal airway space, inferiorly placed hyoid bone, and increased anterior facial height [10]. In such cases, identification of such risk factors through 3D evaluation of craniofacial morphology along with patient-reported symptoms can lead to an early referral to the appropriate specialist (e.g. otorhinolaryngologist), and the orthodontist might in some instances be the first healthcare professional to identify these problems.

CBCTs and CTs might allow us to identify a potential association between sagittal/ vertical craniofacial morphology and upper airway anatomic measurements. Several studies have investigated this [11–14]—albeit with contradictory results. More specifically, a tendency for an inverse relationship between lower pharynx (velopharynx and oropharynx) volume and ANB angle was reported [11], with the oropharyngeal volume being reduced in those with mandibular retrusion and increased in those with mandibular protrusion [12]. Similarly, smaller SNB values were associated with smaller nasopharyngeal and oropharyngeal volumes, with differences between male and female patients becoming more evident at the completion of growth, while the vertical skeletal configuration did not seem to influence the upper airway volumes [13]. Contrary to this, another study found that facial divergence was associated with differences mainly in the oropharyngeal volumes, with hypodivergent patients having greater volumes than hyperdivergent ones [14].

### Objectives

The aim of the present study was therefore to appraise in a systematic manner evidence from studies using 3D imaging modalities on the association between skeletal discrepancies in the sagittal or vertical dimension and the upper airway volume of healthy human patients. The objectives were to identify differences, if these exist, in the upper airway volume among either (i) patients with skeletal Class I, II, and III sagittal patterns; or (ii) patients with normodivergent, hyperdivergent, and hypodivergent skeletal vertical patterns.

## Methods

### Protocol, registration, and eligibility criteria

The present systematic review, conducted according to the Cochrane Handbook [15], is reported using the Preferred Reporting Items for Systematic Reviews and Meta-Analyses (PRISMA) guidelines for network meta-analyses [16]. Its protocol was pre-registered (CRD42022366928), and its dataset is openly provided [17].

The eligibility criteria and the Participants-Exposure-Comparison-Outcome-Setting (PECOS) characteristics of the included studies are outlined in Supplementary Table S1. To compare the upper airway volumes, the sagittal skeletal craniofacial morphology was classified based on the ANB angle, which measures the anteroposterior maxilla-to-mandible relationship and classifies the patients into Class I, II, and III. The vertical skeletal morphology was assessed with the relative position of horizontal reference planes such as the anterior cranial base (SN), the Frankfort plane (FH), or the maxillary plane (ANS-PNS) to the mandibular plane (GoMe). Measurements used included SN-GoMe, FH-GoMe, ANS-PNS-GoMe, or any other cephalometric measurements the authors used to classify the patients into normo-, hypo-, and hyper-divergent.

### Information sources and search

The databases, registers, websites, organizations, and other sources searched to identify potentially eligible studies as well as all search strategies are described in Supplementary Table S2. The last electronic literature search was performed on May 2023 and no limitations regarding publication year, language, status, or type were imposed (apart from filters for studies on humans, where they existed). Additionally, the reference lists of all included studies and all relevant systematic reviews were checked for additional studies.

### Study selection

Two authors (C.A. and A.K.P.) independently performed the database search, de-duplication, and selection of studies according to title, abstract, and full text. Titles and/or abstracts of studies retrieved from the searches and those from additional sources (hand searching or reference/citation lists) were screened to identify articles that potentially met the inclusion criteria. The full text of these potentially eligible studies, as well as of those abstracts which did not provide sufficient information to allow decision-making, were retrieved and assessed by the same review authors (C.A. and A.K.P.), while a third author (G.S.A.) confirmed the decisions and was consulted for consensus in case of discrepancies.

### Data items and collection process

The data collected from each included study are given in Supplementary Table S3. Two authors (C.A. and A.K.P.), independently and in duplicate, performed data extraction using pre-designed and -piloted forms and imported data in digital spreadsheets. Discrepancies were resolved in the same way as above by consulting another author (S.N.P.). All relevant citations were imported to a reference management software (EndNote® 20, Thomson Reuters, Philadelphia, PA) for de-duplication. Researchers were not blinded to the authors of the included studies.

### Geometry of the network

The geometry of the network was visualized with network plots according to whether studies compared different skeletal sagittal categories (Class I, Class II, or Class III) or different skeletal vertical categories (normodivergent, hypodivergent, or hyperdivergent).

### Internal validity/risk of bias within individual studies

A customized risk of bias tool was used independently by two investigators (C.A. and A.K.P.) to assess the internal validity/risk of bias/reporting quality within each included study. This tool was tailored to the scope of this review’s eligible studies and was based on items from The Joanna Briggs Institute’s critical appraisal checklist for cross-sectional studies [18] and the appraisal tool for cross-sectional studies [19]. The specific domains/questions of the customized tool can be seen in Supplementary Table S4.

### Summary measures and planned method of analysis

Studies were considered eligible for pooling if patients fulfilled the same eligibility criteria and the same anatomic boundaries (irrespective of how the authors termed them) were used (if clarification was possible from the description provided). The methods section of all included studies was checked for the boundaries used and irrespective of how the authors named the compartments, data were extracted based on the boundaries and not the naming the authors gave of the compartment. In studies that used landmarks outside of the upper airways such as the borders of the cervical vertebra, these boundaries were transferred to the nearest upper airway landmark for grouping the relevant compartment accordingly.

**Figure 1.**
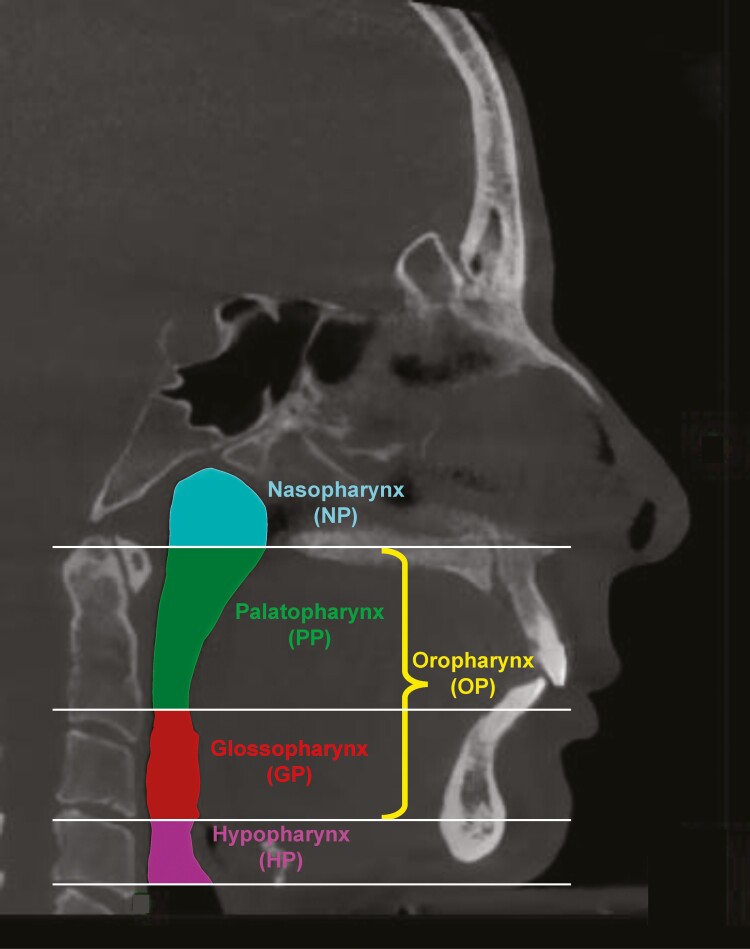
Anatomic boundaries of the upper airway compartments. All the horizontal reference planes are parallel to the Frankfurt horizontal plane. Nasopharynx (NP) is defined superiorly by the top of the pharynx and inferiorly by the plane passing from the posterior nasal spine (PNS). Palatopharynx (PP) is defined superiorly by the plane passing from the PNS and inferiorly by the plane passing from the uvula tip. Glossopharynx (GP) is defined superiorly by the plane passing from the uvula tip and inferiorly by the plane passing from the epiglottis tip. PP and GP together comprise the oropharynx (OP). Hypopharynx (HP) is defined superiorly by the plane passing from the epiglottis tip and inferiorly by the plane passing from the epiglottis base.

Boundary definitions in the present review were adopted by widely accepted definitions used in the medical literature. The compartments of the pharynx were designated by horizontal reference planes from certain anatomic landmarks within the pharynx and parallel to the Frankfort horizontal plane (FH). More specifically, the nasopharynx was the compartment defined superiorly by the top of the pharynx, inferiorly by the plane that passes from the posterior nasal spine (PNS) parallel to FH, anteriorly by the plane that passes from PNS and parallel to the coronal plane. The palatopharynx was the compartment between the plane that passes from the PNS parallel to FH and the plane that passes from the uvula tip parallel to FH. The glossopharynx was the compartment between the previously defined plane and a plane passing from the epiglottis tip parallel to FH. The palatopharynx and the glossopharynx together comprised the oropharynx. The hypopharynx was the compartment defined by the previously described plane and a plane that passes from the epiglottis base and parallel to FH (Fig. 1). Finally, the total airway volume was defined as the sum of the nasal cavity, nasopharynx, palatopharynx, glossopharynx, and the hypopharynx.

To optimize the data yielded from included studies, attempts were made to include data from all available studies independent of their reporting completeness, and data calculations were made by us, where necessary (Supplementary 1). The mean difference (MD) with its 95% confidence interval (CI) was used to estimate airway volume differences among Class I, II, and III or among normodivergent, hypodivergent, and hyperdivergent patient groups. As airway volumes were expected to vary according to patient-related (chronological age, developmental growth stage, sex) or measurement-related factors (radiographical technical characteristics, method error, cut-off values used for categorization), a random-effects model was a priori deemed (using clinical/methodological justification [20]) most appropriate to incorporate this variability and estimate the average distribution of effects across studies.

As far as identifiability is concerned, heterogeneity (tau^2^) was assumed to be the same across all comparisons of each network [15] that was calculated with a REstricted Maximum Likelihood (REML) estimator and its 95% CI using the Q-profile approach, based on empirical evidence [21]. Summary effect sizes and their 95% CIs were calculated using the Hartung–Knapp modification [22] to handle meta-analyses with a small number of studies. Relative between-study heterogeneity (inconsistency) was assessed using the *I*² statistic with its 95% uncertainty interval [23]. Heterogeneity between studies was assessed by comparing 95% prediction intervals to 95% CIs, while inconsistency (discrepancies between direct/indirect evidence) was assessed with both local tests [24] and a global design-by-treatment interaction [25]. When substantial inconsistency was found, only conventional (pairwise) meta-analyses of direct evidence were presented instead of network meta-analyses. To examine the transitivity assumption of network meta-analysis, the distribution of expected effect modifiers (patient age and sex) was examined across studies grouped by comparison.

All given *P*-values are two-sided with a significance level set at 5% (except for tests of between-study or between-subsets heterogeneity where it was 10%) and all statistical analyses were performed by one author (SNP) in R (version 4.0.4), with an openly provided dataset [17].

### Assessment of inconsistency, risk of bias, and additional analyses

Possible sources of heterogeneity in network meta-analyses were identified by pre-specified mixed-effects subset analyses or random-effects network meta-regression and we arbitrarily set the cut-off to networks with at least 10 studies for a specific outcome. Pre-defined network subgroup/meta-regression analyses included subsets according to patient age-group (children, adults, mixed), mean age, and percentage of male patients in the study sample.

The credibility of the evidence from network meta-analyses was rated using the CINeMA (Confidence in Network Meta-Analysis) approach [26]. For comparisons with at least 10 studies, contour-enhanced funnel plots for asymmetry were inspected and the Egger’s test was applied [27].

## Results

### Study selection

**Figure 2.**
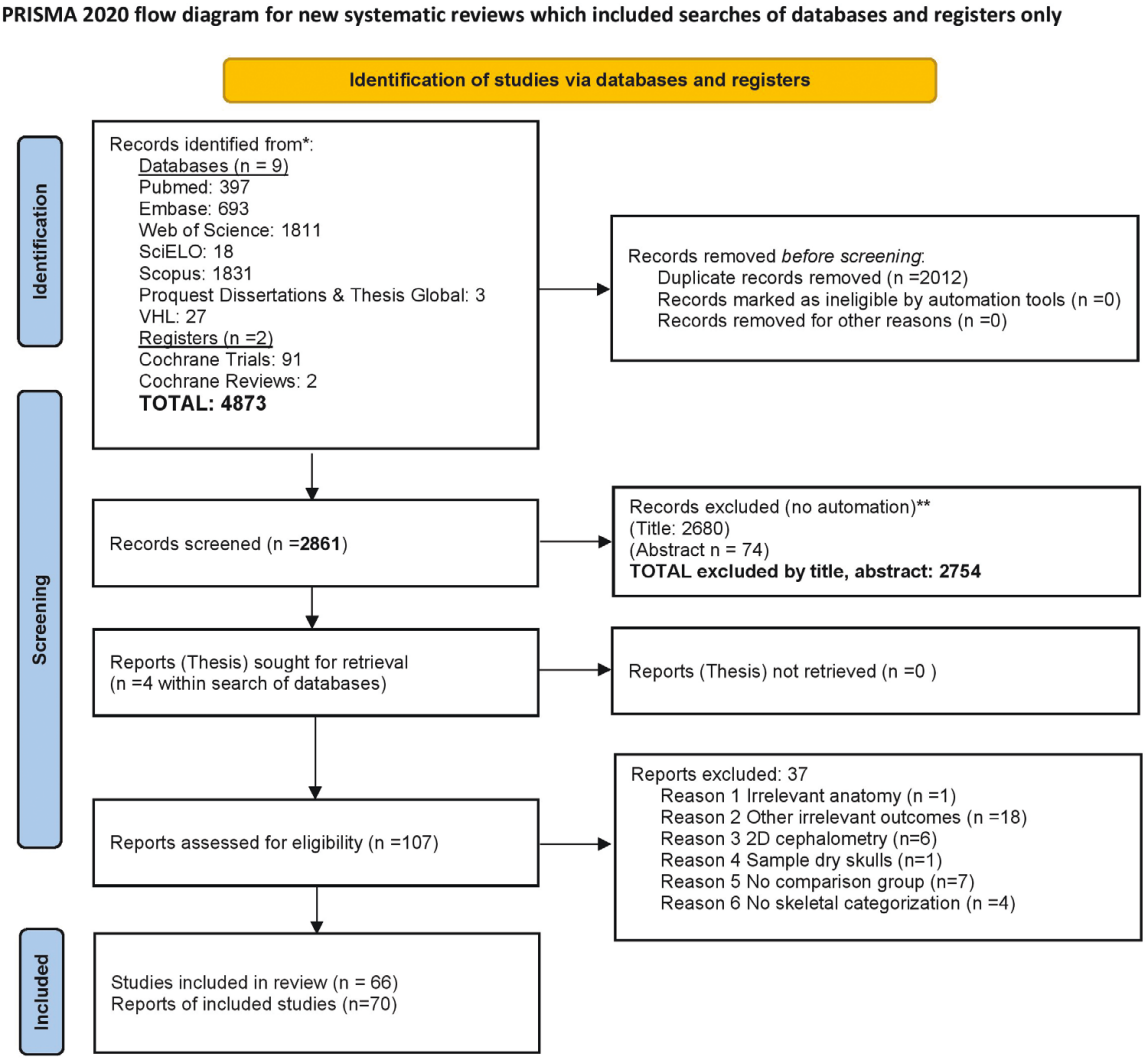
PRISMA flowchart diagram.

A total of 4873 studies were retrieved from the electronic search, which after de-duplication and selection by title, abstract, or full text resulted in 66 studies (70 reports) finally being included in the present review (Supplementary Table S5, Fig. 2). From these, 57 unique studies provided usable data and categorizations according to different sagittal or vertical skeletal discrepancies and were included in network meta-analyses.

### Presentation of network structure and geometry

The network structure for all outcomes assessed by studies allowing network meta-analysis can be seen in Supplementary Figs. S1–S10. All comparisons were found to be reasonably transitive for the most important effect modifiers (age and sex), as can be seen in Supplementary Figs. S11–S14.

### Study characteristics

Study characteristics of all 66 studies (70 reports) included in the descriptive analysis (systematic review) are described in detail in Supplementary Tables S6 and S7. Overall, 10 studies were of prospective design and the remaining 56 were of retrospective design. Three studies were conducted in Europe, 23 studies in the Americas, 39 in Asia, and one study in Oceania. Image aquisition was accomplished using CBCT in most of the studies (61 studies) while conventional medical CTs were used in four studies and MRI was used in only one study. Additionally, nine studies reported only correlations between upper airway volumes with sagittal (SNA, SNB, ANB) and/or vertical (SN-MP, FH-MP, face height) cephalometric measurements without skeletal categorization of their sample. Among the 56 studies included in the network meta-analyses, the median sample size was 74 patients per study (range 25–298 patients per study) to a total of 5734 patients. As far as age groups are concerned, 25% of studies (*n* = 14) included children, 45% (*n* = 25) included adults, and 30% (*n* = 17) included both children and adults. Among the 40 studies reporting on the age of study participants, the median age was 21.7 years, while among the 47 studies reporting on sex, the median percentage of male patients in the sample was 48.3%.

### Internal validity/risk of bias/reporting quality of included studies

The evaluation of the included studies is presented in detail and as a summary in Supplementary Tables S8–S10. All studies were deemed to present a high risk of bias due to several potential threats to their internal validity. The most problematic domains pertained to lack of identifying and dealing with confounders (91% of studies), lacking justification for the study’s sample size (79% of studies), questionable validity of the outcome measurement method (53% of studies), and lack of assessment for the measurement method error (29% of studies). Issues with the reporting quality were often identified for the included studies, as unclarities were seen pertaining to blinding of the outcome assessor (98% of studies), experience/calibration of the outcome assessor (77% of the studies), details about the sample/setting of the study (42% of the studies), and the criteria used for the outcome assessment (23% of the studies).

### Results of individual studies and synthesis of results

The results of all individual studies included in the analyses, for each outcome, and for each comparison across sagittal or vertical skeletal discrepancies can be found in the openly provided dataset of this review [17].

The results of the quantitative data synthesis are provided either through network meta-analyses of direct/indirect evidence (when the transitivity assumption was met) or as pairwise analyses of direct evidence (when violations of the transitivity assumption were judged to exist). The studies contributing with data for each outcome are listed in Supplementary Table S11.

#### Differences according to sagittal skeletal pattern (Class I, Class II, and Class III)

As far as the primary outcome of total pharyngeal volume is concerned, significant differences were seen among all three skeletal Classes (Table 1). Network meta-analysis of 11 studies (1371 patients) indicated that Class II patients showed significantly smaller airway volume by −2256.1 mm^3^ (95% CI −3201.6 to −1310.5 mm^3^) compared to Class I patients, while Class III patients showed significantly greater airway volume by 1098.9 mm^3^ (95% CI 25.4 to 2172.5 mm^3^) compared to Class I patients. In addition, Class III patients had significantly greater overall airway volume by 3355.0 mm^3^ (95% CI 2342.0 to 4368.0 mm^3^) compared to Class II patients.

**Table 1.** Pairwise comparisons of volumes according to sagittal discrepancy based on network meta-analyses of direct & indirect evidence (for outcomes with ≥5 studies) or pairwise meta-analyses of direct evidence (for outcomes with <5 studies). Bold values indicate statistically significant results at the 5% level.

Outcome	Studies (patients)	Analysis	Network *I*^2^ (95% CI)	[Class 2]–[Class 1]	[Class 3]–[Class 1]	[Class 3]–[Class 2]
Total pharynx	11 (1371)	Network	47% (5%, 70%)	**−2256.06** **(−3201.61, −1310.51)**	**1098.93** **(25.41, 2172.45)**	**3354.99** **(2341.99, 4368.00)**
Nasal cavity	3 (184)	Pairwise	–	**−3478.06** **(−6017.22, −938.90)**	–	–
Nasopharynx	24 (2540)	Network	91% (88%, 93%)	–419.32 (–937.46, 98.83)	115.00 (–456.05, 686.06)	534.32 (**−**34.55, 1103.19)
Palatopharynx	11 (913)	Network	96% (94%, 97%)	**–1996.17** **(–3126.12, –866.23)**	715.62 (**−**590.17, 2021.41)	**2711.79** **(1516.90, 3906.68)**
Glossopharynx	10 (853)	Network	81% (70%, 88%)	**−1101.15** **(−1777.29, −425.00)**	194.16 (**−**596.56, 984.89)	**1295.31** **(529.05, 2061.57)**
Oropharynx	19 (2035)	Network	87% (83%, 90%)	**−2586.80** **(−3539.76, −1633.83)**	**1914.20** **(840.40, 2988.00)**	**4501.00** **(3467.93, 5534.07)**
Hypopharynx	6 (480)	Network	93% (89%, 95%)	**−**612.24 (**−**1404.46, 179.98)	**1233.45** **(434.57, 2032.33)**	**1845.68** **(1051.07, 2640.30)**
Intraoral	4 (229)	Pairwise	–	530.02 (**−**560.90, 1620.94)	**1351.33** **(296.08, 2406.58)**	821.31 (**−**221.45, 1864.07)
Nasal cavity and nasopharynx	3 (94)	Pairwise	–	**−**804.32 (**−**4567.38, 2958.75)	**−**2760.58 (**−**8567.17, 3046.02)	**−**1956.26 (**−**6651.99, 2739.47)
Naso- and Oropharynx	4 (268)	Pairwise	–	**−2285.72** **(−4441.33, −130.11)**	2785.65 (**−**342.17, 5913.48)	**5071.37** **(2025.30, 8117.44)**
Oro- and hypopharynx	21 (2001)	Network	95% (94%, 96%)	**−1489.17** **(−2948.17, −30.17)**	**2377.57** **(957.26, 3797.88)**	**3866.74** **(2441.24, 5292.24)**

CI, confidence interval.

Considerable differences were seen in the volumes of the different airway compartments among the three skeletal Classes. The nasal cavity volume was compared only between Class II and Class I patients (pairwise meta-analysis; three studies; 184 patients) where it was found to be significantly smaller for the former (difference −3478.1 mm^3^; 95% CI −6017.2 to −938.9 mm^3^).

No statistically significant differences were found among skeletal Classes regarding the nasopharynx volume.

The volume of the palatopharynx was assessed in a network of 11 studies (913 patients) that found that Class II patients had a significantly smaller palatopharynx than Class I patients by −1996.2 mm^3^ (95% CI −3126.1 to −866.2 mm^3^). However, Class III patients had a significantly larger palatopharynx than Class II patients by 2711.8 mm^3^ (95% CI 1516.9 to 3906.7 mm^3^).

The volume of the glossopharynx was assessed in a network of 10 studies (853 patients) that found that Class II patients had a significantly smaller glossopharynx than Class I patients by −1101.2 mm^3^ (95% CI −1777.3 to −425.0 mm^3^). However, Class III patients had a significantly larger palatopharynx than Class II patients by 1295.3 mm^3^ (95% CI 529.1 to 2061.6 mm^3^).

The volume of the oropharynx was assessed in a network of 19 studies (2035 patients) that found significant differences across all three Classes. Specifically, Class II patients had a smaller oropharynx than Class I patients (difference −2586.8 mm^3^; 95% CI −3539.8 to −1633.8 mm^3^), while Class III patients had a larger oropharynx than both Class I patients (difference 1914.2 mm^3^; 95% CI 840.4 to 2988.0 mm^3^) and Class II patients (difference 4501.0 mm^3^; 95% CI 3467.9 to 5534.1 mm^3^).

The volume of the hypopharynx was assessed in a small network of six studies (480 patients) that found that Class III patients had a larger hypopharynx than both Class I patients (difference 1233.5 mm^3^; 95% CI 434.6 to 2032.3 mm^3^) and Class II patients (difference 1845.7 mm^3^; 95% CI 1051.1 to 2640.3 mm^3^).

The volume of the intraoral cavity was assessed in four studies (229 patients) and pairwise meta-analyses indicated that Class III patients had a larger volume than Class I patients (difference 1351.3 mm^3^; 95% CI 296.1 to 2406.6 mm^3^).

Some studies assessed the combined volumes from two airway compartments. Three studies assessed the nasal cavity combined with the nasopharynx and found no significant differences among any of the three Classes. The volume of the nasopharynx combined with the oropharynx was assessed in four studies (268 patients) and pairwise meta-analyses indicated that Class II patients had smaller volumes than Class I patients (difference −2285.7 mm^3^; 95% CI −4441.3 to −130.1 mm^3^) and Class III patients had larger volumes than Class II patients (difference 5071.4 mm^3^; 95% CI 2025.3 to 8117.4 mm^3^). Finally, the volume of the oropharynx together with the hypopharynx was assessed by a network of 21 studies (2001 patients) that found significant differences among all three Classes: Class II patients had smaller volume than Class I patients (difference −1489.2 mm^3^; 95% CI −2948.2 to −30.2 mm^3^), while Class III had larger volume both than Class I (difference 2377.6 mm^3^; 95% CI 957.3 to 3797.9 mm^3^) and Class II patients (difference 3866.7 mm^3^; 95% CI 2441.2 to 5292.2 mm^3^).

#### Differences according to the vertical skeletal pattern (normodivergent, hypodivergent, and hyperdivergent)

As far as the different vertical skeletal patterns are concerned, few statistically significant differences were seen (Table 2). For the primary outcome of total pharynx volume, analysis of a network of seven studies (1066 patients) showed that hyperdivergent patients had smaller airway volumes than hypodivergent patients (difference −1957.6 mm^3^; 95% CI −3063.3 to −851.8 mm^3^).

**Table 2.** Pairwise comparisons of volumes according to vertical configuration based on network meta-analyses of direct and indirect evidence (for outcomes with ≥5 studies) or pairwise meta-analyses of direct evidence (for outcomes with <5 studies). Bold values indicate statistically significant results at the 5% level.

Outcome	Studies (patients)	Analysis	Network *I*^2^ (95% CI)	[Hypodivergent]–[normodivergent]	[Hyperdivergent]–[normodivergent]	[Hyperdivergent]–[hypodivergent]
Total pharynx	7 (1066)	Network	36% (0%, 69%)	1080.83 (**−**14.51, 2176.17)	**−**876.75 (**−**1921.53, 168.04)	**−1957.58** **(−3063.32, −851.83)**
Nasal cavity	1 (34)	Pairwise	–	**−**1016.45 (**−**8092.12, 6059.22)	**−**1002.08 (**−**6317.90, 4313.74)	14.37 (**−**7193.24, 7221.98)
Nasopharynx	11 (1424)	Network	86% (79%, 91%)	**−**370.54 (**−**1054.86, 313.78)	-436.15 (**−**998.52, 126.23)	**−**65.60 (**−**741.78, 610.57)
Oropharynx	3 (669)	Pairwise	–	**−**353.01 (**−**1261.75, 1967.77)	**−1716.77** **(−3296.42, −137.12)**	**−**1413.52 (**−**3041.78, 214.75)
Hypopharynx	2 (239)	Pairwise	–	17.55 (**−**1226.92, 1262.02)	**−**299.32 (**−**1221.74, 623.09)	118.07 (**−**1143.42, 1379.55)
Naso- & Oropharynx	2 (215)	Pairwise	–	**−**2204.87 (**−**10236.92, 5827.18)	**−**1885.01 (**−**7533.94, 3763.92)	**−**2565.68 (**−**10516.27, 5384.90)
Oro- & hypopharynx	9 (1196)	Network	24% (0%, 58%)	413.53 (**−**520.47, 1347.54)	**−**455.44 (**−**1315.97, 405.08)	**−**868.97 (**−**1775.48, 37.53)

CI, confidence interval.

No statistically significant differences between vertical divergency groups were seen for the nasal cavity, the nasopharynx, the glossopharynx, the oropharynx, the hypopharynx, and combinations of naso- & oropharynx or oro- & hypopharynx.

Limited pairwise meta-analyses indicated that hyperdivergent patients had smaller oropharynx volumes than normodivergent patients (three studies; 669 patients; difference −1716.8 mm^3^; 95% CI −3296.4 to −137.1 mm^3^).

### Exploration of inconsistency and transitivity

No substantial evidence for inconsistency between direct and indirect evidence was found for most comparisons among the different sagittal patterns (Supplementary Table S12). The only exceptions were the network for the nasopharynx and the oro- & hypopharynx, where differences between direct/evidence were seen. Post-hoc comparisons between results from the network and pairwise meta-analyses (Supplementary Table S13) showed only small differences both in terms of statistical significance and effect magnitude (with the network meta-analysis being more conservative than the pairwise meta-analysis). Similarly, for comparisons among different vertical patterns inconsistency was found for the total pharyngeal volume, but comparisons network and pairwise meta-analyses showed robustness of the results.

The distributions of all potential effect modifiers were relatively similar among the various comparisons for all networks (Supplementary Figs. S11–S14) and therefore no serious threats to the transitivity assumption were judged to exist.

### Risk of bias across studies and results of additional analyses

The credibility of evidence provided by the analyses was assessed with the CINeMA approach and was found to be in all instances ‘very low’. The main reasons for downgrading were the potential for a high risk of bias due to weak study designs with methodological issues, heterogeneity, and in some instances incoherence.

The assessment of reporting biases through small-study effects for meta-analyses with at least 10 studies can be seen in Supplementary Figs. S15–S21 that show the contrast-adjusted funnel plots and the results of Egger’s asymmetry test. Hints of funnel plot asymmetry were found for the sagittal effects on the nasopharynx, the sagittal effects on the oro- and hypopharynx, and the vertical effects on the nasopharynx. For these outcomes, post hoc sensitivity analyses according to study precision (inversely to their standard error) indicated robustness of the results (Supplementary Table S14). For the two instances, where significant differences were seen between most-precise and least-precise studies, both subgroups gave statistically non-significant differences and therefore no threat to the results’ validity was identified.

Pre-specified network subgroup or meta-regression analyses were performed to identify if patient age-group, mean age, or patient sex could influence the results of the analyses (Supplementary Table S15). Significant interactions with the age of the included patients were seen—both according to the patient’s mean age and according to the age categories—while patient sex was a non-significant effect modifier. Subgroup network meta-analyses according to whether the study included children, adults, or mixed populations (Table 3) indicated that reduced nasopharyngeal volume was seen mostly for children with Class II pattern (compared to Class I), but not for adults with Class II. On the other hand, differences in the volume of the oro- and hypopharynx between the three Classes were seen both for studies with adult patients and mixed (children/adult) populations.

**Table 3.** Subgroup analysis by age group is given as network meta-analyses of direct and indirect evidence (≥5 studies) or pairwise meta-analyses of direct evidence (<5 studies). Bold values indicate statistically significant results at the 5% level.

Dimension	Outcome	Age-group	Studies (patients)	Network *I*^2^ (95% CI)	[Class 2]–[Class 1]	[Class 3]–[Class 1]	[Class 3]–[Class 2]
Sagittal	Nasopharynx	Children	9 (1638)	89% (83%, 93%)	**−772.49** **(−1457.95, −87.03)**	**−**511.25 (**−**1393.24, 370.73)	261.23 (**−**601.62, 1124.09)
		Adults	8 (2240)	76% (58%, 86%)	65.04 (**−**490.23, 620.32)	184.37 (**−**349.39, 718.13)	119.32 (**−**443.64, 682.29)
		Mixed	7 (1962)	94% (92%, 96%)	**−**153.40 (**−**2101.94, 1795.15)	1019.98 (**−**1044.96, 3084.93)	1173.38 (**−**888.16, 3234.92)
Sagittal	Oro- and hypopharynx	Children	5 (649)	94% (91%, 97%)	**−**1238.82 (**−**4846.44, 2368.80)	1956.43 (**−**1376.16, 5289.02)	3195.25 (**−**116.78, 6507.27)
		Adults	13 (3679)	91% (88%, 94%)	**−2379.11** **(−4173.33, −584.89)**	1676.26 (**−**103.62, 3456.14)	**4055.37** **(2254.90, 5855.84)**
		Mixed	2 (237)	–	605.74 (**−**1206.68, 2418.16)	**6792.00** **(4991.24, 8592.76)**	**5938.70** **(4201.92, 7675.48)**
Vertical	Nasopharynx	Children	0 (0)	–	–	–	–
		Adults	5 (1464)	59% (6%, 82%)	**−**703.65 (**−**1814.40, 407.10)	**−**100.19 (**−**989.93, 789.56)	603.46 (**−**487.18, 1694.11)
		Mixed	6 (2150)	90% (84%, 94%)	494.00 (**−**365.19, 1353.20)	**756.45** **(25.11, 1487.78)**	262.44 (**−**623.07, 1147.96)

CI, confidence interval.

## Discussion

### Evidence in context

The present systematic review critically appraised and synthesized evidence from human clinical studies on upper airway volume differences according to their sagittal or vertical skeletal craniofacial configuration. Meta-analyzed data from 56 studies indicated that skeletal morphology is significantly associated with differences in the volume of the upper airways. Specifically, Class II was consistently associated with smaller total airway volume than Class I, which was seen in specific compartments, namely the nasal cavity, the palatopharynx, and the glossopharynx (with the two latter comprising the oropharynx). On the contrary, Class III was associated with increased total airway volume compared to Class I, which was seen mostly in the oropharynx, the hypopharynx, and the oral cavity. As far as the vertical dimension is concerned, hyperdivergent patterns were consistently associated only with decreased oropharyngeal volumes compared to normodivergent patterns.

Evidence of a possible relationship between craniofacial morphology and function has long been the subject of orthodontic research. Early data on primates by Harvold et al. [28]. indicated that experimental nasal obstruction led to differences in soft tissues such as the lips and tongue, as well as in the recruitment and electromyographic activity of the genioglossus, geniohyoid, digastric, temporalis, median, and lateral pterygoid muscles. These had diverse effects including tongue protrusion, lip elevation, lowering, and advancement of the mandible, all of which led to the development of malocclusions as an effort to maintain patency of the upper airways. Similarly, experimental nasal obstruction in human adults led to head extension and lower positioning of the mandible, as well as decreased activity of the postcervical and anterior temporalis muscles and increased activity of the suprahyoid muscles [29]. Among children, normal breathing might often be impaired by enlarged adenoids and tonsils, which might lead to anterior-inferior tongue posture accompanied by a retrognathic and posteriorly inclined mandible, increased anterior face height, head extension, and lowering of the hyoid bone causing mouth breathing, especially during the night [30, 31]. Evidence however still remains inconclusive as to whether such a relationship exists and especially what is its direction—i.e. whether impaired breathing might affect skeletal morphology or whether a deviation in morphology might lead to impaired breathing.

Morphological differences have been reported between healthy patients and patients with several breathing disorders. As far as breathing patterns are concerned (nose-breathers versus mouth-breathers), characteristics observed in mouth-breathing children include mandibular retrusion, posterior rotation of the mandibular plane, increased anterior face height, decreased posterior face height, elevated hyoid bone position, and significantly smaller nasopharyngeal airway dimensions [32–34]. Habitual snoring and upper airway resistance syndrome in children has been associated with posterior rotation of the mandibular plane, decreased soft palate thickness, and decreased inferior pharyngeal space [35]. More severe breathing disorders in children, specifically obstructive sleep apnea (OSA), have been associated with an increased Class II skeletal configuration (increased ANB angle), increased vertical face dimensions, reduced intercanine distances, and decreased superior and inferior pharyngeal dimensions [35, 36]. Among adults, OSA has been associated with an inferiorly placed hyoid bone, increased anterior face height, and reduced pharyngeal airway space [10]. Nevertheless, it is important to stress that the pathophysiology of OSA is multifactorial, including among others obesity, increased fat deposition in the pharyngeal pads, and upper airway collapse due to not only morphological craniofacial abnormalities, but also nocturnal fluid shift from the legs to the upper body, decreased upper airway dilator muscle activity during sleep, critical pressure inside the upper airways becoming less negative rendering the pharynx unable to remain open, and ventilatory chemoreflex control instability [37]. As a result, the volume of the upper airways as an isolated parameter should only be evaluated in conjunction with other risk factors for OSA patients, as it does not correlate strongly with objective OSA symptoms such as the Apnea-Hypopnea-Index (AHI) [38]. Some data indicate that 3D craniofacial morphology, and specifically (i) the length of the retropalatal airway, (ii) the length of the mandibular body, and (iii) the narrowest cross-sectional areas of the airway, are associated with OSA severity through AHI and could be used in both diagnosis and treatment planning [39, 40] but CBCT does not formally belong in the gold standard diagnostics for OSA.

Strong heritability has been reported for specific aspects of craniofacial morphology, as evaluated by two-dimensional imaging methods. Data from monozygotic twins have identified an inverse association between nasopharyngeal airway size and the mandibular gonial angle, while linear measurements of the mandibular ramus and body were not found to be associated with the nasopharyngeal airway [41]. Several morphological characteristics of the mandible, including mandibular length, gonial angle, SNB angle, anterior face height, and mandibular plane angle, have been shown to be under strong genetic control [42, 43]. Similarly, in familial OSA, decreased mandibular body length is accompanied by decreased pharyngeal depth at the level of the gonial angle [44]. Given that certain compartments of the upper airway such as the palatopharynx and the hypopharynx have shown higher heritability traits [45], establishing a relationship between the upper airways volumes and the craniofacial pattern would allow for an enhanced orthodontic diagnostic process, or even the consideration of therapeutical approaches that might positively influence upper airway dimensions [3, 4, 46–48].

The accuracy of the performed measurements can also be affected by the segmentation methods and the experience of the person manually performing them, with more consistent performance shown by educated and experienced examiners [49, 50]. Several fully- or semi-automatic methods for airway segmentation using three- or two-dimensional convolutional neural networks have been reported [51–55] but require increased processing power and involve subjectively chosen thresholds [56]. Comparisons between these novel approaches with manual segmentation as the gold standard have indicated that absolute differences exist, although high reliability is seen (intraclass correlation coefficients between 0.90 and 0.99)—bringing thereby other factors into focus such as costs, complexity, learning curve, and compatibility with operating systems [57]. Additionally, concerns regarding radiation safety exist; however, there is lack of consistent standards on the values of acceptable doses as well as of the accurate equivalence of doses between the standard radiographs used for orthodontic diagnosis and treatment (orthopantomogram and cephalograms) over CBCTs with manufacturers not providing clear data for comparisons.

The majority of the studies included in the present review provided very limited information about the age of included participants and/or did not analyze data according to age subgroups. It has been well documented that the upper airway volume undergoes a significant increase between the ages of 8 and 18 years [58] with a simultaneous increase in the nasal airflow rate and cross-sectional area from 7 to 24 years of age [59]. These differences have further been confirmed by data showing that adults have considerably larger airway volumes with more elliptical shape (increased width/depth ratio) that are less uniform and less compact than children while a significant increase in airway volume is seen during the transition from the primary to the permanent dentition [60].

Similarly, the often-seen incomplete reporting of patient sex among the included studies precluded an adequate assessment of sexual dimorphism in the relationship between skeletal configuration and upper airway volume. Patient sex has been reported to have an impact, with male patients showing greater upper airway volumes than female patients, especially in the oropharyngeal compartment [61], while these sex-specific changes seem to emerge mainly during puberty [62]. All the above underline the necessity for studies that apply and report clearly on patient demographics, account for these parameters in the analyses, and ideally provide their dataset openly to improve data use [63].

### Strengths and limitations

The strengths of the present systematic review include its pre-registered protocol [64], an exhaustive literature search, the systematic appraisal of the studies’ internal validity, the combination of direct and indirect data via network meta-analyses, appraisal of the quality of meta-evidence using the CINeMA approach [26] and the transparent provision of the study’s dataset [17]. Finally, contrary to the majority of the existing studies that are based on two-dimensional methods, the present review synthesized 3D imaging data, which are more precise in evaluating the complex upper airway morphology [65].

Nevertheless, this review has several limitations that should be considered. Included cross-sectional studies presented high risk of bias due to issues with (i) justification of their sample size, (ii) the identification and the accounting of confounding factors in the analyses (e.g. maxillary and/or mandibular assessment for prognathism and/or retrognathism so that the ANB angle is not the sole indicator for the sagittal categorization into Class I, II, and III, vertical sub-categorization for the sagittal groups and vise-versa), (iii) the non-unanimous adoption of well-accepted upper airway compartment boundaries, (iv) the lack of method error assessment, (v) lack of information on whether the upper airway CBCT assessor was blinded to the values of the craniofacial pattern, (vi) inappropriate statistical analyses, and (vii) incomplete reporting of sample characteristics including the cut-off cephalometric values used for the sagittal (Class I, II, III) and vertical (normo-, hypo- and hyperdivergent) categorization in groups (viii) incomplete reporting of the outcomes with numerous studies reporting only correlations between upper airway and cephalometric parameters, and (ix) mixed samples regarding age.

In the included studies in the present review, upper airway volume image acquisition and measurements were performed using various methods and software. In most of the studies, measurements were performed on data obtained from CBCT while CT was used in four studies and MRI in one study. CBCT is considered a more preferable imaging method to multi-detector CT due to the decreased radiation exposure with both methods being considered accurate in measuring the upper airways; however, the accuracy depends on the brand of the equipment, the parameters used during image acquisition and the segmentation technique, including the software programs, with the methodological differences amongst those accuracy studies leading to variable results [66]. MRI is a more appealing alternative for upper airway imaging due to the complete absence of radiation with both manual and automatic segmentation methods found to be highly accurate [67].

In addition, the study design of most of the included studies was retrospective cross-sectional, which cannot prove a cause-and-effect relationship between airway dimensions and sagittal/vertical skeletal discrepancies (or which was observed first)—especially without adequate controlling for potential confounders.

## Conclusion

According to the results of the present systematic review on the relationships between skeletal configuration and upper airway volume assessed on 3D imaging, Class II patients were found to have smaller upper airway volumes compared to Class I patients, and Class III patients tend to have greater upper airways volumes compared to Class I and Class II patients. Additionally, hyperdivergent patients were found to have smaller oropharyngeal volumes compared to normodivergent patients. The clinical relevance of these findings and how they relate to the breathing function of patients remains to be seen.

## Supplementary Material

cjae010_suppl_Supplementary_Tables_S1-S4_S6-S15_Figures_S1-S21

cjae010_suppl_Supplementary_Tables_S5

## Data Availability

Data are openly available through Zenodo (DOI: 10.5281/zenodo.10042587).
