# Supplementary material for: Do patients with different craniofacial patterns have differences in upper airway volume? A systematic review with network meta-analysis
Source: Eur J Orthod. 2024 Mar 25;46(2):cjae010. doi: 10.1093/ejo/cjae010 (PMC10962399; doi:10.1093/ejo/cjae010)
Supplement: cjae010_suppl_Supplementary_Tables_S1-S4_S6-S15_Figures_S1-S21 [file cjae010_suppl_supplementary_tables_s1-s4_s6-s15_figures_s1-s21.docx]

**SUPPLEMENTARY MATERIAL**

**Do patients with different craniofacial patterns have different volumes of the upper airways? A systematic review with network meta-analysis.**

**Supplement 1.** Additional review methods and deviations from protocol.

**Additional methods**

- In case of data not (or inadequately) reported in the published paper, the corresponding authors were contacted to request this data in either aggregate or individual-patient-level format.
- Whenever needed, appropriate data transformations were performed to prepare data for synthesis. This includes: pooling similar study-arms from multi-arm studies to avoid clustering; converting non-parametric to parametric data; calculating descriptive statistics from raw datasets provided by the corresponding authors.
- Correspondence with authors of retrieved studies:
  - Emailed all corresponding authors of the following studies that reported correlations.
    - Anandarajah 2017
    - Chan 2020
    - Claudino 2013
    - Diwakar 2021
    - Elagib 2022
    - Gong 2018
    - Indriskone & Jakobsone 2015
    - Miranda-Viana 2021
    - Nath 2021
    - Vuong & Kang 2021
    - Yanagita 2017
  - Only the following responded and sent the requested data.
    - Claudino 2013
    - Elagib 2022
    - Indriskone & Jakobsone 2015
    - Miranda-Viana 2021
  - The corresponding author of Anandarajah 2017 responded and refused to share anonymised data

**Deviations from protocol**

There were no deviations from the protocol.

**Supplementary Table 1.** Eligibility criteria used for the study selection.

| **Category** | **Inclusion criteria** | **Exclusion criteria** |
| --- | --- | --- |
| Participant characteristics | Healthy humans of any age, sex, ethnicity with different craniofacial morphology (anteroposterior and vertical) other than ideal, meaning Class II and Class III anteroposterior jaw relationship as well as hypodivergent and hyperdivergent jaw relationship as per the divergence (vertical) development pattern.  Assessment of the upper airways using three-dimensional technology such as conventional computed tomography (CT), cone-beam computed tomography scans (CBCTs), magnetic resonance imaging (MRI), or ultrasonography. | Patients with: developmental defects (syndromes, clefts); craniofacial anomalies; obstructive sleep apnea; sleep-related disorders; other pathology. |
| Exposure | Patients with anteroposterior skeletal anomalies (Class II or Class III) or vertical skeletal deviations (hypodivergent or hyperdivergent). | Assessment of the upper airways using two-dimensional technology (eg. lateral cephalometry). |
| Comparison | Subjects with normal sagittal (anteroposterior) craniofacial morphology/pattern, meaning Class I anteroposterior jaw relationship and normal vertical divergence of the maxillomandibular complex (normodivergent). | Comparison of treatment effects. |
| Outcome | **Primary:** Total upper airway volume.  **Secondary:** Volume of the individual compartments of the upper airway (nasal cavity, nasopharynx, palatopharynx, glossopharynx, oropharynx, hypopharynx, total pharynx). | Studies that do not provide the outcomes of interest. |
| Study design | Randomized / non-randomized prospective / retrospective clinical (cross-sectional / cohort) studies. | Case reports/series, review articles, unsupported opinions, interviews & newsletters, commentaries, conference abstracts, replies to the editor/author, non-clinical studies (in vitro, ex vivo, in silico, etc), studies with only one group (only Class I, only Class II, only Class III / only normodivergent, only hypodivergent, only hyperdivergent) and systematic reviews (after checked for additional studies), animal studies. |

**Supplementary Table 2.** Search Strategy.

| **Nr** | **Database** | **Search strategy used** | **Extend of search** | **Sum** |
| --- | --- | --- | --- | --- |
| 1 | MEDLINE (via PubMed) | ((((airway* OR "nasal cavity" OR nose OR nasopharyn* OR palatopharyn* OR velopharyn* OR retropalatal OR glossopharyn* OR retroglossal OR oropharyn* OR hypopharyn* OR pharyn* OR oronasal) AND (volum* OR cross-section* OR width OR depth OR height OR length OR morph* OR position*)) AND ((("cone beam" OR digital OR ct) AND (tomograph* or radio* OR scan* OR imag*)) OR (CBCT OR CBVT OR "computed tomography" OR "computerized tomography" OR "magnetic resonance imag*" OR DVT OR MRI OR 3D OR three-dimens* OR ultraso* OR "computational fluid dynamic*" OR CFDs OR "CFD analysis"))) AND (craniofac* OR dentocraniofac* OR maxillofac* OR dentofac* OR skelet* OR malocclusion* OR grow* OR prognath* OR retrognath* OR hypodivergen* OR hyperdivergen* OR normodivergen* OR diverg* OR brachyfac* OR dolychofac* OR "short face" OR "long face" OR "Angle Class*")) NOT (tumor* OR carc* OR cancer* OR malign* OR COVID-19 OR SARS-CoV-2 OR pulmonary OR syndrome*) | In: Title, Abstract  Limit: humans | 397 |
| 2 | Embase | Same as Pubmed | In: All fields; Limit: humans | 693 |
| 3 | Web of Science | Same as Pubmed | In: All fields; Timespan: All years; Language: All languages, All document types | 1811 |
| 4 | ScielLo (via Web of Science) | Same as Pubmed | In: Topic | 18 |
| 5 | Proquest (Dissertations and Thesis Global) | Same as Pubmed | In: Abstract  Manuscript type: Doctoral Dissertations | 3 |
| 6 | Scopus | Same as Pubmed | In: Title, Abstract, Key words | 1831 |
| 7 | Cochrane Library | Same as Pubmed | Title Abstract Keyword  (Word variations have been searched) | Cochrane trials: 91  Cochrane reviews: 2 |
| 8 | Virtual Health Library | Same as Pubmed | In: Title, Abstract, Subject | LILACS: 16; BBO-DENTISTRY: 6; IBECS: 6; CUMED: 1; bioRxiv: 1; SecEst.Saude SP: 1 / TOTAL (unique): 27 |
|  |  |  | TOTAL | 4873 |

Supplementary Table 3. Data extraction form.

| **Item** |
| --- |
| Study Name (author, year, clinical setting): |
| Study design:  Purpose of study (if stated):  **Ethics approval** (Yes/No/Unclear)  **Funding** (if stated): |
| **PATIENTS GROUPS inclusion & exclusion criteria, demographics (age, sex)**  Inclusion Criteria  Exclusion criteria  **Sagittal skeletal relationships**  Class I  Class II  Class III  **Vertical skeletal relationships**  Normodivergent  Hypodivergent  Hyperdivergent |
| **INTERVENTIONS (treatment & comparison group)**  **Type of 3D analysis (CT, CBCT, MRI, Ultrasound etc)**  Patient positioning. STANDING/SUPINE  Instructions during image acquisition. YES/NO  Equipment: type of scanner (eg NewTom)  Software used for analysis (eg. Dolphin) |
| **OUTCOMES**  **Volume Measurements**  Upper airway (total):  Upper airway (compartments):   - Nasal cavity - Nasopharynx - Palatopharynx - Glossopharynx - Oropharynx - Hypopharynx - Pharynx (total) |
| **Other Measurements:** |
| **Statistical methods** (appropriateness; if relevant) |
| **Error Assessment:** |
| **Other comments:** |

.

**Supplementary Table 4.** Domains / questions of the customized tool used for the assessment of internal validity / risk of bias. Each question was answered with yes, unclear, or no.

| **Nr** | **Question** |
| --- | --- |
| 1 | Were the aims/objectives of the study clear? |
| 2 | Were the criteria for inclusion in the sample clearly defined? |
| 3 | Were the study subjects and the setting described in detail (time, location, demographics)? |
| 4 | Was the sample size justified (sample size calculation)? |
| 5 | Were objective, standard criteria used for measurement of the condition (cut-off values for cephalometric parameters used to categorize the patients in the sagittal and vertical groups)? |
| 6 | Were confounding factors identified (presence of pathology, adenoids, tonsils, infection, nose or mouth breathing, baseline obstructive sleep apnea assessment such as with questionnaires, age and maturation, sex, body mass index, transversal discrepancies, vertical sub-categorization for the sagittal groups and vise-versa, conditions during image acquisition like providing patients with instructions for breathing & tongue posture, automatic or manual segmentation and thresholds, etc.)? |
| 7 | Were strategies to deal with confounding factors stated (analyses adjusted for confounders)? |
| 8 | Were the outcomes measured in a valid way (valid and accepted boundaries used also in the medical terminology)? |
| 9 | Were the outcomes measured in a reliable way (experience and/or training and/or calibration of the one-s measuring the airways)? |
| 10 | Method error calculation performed? |
| 11 | Were the assessors blinded to the groups (the one-s measuring the airways being aware of the cephalometric values)? |
| 12 | Was appropriate statistical analysis used? |
| 13 | Is it clear what was used to determined statistical significance and/or precision estimates (p values, confidence intervals, etc.)? |
| 14 | Were all specified outcome data adequately described and reported (incomplete reporting)? |

Supplementary Table 6. Study Design, equipment and method used for upper airway image acquisition, software used for upper airway assessment, patient demographics, inclusion, and exclusion criteria.

| **Study** | **Study design**  **Institution** | **Upper airway image acquisition (scanner and software), patient position & instructions during image acquisition** | **Class I**  **Normodivergent (AV)**  **Hypodivergent (LA)**  **Hyperdivergent (HA)** | **Class II**  **Normodivergent (AV)**  **Hypodivergent (LA)**  **Hyperdivergent (HA)** | **Class III**  **Normodivergent (AV)**  **Hypodivergent (LA)**  **Hyperdivergent (HA)** | **Vertical subgroups**  **Normodivergent (AV)**  **Hypodivergent (LA)**  **Hyperdivergent (HA)** | **Inclusion criteria** | **Exclusion criteria** |
| --- | --- | --- | --- | --- | --- | --- | --- | --- |
| Abdelkarim et al. 2012 | Retrospective; Uni; USA | CBCT / i-CAT (Xoran, Imaging Sciences, Hatfield, PA) / Dolphin 3D imaging software version 11.0 / Upright (sitting) / Instructions: YES | Normal mandible  76≤SNB≤82  N: 46  Males: 23  Females: 23 | Retrognathic mandible  SNB<76  N: 51  Males: 25  Females: 26 | Prognathic mandible  SNB>82  N: 31  Males: 17  Females: 14 | No categorization | Age: 16 - 35 years | Craniofacial deformities and syndromes |
| Aby et al. 2020 | Retrospective; College; IND | CBCT / Carestream Software (Carestream 9300) / Dolphin 3D software / Upright / Instructions: YES | N: 30 | N: 30 | No sample | No categorization | Age: 14 - 20  CBCT in occlusion | CBCT with biteblock, previous orthodontic treatment, previous surgery, congenital anomalies (Cleft lip and palate), enlarged tonsils or adenoids |
| Alhammadi et al 2021 | Retrospective  Cross-sectional; College; SAU | CBCT / i-CAT (Imaging Sciences International, Hatfield, PA, USA) / Dolphin 11.5 software / Upright (sitting) / Instructions: YES | 0<ANB<5°  N: 50  Males AV: 14  Age: 22.24 (3.03)  Age range: 17.42-27.75  Males HA: 14  Age: 20.09 (2.17)  Age range: 17.42-23.92  Females AV: 16  Age: 22.82 (2.1)  Age range: 18.50-25.5  Females HA: 16  Age: 20.46 (1.91)  Age range: 17.42-23.92 | ANB> 5°  N: 50  Males AV: 15  Age: 20.01 (1.86)  Age range: 17.22-23.5  Males HA: 12  Age: 19.29 (1.35)  Age range: 17.58-21.75  Females AV: 15  Age: 20.19 (1.75)  Age range: 17.75-24.92  Females HA: 18  Age: 19.92 (1.3)  Age range: 18.58-22.75 | No sample | **Normodivergent**  N: 60  SN-MP: 32° (5°)  **Hyperdivergent**  N: 60  SN-MP>37° | Above 17 years old  Normal nasal breathing  Normal body mass index (BMI) | Previous orthodontic treatment, major craniofacial surgery |
| Alves Jr. M et al. 2012 | Retrospective; Uni; BRA | CBCT / i-CAT (Imaging  Sciences International, Hatfield, PA, USA) / Dolphin Imaging  software, version 11.0 / Upright (sitting) / Instructions: YES | ANB: 2-5°  N: 25  Males: 14  Females: 11  Age total sample Class I and Class II 9.16 (0.64)  Age range: 8-10 | ANB>5°  N: 25  Males: 13  Females: 12  Age total sample Class I and Class II 9.16 (0.64)  Age range: 8-10 | No sample | No categorization | Craniocervical angulation 90-110ᵒ | Obvious hyperplasia of  tonsils and adenoids, tonsillectomy or adenoidectomy, OSA,  symptoms of upper respiratory infection, pharyngeal pathology |
| Alves PMV et al. 2008 | Retrospective; Uni; BRA | 3-D CT (spiral CT) / No further info | No sample | N=30  Males (N=15)  Age: 17.3 (2.0)  ANB: 5.5° (2.5°)  Females (N=15)  Age: 18.0 (1.8)  ANB: 6° (2°) | N=30  Males (N=15)  Age: 18.2 (1.2)  ANB: -3.5° (1.2°)  Females (N=15)  Age: 17.5 (2.0)  ANB: -3.2° (2.1°) | No categorization | Skeletal AP malocclusion | Vertical discrepancies, genetic syndromes, congenital deformities, sleep disorder snoring, upper airway disease, adenoidectomy, pathology in the pharynx, orthognathic treatment |
| Anandarajah et al. 2017 | Retrospective; Pract; AUS | CBCT / i-CAT (Next Generation CBCT machine) / Dolphin Imaging software, version 11.5 / Upright (sitting) / Instructions: YES | Total sample  ANB: 4.1 (2.6), ANB range: -2.1°-8.9°  SN-MP: 34.3° (5.4)°, SN-MP range: 23°-48° | | | No categorization | Healthy children  Age: 8-16 years of age  Biting in centric occlusion | Previous orthodontic treatment and/or orthognathic surgery, adeno-tonsillectomy, syndromes, upper airway pathology, OSA, movement artefact, swallowing during scan acquisition |
| Bozzini et al. 2018 | Prospective; Uni; BRA | CBCT / i-Cat® (version 11.5, Imaging Sciences International, Hatfield, PA, USA) / Dolphin 3D software / Upright (sitting) / Instructions: YES | N: 25  Male: 11  Female: 14  Age: 26.0 (3.0)  Age range: 22-32 | N/A | N: 25 (Surgical cases)  Male: 12  Female: 13  Age: 26.1 (6.1)  Age range: 18-41 | No categorization | Systemically healthy adults, permanent dentition, good periodontal conditions | Previous facial surgery or trauma, morbid obesity, bronchial asthma, tonsillectomy, adenoidectomy and/or uvulopalatoplasty,  craniofacial abnormalities |
| Brasil et al. 2016 | Retrospective; Uni; BRA | CBCT / i-CAT unit (Imaging Sciences, Hatfield, Pa) / Insight ITK-SNAP version 2.4.0 / Upright (sitting)  Instructions: No info | No sample | N:50  ANB: 5.2° (2.6)  MP angle: 22.9° (8.55)° | N:24  ANB: -2.8° (4.35)°  MP angle: 18.4° (14.58)° | **Normodivergent**  N: 19  FH-MP: 24.8° (3.75)°  **Hypodivergent**  N:39  FH-MP: 17.4° (6)°  **Hyperdivergent**  N:16  FH-MP: 32.3° (6.18)° | 18 - 56 years old | Previous orthognathic surgery, radiographic signs of upper airway pathology, cervical spine that was not upright, and CBCT images without nasion or the base of the third cervical vertebra region |
| Brito et al. 2019 | Retrospective; Uni; BRA | CBCT / KODAK 9500 Cone Beam 3D System; Carestream Health, Rochester, N.Y.) / Dolphin Imaging software version 11.7 / Upright (standing) / Instructions: YES | 0≤ANB≤4  **Normodivergent**  N:24  Age: 15.50 (2.62)  **Hypodivergent**  N:23  Age:17.17 (3.37)  **Hyperdivergent**  N:22  Age: 17.45 (3.69) | ANB>4  **Normodivergent**  MaxP: N:20  Age:14.55 (2.58)  MandR: N: 24  Age: 16.37 (3.53)  **Hypodivergent**  MaxP: N:20  Age: 16.19 (3.85)  MandR: N:19  Age: 15.15 (2.75)  **Hyperdivergent**  MaxP: N: 22  Age: 14.86 (2.41)  MandR: N: 23  Age:16.08 (3.65) | No sample | **Normodivergent**  N :68  19<FH-MP<30  **Hypodivergent**  N :62  FH-MP≤19  **Hyperdivergent**  N :67  FH-MP≥30 | 13 – 23 years  Skeletal Class I or Class II malocclusions  Good general health.  Craniocervical angle 90.8° - 110.8° | Previous orthodontic treatment, syndromes, Class I bimaxillary protrusion, scans with artifacts |
| Cabral et al. 2016 | Retrospective; Uni; BRA | CBCT / i-CAT unit (Imaging Sciences International, Hatfield, PA) / Dolphin Imaging (version 11.5) / Upright (sitting) / Instructions: YES | N: 21  Total sample median age:  27.4 years | N:21  Total sample median age:  27.4 years | No sample | No categorization | Over 18 years | Respiratory disorders, trauma or surgery in the head and neck region |
| Castro-Silva et al. 2015 | Retrospective; Uni; BRA | CBCT / i-CAT (Imaging Sciences International, Hatfield, PA, USA) / Dolphin Imaging 3-D, InVivoDental, and OnDemand 3-D / Upright (sitting) / Instructions: YES | N: 20  Males: 9  Females: 11  ANB: 0.6°- 3.3°  Age total sample: 27.4  Age range: 18-47 | N: 20  Males: 6  Females: 14  ANB: 0.3°-10°  Age total sample: 27.4  Age range 18-47 | N: 20  Males: 14  Females: 6  ANB: -0.5° - -9.1°  Age total sample: 27.4  Age range 18-47 | No categorization | Over 18 years, complete growth (hand wrist radiograph), normal body mass index (18.5-24.9) | Previous orthognathic surgery, OSA, cleft and craniofacial anomalies, transversal discrepancies |
| Chan et al. 2020  &  Vuong & Kang 2021 | Retrospective; Uni; USA | CBCT / i-CAT (Imaging Sciences International, Hatfield, Pa, USA) / Dolphin 3D / Upright (sitting) / Instructions: No info | 1°< ANB < 5°  N: 140 | ANB ≥ 5°  N: 140 | ANB ≤ 1°  N: 140 | No categorization | 9-15 years, white | Craniofacial syndromes, facial neoplasms, airway abnormalities, visibly enlarged tonsils noted in oral examination records, previous orthodontic treatment, noticeable head extension and flexion |
| Chen et al. 2021 | Retrospective; Uni; TWN | CBCT / New Tom VGi Evo (Imola, Italy) / Dolphin® 11.0 / Upright (standing) / Instructions: No info | 0°≤ANB≤4°  N: 30  Males: 11 Females: 19  Age: 25.3 (5.70)  ANB: 2.2° (1.21)° | ANB>4°  N: 40  Males: 12 Females: 28  Age: 25.8 (5.95) ANB: 7.1° (2.05)° | ANB<0°  N: 77  Males: 33 Females: 44  Age: 23.8 (5.54)  ANB: -4.4° (3.10)° | No categorization | No information | Craniofacial disorders or malformation, pharyngeal or laryngeal pathology, craniofacial injuries |
| Cho et al. 2021 | Retrospective; Uni; KOR | CBCT / Alphard-Vega 3030 machine (Asahi Roentgen Ind, Co Ltd, Kyoto, Japan) / Upright (sitting) / Instructions: No info / Mimics (version 20.0, Materialise, Leuven, Belgium) | 0°≤ANB<4°  N:32  ANB: 2.34° (0.97)° | ANB≥4°  N:40  ANB: 6.12° (1.48)° | ANB<0°  N:28  ANB: -2.63° (3.14)° | **Normodivergent**  25°≤MPA≤30°  N: 41  ANB: 2.20° (3.22)°  **Hypodivergent**  MPA<25°  N:24  ANB: 1.39° (3.60)°  **Hyperdivergent**  MPA>30°  N:35  ANB: 3.61° (4.91)° |  | Syndromic craniofacial deformity, dental implants or orthodontic devices, craniofacial surgical treatment history, pharyngeal soft tissue surgery |
| Claudino et al. 2013 | Retrospective; Uni; BRA | CBCT / i-CAT (Imaging Sciences International, Hatfield, Pa) / Dolphin Imaging / Upright (sitting) / Instructions: No info | 1° ≤ ANB ≤ 3°  N: 17  Males: 5  Females: 12  Age: 15.62 (2.17) | ANB > 3°  N: 20  Males: 10  Females: 10  Age: 16.83 (2.74) | ANB < 1°  N: 17  Males: 6  Females: 11  Age: 16.28 (1.74) | No categorization | 13-20 years, good health conditions,  cranio-cervical inclination 90°-110°, CBCT image including the whole fourth cervical vertebra, FH-MP angle 19°-30°. | Previous orthodontic treatment or other treatment, airway pathology, severe hyperdivergence |
| Costa et al. 2022 | Retrospective; Uni; BRA | CBCT / i-CAT unit (KaVo) / OnDemand3D software (version 1.0.10.5385) &  ITK-SNAP software (version 3.0) / Upright (sitting) / Instructions: YES | N: 35 | N: 35 | N: 37 | No categorization | Over 18 years, complete airway development | History of surgery or respiratory pathosis in the pharyngeal airway |
| Di Carlo et al. 2014 | Retrospective; Uni; DNK | CBCT / NewTom 3G  (Verona, Italy)  Mimics 15.0 (Materialise, Belgium) / Supine / Instructions: YES | -0.5° < ANB < 4.5°  N:30  Total sample  Age range: 13-43 | ANB > 4.5°  N: 30  Total sample  Age range: 13-43 | ANB < -0.5°  N: 30  Total sample  Age range: 13-43 | No categorization | No information | Previous orthodontic  treatment, orthognathic surgery, syndromes,  pathology upper airway,  adeno-tonsillectomy, and subjective respiratory problem |
| Diwakar et al. 2021 | Retrospective; Uni; IND | CBCT / i-CAT Classic (Hatfield, PA, USA)  In Vivo Dental 5.1 (Anatomage, San Jose, CA, USA) / Upright (sitting) / Instructions: no info | No categorization. Total sample N: 80. Age: 15.38±1.10  ANB males: 4.19° (2.08)°, ANB females: 4.01° (2.89)°  SN-MP males: 28.94° (5.29)°, SN-MP females: 32.11° (6.33)° | | | No categorization | Teeth in complete  Intercuspation. | Previous orthodontic /orthognathic surgery treatment; facial clefts or other craniofacial  anomalies, OSA |
| El & Palomo 2011 | Retrospective; Uni; USA | CBCT / CB Mercuray Scanner (Hitachi Medical Systems America, Twinsburg, Ohio) / inVivoDental (IVD) program (version 4.0 Anatomage, San Jose, CA) / Upright (sitting) / Instructions: YES | 1°≤ANB≤3°  N:46  Age: 15.6 (0.6)  Age range: 14.5-17.3 | ANB>3°  N:50  Age: 15.4 (0.6)  Age range:14.3-16.9 | ANB<1°  N: 44  Age: 15.4 (0.8)  Age range: 14.2-17.0 | No categorization | 14-18 years, white | Transverse deficiencies, severe hypodivergence (FMA<19), severe hyperdivergence (FMA>31), obese (BMI>30), congenital craniofacial deformities, pharyngeal pathology, nasal obstruction, snoring, OSA, adenoidectomy, scans with incomplete imaging of the airway |
| El & Palomo 2013 | Retrospective; Uni; USA | CBCT / CB Mercuray Scanner (Hitachi Medical Systems America, Twinsburg, Ohio) / inVivoDental (IVD) program (version 4.0 Anatomage, San Jose, CA) / Upright (sitting) / Instructions: YES | 81°≥SNA≥77°  80°≥SNB≥76°  3°≥ANB≥1°  N: 21  Age: 15.64 (0.50) | **Maxillary Protrusion** with normal mandible:  SNA>80°  80°≥SNB≥76°  ANB>3°  N: 21  Age: typo errors  **Mandibular Retrusion** with normal maxilla:  81°≥SNA≥77°  SNB<76°  ANB>3°  N: 20  Age: typo errors | **Maxillary Retrusion** with normal mandible:  SNA<77°  80°≥SNB≥76°  ANB<1°  N: 19  Age: typo errors  **Mandibular protrusion** with normal maxilla  81°≥SNA≥77°  SNB>80°  ANB<1°  N: 20  Age: typo errors |  | 14-18 years, Caucasians | Transverse deficiencies, severe hypodivergence (FMA<19), severe hyperdivergence (FMA>31), obese (BMI>30), congenital craniofacial deformities, pharyngeal pathology, nasal obstruction, snoring, OSA, adenoidectomy, scans with incomplete imaging of the airway |
| Elagib et al. 2022 | Retrospective; Uni; KOR | CBCT / CB MercuRay (Hitachi, Osaka, Japan) / InVivo software ver.5.2 (Anatomage, San Jose, CA, USA) / Upright (sitting) / Instructions: YES | 1°≤ANB<4°  N:32  Males: 14  Females: 18 | ANB≥4  N: 32  Males: 13  Females: 19 | ANB<1°  N: 31  Males: 21  Females: 10 |  | 12-19 years, good general health | Previous orthodontic treatment, upper respiratory infection, pharyngeal pathology, adenoid or tonsil removal, adenoid and / or tonsillar hypertrophy |
| Firwana et al. 2019 | Retrospective; Uni; CHN | CBCT / Newtom 5G system (Verona, Italy) / Dolphin imaging software (version11.8) / Supine / Instructions: no info | N:88  Males: 46  Females: 42  Age: 22.56 (4.0)  ANB: 0.7°- 4.7°  FH-MP: 24.66° (0.61°) | N:68  Males: 26 Females: 42  Age: 22.32 (3.6)  ANB>4.7°  FH-MP: 28.35° (0.72°) |  | No categorization | Adults 18–39 years | Previous orthognathic surgery, severe hypodivergence (FMA<23.5°) and severe hyperdivergence (FMA>30.5°), respiratory disorders, pharyngeal pathology, snoring, nasal obstruction, OSA, adenoidectomy, syndromes |
| Gong et al. 2018 | Prospective; Uni; CHN | CBCT / DCT PRO Dentofacial CBCT System (VATECH, Gyeonggi-do, South Korea) / Dolphin Imaging 11.8 Software / Upright (sitting) / Instructions: YES | 1°≤ANB≤4.5°  N: 16  Mean age: 25.13 (4.21) | ANB>5°  N:15  Mean age: 25.67 (6.55) | ANB<0°  N:16  Mean age: 24.75 (5.13) | No categorization | 18-35 years, BMI<30, normal maxillary sagittal position, | Previous orthodontic treatment or orthognathic surgery, cleft lip or palate treatment, crowding, upper dental arch stenosis, nasal cavity or sinus surgery, subjective feeling od long term nasal obstruction, acute upper respiratory tract infection in the past two weeks |
| Grauer et al. 2009 | Retrospective; Uni; USA | CBCT / i-CAT scanner (Imaging Sciences International, Hatfield) / Dolphin 3D beta, version 2.3 / Upright (sitting) / Instructions: no info | N:21  Females: 14 Males: 7  Age: 25.16 (7.63) | N:22  Females: 14 Males: 8  Age: 24.83 (7.61) | N:19  Females: 10 Males: 9  Age: 23.97 (7.57) | **Bony facial index ratio:**  Bizygomatic width/Na-Me  (No other info)  **Normodivergent**  N: 20  **Hypodivergent**  N:21 **Hyperdivergent**  N: 21 | Not reported | Previous orthognathic surgery, syndromes, detectable pathology along the upper airway |
| Gupta et al. 2016 | Retrospective; College; IND | CBCT / i‑CAT (Imaging Sciences International, Hatfield, England) / Dolphin 3D software, version 11.5 / Upright (sitting) / Instructions: no info | N: 16  Age range: 18-28 years | N: 15  Age range: 18-28 years | N: 3  Age range: 18-28 years | **Bony facial index ratio:**  Bizygomatic width/Na-Me  **Normodivergent**  N:11  **Hypodivergent**  N:5  **Hyperdivergent**  N:18 | Healthy adults (18-28 years) | Previous maxillary expansion, orthognathic surgery, appliances in the mouth, congenital anomalies (cleft lip and palate), respiratory pathology, nasal respiratory pathology |
| Habumugisha et al. 2022 | Retrospective (cross-sectional and observational); Uni; CHN | CBCT / i-CAT (Imaging Sciences International, Hatfiel, PA, USA) / Dolphin Imaging software version 11.7 / Upright (sitting) / Instructions: YES | 1°≤ANB≤5°  N: 17  Males: 9 Females: 8  Age: 11.4 (0.71) | ANB>5°  N: 18  Males: 9 Females: 9  Age: 10.56 (1.04) | No sample | No categorization | 10-12 years, skeletal Class I and Class II, normal divergence,  SN-MP: 27.3° - 35.5°  FH- MP: 25.32° - 33.16°  BMI: 18.5-24 | Previous orthodontic treatment or orthognathic surgery, syndromes, craniofacial anomalies (cleft lip and palate), growth disturbances, enlarged tongue or ankyloglossia |
| Hong et al. 2010 | Prospective; Uni; KOR | CBCT / Master Dental 3D imaging system (Vatech Inc., Seoul, Korea) / InVivoDental (Anatomage Inc., San Jose, CA, USA) / Upright (standing) / Instructions: YES | 0≤ANB≤4°  N:18  Males: 9 Females: 9  Age males: 11.16 (0.69)  Age females: 11.29 (0.94)  ANB: 3.00° (1.25)°  FH-MP: 29.89° (4.22)° | ANB>4°  N:19  Males: 8 Females: 11  Age males: 10.59 (1.73)  Age females: 11.05 (1.61)  ANB: 5.85° (1.44)°  FH-MP: 31.95° (2.39)° | No sample | No categorization | 8-12 years | Adenoidectomy, tonsillectomy |
| Hong et al. 2011 | Prospective; Uni; KOR | CBCT / Master 3D dental imaging system (Vatech, Seoul, Korea) / InVivoDental software (Anatomage, San Jose, CA, USA) / Upright (standing) / Instructions: YES | N: 29  Male: 14  Female: 15  Age males: 27.9 (4.9)  Age females: 25.2 (5.0) | No sample | N: 31  Male: 16  Female: 15  Age males: 24.1 (3.2)  Age females: 25.3 (5.0) | No categorization | 18 - 30 years, permanent dentition (including 7s) | Adenoidectomy, tonsillectomy, craniofacial anomaly, pharyngeal pathology |
| Indriksone & Jakobsone 2015 | Retrospective; Uni; LVA | CBCT / iCAT Scanner (Imaging Sciences International,  Hatfield, Pa) / Dolphin 11.7 software / Upright (sitting) / Instructions: No info | ANB: 1°-5°  Total sample N: 276  Total sample age: 21 (3.2) | ANB > 5°  Total sample N: 276  Total sample age: 21 (3.2) | ANB < 1°  Total sample N: 276  Total sample age: 21 (3.2) | Total sample  SN-MP: 34.8 (7.0)  FH-MP: 25.4 (6.7) | 17-27 years, central occlusion, craniocervical inclination 90°-110° | Medically compromised patients (OSA, syndromes and arthritis), previous orthognathic surgery, facial clefts,  severe craniofacial asymmetries |
| Iwasaki et al. 2009 | Prospective; Uni; JPN | CBCT / CB MercuRay, Hitachi Medical, Tokyo, Japan / Software INTAGE volume Editor, KGT, Tokyo, Japan / Upright (sitting) / Instructions: No info | N: 25  Males: 13 Females: 12  Age: 8.8 (1.0)  ANB: 3.56 (1.97) | No sample | N: 20  Males: 12  Females: 8  Age: 8.4 (1.0)  ANB: 0.40 (1.45) | No categorization | Class I: molar and canines, overjet 2-4mm.  Class II: molars and canines, negative overjet  Craniocervical inclination 90° - 110° |  |
| Iwasaki et al. 2017 | Prospective; Pract; JPN | CBCT / Alphard 3030; Asahi Roentgen Industry, Kyoto, Japan) / Software INTAGE Volume Editor; Cybernet Systems, Tokyo, Japan) / Upright (sitting) / Instructions: YES |  | ANB>5°  N: 33  Males: 12  Females: 21  Age: 9.4 (1.1) | ANB<1°  N: 31  Males: 9  Females: 22  Age: 9.2 (1.5) | No categorization | 7-12 years, craniocervical inclination 95°- 105°  FH-MP: 25° - 33° | Previous orthodontic treatment, craniofacial or growth abnormalities, tonsillectomy, adenoidectomy,  systemic disease, temporomandibular joint disorders (TMD) |
| Iwasaki et al. 2019 | Retrospective; Uni; JPN | CBCT / Alphard 3030, Asahi Roentgen, Kyoto, Japan) / Simpleware ScanIP version 7.0.  Synopsis, <mountain View, CA, USA / Upright (sitting) / Instructions: YES | 76°<SNB<80°  N: 20  Males: 9 Females: 11  Age: 9.3 (0.8) | SNB<76°  N:20  Males: 9 Females: 11  Age: 9.2 (1.1) | SNB>80°  N:20  Males: 9 Females: 11  Age: 9.1 (1.4) | No categorization | 7-11years; craniocervical inclination of 95°-105° | Previous orthodontic treatment, craniofacial or growth abnormalities, previous treatment for tonsillectomy or adenoidectomy, systemic disease and temporomandibular joint disorders (TMDs) |
| Jadhav et al. 2019 | Retrospective; College; IND | CBCT / i‑Cat 17/19 C, Imaging Sciences International, USA) / Invivo 5.1 software, viewer version 1.9 / Upright (sitting) / Instructions: YES | 1°<ANB<4°  N: 15 | ANB≥4°  N: 15 | ANB<1°  N: 15 | No categorization | 16-25 years, permanent dentition | Very high‑angle  FH-MP: 35°–40° or  very low‑angle  FH-MP <10‑15° and noticeable pharyngeal pathology |
| Jayaratne et al. 2016 | Retrospective; Uni; USA | CBCT / i-CAT System, Imaging Sciences International, Hatfield, PA, USA) / 3dMDVultus software (3dMD LLC, Atlanta, GA, USA) / Upright (sitting) / Instructions: YES |  | N: 27  Age: 24.7 (7.21) | N: 35  Age: 20.7 (3.57) |  | No further information other than Class II and Class III deformities | Obvious facial asymmetry, OSA, congenital deformities, cleft lip/palate, syndromes, trauma or previous maxillofacial surgery |
| Kikuchi et al. 2008 | Retrospective; College; JPN | Spiral scan-type CT / SOMATOM Plus 4 Volume Zoom, SIEMENS Co. / Software Real INTAGE, K.G.T. Co. / Supine / Instructions: YES | Total sample N: 25  Adult females  Age mean: 19y10m  Age range: 15y8m-28y7m  SNA average: 80.9 SNB average: 78 ANB average: 2.9 | Total sample N: 25  Adult females  Age mean: 19y10m  Age range: 15y8m-28y7m  SNA average: 81.3 SNB average: 73.6 ANB average: 7.7 | Total sample N: 25  Adult females  Age mean: 19y10m  Age range: 15y8m-28y7m  SNA average: 81.8 SNB average: 83.9 ANB average: -2.1 | **Normodivergent**  SN-MP: 28.8°±4°  N: 10, Age: 18y4m  **Hypodivergent**  SN-MP< 24.8°  N: 5, Age: 22y6m  **Hyperdivergent**  SN-MP≥ 32.9°  N: 10, Age : 18y1m | Facial asymmetry absent or slight, deviation between the maxillo-mandibular anterior tooth medians within 4mm. | Previous orthodontic treatment, prostheses larger than full cast crowns, systemic diseases and congenital deformity influencing growth, inflammation in the pharyngeal airway, tonsillar hypertrophy (objectively or subjectively noted). |
| Kim et al. 2010 | Retrospective; Uni; KOR | CBCT / Master 3D dental-imaging system (Vatech,  Seoul, Korea) / InVivoDental software (Anatomage, San Jose, Calif) / V-ceph software (Osstem Implant, Seoul, Korea) / Upright (standing) / Instructions: YES | 2°≤ANB≤5°  N: 14  Males: 7 Age: 11.46 ± 0.69  Age range: 10.50-12.58  Females: 7  Age: 11.71 ± 0.86  Age range: 10.67-12.92 | ANB>5°  N: 13  Males: 5  Age: 10.23 ± 0.73  Age range: 9.42-12.33  Females: 8  Age: 11.09 ± 1.03  Age range: 9.08-12.17 | No sample | No categorization | Healthy children with a mean age of 11.19 ± 1.28 years | Symptoms of upper respiratory infection, pharyngeal pathology such as adenoid hypertrophy and tonsillitis or a history of adenoidectomy or tonsillectomy |
| Kochhar et al. 2021 | Retrospective; College; IND | CBCT / i-Cat CBCT (Imaging Sciences Hatfield, PA, USA / InVivoDental software (Anatomage, San Jose, CA, USA) / Upright (sitting) / Instructions: YES | ANB<4°  N: 56  Males: 26 Females: 30  Age: 15.19 (1.28) | ANB>4°  N: 64  Males: 28 Females: 36  Age: 15.19 (1.28) | No sample | No categorization | Not reported any. | Upper respiratory infection, pharyngeal pathology, adenoid or tonsil removal. |
| Lee et al. 2019 | Retrospective; Uni; KOR | CBCT / Alphard 3030, Asahi, Kyoto, Japan / software Mimics version 17, Materialise / Upright (sitting) / Instructions: No info | N: 5 | N: 5 | N: 5 |  | Male adults | Missing or asymmetric teeth, psychological disorder |
| Li et al. 2015 | Retrospective; Uni; CHN | CBCT / KaVo 3D eXam / InVivo5 Software / Upright (sitting) / Instructions: YES | ANB: 3,28 (1.25)  Age: 24.40 (3.75) | No sample | **Maxillary Retrusive**  ANB: -0.90 (0.49)  Age: 26.00 (1.73)  **Mandibular Protrusive**  ANB: -1.22 (1.19)  Age: 25.50 (4.20) | No categorization | 19-33 years old  BMI ≤28 | Previous orthodontic treatment, cleft lip & palate, congenital craniofacial deformities,  upper respiratory tract disease, nasal disorders, adenoidectomy, tonsillectomy, long term mouth breathing, snoring, DSAHS, pharyngeal pathology |
| Mei et al. 2019 | Retrospective; Hospital; CHN | CBCT / No information on CBCT equipment / Mimics 20.0 software / Upright / Instructions: YES | ANB ≥ 0.7°  27.3 °≤ SN-MP ≤ 37.7 °  N: 21  Males: 10 Females: 11 | No sample | ANB < 0.7 °  SNA > 79°  SNB > 84°  SN-MP > 37.7 °  N: 21  Males: 10 Females: 11 |  | 18-35 years, harmonious and symmetrical face, clear CBCT,  BMI: 18.5-23.9 | Craniofacial deformity, cleft lip / palate, previous orthodontic or orthognathic surgical treatment, history of tonsil / adenoid hypertrophy / surgery, obesity (BMI> 24),  skeletal class III with pure maxillary hypoplasia or pure mandibular hyperplasia |
| Mello et al. 2019 | Retrospective; Uni; BRA | CBCT / Kodak® 9500 Cone Beam 2D System (Carestream Health, Rochester, NY, EUA) /  Dolphin Imaging software, version 11.8 Premium (Dolphin  Imaging, Chatsworth, CA, USA) / Upright: standing / Instructions: YES | 1°≤ANB≤3°  N:41  Male:17  Female: 24  Age: 14.54 (1.94) | ANB > 3°  N:45  Male: 26 Female: 19  Age: 14.62 (1.91) | ANB < 1°  N:40  Male:13  Female: 27  Age: 15.73 (2.09) | No categorization | 13-20 years before CBCT acquisition, craniocervical angle between 90°-110° | Previous orthodontic treatment, therapy that could interfere with maxillomandibular growth, systemic and/or oral diseases; airway pathology; craniofacial congenital or syndromic anomalies, severe divergence  (19° ≤ FH-MP ≤30°) |
| Miranda-Viana et al. 2021 | Retrospective; Uni; BRA (only nasal breathers) | CBCT / i-CAT Next Generation; Imaging Sciences International, Hatfield, PA, USA / ITK-SNAP  v.3.0 (Cognitica, Philadelphia, PA) / Upright (sitting) / Instructions: No info | 0°≤ANB≤4°  upper airways volume analysis:  N: 126  Maxillary sinuses volume analysis:  N: 91 | ANB>4°  upper airways volume analysis:  N: 108  Maxillary sinuses volume analysis:  N: 73 | ANB<0°  upper airways volume analysis:  N: 64  Maxillary sinuses volume analysis:  N: 48 | **Vertical index**  Arithmetic average: facial depth FH-NPog, facial axis NBa-PtGn, lower facial height Xi-ANS/XiPm, mandibular plane angle FH-MP, mandibular arch DcXi-XiPm  -LA > 0.5  HA< -0.5  -0.5 < AV < 0.5  Upper airways volume analysis:  LA: N: 122  AV: N:111 HA: N:65  Maxillary sinuses volume analysis:  LA: N:90 AV: N:72 HA: N:50 | Over 18 years, both sexes, all teeth present (3^rd^ molars not required) | Trauma or orthognathic surgery, presence of bone fracture, syndromes, bone exostoses, pathological lesions, cleft lip/palate |
| Moshajari et al. 2020 | Retrospective; Uni; IRN | CT / Somatom Scope CT scan system, VC30A, Siemens, Germany / CT software SOMARIS/5 VC30, Siemens, Germany / Supine / Instructions: YES | N: 25  ANB: 3.42 (0.61) | N: 45  ANB: 6.76 (1.62) | N: 23  ANB: 0.02 (1.52) | **Normodivergent**  N: 8  **Hypodivergent**  N: 22  **Hyperdivergent**  N: 63 | Over 15 years | Previous orthodontic treatment, jaw orthopaedics, orthognathic surgery, tonsillectomy, adenoidectomy, rhinoplasty, cleft lip palate, craniofacial syndromes, airway pathology, OSA, trauma |
| Nath et al. 2021 | Retrospective; College; IND | CBCT / Planmeca Promax 3D Mid unit (Planmeca Oy,Helsinki, Finland) / Romexis software version 4.6.2 / Upright (standing) / Instructions: no info | 0°≤ANB≤4°  SNB: 80°  Wits appraisal: 0-2mm  N: 55  Males: 36 Females: 19  Age: 21.04 (3.04) | ANB >4°  SNB<80°  Wits appraisal ˃ 2mm  N: 55  Males: 17 Females: 38  Age: 21.16 (2.28) | ANB<0°  SNB>80°  Wits appraisal < 1mm  N: 70  Males: 29 Females: 41  Age: 21.11 (2.67) |  |  | Previous orthodontic treatment, facial asymmetry, deformities (cleft lip and palate), trauma, upper respiratory infections |
| Nejaim et al. 2018 | Retrospective; Uni; BRA | CBCT / i-CAT Next Generation Imaging Sciences International, Hatfield, PA, USA / ITK-SNAP  v.3.0 (Cognitica, Philadelphia, PA) / Upright (sitting) / Instruction: YES | N: 60  Males: 23  Females: 37 | N: 60  Males: 29  Females: 31 | N: 41  Males: 28  Females: 13 | **Normodivergent**  N : 53  Males: 24  Females: 29 **Hypodivergent**  N : 70  Males: 35  Females: 35 **Hyperdivergent**  N : 38  Males: 21  Females: 17 | 21 - 58 years | Younger than 21 years (incomplete development of their craniofacial structures), orthognathic surgery, pathologies in the region of the head and neck or syndromes |
| Oh et al. 2011 | Prospective; Uni; KOR | CBCT  InVivoDental software (AnatomageInc, San Jose, Calif) / Master 3D dental imaging system(Vatech Inc, Seoul, Korea) / Upright / Instructions: YES | 1°≤ ANB≤ 4°  N: 19  Males: 8  Age: 11.8 (0.62)  Females: 11  Age: 11.5 (1.23) | ANB≥4°  N: 27  Males: 14  Age: 11.39 (0.94)  Females: 13  Age: 11.19 (1.14) | ANB<1°  N: 14  Males: 3  Age: 10.80 (2.29)  Females: 11  Age: 10.83 (1.73) | No categorization | 10-13 years, healthy growing children | Congenital anomalies (eg. cleft lip and palate), adenoidectomy or tonsillectomy or symptoms of respiratory pathology |
| Paredes et al. 2021 | Retrospective  Cross-sectional; Uni; PER | CBCT / Planmeca Pro Max 3D Mid (Planmeca Oy,Helsinki, Finland) / PLANMECA Romexis Viewer / Upright (standing) / Instructions: No info | 1°< ANB ≤3°  N: 20  Males: 8  Females: 12 | ANB>3°  N: 20  Males: 8  Females: 12 | ANB<1°  N: 20  Males: 11  Females: 9 |  | 14-20 years, peruvian origin, permanent dentition | Previous orthodontic treatment or orthognathic surgery, congenital and acquired deformities, airway pathologies, |
| Paul D et al 2015 | Retrospective; Uni; IND | Peripheral nervous system CT scans / Somatom  DRH scanner (Siemens sensation 64, Germany) / SYNGO software to create DICOM files / Dolphin software to measure airways / Supine / Instructions: YES | ANB ≤ 4.5°  N: 15 | ANB > 4.5°  N: 15 |  |  |  | Less than 18 years, precious orthodontic treatment of orthognathic surgery, adenoidectomy or tonsillectomy, severely enlarged adenoids, cleft lip/palate, or syndromes, |
| Paul P et al. 2022 | Retrospective; College; IND | CBCT / No further info / ITK SNAP Software (version 3.8.0, Cognitica) / No further info |  |  |  | **Normodivergent**  SN-MP : 27°-37°  N: 15  Males: 6  Females:9  Age: 25.53 (1.89)  **Hypodivergent**  SN-MP <27°  N:16  Males:4  Females:12  Age: 22.56 (3.39)  **Hyperdivergent**  SN-MP >37°  N:15  Males:5  Females:10  Age: 23.13 (2.67) | 18-30 years | Previous orthodontic treatment and orthognathic surgery, cleft lip or palate tonsillectomy or adenoidectomy , upper airway pathology, trauma |
| Perrotti et al. 2021 | Retrospective; Uni; ITA | CBCT / No information on equipment / Mimics 17.0 (Materialize, Leuven, Belgium) / Position not defined / Instructions: YES | 1≤ANB≤3  N: 33  Male: 17  Female: 16 | ANB>3  N: 33  Male: 14  Female: 19 | ANB<1  N: 33  Male: 17  Female: 16 | No categorization | 18-65 years, healthy | Previous orthodontic treatment, pathologies (congenital or gained) upper airways, surgery upper airways, OSAS |
| Ravello et al. 2020 & 2021 | Prospective; Uni; CHL | CBCT / NewTom 3D Tomograph, Model VGi EVO (Verona, Italy) / NNT New Tom  software (Imola, Italy) / Upright (standing) / Instructions: YES | No sample | ANB>4°  Total sample N : 115  No information per Class | ANB<0°  Total sample N : 115  No information per Class | Total sample N : 115  **Normodivergent**  N:7  **Hypodivergent**  N : 34  **Hyperdivergent**  N : 74 | Over 18 years | Previous surgery, trauma, syndromes, asymmetries (more than 5mm chin deviation) |
| Saati et al. 2021 | Retrospective; Uni; IRN | CBCT / NewTom 3G / ITK SNAP Software (version 3.6.0) / Supine / Instructions: YES | 1°≤ANB≤5°  N: 95 | ANB>5°  N: 57 | ANB<1°  N: 63 | AV: SN-MP 32°  LA : SN-MP < 32°  HA : SN-MP > 32° | Over 18 years | Previous orthodontic treatments or maxillofacial surgeries, syndromes, cleft lip or palate, trauma, pharyngeal pathology |
| Sharma et al. 2016 | Retrospective; Uni; IND | MRI / Brivo TM MR355 1.5T ; General Electric / Software Osirix v5.6 Biomedical Visualizers, Geneva, Switzerland / Supine / Instructions: No info | N: 15  Males: 8  Females: 7 | N:15  Males: 8  Females: 7 | No sample | Class I: normodivergent  Class II: hyperdivergent | 12-18 years | Previous orthodontic treatment |
| Shokri et al. 2018, 2020, 2021 | Retrospective Cross-Sectional; Uni; IRN | CBCT / NewTom 3G CBCT system  ITK-SNAP version 3.6.0 (Penn Image Computing and Science Laboratory, Philadelphia, PA, USA) / Supine / Instructions: YES | 0°<ANB<4°  N: 52 | ANB≥4  N: 66 | ANB<1°  N: 62 | N : 60  **Normodivergent**  PFH/AFH : 62-65%  N:20  **Hypodivergent**  PFH/AFH >65%  N:20  **Hyperdivergent**  PFH/AFH <62%  N:20 | Over 18 | Previous orthognathic surgery, pharyngeal pathology, respiratory disorders, |
| Sparks RJ 2012  (Thesis) | Retrospective; Uni; USA  (Data from Advanced Dental imaging Center) | CBCT / Hitachi CB Mercuray / InVivo Dental software (version 5.1) / Upright (sitting) / Instructions: No info | 0°<ANB<5°  N:93  (31 of each divergence) | ANB>5°  N:93  (31 of each divergence) | ANB<0°  N:93  (31 of each divergence) | **Normodivergent**  FH-MP: 22°-30°  N:93 (31 of each class)  **Hypodivergent**  FH:MP <22°  N:93 (31 of each class)  **Hyperdivergent**  FH-MP >30°  N:93 (31 of each class) | Adults, craniocervical  inclinations 90°-110° | No information |
| Tseng et al. 2021 | Retrospective; Uni; TWN | CBCT / NewTom BGi evo, Imola, Italy) / Soteria DcmRecons (version Alpha v0.7; soteria Biotech Ltd., New Taipei City, Taiwan) / Upright / Instructions: YES | 0°≤ANB≤4°  N:30  Male: 8 Female: 22  Age: 25.7 (6.52) | ANB>4°  N:30  Male: 10 Female: 20  Age: 24.8 (3.69) | ANB<0°  N:30  Male: 15 Female: 25  Age: 24.4 (4.12) | No categorization | No information | Craniofacial symptoms, tumor in the pharyngeal airway space, orthognathic surgery, facial bone injury in the craniofacial area. |
| Unal & Soydinc 2021 | Retrospective; Uni; TUR | CBCT / NewTome 5G / Mimics 17.0 (Materialize Europe, Leuven, Belgium) / Supine / Instruction: YES | N: 60  Normodivergent N:20  Hypodivergent N: 20  Hyperdivergent N 20 | N: 60  Normodivergent N:20  Hypodivergent N: 20  Hyperdivergent N 20 | N: 60  Normodivergent N: 20  Hypodivergent N: 20  Hyperdivergent N 20 | N: 180  Normodivergent N: 60  Hypodivergent N: 60  Hyperdivergent N : 60 | Over 16 years | Previous orthodontic or orthognathic treatment, disease or syndrome of the skeletal system, more than 4 teeth missing (loss vertical dimension), hard or soft tissue operation at head and neck region. |
| Unuvar et al. 2021a | Retrospective; Uni; TUR | CBCT / i-CAT (Imaging Sciences International, Hatfield, PA, USA) / Upright (sitting) / Instruction: No info | 0°<ANB<4°  N: 82  Males: 40  Females: 42 | ANB>4°  N: 83  Males: 43  Females: 40 | ANB<0°  N: 70  Males: 38  Females:32 | **Normodivergent**  28<SN-MP<36  N: 79  Males: 41  Females:38  **Hypodivergent**  SN-MP<28,  N: 78  Males: 39  Females:38  **Hyperdivergent**  SN_MP > 36  N: 78  Males:41  Females:37 | Over 16 years | Previous orthodontic treatment or orthognathic surgery, detectable pathology upper airway, missing teeth (except 3^rd^ molars), craniofacial syndrome and cleft lip and palate, adenoidectomy or tonsillectomy, nasal obstruction |
| Unuvar et al. 2021b | Retrospective; Uni; TUR | CBCT / ProMax 3D scanner (Planmeca, Helsinki, Finland) / Dolphin 3D (version 11 Dolphin Imaging & Management Solutions, LA, CA) / Upright (standing) / Instruction: No info | 0°<ANB<4°  N:51  Age: 30.57 (11.88) | ANB>4°  N:42  **Retrognathic mandible**  Normal maxilla  84>SNA>80  SNB<78  N:21  Age: 31.57 (11.88)  **Prognathic maxilla** Normal mandible  SNA>84  82>SNB>78  N: 21  Age: 31.09 (10.87) | No sample | No categorization | 16-43 years | Previous orthodontic treatment or orthognathic surgery, detectable pathology along the upper airway, missing teeth (except 3^rd^ molars), craniofacial syndrome, adenoidectomy or tonsillectomy, severe hypodivergent growth pattern, (FMA>31) nasal obstruction |
| Vidal-Manyari et al. 2020 | Retrospective Cross-Sectional; Uni; BRA | CBCT / Vatech E-WOO Picasso Master 3D scanner / Planmeca Romexis software / Unright (sitting) / Instructions: YES | 2°≤ANB≤4°  N: 41  Normodivergent: 30  Hyperdivergent: 11 | ANB≥4°  N: 49  Normodivergent: 30 Hyperdivergent: 19 | ANB≤0°  N: 47  Normodivergent: 30 Hyperdivergent: 17 | **Normodivergent**  N: 90  Age: 26.87 (7.28)  ANB: 2.09 (3.83)  **Hyperdivergent**  N: 47  Age: 27.89 (11.50)  ANB: 2.31 (6.05) | 15-60 years  Latin-Americans | Active or previous orthodontic or orthopaedic treatment, orthognathic surgery, completely or partially edentulous in the anterior region, had bone alterations. |
| Vidya et al. 2020 | Prospective; College; IND | CBCT / CareStream 9000 / TurtleSeg software / Upright (standing) / Instructions: YES | N:15 | N:15 | N:15 | No categorization | 20-35 years | Transverse deficiencies,  severe hypodivergent growth pattern, severe hyperdivergent growth pattern, obese (BMI≥30), congenital craniofacial deformities, pharyngeal pathology,  nasal obstruction, adenoidectomy |
| Wan et al. 2019 | Retrospective; Uni; CHN | CBCT / CBCT scanner (KaVO eXam Vision, America) / Mimics 17.0 / Upright(sitting) / Instructions: YES | 0.7°≤ANB≤4.7°  76°<SNB<84°  N:30  Age: 15.13 (1.20) | ANB≥4.7°  SNB<76°  N:30  Age: 14.93 (1.05) | No sample |  | 13-16 years, healthy, Han Chinese ethnicity, permanent teeth, clinically symmetric, normal length of mouth opening, normal type of mouth opening,  BMI: 18.1-24,  SN-MP: 27.3° - 37.7° | Previous orthodontic treatment, nasal diseases,  cheilopalatognathus, maxilla facial malformation, |
| Wanzeler et al. 2019 | Retrospective; Uni; BRA | CBCT / No information on equipment, position, instructions etc. / Cephalometrics: InVivoDental 5.0 (Anatomage Inc. San Jose, CA, USA) / Airways: ITK-SNAP 2.1.4 | No categorization | No categorization | No categorization | Normodivergent N: 30  Hypodivergent N: 30  Hyperdivergent N: 30 | Adults | Surgery prior to the examination, diagnosed with airway disorders, syndromes. |
| Yanagita et al. 2017 | Prospective; Uni; JPN | CBCT / CB MercuRay (Hitachi Medical,  Japan) / Software: VG Studio MAX 1.2, Nihon Visual Science,  Tokyo) / Upright (sitting) / Instructions: No info | Adolescent group N: 34  Age: 11.15 (0.86)  ANB: 4.43 (2.36)  FH-MP: 31.51 (3.66)  Late adolescent group N: 28  Age: 14.23 (1.13)  ANB: 3.74 (1.97)  FH-MP: 28.00 (4.57) | | | | 10-16 years,  Japanese, females,  Craniocervical angulation 80°-108° | Previous orthodontic treatment; congenital anomalies, endocrine problems, facial or spinal abnormalities,  dentomaxillofacial and neck region trauma, allergic rhinitis, clinical signs or symptoms and complaints of nasal  obstruction, low tongue posture without  nasal obstruction, pharyngeal pathology  (adenoid and/  or tonsil hypertrophy), adenoidectomy,  tonsillectomy. |
| Zheng et al. 2014 | Retrospective; Uni; JPN | CBCT / CB MercuRay, Hitachi Medical, Tokyo, Japan / Software: CBWorks 2.1, CyberMed Corp, Seoul, Korea / Upright (sitting) / Instructions: YES | 1°≤ANB≤3°  N: 20  Males: 10  Age: 15.20 (1.03)  Females: 10  Age: 15.30 (1.34) | ANB>3°  N: 20  Males: 8  Age: 15.25 (1.58)  Females: 12  Age: 15.25 (1.58) | ANB<1°  N: 20  Males: 11  Age: 16.73 (0.91)  Females: 19  Age: 16.11 (1.36) | No categorization | 14-18 years  complete dentition, symmetric mandible | Previous orthodontic treatment or orofacial surgery, BMI >28, congenital craniofacial  deformities, nasal obstruction,  snoring, OSA, detectable airway  pathology |
| Zou et al. 2020 | Retrospective; Uni; JPN | CBCT / NewTome 5G, Verona, Italy / Mimics 17.0 (materialize, Louven, Belgium) / Supine / Instructions: YES | N: 42  Males: 18  Females: 24  Age: 11.21 (1.12) | N: 38  Males: 16  Females: 22  Age: 11.26 (1.15) | N: 32  Males: 18  Females: 24  Age: 11.21± (1.12) | No categorization | 7-14 years  Craniocervical angulation 95°-105° | Previous orthodontic treatment or orthognathic surgery, oral habits (thumb sucking), mouth breathing, anterior tongue posture, chronic rhinitis, hypertrophic tonsils, hypertrophic adenoids, other respiratory diseases, tonsillectomy, adenoidectomy |

N: number of participants, SD: standard deviation, SE: standard error, AV: Average: HA: high angle, LA: low angle, AP: anteroposterior; OSA: obstructive sleep apnea; SN: anterior cranial base line: FH: Frankfort Horizontal; MP: mandibular plane; SPA: sum of posterior angles; MaxP: maxillary protrusive; MandR: mandibular retrusive; MaxR: maxillary retrusive; MandP: mandibular protrusive; AV: normodivergent; LA: hypodivergent; HA: hyperdivergent; OSA: Obstructive Sleep Apnea

**Supplementary Table 7. Outcomes and key results of the included studies.**

| **Study** | **Outcomes**  **Class I**  **Normodivergent (AV)**  **Hypodivergent (LA)**  **Hyperdivergent (HA)**  **Mean (SD) mm^3^** | **Outcomes**  **Class II**  **Normodivergent (AV)**  **Hypodivergent (LA)**  **Hyperdivergent (HA)**  **Mean (SD) mm^3^** | **Outcomes**  **Class III**  **Normodivergent (AV)**  **Hypodivergent (LA)**  **Hyperdivergent (HA)**  **Mean (SD) mm^3^** | **Outcomes**  **Vertical skeletal patterns**  **Normodivergent (AV)**  **Hypodivergent (LA)**  **Hyperdivergent (HA)**  **Mean (SD) mm^3^** | **Differences between groups**  **Mean (SD) mm^3^** | ***P*** |
| --- | --- | --- | --- | --- | --- | --- |
| Abdelkarim et al. 2012 | OP : 10247 (4138) | OP: 7406 (3541) | OP:12909 (5226) | No categorization | II < I < III | 0.00* |
| Aby et al. 2020 | TP: 20733.2 (6717.953)  TP male:17252.0 (5137.170)  TP female: 22225.2 (6862.420) | TP: 19032.2 (4394.530)  TP male: 17317.8 (4245.412)  TP: female: 19889.4 (4314.881) | No sample | No categorization | TP: NS  TP male: NS  TP: female: NS | 0.251 |
| Alhammadi et al 2021 | **Normodivergent (AV)**  NP: 5154.6667 (1907.28855)  PP: 6650.4667 (2639.86773)  GP: 6969.5667 (3855.92036)  NP+PP+GP: 18719.9600 (5574.93816)  **Hyperdivergent (HA)**  NP: 4352.9400 (2139.47529)  PP: 6891.4967 (5293.80328)  GP: 6210.9833 (4003.55774)  NP+PP+GP: 17723.4667 (10478.34395) | **Normodivergent (AV)**  NP: 5872.8067 (2092.36213)  PP: 5930.2933 (2552.94941)  GP : 4991.8933 (2021.08030)  NP+PP+GP: 16841.9733 (4399.28127)  **Hyperdivergent (HA)**  NP: 5184.5867 (1491.38898  PP: 7534.0667 (2051.31766)  GP: 7233.6933 (2741.82782)  NP+PP+GP: 19826.0200 (5093.35617) | No sample | No categorization | **I vs II(AV)**  NP: NS  PP: NS  GP: II < I  NP+PP+GP: NS  **I vs II(HA)**  NP: NS  PP : NS  GP : NS  NP+PP+GP: NS  **I(AV) vs I(HA)**  NP: NS  PP : NS  GP : NS  NP+PP+GP: NS  **II(AV) vs II(HA)**  NP : NS  PP : HA > AV  GP : HA > AV  NP+PP+GP: HA > AV | **I vs II(AV)**  0.170  0.287  0.017*  0.153  **I vs II(HA)**  0.086  0.538  0.253  0.327  **I(AV) vs I(HA)**  0.131  0.824  0.458  0.647  **II(AV) vs II(HA)**  0.148  0.010*  0.001*  0.018* |
| Alves Jr. M et al. 2012 | OP: 7588.82 (1892.75) | OP: 5561.92 (1778.13) | No sample | No categorization | OP: II < I | 0.002* |
| Alves PVM et al. 2008 | No sample | NC: 25328.2 (5222.0)  PP: 5453.7 (1744.0)  GP + HP: 6140.0 (2498.8)  OP+HP: 11593.7 (3711.8)  TA: 36922.0 (7890.6) | NC: 23343.5 (6591.4)  PP: 5809.5 (2302.0)  GP + HP: 6423.7 (2709.7)  OP + HP: 12233.2 (4569.5)  TA: 35576.7 (7992.4) | No categorization | NC: NS  PP: NS  GP + HP: NS  OP + HP: NS  TA: NS | 0.268  0.513  0.679  0.562  0.554 |
| Anandarajah et al. 2017 | OP + HP: Correlations reported for the whole sample.  Unadjusted: No significant associations  Adjusted for age, skeletal maturity and sex: significant associations with:   - Total anterior face height (N-Me) (r:0.51) - Upper anterior face height (N-ANS) (r:0.52) - Maxillary width (Mx΄- Mx) (r:0.53)   Mandibular width (Go΄- Go) (r:0.60 | | | | | OP + HP  0.020*  0.013*  0.003*  0.000* |
| Bozzini et al. 2018 | **Males**  OP + HP: 19419.7 (7916.7)  **Females**  OP + HP: 16630.9 (5730.4) | No sample | **Males**  OP + HP: 30348.3 (10272.4)  **Females**  OP + HP: 18369. (6235.8) | No categorization | OP + HP males: I < III  OP + HP females : NS | <0.05*  ˃0.05 |
| Brasil et al. 2016 | No sample | NP: 10868.8 (3734)  OP + HP: 10619.6 (7377.8)  TP: 21649.8 (10044.4) | NP: 8948.6 (3307.1)  OP + HP: 13132.6 (5491.4)  TP: 24031 (8682.6) | **Normodivergent**  NP: 9756.6 (3953.6)  OP + HP: 10872.9 (4854.6)  TP: 21291.1 (7779.3)  **Hypodivergent**  NP: 9207.9 (4001.6)  OP + HP: 12987.8 (9695.3)  TP: 23283.7 (13335.1)  **Hyperdivergent**  NP: 11061.4 (4300.5)  OP + HP: 11513.6 (4825.3)  TP: 22237.1 (7633.9) | **Comparisons per Class**  NP: NS  OP + HP: NS  TP: NS  **Comparisons per divergence**  NP: NS  OP + HP: NS  TP: NS | 0.467  0.094  0.221  0.320  0.632  0.966 |
| Brito et al. 2019 | **Normodivergent**  NP: 4267.61 (1546.19)  OP + HP: 11937.16 (3726.56)  Lower HP: 4001.29 (1489.16)  **Hypodivergent**  NP: 4296,50 (1787.72)  OP+ HP: 12988.68 (4755.63)  Lower H : 3719.57 (1278.36)  **Hyperdivergent**  NP: 4958.21 (1955.48)  OP + HP: 13690.29 (4783.87)  Lower HP: 4179.47 (1771.02) | **Normodivergent**  NP MaxP: 4771.36 (1456.06)  NP MandR: 4677.50 (1550.72)  OP+HP MaxP: 15155.09 (5637.77)  OP+HP MandR: 12864.60 (4247.02)  Lower HP MaxP: 3697.91 (1962.35)  Lower HP MandR: 4099.37 (1450.22)  **Hypodivergent**  NP MaxP:5235.03 (2187.83)  NP MandR: 4600.41 (1394.40)  OP + HP MaxP: 12808.56 (6092.48)  OP + HP MandR: 12098 (3487.24)  Lower HP MaxP: 4245.27 (1660.52)  Lower HP MandR: 3964.56 (1157.77)  **Hyperdivergent:** NP MaxP: 4805.92 (2133.10)  NP MandR: 4798.40 (2260.02)  OP + HP MaxP: 12394.94 (4311.77)  OP + HP MandR: 11514.50 (3907.02)  Lower HP MaxP: 4206.81 (1529.33)  Lower HP MandR: 3870.24 (1531.54) | No sample |  | NP: NS  OP+HP: NS  Lower HP: NS | ˃0.05  ˃0.05  ˃0.05 |
| Cabral et al. 2016 | OP (Median): 15909 | OP (Median): 10530 | No sample | No categorization | II < I (median) | Not reported |
| Castro-Silva et al. 2015 | OP (PP+GP): 14858 (5719) | OP (PP+GP): 10923 (3161) | OP (PP+GP): 19879 (5066) | No categorization | II < I < III | <0.05* |
| Chan et al. 2020  &  Vuong & Kang 2021 | Not reported | Not reported | Not reported | No categorization | NP: NS  OP: I < III & II < III  NP+OP: NS | 0.653  0.019*  0.114 |
| Chen et al. 2021 | PP: 12682.9 (4100.43) GP: 7159.7 (3020.93) OP (PP+GP): 19842.6 (5952.78) | PP: 10658. 7 (3425.23) GP: 6051.5 (3215.32) OP (PP+GP): 16710.1 (6007.16) | PP: 13801. 9 (5466.62) GP: 7773.5 (3912.60) OP (PP+GP): 21575.4 (8638.67) | No categorization | PP: II < III  GP: II < III  OP(PP+GP): II < III | 0.004*  0.049*  0.005* |
| Cho et al. 2021 | TP: 30158.75 (10480.58) | TP: 26744.75 (8143.759) | TP: 3534.49 (9880.75) | TP AV: 31620.77 (10550.90)  TP LA: 35129.54 (9281.13)  TP HA: 27156.20 (9187.96) | **Comparisons per Class**  TP: II < I < III  **Comparisons per divergence**  TP: HA < AV < LA | 0.004*  0.014* |
| Claudino et al. 2013 | NC + NP: no association with skeletal pattern  NP: no association with skeletal pattern  HP: no association with skeletal pattern  OP (PP + GP) + HP: smaller in greater ANB angles | | | No categorization | NC + NP: NS  NP: NS  HP: NS  OP(PP+GP)+HP & ANB: inverse | ˃0.05  ˃0.05  ˃0.05  <0.05* |
| Costa et al. 2021 | NP: 3474.91 (1144.11)  OP + HP: 11758.15 (3238.41)  Lower HP: 3404.95 (1082.09) | NP: 3660.90 (1002.89)  OP + HP: 11071.28 (3618.70)  Lower HP: 3277.02 (852.43) | NP: 3921.50 (1407.74)  OP + HP: 12683.75 (5204.65)  Lower HP: 3742.82 (1597.02) |  | NP: NS  OP+HP: NS  Lower HP: NS | > 0.05  > 0.05  > 0.05 |
| Di Carlo et al. 2014 | NP, PP, GP, TP: Not reported. Insignificant differences across the 3 groups.  TP decreases when ANB increases  TP increases when posterior face height increases | | | No categorization | NS | > 0.05 |
| Diwakar et al. 2021 | NP, OP, NP +OP: No significant association with ANB and SN-MP | | | No categorization |  | > 0.05 |
| El & Palomo 2011 | NC+NP : 6603.9 (2556.0)  OP : 7762.3 (2783.7) | NC+NP : 5304.1 (2325.2)  OP : 6292.8 (2709.9) | NC+NP : 5570.9 (1900.1)  OP : 8042.9 (2407.7 | No categorization | NC+NP: II < I  OP: II < I  OP: II < III | < 0.05*  < 0.05*  < 0.01* |
| El & Palomo 2013 | NC+NP: 6779.14 (3055.53)  OP: 6956.10 (1752.96) | **Maxillary Protrusion (MaxP)** NC+NP: 5209.62 (2401.73)  OP: 6638.10 (3126.54)  **Mandibular Retrusion (MandR)** NC + NP: 4962.80 (2130.31)  OP: 5837.80 (2812.30) | **Maxillary Retrusion (MaxR)**  NC+NP: 5585.58 (1918.99)  OP: 6978.32 (1804.46)  **Mandibular Protrusion (MandP)** NC + NP: 5541.95 (1999.47)  OP: 9332.60 (2468.67) | No categorization | NC+NP: II MandR<I  OP: II MandR < I  OP: I < III MandP  OP: II MaxP < III MandP  OP: II MandR < III MaxR  OP: II MandR < III MandP  OP: III MaxR < III MandP | < 0.05*  < 0.05*  < 0.01*  < 0.01*  < 0.05*  < 0.001*  < 0.01* |
| Elagib et al. 2022 | NP, OP and NP + OP were significantly associated (multiple regression) with SNB | | | | NP & SNB  OP & SNB  NP+OP & SNB | < 0.001*  0.004*  0.002* |
| Firwana et al. 2019 | OP (PP + GP) + HP  Total sample: 14.890 (5591)  Female: 13.800 (4048)  Male: 16.780 (6586) | OP (PP + GP) + HP  Total sample: 12.770 (4345)  Female: 11.760 (3732)  Male: 14.420 (4816) | No sample | No categorization | OP (PP + GP) + HP  Total sample: II < I  Female: II < I  Male: NS | <0.05* <0.05* NS |
| Gong et al. 2018 | Report only correlations with SNA, SNB and ANB controlling for FH-MP  NP: significantly correlated with SNB (r: 0.334)  PP: significantly correlated with SNB (r: 0.368)  GP: significantly correlated with SNB (r: 0.338) & ANB (r: -0.301)  TP (NP + PP + GP + HP): significantly correlated with SNB (r: 0.397) & ANB (r: -0.346) | | | | NP & SNB  PP & SNB  GP & SNB  GP & ANB inverse  TP & SNB  TP & ANB inverse | 0.025*  0.013*  0.023*  0.045*  0.007*  0.020* |
| Grauer et al. 2009 | NP: 9497 (2782.54)  OP + HP: 13163 (4425.17)  TP: 22660 (not available SD) | NP: 8771 (2797.25)  OP + HP: 9399 (4449.52)  TP: 18170 (not available SD) | NP: 8183 (2728.87)  OP + HP: 12187 (4436.84)  TP: 20318 (not available SD) | **Normodivergent**  NP: 9727 (2737.96)  OP + HP: 13228 (4597.49)  TP: 22485 (not available SD)  **Hypodivergent**  NP: 7930 (2713.56)  OP + HP: 10784 (4557.28)  TP: 18641 (not available SD)  **Hyperdivergent**  NP: 8850 (2762.66)  OP + HP: 10654 (4641.46)  TP: 20025 (not available SD) | **Comparisons per Class**  NP: NS  OP+HP: II < I  OP+HP: II < III  TP: not reported  **Comparisons per divergence**  NP: NS  OP+HP: NS  TP: NS | ˃0.05  <0.01*  0.05*  ˃0.05  ˃0.05  ˃0.05 |
| Gupta et al. 2016 | NC+NP: 30460 (6124.96) | NC+NP: 32.737 (57) | NC+NP: 29.112 (10.156) | NC+NP AV: 31925.35 (6144.92)  NC+NP LA: 30908.90 (6928.24) NC+NP HA: 30923.27 (8403.56) | **Comparisons per Class**  NC+NP: NS  **Comparisons per divergence**  NC+NP: NS | 0.572  0.936 |
| Habumugisha et al. 2022 | NP: 5661.98 (2269.91)  OP: 18838.41 (4367.04) NP+OP: 24500.4 (6035.87) | NP: 3843.31 (1158.02)  OP: 14750.96 (3741.34)  NP+OP: 18594.2 (4303.40) | No sample | No categorization | NP: II < I  OP: II < I  NP+OP: II < I | 0.005*  0.005*  0.002* |
| Hong et al. 2010 | NC: 13108.85 (3081.51)  NP: 2605.31 (913.47)  NC+NP: 15714.15 (3756.65) | NC: 11128.21 (3173.28)  NP: 2177.14 (648.86)  NC+NP: 13305.36 (3440.63) | No sample | No categorization | NC: NS  NP: NS  NC+NP: NS | 0.113  0.170  0.094 |
| Hong et al. 2011 | NP: 3050.58 (609.57)  OP+HP: 24827. 46 (7353.51)  TP:27878.04 (7646.36)  **Males**  NP: 3687.69 (966.46) OP+HP: 29680.45 (7023.32) TP: 33368.15 (7355.60) **Females**  NP : 2913.87 (650.08) OP + HP : 20772.4 (5233.45) TP: 23686.27 (5360.08) | No sample | NP: 4411.62 (1002.37)  OP+HP: 28169.54 (9149.27)  TP: 32581.15 (9750.70)  **Males**  NP: 4665.57 (975.66) OP+HP: 29396.79 (11841.21) TP: 34062.36 (12517.31) **Females**  NP: 4115.33 (990.3) OP+HP : 26737.75 (4515.21) TP: 30853.08 (4986.11) | No categorization | **Comparisons per Class**  NP: I< III  OP+HP: NS  TP: NS  **Comparisons per Class within sex**  **Males**  NP: I < III  OP+HP: NS  TP: NS  **Females**  NP: I < III  OP+HP: I < III  TP: I < III | 0.00*  0.56  0.28  0.02*  0.94 0.86  0.00* 0.00* 0.00* |
| Indriksone & Jakobsone 2015 | Significant correlations with skeletal parameters.  NP: with SN-MP (-0.146*), SNA (0.238**), presence of adenoids (-0.432**)  OP: with SNB (0.144*), presence of adenoids (-0.157**) | | | |  | 0.05*  0.01** |
| Iwasaki et al. 2009 | OP+HP: 6260.15 (3010.03) IO: 702.02 (1289.18) | No sample | OP+HP: 7326.89 (2593.40) IO: 2101.51 (2148.17) | No categorization | OP+HP: NS  IO: I < III | 0.295  0.016 |
| Iwasaki et al. 2017 |  | OP + HP: 9908 (2690)  IO: 1070 (1.21) | OP + HP: 9610 (2530)  IO: 1660 (1.93) | No categorization | NS  NS | 0.574 0.151 |
| Iwasaki et al. 2019 | OP+HP: 6440 (1800)  IO: 390 (720) | OP+HP: 6690 (1620)  IO: 1.570 (970) | OP+HP: 8960 (2900)  IO: 1180 (1700) | No categorization | OP+HP: I < III & II < III  IO: I < II | 0.001*  0.010* |
| Jadhav et al. 2020 | NP: 5550 (1920)  PP: 7250 (1440) GP: 5940 (1370)  NP+PP+GP: 17870 (7140) | NP: 2930 (1220)  PP: 4000 (1240) GP: 4740 (2220)  NP+PP+GP: 16500 (5350) | NP: 9650 (1290)  PP: 9250 (1170) GP: 7250 (1920)  NP+PP+GP: 22170 (5400) | No categorization | NP: II < I < III  PP: II < I < III  GP: II < I < III  NP+PP+GP:NS | 0.001*  <0.001*  <0.051  0.170 |
| Jayaratne et al. 2016 | No sample | PP: 7560 (2960)  GP: 4310 (2330) | PP: 10440 (4970)  GP: 6260 (4560) | No categorization | PP: II < III  GP: II < III | 0.005*  0.048* |
| Kikuchi et al. 2008 | OP+HP: 10190 (4040) | OP+HP: 11410 (4080) | OP+HP: 13960 (3160) | OP+HP AV: 12800 (3900)  OP+HP LA: 12740 (4270)  OP+HP HA: 11290 (3930) | **Comparisons per Class**  OP+HP: NS  **Comparisons per divergence**  OP+HP: NS | >0.05  >0.05 |
| Kim et al. 2010 | NC: 13479.62 (2547.12)  NP: 2620.77 (899.23)  PP: 1581.23 (509.83)  GP: 3278.00 (1101.55)  NC+ NP+PP+GP: 20959.62 (3611.26) | NC: 11124.00 (3302.82)  NP: 2138.38 (658.27)  PP: 1402.92 (662.49)  GP: 2498.77 (1095.03)  NC+NP+PP+GP: 17164.8 (4238.46) |  |  | NC: NS  NP: NS  PP: NS  GP: NS  NC+NP+PP+GR: II< I | 0.06  0.13  0.45  0.08  0.02* |
| Kochhar et al. 2021 | NC: 36407.36 (2526.59) NP: 5563.27 (1350.80) PP: 5322.45 (2124.81) GP: 5487.82 (2018.25) NC+ NP+PP+GP: 52780.91 (6435.84) | NC: 30446.00 (7060.88) NP: 4559.67 (1263.62) PP: 4213.89 (1291.90) GP: 5077.67 (1521.36) NC+ NP+PP+GP: 44297.22 (8662.49) |  |  | NC: NS  NP: NS  PP: NS  GP: NS  NC+ NP+PP+GP: II < I | 0.037 0.106 0.188 0.621 0.022* |
| Lee et al. 2019 | NP: 11521.9600 (3595.047089)  OP: 10946.05500 (2755.05117) | NP: 11925.5850 (1306.05734) OP: 8547.6125 (539.30330) | NP: 13167.2800(2089.73790) OP: 12126.3125(1689.51491) | No categorization | NP: NS  OP: NS | 0.643 0.067 |
| Li et al. 2015 | OP: 16280 (3620) |  | OP MaxR: 16470 (6750)  OP MandP: 24530 (5630) | No categorization | OP: I < III MandP  OP: III MaxR < III MandP | <0.01* |
| Mei et al. 2019 | **Normodivergent**  NP: 5249.99 (1255.79)  PP: 8474.67 (3570.06)  GP: 6074.17 (2647.23)  HP: 3601.68 (1331.97) TP: 23685.41 (7216.65) |  | **Hyperdivergent**  NP: 6678.24 (3114.87)  PP: 11365.31 (4820.31)  GP: 6042.74 (2292.79)  HP: 2793.11 (1031.16)  TP: 25764.80 (6660.94) | Class III group was HA  No further categorization | NP: NS  PP: I (AV) < III (HA)  GP: NS  HP: III (HA) < I (AV)  TP: NS | 0.058  0.033*  0.929  0.034*  0.338 |
| Mello et al. 2019 | PP: 8138.91 (3371.74)  GP: 3658.56 (2178.54)  OP: 11776.01 (5239.32) | PP: 6863.75 (2627.20)  GP: 3784.67 (1794.79)  OP: 10838.97 (3754.19) | PP: 9011.62 (34442.56)  GP: 4012.18 (2460.78)  OP: 13122.88 (5287.84) | No categorization | PP: II < I < III  GP: NS  OP: NS | <0.05*  >0.05  >0.05 |
| Miranda-Viana et al. 2021  Nasal breathers data | **NP (males)**  Normodivergent: 8170 (3740)  Hypodivergent: 9130 (2410) Hyperdivergent: 8510 (3290)  **NP (females)**  Normodivergent: 8820 (1790)  Hypodivergent: 8210 (2200)  Hyperdivergent: 7700 (840)  **OP + HP (males)**  Normodivergent: 11410 (7140)  Hypodivergent: 11990 (5620) Hyperdivergent: 10940 (6350)  **OP + HP (females)**  Normodivergent: 10620 (2850  Hypodivergent: 11410 (4460)) Hyperdivergent: 7280 (1270)  **TP (males)**  Normodivergent: 19600 (9500) Hypodivergent: 21130 (6090)  Hyperdivergent: 19440 (8610)  **TP (females)**  Normodivergent: 19440 (3750)  Hypodivergent: 18860 (6460) Hyperdivergent: 14990 (1630) | **NP (males)**  Normodivergent: 9180 (3840)  Hypodivergent: 9220 (6630) Hyperdivergent: 8990 (3380)  **NP (females)**  Normodivergent: 7860 (3320)  Hypodivergent: 6850 (2410) Hyperdivergent: 9100 (3090)  **OP + HP (males)**  Normodivergent: 11580 (4030)  Hypodivergent: 11920 (5650)  Hyperdivergent: 10790 (6990) **OP + HP (females)**  Normodivergent: 10980 (4850)  Hypodivergent: 11960 (3750) Hyperdivergent: 11060 (4400)  **TP (males)**  Hypodivergent: 21110 (8140) Normodivergent: 20760 (6420) Hyperdivergent: 19690 (9000)  **TP (females)**  Normodivergent: 18840 (7310)  Hypodivergent: 18450 (4760) Hyperdivergent: 20170 (6740) | **NP (males)**  Normodivergent: 10510 (2460)  Hypodivergent: 8670 (2950) Hyperdivergent: 10100 (2780)  **NP (females)**  Normodivergent: 8320 (4990)  Hypodivergent: 7570 (2670) Hyperdivergent: 4910 (2670)  **OP + HP (males)**  Normodivergent: 12680 (6080)  Hypodivergent: 14080 (6800) Hyperdivergent: 12500 (2450)  **OP + HP (females)**  Normodivergent: 13920 (6690)  Hypodivergent: 11990 (4600) Hyperdivergent: 6020 (2190)  **TP (males)**  Normodivergent: 23200 (8210) Hypodivergent: 22770 (8410)  Hyperdivergent: 22710 (2830)  **TP (females)**  Normodivergent: 22240 (11390)  Hypodivergent: 19570 (5450) Hyperdivergent: 10930 (4770) |  | Not reported | Not report |
| Moshajari et al. 2021 | NP: 6376.40 (2189.07) | NP: 6464.89 (2520.67) | NP: 6385.22 (2897.80) | NP AV: 6720.0 (2243.57)  NP LA: 6185.56 (2535.78)  NP HA: 7457.50 (2953.16) | **Comparisons per Class**  NP: NS  **Comparisons per divergence**  NP: NS | 0.987  0.331 |
| Nath et al 2021 | PP: inverse correlation ANB  GP: direct correlation SNB |  | GP: direct correlation ANB  GP: direct correlation SNB | No categorization | Not reported | <0.05* |
| Nejaim et al. 2018 | OP + HP: 14560.2 (5114.84) | OP + HP: 16110.8 (7054.28) | OP + HP: 18840.8 (6210.43) | OP+HP AV: 15080 (5476.16)  OP+HP LA: 17370.9 (6786.14)  OP+HP HA : 15720.9 (6452.53) | **Comparisons per Class**  OP+HP: I < II < III  **Comparisons per divergence**  OP+HP: LA > AV  OP+HP : LA > HA | < 0.05*  < 0.05* |
| Oh et al. 2018 | NP: 2954.89 (2122.92) OP+HP: 4448.44 (3019.59)  TP: 7663.47 (5067.19) | NP: 2421.29 (1342.77) OP+HP: 4189.29 (3072.85)  TP: 6398.15 (3994.07) | NP: 2722.93 (1843.75) OP+HP: 4745.63 (3995.34)  TP: 7519.97 (6114.42) | No categorization | NP: NS  OP+HP: NS  TP: NS | >0.05  >0.05  >0.05 |
| Paredes Vilchez et al. 2021 | NP: 5920 (2190)  OP: 15270 (5770)  HP: 4480 (1120)  **Males**  NP: 4788 (1747)  OP: 12375 (3278)  HP: 4863 (1537)  **Females**  NP: 6667 (2193)  OP: 17192 (6360)  HP: 4217 (683) | NP: 6470 (1650)  OP: 13080 (4040)  HP: 4570 (1420)  **Males**  NP: 6538 (1919)  OP: 13525 (4109)  HP: 5388 (1562)  **Females**  NP: 6417 (1541)  OP: 12783 (4146)  HP: 4017 (1062) | NP: 7370 (2330)  OP: 19140 (4590)  HP: 5650 (2160)  **Males**  NP: 7582 (1353)  OP:19682 (3356)  HP: 6845 (1786)  **Females**  NP: 7111 (3243)  OP:18467 (5921)  HP: 4189 (1640) | No categorization | **Comparisons per Class**  NP: NS  OP: II < I < III  HP: I < III  **Comparisons per Class within sex**  Not reported | 0.0916  0.0009*  0.0465* |
| Paul D et al. 2015 | NP : 10042.76 (2626.52)  OP+HP : 13240.12 (5112.14)  TP: 24237.07 (5622.03) | NP : 9344.87 (3167.04)  OP+HP : 7816.89 (2767.98)  TP: 18740.13 (5713.55) | No sample | No categorization | NP : NS  OP+HP : II < I  TP: II < I | 0.517  0.001*  0.013* |
| Paul P et al. 2022 | No categorization | No categorization | No categorization | OP+HP AV: 13716.13 (9267.45)  OP+HP LA: 17480.50 (6470.34)  OP+HP HA: 11058.27 (5159.36) | OP+HP : NS | >0.05 |
| Perrotti et al. 2021 | OP: 8963.4 (1620.4)  HP: 5642.1 (1336.2)  OP + HP: 14605 (2879) | OP: 5709.3 (1026.9)  HP: 3911.9 (851.1)  OP + HP: 9621 (1838) | OP: 12873.5 (1467.4)  HP: 6665.8 (801.4)  OP + HP: 19539 (2240) |  | OP: II < I < III  HP: II < I < III  OP+HP: II < I < III | < 0.05*  < 0.05*  < 0.05* |
| Ravello et al. 2020 & 2021 |  | TP: 26100 (7300) | TP: 30200 (9600) |  | TP: II < III | 0.034* |
| Saati et al. 2021 | OP+HP: 13354.9671 (6322.25499)  OP+HP AV: 12660.0992(7159.29245) OP+HP LA: 16733.9829 (7335.13512)  OP+HP HA: 12879.0766 (4664.76135) | OP+HP: 12936.4919 (5472.81991)  OP+HP AV: 14640.5638 (5461.01604) OP+HP LA: 14317.2743 (5442.52015)  OP+HP HA: 12381.7764 (5502.35606) | OP+HP: 13242.1600 (4583.23387)  OP+HP AV: 12941.3217 (5254.10102) OP+HP LA: 11443.9473 (3770.25317)  OP+HP HA: 14730.8062 (4486.93008) | OP+HP (per divergence)  OP+HP AV: 12980.4057 (6558.15532)  OP+HP LA: 13634.0353 (5812.58420)  OP+HP HA: 13172.0547 (5000.57235) | **Comparisons per Class**  OP+HP: NS  **Comparisons per divergence**  OP+HP: NS | 0.905  0.840 |
| Sharma et al. 2016 | **Normodivergent**  NP : 6910 (380)  OP+HP : 5930 (450) | **Hyperdivergent**  NP : 4640 (540)  OP+HP : 3730 (410) | No sample |  | NP: II HA < I AV  OP+HP: II HA < I AV | < 0.0001  < 0.0001 |
| Shokri et al. 2018, 2020, 2021 | NP: 2117.7 (1201.6) | NP: 2107.8 (844.7) | NP: 2826.6 (844.7) | Values not reported | **Comparisons per Class**  NP: I < III & II < III  **Comparisons per divergence**  NP: NS | < 0.05*  > 0.05 |
| Sparks (thesis) 2012 | OP+HP: 12170 (4210)  OP+HP AV: 13670 (5180)  OP+HP LA: 12490 (3820) OP+HP HA: 10350 (2690) | OP+HP: 10720 (3600)  OP+HP AV: 10840 (3180)  OP+HP LA: 10810 (4280) OP+HP HA: 10510 (3350) | OP+HP: 13260 (5140)  OP+HP AV: 12460 (5430)  OP+HP LA: 13570 (5060) OP+HP HA: 13730 (5000) | OP + HP AV: 12320 (4790)  OP + HP LA: 12290 (4520)  OP + HP HA: 11530 (4080) | II < III  I HA < I AV  II AV < I AV  II HA < I AV  II LA < I AV  II AV < III HA  II AV < III LA  I HA < III HA  I HA < III LA  II HA < III LA  II HA < III HA  II LA < III HA  II LA < III LA  **Comparisons per divergence**  OP+HP: NS | < 0.05*  < 0.05*  < 0.05*  < 0.05*  < 0.05*  < 0.05*  < 0.05*  < 0.05*  < 0.05*  < 0.05*  < 0.05*  < 0.05*  < 0.05*  > 0.05 |
| Tseng et al. 2021 | PP : 14994.7 (5557.95) GP : 7818.7 (3855.36) OP: 22812.3 (8793.61)  HP : 8377.7 (3001.73)  OP+HP: 31190.1 (10197.48) | PP : 11160.8 (4401.20)  GP : : 4894.1 (3024.09) OP: 16055.0 (6670.92)  HP : 6331.0 (3237.93)  OP+HP: 22386.0 (8956.80) | PP : 15467.1 (5422.58) GP : 6499.3 (3326.27) OP: 21966.4 (7167.82)  HP : 8846.3 (3409.61)  OP+HP: 30969.2 (9621.18) | No categorization | PP: II < I & II < III  GP: II < I  OP: II < I & II < III  HP: II < I & II < III  OP+HP: II < I & II < III | < 0.05*  < 0.05*  < 0.05*  < 0.05*  < 0.05* |
| Unal & Soydinc 2021 | TP: 14653.37 (5018.75)  TP AV: 14916.55 (5369.37)  TP LA: 15232.37 (5096.46)  TP HA: 13811.19 (4715.76) | TP: 12970.86 (4567.79)  TP AV: 13121.08 (4938.62)  TP LA: 13214.72 (4826.43)  TP HA: 12576.79 (4105.62) | TP. : 15108.83 (6772.64)  TP AV: 15590.02 (6915.51)  TP LA: 14887.5 (7068.23)  TP HA: 14848.96 (6654.6) |  | **Comparisons per Class**  TP: NS  **Comparisons per Class within divergence**  TP AV subgroups: NS  TP LA subgroups: NS  TP HA subgroups: NS  **Comparisons per divergence within Class**  TP Class III subgroups: NS  TP Class II subgroups: NS  TP Class I subgroups: NS | 0.153  0.386  0.366  0.4  0.901  0.965  0.650 |
| Unuvar et al. 2021a | TP: 22488.94 (7407.03)  TP AV: 23406.45 (8111.74)  TP LA: 24032.98 (7308.16)  TP HA: 20027.39 (6311.91) | TP: 18663.93 (5232.03)  TP AV: 18523.52 (8010.50)  TP LA: 20271.63 (6678.80)  TP HA: 17196.64 (6979.50) | TP: 22121.50 (5997.42)  TP AV: 22375.83 (6241.54)  TP LA: 23897.87 (7534.68)  TP HA: 20090.8 (6832.01) |  | **Comparisons per Class**  I> II & III > II  **Comparisons per divergence within Class**  Class I subgroups: I LA > I HA  Class II subgroups: II LA > II HA  Class III subgroups: III LA > III HA | < 0.01*  < 0.05*  < 0.05*  < 0.05* |
| Unuvar et al. 2021b | NP : 15262.58 (5515.48)  OP : 14363.56 (3036.34)  NP+OP: 29626.14 (6540.06) | **Mandibular retrusion (MandR)**  NP: 15092.71 (5473.82)  OP: 9509.35 (2305.31)  NP+OP: 24602.07 (6979.79)  **Maxillary protrusion (MaxP)**  NP : 16112.72 (3478.88)  OP : 14119.08 (4099.82)  NP+OP: 30231.80 (4975.67) |  |  | NP: NS  OP: I > II MandR  OP: II MaxP > II MandR  NP+OP: I > II MandR  NP+OP: II MaxP > II MandR | > 0.05  < 0.001*  < 0.001*  < 0.05*  < 0.05* |
| Vidal-Manyari et al. 2020 |  |  |  | **Normodivergent**  NP: 6962.64 (2259.99)  OP+HP: 16337.07 (6807.68)  TP: 23117.97 (8421.86)  **Hyperdivergent:**  NP: 7064.49 (2285.49)  OP+HP : 17834.94 (8685.36)  TP : 24963.45 (9811.01) | **Comparisons per divergence**  NP: NS  OP+HP: NS  TP: NS | 0.710  0.377  0.285 |
| Vidya et al. 2020 | NP: 9899.57 (2274.40)  OP: 8294.73 (1786.59) | NP : 7916.48 (2307.04)  OP: 6876.40 (2433.72) | NP : 8166.30 (1673.94)  OP : 10941.43 (2863.22) |  | NP: II < I  OP: I < III  OP: II < III | 0.04*  0.01*  <0.00* |
| Wan et al. 2019 | PP : 13826.2 (3545.50)  GP : 8076.4 (2634.88)  OP : 21968.1 (4872.51)  **Male**:  PP: 14067.11 (3937.72)  GP.: 8218.39 (2514.85) OP: 22361.99 (4928.55)  **Female**:  PP: 13546.71 (3165.40)  GP.: 7914.26 (2852.48) Op.: 21518.01 (4952.25) | PP : 7969.3 (2548.50)  GP : 4549.21 (2066.81)  OP : 12519.4 (3640.88)  **Male**:  PP: 7750.44 (3003.39)  GP: 4745.74 (2224.38) OP: 12496.91 (4133.30)  **Female**:  PP: 8136.71 (2222.13)  GP: 4398.93 (1994.04) OP: 12536.60 (3348.53) | No sample | No categorization | PP: II < I  GP: II < I  OP: II < I  Not reported | 0.00*  0.00*  0.00* |
| Wanzeler et al. 2019 |  |  |  | NP AV: not reported  NP LA: 41384.5  NP HA: 5336.3 | Not reported | Not reported |
| Yanagita et al. 2017 | **Adolescents**  NP: 3763.09 (1162.59). Significantly correlated with FH-NA (0.42), FH-NB (0.41), ANB (r: -0.07) and FH-MP (r: -0.06).  OP + HP: 8244.12 (1986.92). Significantly correlated with FH-NB (0.38), Go-Me (0.46), ANB (r: -0.31) and FH-MP (r: -0.38).  **Late adolescents**  NP: 5557.68 (1720.30). NS correlations with skeletal parameters.  OP + HP: 11018.75 (3781.16). NS correlations with skeletal parameters. | | | |  | <0.05* |
| Zheng et al. 2014 | NP: 5444.5 (1089.2)  OP: 8673.7 (2707.3)  HP: 5003.1 (2394.2)  OP+HP:13676.7 (3304.3  IO: 1100.1 (1020.7) | NP: 4054.2 (1196.1)  OP: 5207.5 (1662.1)  HP: 3334.4 (1982.9)  OP+HP: 8541.9 (2628.1)  IO: 812.1 (1164.3) | NP: 6053.5 (1148.5)  OP: 12505.6 (2403.9)  HP: 6885.7 (1538.6)  OP+HP: 19391.3 (3385.9)  IO: 3638.8 (3127.2) | No categorization | NP: II < II & II < III  OP: II < I < III  HP: II < I < III  OP+HP: II < I < III  IO: III> I & III> II | < 0.01*  < 0.01*  < 0.05*  < 0.01*  < 0.01* |
| Zou et al. 2020 | NP : 2863.12 (1312.43)  PP : 3845.69 (1175.15)  GP : 3125.45 (1314.25)  TP : 13021.25 (2958.15) | NP : 3893.45 (963.29)  PP : 3425.24 (1163.43)  GP : 2884.51 (1124.36)  TP : 9864.35 (3325.46) | NP : 2180.32 (896.48)  PP : 3212.62 (1158.46)  GP : 2687.64 (924.72)  TP :13769.25 (3856.64) |  | NP: II > I & II ˃ III  PP: NS  NS  TP: II < I & II < III | < 0.01*  0.061  0.266  < 0.01* |

SD: standard deviation; I: Class I, II: Class II; III: Class III; m: male ; f :female; vs: versus; NC : nasal cavity; NP: nasopharynx; GP: glossopharynx: OP = (PP+GP): oropharynx: HP: hypopharynx: TP: total pharynx (NP + PP + GP + HP) ; TA : total airway (NC + TP); IO: intraoral; AV: average=normodivergent; LA: low angle=hypodivergent; HA: high angle=hyperdivergent; MaxP: maxillary protrusion; MaxR: maxillary retrusion; MandR: mandibular retrusion; MandP: mandibular protrusion; *: statistically significant; NS: not significant

**Supplementary Table 8.** Risk of Bias (RoB) assessment of the included studies. Domains of the RoB assessment tool. 1. Were the aims/objectives of the study clear? 2. Were the criteria for inclusion in the sample clearly defined? 3. Were the study subjects and the setting described in detail (time, location, demographics)? 4. Was the sample size justified (sample size calculation)? 5. Were objective, standard criteria used for measurement of the condition (cut-off values for cephalometric parameters used to categorize the patients in the sagittal and vertical groups)? 6. Were confounding factors identified (presence of pathology, adenoids, tonsils, infection, nose or mouth breathing, baseline OSA assessment with questionnaires, age and maturation, sex, BMI, transversal discrepancies, vertical sub-categorization for the sagittal groups and vise-versa, conditions during image acquisition like providing patients with instructions for breathing & tongue posture, automatic or manual segmentation and thresholds, etc.)? 7. Were strategies to deal with confounding factors stated (analyses adjusted for confounders)? 8. Were the outcomes measured in a valid way (valid and accepted boundaries used also in the medical terminology)? 9. Were the outcomes measured in a reliable way (experience and/or training and/or calibration of the one-s measuring the airways)? 10. method error calculation performed? 11. Were the assessors blinded to the groups (the one-s measuring the airways being aware of the cephalometric values)? 12. Was appropriate statistical analysis used? 13. Is it clear what was used to determined statistical significance and/or precision estimates (*p* values, Confidence Intervals-CIs etc.)? 14. Were all specified outcome data adequately described and reported (incomplete reporting)? Y: Yes; U: Unclear; N: No.

| **Study** | **1** | **2** | **3** | **4** | **5** | **6** | **7** | **8** | **9** | **10** | **11** | **12** | **13** | **14** |
| --- | --- | --- | --- | --- | --- | --- | --- | --- | --- | --- | --- | --- | --- | --- |
| Abdelkarim 2020 | Y | U | U | Y | Y | N | N | N | U | N | U | Y | Y | Y |
| Aby 2020 | Y | Y | U | N | Y | N | N | N | Y | N | U | Y | Y | N |
| Alhammadi 2021 | Y | Y | Y | Y | Y | N | N | Y | U | Y | U | Y | Y | Y |
| Alves JrM 2012 | Y | Y | U | N | Y | N | N | Y | U | Y | U | Y | Y | Y |
| Alves PVM 2008 | Y | Y | U | N | Y | N | N | N | U | Y | U | Y | Y | Y |
| Anandarajah 2017 | Y | Y | U | N | Y | N | N | N | U | Y | U | N | Y | N |
| Bozzini 2018 | Y | Y | U | N | U | N | N | Y | Y | Y | U | Y | Y | Y |
| Brasil 2016 | Y | N | N | N | U | N | N | N | Y | Y | U | Y | Y | Y |
| Brito 2019 | Y | Y | Y | Y | Y | N | N | N | U | Y | U | Y | Y | Y |
| Cabral 2017 | Y | Y | U | N | U | N | N | Y | U | N | U | N | N | N |
| Castro-Silva 2015 | Y | Y | Y | N | U | N | N | N | U | Y | U | Y | Y | Y |
| Chan 2020, Vuong & Kang 2021 | Y | Y | Y | N | Y | N | N | Y | U | Y | U | Y | Y | N |
| Chen 2021 | Y | U | Y | N | Y | N | N | Y | U | N | U | Y | Y | Y |
| Cho 2022 | Y | Y | Y | N | Y | N | N | Y | U | Y | U | Y | Y | Y |
| Claudino 2013 | Y | Y | Y | Y | Y | N | N | N | U | Y | U | Y | Y | N |
| Costa 2022 | Y | Y | N | Y | U | N | N | N | Y | Y | U | Y | Y | Y |
| Di Carlo 2015 | Y | Y | U | N | Y | N | N | N | Y | Y | U | Y | Y | N |
| Diwakar | Y | Y | U | N | U | N | N | N | U | Y | U | Y | Y | N |
| El & Palomo 2011 | Y | Y | Y | N | Y | Y | Y | N | Y | Y | U | Y | Y | Y |
| El & Palomo 2013 | Y | Y | U | N | Y | Y | Y | N | Y | Y | U | Y | Y | Y |
| Elagib 2022 | Y | Y | Y | N | Y | Y | Y | N | U | Y | U | Y | Y | Y |
| Firwana 2019 | Y | Y | Y | N | Y | N | N | N | Y | Y | U | Y | Y | Y |
| Gong 2018 | Y | Y | Y | Y | Y | N | N | Y | U | Y | Y | Y | Y | Y |
| Grauer 2009 | Y | Y | Y | N | Y | N | N | N | U | Y | U | Y | Y | Y |
| Gupta 2016 | Y | Y | U | N | U | N | N | N | U | N | U | U | Y | Y |
| Habumugisha 2022 | Y | Y | Y | Y | Y | N | N | Y | U | Y | U | Y | Y | Y |
| Hong 2010 | Y | Y | Y | N | Y | N | N | Y | U | Y | U | Y | Y | Y |
| Hong 2011 | Y | Y | U | N | U | N | N | Y | U | Y | U | Y | Y | Y |
| Indriskone & Jakobsone 2015 | Y | Y | U | N | U | N | N | Y | U | Y | U | Y | Y | N |
| Iwasaki 2009 | Y | Y | Y | N | Y | N | N | Y | U | Y | U | Y | Y | Y |
| Iwasaki 2017 | Y | Y | Y | N | Y | N | N | Y | U | N | U | Y | Y | Y |
| Iwasaki 2019 | Y | Y | Y | Y | Y | N | N | Y | U | Y | U | Y | Y | Y |
| Jadhav 2020 | Y | Y | U | N | Y | N | N | Y | U | N | U | Y | Y | Y |
| Jayaratne 2016 | Y | Y | Y | N | U | N | N | Y | U | Y | U | Y | Y | Y |
| Kikuchi 2008 | Y | N | U | N | Y | N | N | N | U | N | U | Y | Y | Y |
| Kim YJ 2010 | Y | Y | Y | N | Y | N | N | Y | U | Y | U | Y | Y | Y |
| Kochlar 2021 | Y | U | U | N | Y | N | N | Y | U | N | U | Y | Y | Y |
| Lee 2019 | Y | N | N | N | N | N | N | N | U | N | U | Y | Y | Y |
| Li 2015 | Y | Y | Y | N | Y | N | N | Y | U | Y | U | Y | Y | Y |
| Mei 2019 | Y | Y | U | N | Y | N | N | Y | U | Y | U | Y | Y | Y |
| Mello 2019 | Y | Y | Y | Y | Y | N | N | Y | U | Y | U | Y | Y | Y |
| Miranda-Viana 2021 | Y | Y | U | N | Y | N | N | N | Y | Y | U | Y | Y | N |
| Moshajari 2020 | Y | Y | U | N | Y | N | N | Y | U | N | U | Y | Y | Y |
| Nath 2021 | Y | Y | Y | Y | Y | N | N | Y | Y | N | U | U | Y | N |
| Nejaim 2018 | Y | Y | Y | N | U | N | N | N | Y | Y | U | Y | Y | Y |
| Oh 2011 | Y | Y | Y | N | Y | N | N | N | U | Y | U | Y | Y | Y |
| Paredes Vilchez 2021 | Y | Y | U | N | Y | N | N | N | U | N | U | Y | Y | Y |
| Paul D 2015 | Y | Y | U | Y | Y | N | N | N | U | Y | U | Y | Y | Y |
| Paul P 2022 | Y | N | U | Y | Y | N | N | N | U | N | U | Y | Y | Y |
| Perrotti 2021 | Y | Y | U | N | Y | N | N | N | Y | N | U | Y | Y | Y |
| Ravelo 2020 & 2021 | Y | U | Y | N | Y | N | N | N | U | Y | U | Y | Y | Y |
| Saati 2021 | Y | Y | U | N | Y | N | N | Y | U | Y | U | Y | Y | Y |
| Sharma 2016 | Y | U | U | N | Y | N | N | Y | U | Y | U | Y | Y | Y |
| Shokri 2018, 2020, 2021 | Y | Y | N | Y | Y | N | N | N | Y | Y | U | Y | Y | Y |
| Sparks 2012 | Y | N | N | Y | Y | N | N | Y | U | Y | U | Y | Y | Y |
| Tseng 2021 | Y | Y | Y | N | Y | Y | Y | Y | U | N | U | Y | Y | Y |
| Unal 2021 | Y | Y | U | N | U | N | N | N | U | N | U | Y | Y | Y |
| Unuvar 2021 a | Y | Y | U | N | Y | N | N | N | U | Y | U | Y | Y | Y |
| Unuvar 2021 b | Y | Y | Y | N | Y | N | N | N | U | Y | U | Y | Y | Y |
| Vidal-Manyani 2020 | Y | Y | Y | N | Y | Y | Y | N | Y | Y | U | Y | Y | Y |
| Vidya 2020 | Y | Y | U | N | U | N | N | N | U | N | U | Y | Y | Y |
| Wan 2019 | Y | Y | Y | N | Y | Y | Y | Y | U | Y | U | Y | Y | Y |
| Wanzeler 2019 | Y | U | N | N | N | N | N | N | Y | Y | U | N | Y | N |
| Yanagita 2017 | Y | Y | Y | N | U | N | N | Y | U | Y | U | Y | Y | N |
| Zheng 2014 | Y | Y | Y | N | Y | N | N | N | U | Y | U | Y | Y | Y |
| Zou 2020 | Y | Y | Y | N | U | N | N | Y | U | N | U | Y | Y | Y |

**Supplementary Table 9.** Colour-coded Risk of Bias (RoB) assessment of the included studies.

**Supplementary Table 10.** Summary risk of bias among included studies.

| **Nr** | **Question** | **Yes** | **No** | **Unclear** |
| --- | --- | --- | --- | --- |
| 1 | Were the aims/objectives of the study clear? | **100% (66)** | **0% (0)** | **0% (0)** |
| 2 | Were the criteria for inclusion in the sample clearly defined? | **83% (55)** | **8% (5)** | **9% (6)** |
| 3 | Were the study subjects and the setting described in detail (time, location, demographics)? | **48% (32)** | **9% (6)** | **42% (28)** |
| 4 | Was the sample size justified (sample size calculation)? | **21% (14)** | **79% (52)** | **0% (0)** |
| 5 | Were objective, standard criteria used for measurement of the condition  (cut-off values for cephalometric parameters used to categorize the patients in the sagittal and vertical groups)? | **74% (49)** | **3% (2)** | **23% (15)** |
| 6 | Were confounding factors identified (presence of pathology, adenoids, tonsils, infection, nose or mouth breathing, baseline sleep apnea assessment with questionnaires, age and maturation, sex, BMI, transversal discrepancies, vertical sub-categorization for the sagittal groups and vise-versa, conditions during image acquisition like providing patients with instructions for breathing & tongue posture, automatic or manual segmentation and thresholds, etc.)? | **9% (6)** | **91% (60)** | **0% (0)** |
| 7 | Were strategies to deal with confounding factors stated (analyses adjusted for confounders)? | **9% (6)** | **91% (60)** | **0% (0)** |
| 8 | Were the outcomes measured in a valid way (valid and accepted boundaries used also in the medical terminology)? | **47% (31)** | **53% (35)** | **0% (0)** |
| 9 | Were the outcomes measured in a reliable way (experience and/or training and/or calibration of the ones measuring the airways)? | **23% (15)** | **0% (0)** | **77% (51)** |
| 10 | Method error calculation performed? | **71% (47)** | **29% (19)** | **0% (0)** |
| 11 | Were the assessors blinded to the groups (the ones measuring the airways being aware of the cephalometric values)? | **2% (1)** | **0% (0)** | **98% (65)** |
| 12 | Was appropriate statistical analysis used? | **92% (61)** | **5% (3)** | **3% (2)** |
| 13 | Is it clear what was used to determined statistical significance and/or precision estimates (*p* values, Confidence Intervals-CIs etc.)? | **98% (65)** | **2% (1)** | **0% (0)** |
| 14 | Were all specified outcome data adequately described and reported (incomplete reporting)? | **82% (54)** | **18% (12)** | **0% (0)** |

**Supplementary Table 11.** Studies contributing with data for each outcome.

| **Total pharynx** | **Sagittal** | **Vertical** |
| --- | --- | --- |
| 1.       Aby et al. 2020 | ● |  |
| 2.       Brasil et al. 2016 | ● | ● |
| 3.       Cho et al. 2022 | ● | ● |
| 4.       Hong et al. 2011 | ● |  |
| 5.       Mei et al. 2019 |  | ● |
| 6.       Miranda-Viana et al. 2021 | ● | ● |
| 7.       Oh et al. 2011 | ● |  |
| 8.       Paul et al. 2015 | ● |  |
| 9.       Ravelo et al. 2020 | ● |  |
| 10.    Unal et al. 2021 | ● | ● |
| 11.    Unuvar et al. 2021 a | ● | ● |
| 12.    Vidal-Manyari et al. 2020 |  | ● |
| 13.    Zou et al. 2020 | ● |  |
|  |  |  |
| **Nasal cavity** |  |  |
| 1.       Gupta et al. 2016 |  | ● |
| 2.       Hong et al. 2010 | ● |  |
| 3.       Kim et al. 2010 | ● |  |
| 4.       Kochhar et al. 2021 | ● |  |
|  |  |  |
| **Nasopharynx** |  |  |
| 1.       Alhammadi et al. 2021 |  | ● |
| 2.       Brasil 2016 | ● | ● |
| 3.       Brito et al. 2019 |  | ● |
| 4.       Claudino et al. 2013 | ● |  |
| 5.       Costa et al. 2022 | ● |  |
| 6.       El & Palomo 2011 | ● |  |
| 7.       El & Palomo 2013 | ● |  |
| 8.       Elagib et al. 2022 | ● | ● |
| 9.       Grauer et al. 2009 | ● | ● |
| 10.    Habumugisha et al. 2022 | ● |  |
| 11.    Hong et al. 2010 | ● |  |
| 12.    Hong et al. 2011 | ● |  |
| 13.    Indriksone & Jakobsone 2015 | ● | ● |
| 14.    Jadhav et al. 2019 | ● |  |
| 15.    Kim et al. 2010 | ● |  |
| 16.    Kochhar et al. 2021 | ● |  |
| 17.    Mei et al. 2019 |  | ● |
| 18.    Miranda Viana et al. 2021 | ● | ● |
| 19.    Moshajari et al. 2020 | ● | ● |
| 20.    Oh et al. 2011 | ● |  |
| 21.    Paredes et al. 2021 | ● |  |
| 22.    Paul et al. 2015 | ● |  |
| 23.    Sharma et al. 2016 |  | ● |
| 24.    Shokri et al. 2018 | ● |  |
| 25.    Unuvar et al. 2021 b | ● |  |
| 26.    Vidal-Manyari et al. 2020 |  | ● |
| 27.    Vidya et al. 2020 | ● |  |
| 28.    Zheng et al. 2014 | ● |  |
| 29.    Zou et al. 2020 | ● |  |
|  |  |  |
| **Nasal cavity + nasopharynx** |  |  |
| 1.       Alves et al. 2008 | ● |  |
| 2.       Gupta et al. 2016 | ● |  |
| 3.       Hong et al. 2010 | ● |  |
|  |  |  |
| **Palatopharynx** |  |  |
| 1.       Alves et al. 2008 | ● |  |
| 2.       Chen et al. 2021 | ● |  |
| 3.       Claudino et al. 2013 | ● |  |
| 4.       De Mello et al. 2019 | ● |  |
| 5.       Jadhav et al. 2019 | ● |  |
| 6.       Jayaratne & Zwahlen 2016 | ● |  |
| 7.       Kim et al. 2010 | ● |  |
| 8.       Kochhar et al. 2021 | ● |  |
| 9.       Mei et al. 2019 |  | ● |
| 10.       Tseng et al. 2021 | ● |  |
| 11.    Wan et al. 2019 | ● |  |
| 12.    Zou et al. 2020 | ● |  |
|  |  |  |
| **Glossopharynx** |  |  |
| 1.       Chen et al. 2021 | ● |  |
| 2.       Claudino et al. 2013 | ● |  |
| 3.       de Mello et al. 2019 | ● |  |
| 4.       Jadhav 2019 | ● |  |
| 5.       Jayaratne & Zwahlen 2016 | ● |  |
| 6.       Kim et al. 2010 | ● |  |
| 7.       Kochhar et al. 2021 | ● |  |
| 8.       Mei et al. 2019 |  | ● |
| 9.       Tseng et al. 2021 | ● |  |
| 10.    Wan et al. 2019 | ● |  |
| 11.    Zou et al. 2020 | ● |  |
|  |  |  |
| **Oropharynx** |  |  |
| 1.       Abdelkarim 2021 | ● |  |
| 2.       Alves Jr et al. 2012 | ● |  |
| 3.       Castro-Silva et al. 2015 | ● |  |
| 4.       Chen et al. 2021 | ● |  |
| 5.       de Mello et al. 2019 | ● |  |
| 6.       El & Palomo et al. 2011 | ● |  |
| 7.       El & Palomo et al. 2013 | ● |  |
| 8.       Elagib et al. 2022 | ● | ● |
| 9.       Habumugisha et al. 2022 | ● |  |
| 10.    Indriksone & Jakobsone 2015 | ● | ● |
| 11.    Jayaratne & Zwahlen 2016 | ● |  |
| 12.    Miranda Viana et al. 2021 | ● | ● |
| 13.    Paredes et al. 2021 | ● |  |
| 14.    Perrotti al. 2021 | ● |  |
| 15.    Tseng et al. 2021 | ● |  |
| 16.    Unuvar et al. 2021 b | ● |  |
| 17.    Vidya et al. 2021 | ● |  |
| 18.    Wan et al. 2019 | ● |  |
| 19.    Zheng et al. 2014 | ● |  |
|  |  |  |
| **Glosso + hypopharynx** |  |  |
| 1.       Alves et al. 2008 | ● |  |
|  |  |  |
| **Hypopharynx** |  |  |
| 1.       Brito et al. 2019 |  | ● |
| 2.       Claudino et al. 2013 | ● |  |
| 3.       Costa et al. 2022 | ● |  |
| 4.       Mei et al. 2019 |  | ● |
| 5.       Paredes et al. 2021 | ● |  |
| 6.       Perrotti et al. 2021 | ● |  |
| 7.       Tseng et al. 2021 | ● |  |
| 8.       Zheng et al. 2014 | ● |  |
|  |  |  |
| **~~Nasopharynx + oropharynx~~** |  |  |
| ~~2.       Elagib et al. 2022~~ |  |  |
|  |  |  |
| **Nasopharynx + oropharynx** |  |  |
| 1.       Elagib et al. 2022 | ● |  |
| 2.       Habumugisha et al. 2022 | ● |  |
| 3.       Jadhav et al. 2019 | ● |  |
| 4.       Unuvar et al. 2021 b | ● |  |
|  |  |  |
| **Oropharyunx + hypopharynx** |  |  |
| 1.       Alves et al. 2008 | ● |  |
| 2.       Bozzini et al. 2018 | ● |  |
| 3.       Brasil et al. 2016 | ● | ● |
| 4.       Brito et al. 2019 |  | ● |
| 5.       Claudino et al. 2013 | ● |  |
| 6.       Costa et al. 2022 | ● |  |
| 7.       Firwana et al. 2019 | ● |  |
| 8.       Grauer et al. 2009 | ● | ● |
| 9.       Hong et al. 2011 | ● |  |
| 10.    Iwasaki et al. 2009 | ● |  |
| 11.    Iwasaki et al. 2017 | ● |  |
| 12.    Iwasaki et al. 2019 | ● |  |
| 13.    Kikuchi et al. 2008 | ● | ● |
| 14.    Li et al. 2015 | ● |  |
| 15.    Nejaim et al. 2018 | ● | ● |
| 16.    Oh et al. 2011 | ● |  |
| 17.    Paul et al. 2015 | ● | ● |
| 18.    Perrotti et al. 2021 | ● |  |
| 19.    Saati et al. 2021 | ● | ● |
| 20.    Sparks et al. 2012 | ● | ● |
| 21.    Tseng et al. 2021 | ● |  |
| 22.    Vidal-Manyari et al. 2020 |  | ● |
| 23.    Zheng et al. 2014 | ● |  |
|  |  |  |
| **Intraoral volume** |  |  |
| 1.       Iwasaki et al. 2009 | ● |  |
| 2.       Iwasaki et al. 2017 | ● |  |
| 3.       Iwasaki et al. 2019 | ● |  |
| 4.       Zheng et al. 2014 | ● |  |

**Supplementary Table 12.** Decomposition of network components with design-by-treatment interaction & comparison of direct vs indirect evidence for every comparison.

| **Dimension** | **Outcome** | **Design-by-treatment P** | **Comparison** | **Direct estimate** | **Indirect estimate** | **Direct-indirect Difference** | **Direct-indirect P** |
| --- | --- | --- | --- | --- | --- | --- | --- |
| Sagittal | Total pharynx | 0.49 | Cl2:Cl1 | -2297.01 | 1214.07 | -3511.08 | 0.19 |
|  |  |  | Cl3:Cl1 | 960.44 | 520.22 | 440.22 | 0.82 |
|  |  |  | Cl2:Cl3 | -3219.31 | -8020.81 | 4801.50 | 0.18 |
|  |  |  |  |  |  |  |  |
| Sagittal | Nasopharynx | 0.20 | Cl2:Cl1 | -504.51 | 3281.24 | -3785.75 | <0.001 |
|  |  |  | Cl3:Cl1 | 197.22 | -2646.12 | 2843.34 | <0.001 |
|  |  |  | Cl2:Cl3 | -446.40 | -2086.96 | 1640.57 | 0.002 |
|  |  |  |  |  |  |  |  |
| Sagittal | Palatopharynx | 0.58 | Cl2:Cl1 | -2116.78 | - | - | - |
|  |  |  | Cl3:Cl1 | 947.47 | -736.52 | 1683.98 | 0.51 |
|  |  |  | Cl2:Cl3 | -2676.65 | - | - | - |
|  |  |  |  |  |  |  |  |
| Sagittal | Glosopharynx | 0.63 | Cl2:Cl1 | - |  |  |  |
|  |  |  | Cl3:Cl1 | - |  |  |  |
|  |  |  | Cl2:Cl3 | - |  |  |  |
|  |  |  |  |  |  |  |  |
| Sagittal | Oropharynx | 0.23 | Cl2:Cl1 | - |  |  |  |
|  |  |  | Cl3:Cl1 | - |  |  |  |
|  |  |  | Cl2:Cl3 | - |  |  |  |
|  |  |  |  |  |  |  |  |
| Sagittal | Hypopharynx | - | Cl2:Cl1 | -602.52 | - | - | - |
|  |  |  | Cl3:Cl1 | 1190.56 | - | - | - |
|  |  |  | Cl2:Cl3 | -1854.39 | - | - | - |
|  |  |  |  |  |  |  |  |
| Sagittal | Oro- & hypopharynx | 0.19 | Cl2:Cl1 | -1948.18 | 2536.49 | -4484.67 | 0.005 |
|  |  |  | Cl3:Cl1 | 2791.51 | -2715.35 | 5506.86 | 0.001 |
|  |  |  | Cl2:Cl3 | -3590.15 | -6760.49 | 3170.34 | 0.12 |
|  |  |  |  |  |  |  |  |
| Vertical | Nasopharynx | 0.65 | Hyper:Hypo | 10.17 | - | - | - |
|  |  |  | Hyper:Normo | -427.42 | - | - | - |
|  |  |  | Hypo:Normo | -309.33 | - | - | - |
|  |  |  |  |  |  |  |  |
| Vertical | Total pharynx | 0.03 | Hyper:Hypo | -2224.81 | - | - | - |
|  |  |  | Hyper:Normo | -858.87 | - | - | - |
|  |  |  | Hypo:Normo | 825.50 | - | - | - |
|  |  |  |  |  |  |  |  |
| Vertical | Oro- & hypopharynx | 0.20 | Hyper:Hypo | - | - | - | - |
|  |  |  | Hyper:Normo | - | - | - | - |
|  |  |  | Hypo:Normo | - | - | - | - |

CI, skeletal Class; Hyper, hyperdivergent; Hypo, hypodivergent; Normo, normodivergent.

**Supplementary Table 13.** Comparison of network / pairwise analysis for outcomes with evidence of inconsistency. Bold values indicate statistically significant results at the 5% level.

| **Dimension** | **Outcome** | **Comparison** | **Network analysis** | **Pairwise analysis** |
| --- | --- | --- | --- | --- |
| Sagittal | Nasopharynx | [Class 2]-[Class 1] | -419.32  (-937.46, 98.83) | **-456.30**  **(-863.10, -49.51)** |
|  |  | [Class 3]-[Class 1] | 115.00 (-456.05, 686.06) | 205.00  (-478.93, 888.94) |
|  |  | [Class 3]-[Class 2] | 534.32  (-34.55, 1103.19) | 438.56  (-530.97, 1408.08) |
|  |  |  |  |  |
| Sagittal | Oro- & hypopharynx | [Class 2]-[Class 1] | **-1489.17**  **(-2948.17, -30.17)** | **-1921.05**  **(-3531.26, -310.84)** |
|  |  | [Class 3]-[Class 1] | **2377.57**  **(957.26, 3797.88)** | **2805.48**  **(1424.22, 4186.75)** |
|  |  | [Class 3]-[Class 2] | **3866.74**  **(2441.24, 5292.24)** | **3587.65**  **(1586.38, 5588.93)** |
|  |  |  |  |  |
| Vertical | Total pharynx | [Hypodivergent]-[Normodivergent] | 1080.83  (-14.51, 2176.17) | 825.50  (-365.22, 2016.22) |
|  |  | [Hyperdivergent]-[Normodivergent] | -876.75  (-1921.53, 168.04) | -826.30  (-2484.26, 831.65) |
|  |  | [Hyperdivergent]-[Hypodivergent] | **-1957.58**  **(-3063.32, -851.83)** | -2600.06  (-5678.81, 478.68) |

CI, skeletal Class.

**Supplementary Table 14.** Post hoc sensitivity analyses according to study precision (standard error). Bold values indicate statistically significant results at the 5% level.

| **Dimension** | **Outcome** | **Comparison** | **50% least**  **precise** | **50% most**  **precise** | **P** |
| --- | --- | --- | --- | --- | --- |
| Sagittal | Nasopharynx | [Class 2]-[Class 1] | -979.00  (-1677.48, -280.52) | -126.23  (-576.53, 324.08) | 0.02 |
|  |  | [Class 3]-[Class 1] | 31.98  (-1370.94, 1434.89) | 355.17  (-224.37, 934.71) | 0.62 |
|  |  | [Class 3]-[Class 2] | -96.57  (-757.72, 564.59) | 1038.47  (-1056.97, 3133.91) | 0.22 |
|  |  |  |  |  |  |
| Sagittal | Oro- & hypopharynx | [Class 2]-[Class 1] | -2835.79  (-6248.15, 576.57) | -1235.26  (-3004.29, 533.77) | 0.31 |
|  |  | [Class 3]-[Class 1] | **2757.67**  **(414.64, 5100.71)** | **2829.60**  **(666.17, 4993.03)** | 0.96 |
|  |  | [Class 3]-[Class 2] | **2142.61**  **(655.34, 3629.89)** | **4636.67**  **(414.84, 8858.51)** | 0.17 |
|  |  |  |  |  |  |
| Vertical | Nasopharynx | [Hypodivergent]-[Normodivergent] | -542.24  (-1876.74, 792.27) | -34.31  (-1145.92, 1077.29) | 0.30 |
|  |  | [Hyperdivergent]-[Normodivergent] | 174.72  (-622.62, 972.06) | -867.47  (-2062.85, 327.91) | 0.05 |
|  |  | [Hyperdivergent]-[Hypodivergent] | 331.19  (-1227.51, 1889.90) | -308.04  (-1417.69, 801.61) | 0.25 |

.

**Supplementary Table 15.** Network meta-regression results (P values) for outcomes with ≥ 10 studies. Bold values indicate statistically significant results at the 10% level.

| **Dimension** | **Outcome** | **Studies** | **Age-group** | **Mean age** | **Male %** |
| --- | --- | --- | --- | --- | --- |
| Sagittal | Nasopharynx | 24 | **0.06** | 0.70 | 0.32 |
|  |  |  |  |  |  |
| Sagittal | Palatopharynx | 11 | 0.41 | 0.65 | 0.42 |
|  |  |  |  |  |  |
| Sagittal | Glossopharynx | 10 | 0.22 | 0.58 | 0.61 |
|  |  |  |  |  |  |
| Sagittal | Oropharynx | 19 | 0.39 | 0.41 | 0.46 |
|  |  |  |  |  |  |
| Sagittal | Total pharynx | 11 | 0.27 | 0.35 | 0.83 |
|  |  |  |  |  |  |
| Sagittal | Oro- & hypopharynx | 21 | **0.09** | 0.78 | 0.26 |
|  |  |  |  |  |  |
| Vertical | Nasopharynx | 11 | 0.19 | **0.07** | 0.77 |

.

**Supplementary Figure 1.** Network graph for the effect of sagittal discrepancies on the volume of the nasopharynx.


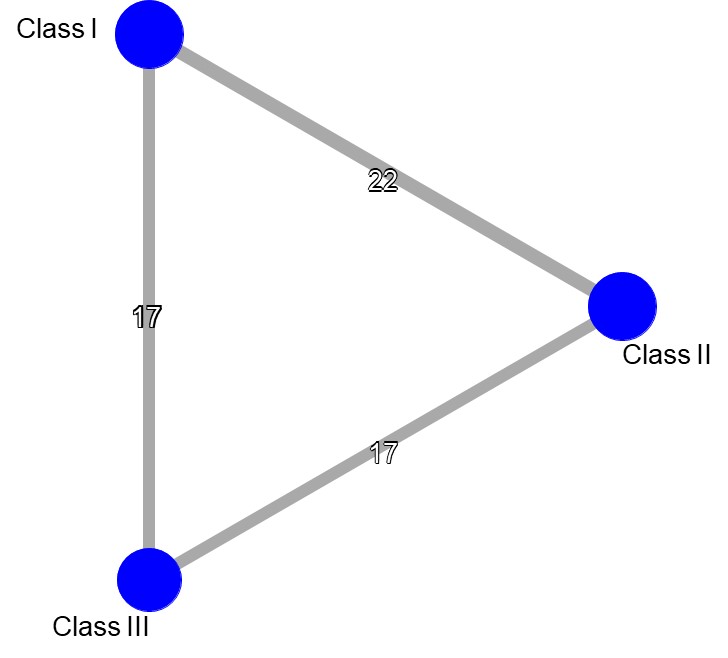


.

**Supplementary Figure 2.** Network graph for the effect of sagittal discrepancies on the volume of the palatopharynx.


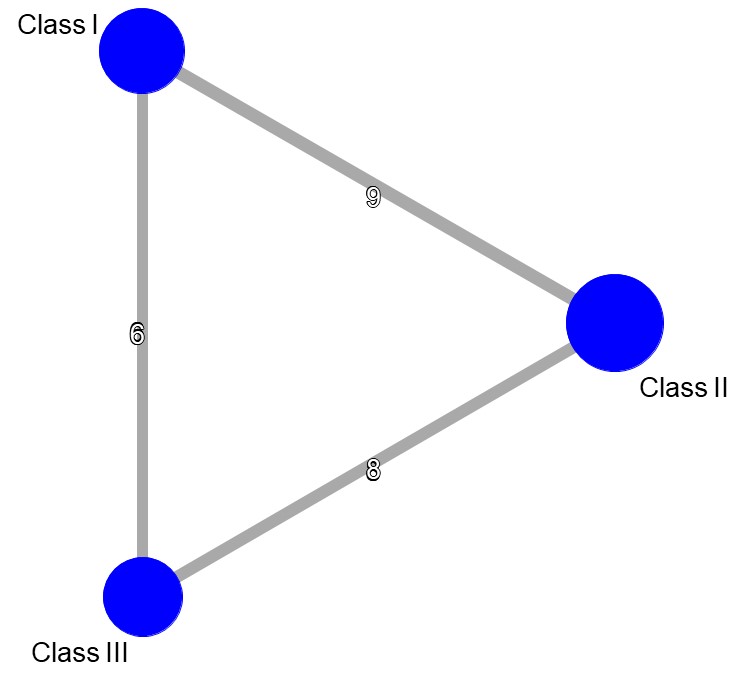


.

**Supplementary Figure 3.** Network graph for the effect of sagittal discrepancies on the volume of the glossopharynx.


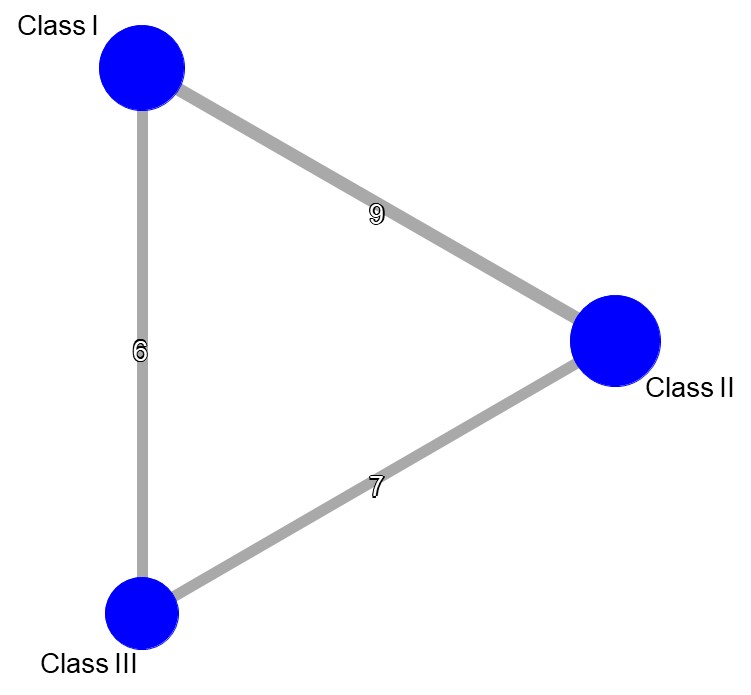


.

**Supplementary Figure 4.** Network graph for the effect of sagittal discrepancies on the volume of the oropharynx.


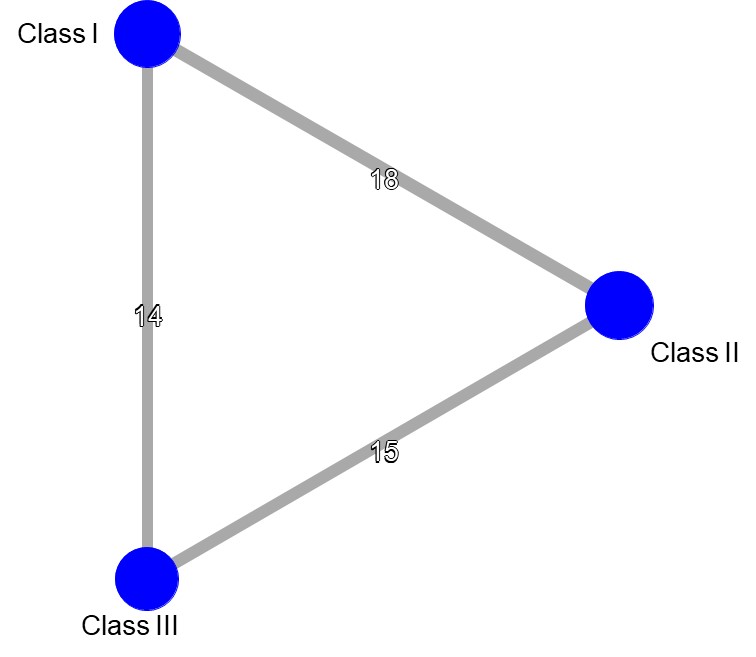


.

**Supplementary Figure 5.** Network graph for the effect of sagittal discrepancies on the volume of the hypopharynx.


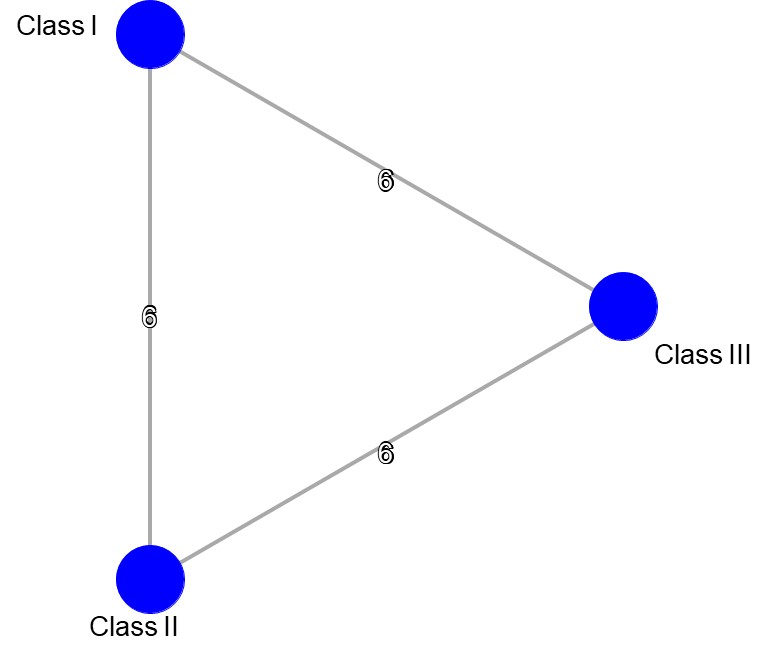


.

**Supplementary Figure 6.** Network graph for the effect of sagittal discrepancies on the volume of the total pharynx.


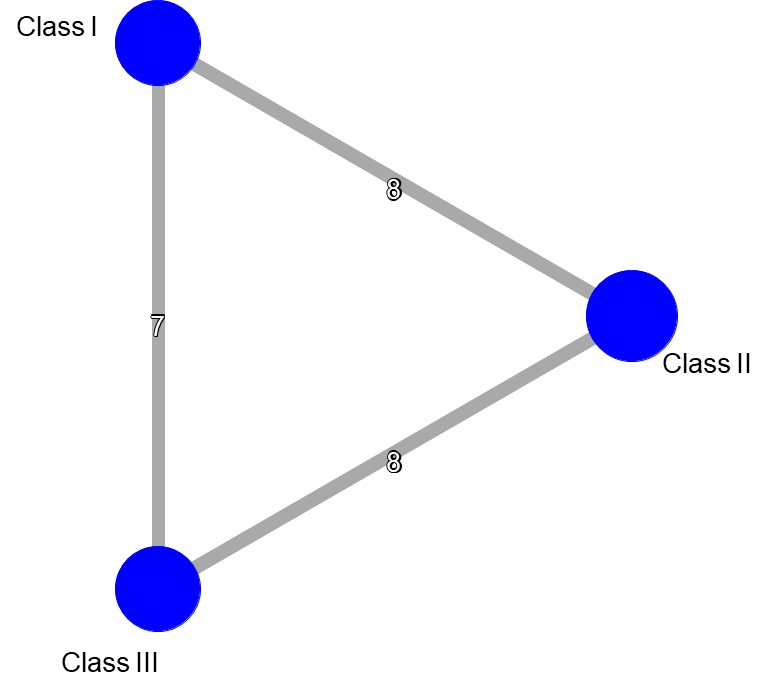


.

**Supplementary Figure 7.** Network graph for the effect of sagittal discrepancies on the volume of the oro & hypopharynx.


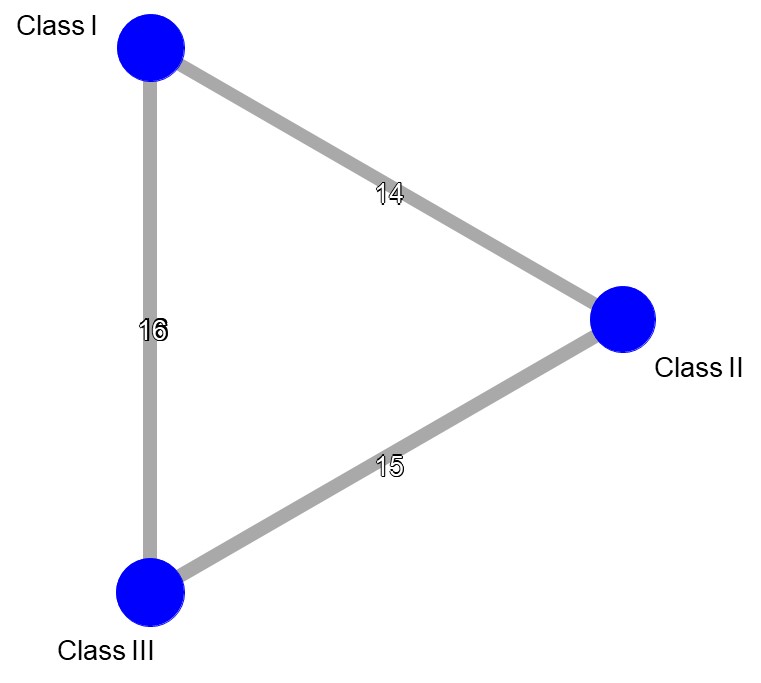


.

**Supplementary Figure 8.** Network graph for the effect of vertical discrepancies on the volume of the oro & hypopharynx.


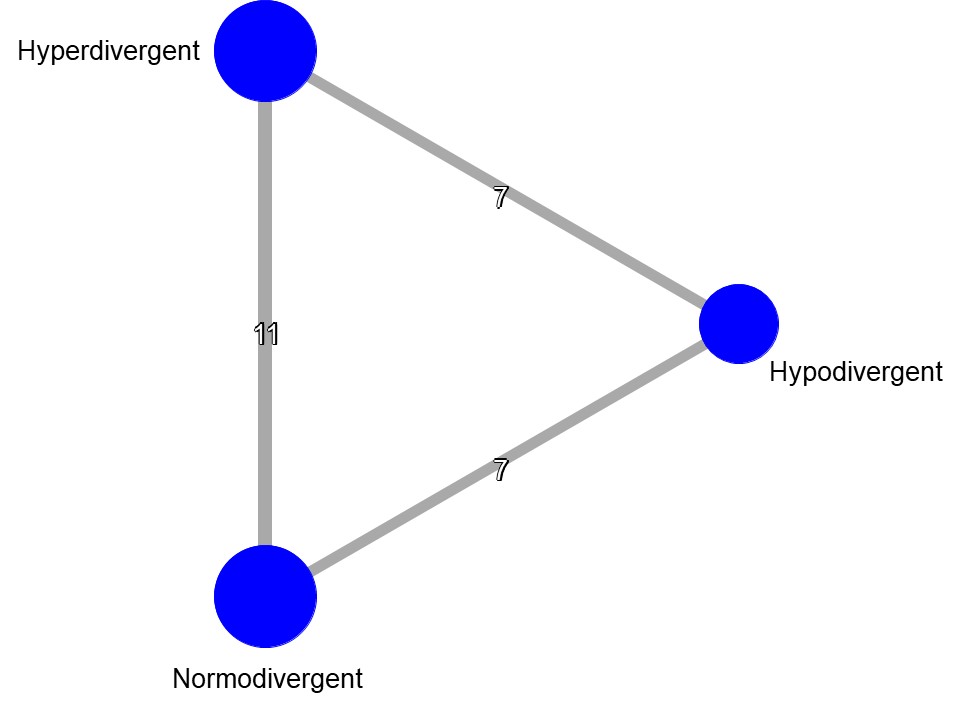


.

**Supplementary Figure 9.** Network graph for the effect of vertical discrepancies on the volume of the total pharynx.


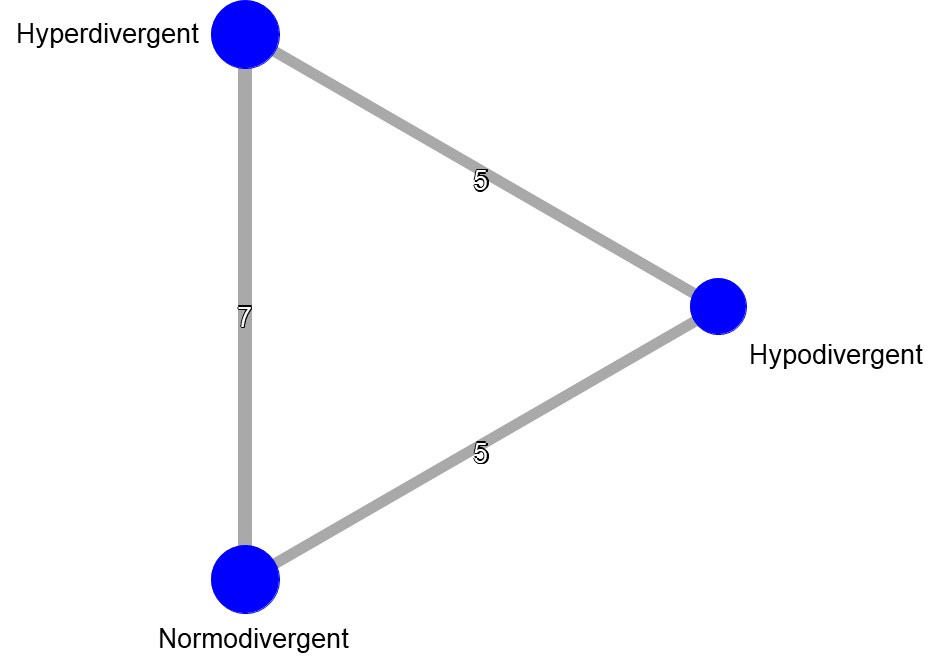


.

**Supplementary Figure 10.** Network graph for the effect of vertical discrepancies on the volume of the oro & hypopharynx.


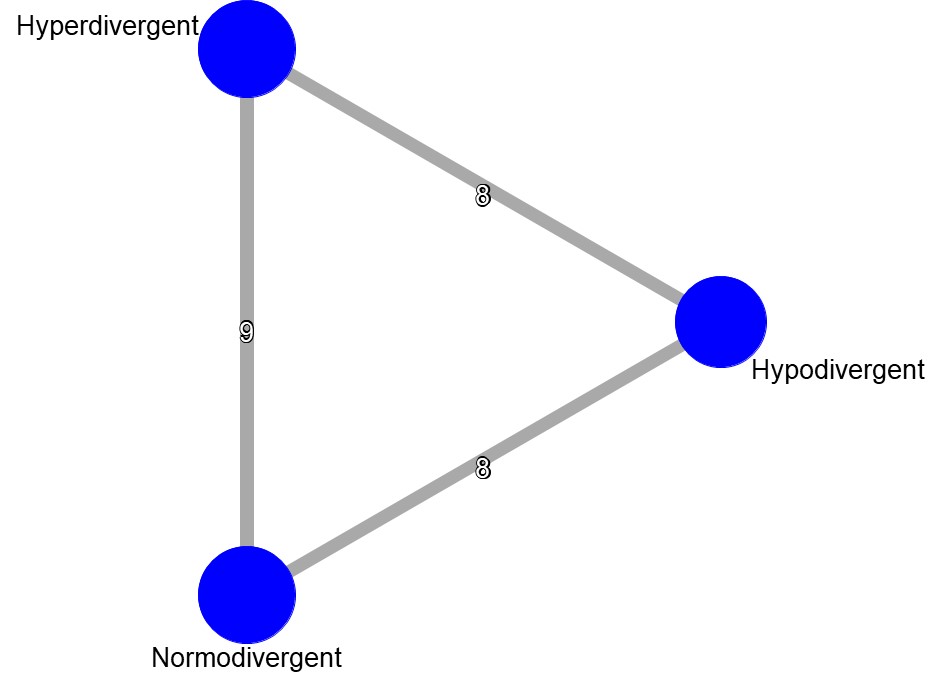


.

**Supplementary Figure 11.** Boxplots assessing the transitivity assumption based on mean age for each comparison of each network for sagittal effects.

.

**Supplementary Figure 12.** Boxplots assessing the transitivity assumption based on mean age for each comparison of each network for vertical effects.

.

**Supplementary Figure 13.** Boxplots assessing the transitivity assumption based on % of male participants in the study sample for each comparison of each network for sagittal effects.

.

**Supplementary Figure 14.** Boxplots assessing the transitivity assumption based on % of male participants in the study sample for each comparison of each network for sagittal effects.

.

**Supplementary Figure 15.** Comparison-adjusted funnel plot for sagittal patterns and the primary outcome of total pharynx volume.

.

**Supplementary Figure 16.** Comparison-adjusted funnel plot for sagittal patterns and the outcome of nasopharynx volume.

.

**Supplementary Figure 17.** Comparison-adjusted funnel plot for sagittal patterns and the outcome of palatopharynx volume.

.

**Supplementary Figure 18.** Comparison-adjusted funnel plot for sagittal patterns and the outcome of glossopharynx volume.

.

**Supplementary Figure 19.** Comparison-adjusted funnel plot for sagittal patterns and the outcome of oropharynx volume.

.

**Supplementary Figure 20.** Comparison-adjusted funnel plot for sagittal patterns and the outcome of oro- & hypopharynx volume.

.

**Supplementary Figure 21.** Comparison-adjusted funnel plot for vertical patterns and the outcome of nasopharynx volume.

.
