# Supplementary material for: Do patients with different craniofacial patterns have differences in upper airway volume? A systematic review with network meta-analysis
Source: Eur J Orthod. 2024 Mar 25;46(2):cjae010. doi: 10.1093/ejo/cjae010 (PMC10962399; doi:10.1093/ejo/cjae010)
Supplement: cjae010_suppl_Supplementary_Tables_S5 [file cjae010_suppl_supplementary_tables_s5.docx]

| **Supplementary Table 5. Full list of deduplicated studies excluded by title, abstract, full text with reasons.** | | |
| --- | --- | --- |
|  | **Title** | **Excluded by** |
|  | 1st Australasian Conference on Computational Mechanics, ACCM 2013, in Applied Mechanics and Materials. 2014. | Title |
|  | 40th International Conference on Environmental Systems. in 40th International Conference on Environmental Systems, ICES2010. | Title |
|  | 53rd AIAA/ASME/ASCE/AHS/ASC Structures, Structural Dynamics and Materials Conference. in 53rd AIAA/ASME/ASCE/AHS/ASC Structures, Structural Dynamics and Materials Conference 2012. 2012. | Title |
|  | 5th International Conference on Medical Image Computing and Computer-Assisted Intervention, MICCAI 2002, in Lecture Notes in Computer Science (including subseries Lecture Notes in Artificial Intelligence and Lecture Notes in Bioinformatics). 2002. p. 1-692. | Title |
|  | Abadi M, Pour OB. Genioplasty. Facial Plastic Surgery. 2015;31(5):513-22. | Title |
|  | Abate A, Gaffuri F, Lanteri V, Fama A, Ugolini A, Mannina L, et al. A CBCT based analysis of the correlation between volumetric morphology of the frontal sinuses and the facial growth pattern in caucasian subjects. A cross-sectional study. Head & Face Medicine. 2022;18(1). | Full text  Anatomic area outside scope of orthodontics |
|  | Abbas, H., Y. Hicks, and D. Marshall. Automatic classification of facial morphology for medical applications. in Procedia Computer Science. 2015. | Title |
|  | Abdalla Y, Brown L, Sonnesen L. Effects of a fixed functional appliance on upper airway volume: A 3-dimensional cone-beam computed tomography study. Am J Orthod Dentofacial Orthop. 2020;158(1):40-9. | Title |
|  | Abdalla Y, Brown L, Sonnesen L. Effects of rapid maxillary expansion on upper airway volume: A three-dimensional cone-beam computed tomography study. Angle Orthod. 2019;89(6):917-23. | Title |
|  | Abdalla Y, Kiliaridis S, Sonnesen L. Airway changes after fixed functional appliance treatment in children with and without morphologic deviations of the upper spine: A 3-dimensional CBCT study. Am J Orthod Dentofacial Orthop. 2022;161(6):791-7. | Title |
|  | Abdalla Y, Kiliaridis S, Sonnesen L. Dentofacial changes following treatment with a fixed functional appliance and their three-dimensional effects on the upper airway. Australasian Orthodontic Journal. 2021;37(2):284-93. | Title |
|  | **Abdelkarim, A., A cone beam CT evaluation of oropharyngeal airway space and its relationship to mandibular position and dentocraniofacial morphology. Journal of the World Federation of Orthodontists, 2012. 1(2): p. e55-e59.** | **Included** |
|  | Abeleira MT, Limeres J, Outumuro M, Vidal PS, Diniz-Freitas M, Ruiz-Pinon M, et al. Benefits of Maxillary Expansion for a Patient With Spinal Muscular Atrophy Type 2. American Journal of Physical Medicine & Rehabilitation. 2019;98(4):E32-E4. | Title |
|  | Aboudara C, Nielsen I, Huang JC, Maki K, Miller AJ, Hatcher D. Comparison of airway space with conventional lateral headfilms and 3-dimensional reconstruction from cone-beam computed tomography. Am J Orthod Dentofacial Orthop. 2009;135(4):468-79. | Title |
|  | Aboudara CA, Hatcher D, Nielsen IL, Miller A. A three-dimensional evaluation of the upper airway in adolescents. Orthod Craniofac Res. 2003;6 Suppl 1:173-5. | Full text  Other outcomes |
|  | Abrahao AR, Ribeiro BNF, Abrahao M, De Freitas Busnardo F, Goldenberg D. Resection and reconstruction of an arteriovenous malformation in the maxillary region. Otolaryngology - Head and Neck Surgery (United States). 2017;157(1):P186. | Title |
|  | Abramson Z, Susarla S, August M, Troulis M, Kaban L. Three-Dimensional Computed Tomographic Analysis of Airway Anatomy in Patients With Obstructive Sleep Apnea. Journal of Oral and Maxillofacial Surgery. 2010;68(2):354-62. | Title |
|  | Abramson Z, Susarla SM, Lawler M, Bouchard C, Troulis M, Kaban LB. Three-dimensional computed tomographic airway analysis of patients with obstructive sleep apnea treated by maxillomandibular advancement. Journal of Oral and Maxillofacial Surgery. 2011;69(3):677-86. | Title |
|  | Abramson ZR, Peacock ZS, Cohen HL, Choudhri AF. Radiology of Cleft Lip and Palate: Imaging for the Prenatal Period and throughout Life. Radiographics. 2015;35(7):2053-63. | Title |
|  | Abramson ZR, Susarla S, Tagoni JR, Kaban L. Three-dimensional computed tomographic analysis of airway anatomy. J Oral Maxillofac Surg. 2010;68(2):363-71. | Abstract |
|  | Abramson ZR, Susarla SM, Lawler ME, Peacock ZS, Troulis MJ, Kaban LB. Effects of mandibular distraction osteogenesis on three-dimensional airway anatomy in children with congenital micrognathia. Journal of Oral and Maxillofacial Surgery. 2013;71(1):90-7. | Title |
|  | Abramyan J, Thivichon-Prince B, Richman JM. Diversity in primary palate ontogeny of amniotes revealed with 3D imaging. Journal of Anatomy. 2015;226(5):420-33. | Title |
|  | Abuanza, M., et al., Spontaneous Intravesical Knotting of a Feeding Tube in a Late Preterm Neonate: A Case Report. Oman Medical Journal, 2022. 37(2). | Title |
|  | Abuhijleh E, Aydemir H, Toygar-Memikoglu U. Three-dimensional craniofacial morphology in unilateral cleft lip and palate. Journal of Oral Science. 2014;56(2):165-72. | Title |
|  | **Aby, D.M., et al., Comparison of airway morphology and volume in skeletal class i and class ii patients using cone-beam computed tomography: A cross-sectional study. World Journal of Dentistry, 2020. 11(5): p. 380-385.** | **Included** |
|  | Acar YB, Yöndem C, Erverdi AN, Bakkalbaşi B. Evaluation of Hyoid Position and Retroglossal Airway After Mandibular Alveolar Archwise Distraction Osteogenesis. J Craniofac Surg. 2020;31(8):2144-7. | Title |
|  | Acevedo AM, Lagravere-Vich M, Al-Jewair T. Diagnostic accuracy of lateral cephalograms and cone-beam computed tomography for the assessment of sella turcica bridging. American Journal of Orthodontics and Dentofacial Orthopedics. 2021;160(2):231-9. | Title |
|  | Actrn. A multi-center, prospective, randomised controlled trial comparing the efficacy and safety of PRODISC-C implant to anterior cervical discectomy and fusion (ACDF) surgery, in the treatment of symptomatic cervical disc disease (SCDD). https://trialsearchwhoint/Trial2aspx?TrialID=ACTRN12611000646943. 2011. | Title |
|  | Actrn. Intensive Early Adaptive Therapy (I-EAT) to Improve Feeding and Swallowing: A Clinical Trial for Infants at Risk of Cerebral Palsy. https://trialsearchwhoint/Trial2aspx?TrialID=ACTRN12618000305224. 2018. | Title |
|  | Adalı, E., et al., Does Concentrated Growth Factor Used With Allografts in Maxillary Sinus Lifting Have Adjunctive Benefits? Journal of Oral and Maxillofacial Surgery, 2021. 79(1): p. 98-108. | Title |
|  | Adam JC, Mauchaufee JC, Potard G, L'Azou D. Accidental intracerebral penetration of a nasal hemostatic probe. Ann Fr Anesth Reanim. 1999;18(4):436-9. | Title |
|  | Adam RJ, Abou Alaiwa MH, Bouzek DC, Cook DP, Gansemer ND, Taft PJ, et al. Postnatal airway growth in cystic fibrosis piglets. Journal of Applied Physiology. 2017;123(3):526-33. | Title |
|  | Adam, J.C., et al., Inadvertent intracerebral insertion of a nasal haemostatic probe. Annales Francaises d'Anesthesie et de Reanimation, 1999. 18(4): p. 436-439. | Title |
|  | Adam, R.J., et al., Postnatal airway growth in cystic fibrosis piglets. Journal of Applied Physiology, 2017. 123(3): p. 526-533. | Title |
|  | Adamczak SK, Pabst DA, McLellan WA, Thorne LH. Do bigger bodies require bigger radiators? Insights into thermal ecology from closely related marine mammal species and implications for ecogeographic rules. Journal of Biogeography. 2020;47(5):1193-206. | Title |
|  | Adriani, W., et al., 1H MRS-detectable metabolic brain changes and reduced impulsive behavior in adult rats exposed to methylphenidate during adolescence. Neurotoxicology and Teratology, 2007. 29(1): p. 116-125. | Title |
|  | Afshari, A., et al., Outcome measures (proms) of implant-supported, relative to tooth-supported fixed dental prostheses: A systematic review and meta-analysis. International Journal of Pharmaceutical Research, 2020. 12(3): p. 1358-1364. | Title |
|  | Afzali P, Joy R, Smith J, Conley S, Hummon G, Hatcher D, et al. Age contributions to airway dimension changes in females. International Journal of Oral and Maxillofacial Surgery. 2017;46:60. | Abstract |
|  | Agacayak KS, Gulsun B, Koparal M, Atalay Y, Aksoy O, Adiguzel O. Alterations in maxillary sinus volume among oral and nasal breathers. Medical Science Monitor. 2015;21:18-26. | Abstract |
|  | Agarwal R, Chandra R. Alar Web in Cleft Lip Nose Deformity: Study in Adult Unilateral Clefts. Journal of Craniofacial Surgery. 2012;23(5):1349-54. | Title |
|  | Agarwal R, Parihar A, Mandhani PA, Chandra R. Three-Dimensional Computed Tomographic Analysis of the Maxilla in Unilateral Cleft Lip and Palate: Implications for Rhinoplasty. Journal of Craniofacial Surgery. 2012;23(5):1338-42. | Title |
|  | Agarwal SS, Datana S, Roy ID, Andhare P. Effectiveness of Titratable Oral Appliance in Management of Moderate to Severe Obstructive Sleep Apnea-A Prospective Clinical Study with Acoustic Pharyngometry. Indian Journal of Otolaryngology and Head & Neck Surgery. | Title |
|  | Agbolade O, Nazri A, Yaakob R, Ghani AA, Cheah YK. Morphometric approach to 3D soft-tissue craniofacial analysis and classification of ethnicity, sex, and age. PLoS ONE. 2020;15(4). | Abstract |
|  | Agrawal A, Chaturvedi TP, Raghav M. Oropharyngeal Airway: The Orthodontist Perspective. Int J Orthod Milwaukee. 2016;27(3):51-6. | Abstract |
|  | Ah Rhee CJ, Castaneda C, Abe O, Woo JH. SEVERE SYMPTOMATIC HYPONATREMIA FROM INTENTIONAL WATER INTOXICATION: A DANGEROUS WEIGHT LOSS FAD EMERGING IN KOREAN CULTURE. Chest. 2019;156(4):A2013-A4. | Title |
|  | Ahn HW, Lee BS, Kim SW, Kim SJ. Stability of Modified Maxillomandibular Advancement Surgery in a Patient With Preadolescent Refractory Obstructive Sleep Apnea. J Oral Maxillofac Surg. 2015;73(9):1827-41. | Title |
|  | Ahn SH, Ha JG, Kim JW, Lee YW, Yoon JH, Kim CH, et al. Torus mandibularis affects the severity and position-dependent sleep apnoea in non-obese patients. Clinical Otolaryngology. 2019;44(3):279-85. | Title |
|  | Akarsu-Guven B, Karakaya J, Ozgur F, Aksu M. Growth-related changes of skeletal and upper-airway features in bilateral cleft lip and palate patients. American Journal of Orthodontics and Dentofacial Orthopedics. 2015;148(4):576-86. | Title |
|  | Akay G, Eren İ, Karadag Ö, Güngör K. Nasal septal deviation in the unilateral cleft lip and palate deformities: a three-dimensional analysis. Oral Radiol. 2021;37(4):567-72. | Title |
|  | Akay MC, Aras I, Günbay T, Aras A. Does transpalatal distraction affect pharyngeal airway dimensions and related soft tissues? Journal of Oral and Maxillofacial Surgery. 2014;72(8):1559-64. | Title |
|  | Akbay E, Cokkeser Y, Yilmaz O, Cevik C. The relationship between posterior septum deviation and depth of maxillopalatal arch. Auris Nasus Larynx. 2013;40(3):286-90. | Title |
|  | Akcam MO, Toygar TU, Wada T. Longitudinal investigation of soft palate and nasopharyngeal airway relations in different rotation types. Angle Orthodontist. 2002;72(6):521-6. | Full text  2D lat. cephalometry |
|  | Akgüner M. Velopharyngeal anthropometric analysis with MRI in normal subjects. Annals of Plastic Surgery. 1999;43(2):142-7. | Abstract |
|  | Akiyama, T., et al., A Japanese case of β-ureidopropionase deficiency with dysmorphic features. Brain and Development, 2017. 39(1): p. 58-61. | Title |
|  | Aksoy, F., et al., Association of accessory sphenoid septa with variations in neighbouring structures. Journal of Laryngology and Otology, 2017. 131(1): p. 51-55. | Title |
|  | Aktaş F, Aktaş T, Özmen Z, Akan H, Aksöz T, Altunkaş A. Evaluation of morphological changes in pharynx with dynamic CT and MRI in snoring patients. Rev Med Chil. 2016;144(9):1125-33. | Full text  Other outcomes |
|  | Aktaş, F., et al., Evaluation of morphological changes in pharynx with dynamic CT and MRI in snoring patients. Revista Medica de Chile, 2016. 144(9): p. 1125-1133. | Title |
|  | Al Ali A, Richmond S, Popat H, Playle R, Pickles T, Zhurov AI, et al. The influence of snoring, mouth breathing and apnoea on facial morphology in late childhood: A three-dimensional study. BMJ Open. 2015;5(9). | Title |
|  | Al Ali, A., et al., The influence of asthma on face shape: A three-dimensional study. European Journal of Orthodontics, 2014. 36(4): p. 373-380. | Title |
|  | Al Amri MS, Sabban HM, Alsaggaf DH, Alsulaimani FF, Al-Turki GA, Al-Zahrani MS, et al. Anatomical consideration for optimal position of orthodontic miniscrews in the maxilla: A CBCT appraisal. Annals of Saudi Medicine. 2020;40(4):330-7. | Title |
|  | Al Ayoubi A, Dalla Torre D, Madlena M. Craniofacial characteristics of Syrian adolescents with Class II division 1 malocclusion: a retrospective study. Peerj. 2020;8. | Full text  2D lat. cephalometry |
|  | Al Khateeb KA, Fotouh MA, Abdelsayed F, Fahim F. Short-Term Efficacy of Presurgical Vacuum Formed Nasoalveolar Molding Aligners on Nose, Lip, and Maxillary Arch Morphology in Infants With Unilateral Cleft Lip and Palate: A Prospective Clinical Trial. Cleft Palate-Craniofacial Journal. 2021;58(7):815-23. | Title |
|  | Al Zayer M, Leung YY. Orthognathic and Aesthetic Surgery Skeletal and airway stability after mandibular setback in patients with mandibular prognathism: A systematic review. International Journal of Oral and Maxillofacial Surgery. 2017;46:152. | Title |
|  | Alagoz, E., et al., Evaluating the changes in nasal airway volume and nasal airflow after surgically assisted rapid maxillary expansion. Oral Surgery, Oral Medicine, Oral Pathology and Oral Radiology, 2022. | Title |
|  | Alam S, Li C, Bradburn KH, Zhao K, Lee TS. Impact of Middle Turbinectomy on Airflow to the Olfactory Cleft: A Computational Fluid Dynamics Study. American Journal of Rhinology and Allergy. 2019;33(3):263-8. | Title |
|  | Al-Anezi, T., et al., A new method for automatic tracking of facial landmarks in 3D motion captured images (4D). International Journal of Oral and Maxillofacial Surgery, 2013. 42(1): p. 9-18. | Title |
|  | Albanese, M., et al., Mandibular distraction of the body and ramus. Minerva stomatologica, 2006. 55(6): p. 327-353. | Title |
|  | Albawaneh, Z., R. Ali, and J. Abramyan, Novel insights into the development of the avian nasal cavity. Anatomical Record, 2021. 304(2): p. 247-257. | Title |
|  | Alcalde, L.F.A., et al., Computed tomography visualizing alterations in the upper airway after orthognathic surgery. Journal of Cranio-Maxillofacial Surgery, 2019. 47(7): p. 1041-1045. | Title |
|  | Alexander, T.H., et al., Insulin-like growth factor-I and growth differentiation factor-5 promote the formation of tissue-engineered human nasal septal cartilage. Tissue Engineering - Part C: Methods, 2010. 16(5): p. 1213-1221. | Title |
|  | Alghamdi, M.A., et al., Succinic semialdehyde dehydrogenase deficiency presenting with central hypothyroidism. Clinical Case Reports, 2021. 9(1): p. 229-235. | Title |
|  | **Alhammadi MS, Almashraqi AA, Halboub E, Almahdi S, Jali T, Atafi A, et al. Pharyngeal airway spaces in different skeletal malocclusions: a CBCT 3D assessment. Cranio. 2021;39(2):97-106.** | **Included** |
|  | Alhammadi MS, Elfeky HY, Fayed MS, Ishaq RAR, Halboub E, Al-Mashraqi AA. Three-dimensional skeletal and pharyngeal airway changes following therapy with functional appliances in growing skeletal Class II malocclusion patients : A controlled clinical trial. J Orofac Orthop. 2019;80(5):254-65. | Title |
|  | Alharethy S, Al-Quniabut I, Jang YJ. Anthropometry of Arabian nose using computed tomography scanning. Annals of Saudi Medicine. 2017;37(2):144-7. | Abstract |
|  | Al-Housami, S.A.D., et al., Three-dimensional soft tissue assessment following mandibular bilateral sagittal split osteotomy. Journal of Craniofacial Surgery, 2015. 26(8): p. e702-e706. | Title |
|  | Ali S, Ehrlich DE, Moreno Uribe LM, Amendt BA, Lee MK, Shaffer JR, et al. Linking the Expression of Facial Shape and BMI via the Hippo Signaling Pathway. FASEB Journal. 2019;33(SUPPL 1):10.3. | Title |
|  | Ali, A., M. Kurien, and K.G. Selvaraj, Correlation of the ethmoid infundibulum to the medial orbital wall in maxillary sinus hypoplasia: "Infundibular lateralization," a diagnostic CT finding. Ear, Nose and Throat Journal, 2007. 86(12): p. 744-747. | Title |
|  | Alisha, K.H., et al., A New Frame for Orienting Infants With Cleft Lip and Palate During 3-Dimensional Facial Scanning. Cleft Palate-Craniofacial Journal, 2022. 59(7): p. 946-950. | Title |
|  | Aljawad H, Lee KM, Lim HJ. Three-dimensional evaluation of upper airway changes following rapid maxillary expansion: A retrospective comparison with propensity score matched controls. PLoS ONE. 2021;16(12 December). | Title |
|  | Aljehani D. Review the impact of mandibular setback surgery for the correction of class iii malocclusion on the upper airway space. Open Access Macedonian Journal of Medical Sciences. 2021;9:644-9. | Title |
|  | Al-Jewair T, Kurtzner K, Giangreco T, Warunek S, Lagravere-Vich M. Effects of clear aligner therapy for Class II malocclusion on upper airway morphology and daytime sleepiness in adults: A case series. International Orthodontics. 2020;18(1):154-64. | Title |
|  | Alkali HY, Amoah CBD, Ibrahim M. Laryngeal papilomatosis presenting with acute upper airway obstruction. Indian Journal of Pediatrics. 2009;76(7):743-4. | Title |
|  | Alkhasov, A., et al., Surgical Treatment of Patients with Full Tracheal Rings: Our Experience. Journal of Laparoendoscopic and Advanced Surgical Techniques, 2021. 31(12): p. 1511-1515. | Title |
|  | Al-Khatib AR, Rajion ZA, Masudi SM, Hassan R, Anderson PJ, Townsend GC. Stereophotogrammetric analysis of nasolabial morphology among Asian Malays: Influence of age and sex. Cleft Palate-Craniofacial Journal. 2012;49(4):463-71. | Title |
|  | Al-Khatib AR, Rajion ZA, Masudi SM, Hassan R, Townsend GC. Dento-facial relationships in individuals with normal occlusion. Homo-Journal of Comparative Human Biology. 2013;64(4):296-311. | Title |
|  | Alkhayer, A., et al., Evaluation of the soft tissue changes after rapid maxillary expansion using a handheld three-dimensional scanner: A prospective study. International Journal of Environmental Research and Public Health, 2021. 18(7). | Title |
|  | Allam E, Mpofu P, Ghoneima A, Tuceryan M, Kula K. The Relationship Between Hard Tissue and Soft Tissue Dimensions of the Nose in Children: A 3D Cone Beam Computed Tomography Study. J Forensic Sci. 2018;63(6):1652-60. | Full text  Other outcomes |
|  | Allen LM, Silverman RK, Nosovitch JT, Lohnes TM, Williams KD. Exploring 3-dimensional imaging techniques in the prenatal interrogation of cebocephaly. Journal of Ultrasound in Medicine. 2008;27(6):983-8. | Title |
|  | Allensworth, J.J., K.D. O'Dell, and J.S. Schindler, Bilateral vocal fold paralysis and dysphagia secondary to diffuse idiopathic skeletal hyperostosis. Head and Neck, 2017. 39(1): p. E1-E3. | Title |
|  | Almukhtar A, Ayoub A, Khambay B, McDonald J, Ju X. State-of-the-art three-dimensional analysis of soft tissue changes following Le Fort I maxillary advancement. British Journal of Oral & Maxillofacial Surgery. 2016;54(7):812-7. | Title |
|  | Almukhtar A, Khambay B, Ju X, Ayoub A. Comprehensive analysis of soft tissue changes in response to orthognathic surgery: mandibular versus bimaxillary advancement. Int J Oral Maxillofac Surg. 2018;47(6):732-7. | Title |
|  | Almuslamani, A., F. Ali, and A. Ali, X-linked adrenoleukodystrophy: A novel mutation of ABCD1 gene in a bahraini boy. Kuwait Medical Journal, 2014. 46(4): p. 355-359. | Title |
|  | Almuzian M, Almukhtar A, Ju X, Al-Hiyali A, Benington P, Ayoub A. Effects of le Fort i Osteotomy on the Nasopharyngeal Airway - 6-Month Follow-Up. Journal of Oral and Maxillofacial Surgery. 2016;74(2):380-91. | Title |
|  | Almuzian M, Ju X, Almukhtar A, Ayoub A, Al-Muzian L, McDonald JP. Does rapid maxillary expansion affect nasopharyngeal airway? A prospective Cone Beam Computerised Tomography (CBCT) based study. Surgeon. 2018;16(1):1-11. | Title |
|  | Almuzian, M., et al., Effects of le Fort i Osteotomy on the Nasopharyngeal Airway - 6-Month Follow-Up. Journal of Oral and Maxillofacial Surgery, 2016. 74(2): p. 380-391. | Title |
|  | Almuzian, M., et al., Three-dimensional nasolabial changes after maxillary advancement osteotomy in class III individuals: a systematic review and meta-analysis. Evidence-Based Dentistry, 2021. | Title |
|  | Al-Omari I, Millett DT, Ayoub AF. Methods of assessment of cleft-related facial deformity: A review. Cleft Palate-Craniofacial Journal. 2005;42(2):145-56. | Title |
|  | Alonso N, Risso GH, Denadai R, Raposo-Amaral CE. Effect of maxillary alveolar reconstruction on nasal symmetry of cleft lip and palate patients: a study comparing iliac crest bone graft and recombinant human bone morphogenetic protein-2. Journal of plastic, reconstructive & aesthetic surgery. 2014;67(9):1201‐8. | Title |
|  | Alpagan Ozdemir S, Esenlik E. Three-dimensional soft-tissue evaluation in patients with cleft lip and palate. Medical Science Monitor. 2018;24:8608-20. | Title |
|  | Alperovich, M., Discussion: Primary Rhinoplasty Does Not Interfere with Nasal Growth: A Long-Term Three-Dimensional Morphometric Outcome Study in Patients with Unilateral Cleft. Plastic and Reconstructive Surgery, 2020. 145(5): p. 1237-1238. | Title |
|  | AlQahtani F, George JM, Bishawi K, Kuriadom ST. Comparison of oropharyngeal airway dimensional changes in patients with skeletal Class II and Class III malocclusions after orthognathic surgery and functional appliance treatment: A systematic review. Saudi Dental Journal. 2021;33(8):860-8. | Title |
|  | Al-Qawasmi R, Parsons S, Wetherill L. Heritability of the pharyngeal airway volume and dimensions as assessed from siblings with overt malocclusions. Int Orthod. 2019;17(4):660-6. | Full text  Other outcomes |
|  | Al-Qawasmi R, Swiderski B, Kaczynski R. Evaluating interactions of airway changes during growth with orthodontic treatment. International Orthodontics. 2020;18(3):461-7. | Full text  Other outcomes |
|  | Alrawi M, McDermott M, Orr D, Russell J. Nasal chondromesynchymal hamartoma presenting in an adolescent. Int J Pediatr Otorhinolaryngol. 2003;67(6):669-72. | Title |
|  | Al-Rudainy D, Ju X, Mehendale FV, Ayoub A. Longitudinal 3D Assessment of Facial Asymmetry in Unilateral Cleft Lip and Palate. Cleft Palate-Craniofacial Journal. 2019;56(4):495-501. | Title |
|  | Al-Rudainy, D., et al., Assessment of regional asymmetry of the face before and after surgical correction of unilateral cleft lip. Journal of Cranio-Maxillofacial Surgery, 2018. 46(6): p. 974-978. | Title |
|  | AlSaty G, Xiang J, Burns M, Eliliwi M, Palomo JM, Martin C, et al. Follow-up observation of patients with obstructive sleep apnea treated by maxillomandibular advancement. Am J Orthod Dentofacial Orthop. 2020;158(4):527-34. | Title |
|  | Alsufyani NA, Dietrich NH, Lagravère MO, Carey JP, Major PW. Cone beam computed tomography registration for 3-D airway analysis based on anatomic landmarks. Oral Surg Oral Med Oral Pathol Oral Radiol. 2014;118(3):371-83. | Title |
|  | Alsufyani, N.A., et al., Upper airway imaging in sleep-disordered breathing: role of cone-beam computed tomography. Oral Radiology, 2017. 33(3): p. 161-169. | Title |
|  | Altieri F, Cassetta M. Comparison of changes in skeletal, dentoalveolar, periodontal, and nasal structures after tooth-borne or bone-borne rapid maxillary expansion: A parallel cohort study. Am J Orthod Dentofacial Orthop. 2022;161(4):e336-e44. | Title |
|  | Altug-Atac AT, Bollatoglu H, Memikoglu UT. Facial soft tissue profile following bimaxillary orthognathic surgery. Angle Orthodontist. 2008;78(1):50-7. | Title |
|  | **Alves Jr M, Franzotti ES, Baratieri C, Nunes LKF, Nojima LI, Ruellas ACO. Evaluation of pharyngeal airway space amongst different skeletal patterns. International Journal of Oral and Maxillofacial Surgery. 2012;41(7):814-9.** | **Included** |
|  | Alves M, Baratieri C, Mattos CT, Brunetto D, Fontes RD, Santos JRL, et al. Is the airway volume being correctly analyzed? American Journal of Orthodontics and Dentofacial Orthopedics. 2012;141(5):657-61. | Abstract |
|  | **Alves PVM, Zhao L, O'Gara M, Patel PK, Bolognese AM. Three-dimensional cephalometric study of upper airway space in skeletal class II and III healthy patients. Journal of Craniofacial Surgery. 2008;19(6):1497-507.** | **Included** |
|  | AlZayer MA, Leung YY. Skeletal and airway stability after mandibular setback in patients with mandibular prognathism: A systematic review. Saudi Dental Journal. 2020;33(4):169-76. | Title |
|  | Amano, K., et al., Indian hedgehog in craniofacial neural crest cells links to skeletal malocclusion by regulating associated cartilage formation and gene expression. FASEB Journal, 2020. 34(5): p. 6791-6807. | Title |
|  | Amendola RL, Reinhardt JM, Zimmerman MB, Sato Y, Diggelmann HR, Kacmarynski DSF. Development of a Preliminary Pediatric Tracheal Growth Model From Magnetic Resonance Images. Laryngoscope. 2014;124(8):1947-51. | Title |
|  | Amendola, R.L., et al. Graph-based segmentation of the pediatric trachea in MR images to model growth. in Progress in Biomedical Optics and Imaging - Proceedings of SPIE. 2013. | Title |
|  | Ami MB, Weiner E, Perlitz Y, Shalev E. Ultrasound evaluation of the width of the fetal nose. Prenatal Diagnosis. 1998;18(10):1010-3. | Title |
|  | Aminabadi, N.A., et al., Does prenatal restraint stress change the craniofacial growth pattern of rat offspring? European Journal of Oral Sciences, 2016. 124(1): p. 17-25. | Title |
|  | Amini F, Borzabadi-Farahani A, Mashayekhi Z, Pousti M, Amirtouri M. Soft-tissue profile characteristics in children with beta thalassaemia major. Acta Odontologica Scandinavica. 2013;71(5):1071-6. | Title |
|  | Amini M, Heravi F, Zandi B, Eslami S, Mohajerzadeh M, Rohani M. The effect of mandibular advancement device on physiologic parameters and volumetric MRI in mild to moderate obstructive sleep apnea-a randomized controlled trial. Sleep medicine. 2017;40:e14‐e5. | Title |
|  | Amm CE, Beidas O, Chaudhry O, Isakson M. Secondary deformities of neonatal and infant mandibular distraction. Cleft Palate-Craniofacial Journal. 2017;54(3):e49. | Title |
|  | Amuk NG, Kurt G, Baysal A, Turker G. Changes in pharyngeal airway dimensions following incremental and maximum bite advancement during Herbst-rapid palatal expander appliance therapy in late adolescent and young adult patients: a randomized non-controlled prospective clinical study. European Journal of Orthodontics. 2019;41(3):322-30. | Title |
|  | An JH, Park SB, Choi YK, Lee SH, Kim KB, Kim YI. Cone-Beam Computed Tomography Evaluation of Pharyngeal Airway Space Changes After Bimaxillary Orthognathic Surgery in Patients With Class III Skeletal Deformities: A 6-Year Follow-Up Study. Journal of Oral and Maxillofacial Surgery. 2019;77(12):2534-44. | Title |
|  | An, Y., et al., Diced costal cartilage graft combined with muscle repositioning improves Cleft-Side Alar asymmetry in Asian secondary unilateral cleft rhinoplasty: A three-dimensional evaluation. Journal of Plastic, Reconstructive and Aesthetic Surgery, 2021. 74(9): p. 2265-2271. | Title |
|  | Anagnostara, A., et al., Evaluation of the anatomical and functional properties of deglutition with various kinetic high-speed MRI sequences. Journal of Magnetic Resonance Imaging, 2001. 14(2): p. 194-199. | Title |
|  | Anandarajah S, Abdalla Y, Dudhia R, Sonnesen L. Proposal of new upper airway margins in children assessed by CBCT. Dentomaxillofacial Radiology. 2015;44(7). | Title |
|  | **Anandarajah S, Dudhia R, Sandham A, Sonnesen L. Risk factors for small pharyngeal airway dimensions in preorthodontic children: A three-dimensional study. Angle Orthod. 2017;87(1):138-46.** | **Included** |
|  | Anas IY, Bamgbose BO, Nuhu S. A comparison between 2D and 3D methods of quantifying facial morphology. Heliyon. 2019;5(6). | Title |
|  | Anderson CW, Nishikawa KC, Keifer J. Distribution of hypoglossal motor neurons innervating the prehensile tongue of the African pig-nosed frog, Hemisus marmoratum. Neuroscience Letters. 1998;244(1):5-8. | Title |
|  | Ando E, Ogawa T, Shigeta Y, Hirai S, Ikawa T, Ishikawa C, et al. A case of obstructive sleep apnoea with anterior cervical osteophytes. Journal of Oral Rehabilitation. 2009;36(10):776-80. | Title |
|  | Angelopoulos, C., Cone Beam Tomographic Imaging Anatomy of the Maxillofacial Region. Dental Clinics of North America, 2008. 52(4): p. 731-752. | Title |
|  | Angelova, I., et al., Computed tomography in the diagnosis of fractures of the maxillofacial area. Rentgenologiya i Radiologiya, 2013. 52(4): p. 265-269. | Title |
|  | Anton-Pacheco JL, Cano I, Comas J, Galletti L, Polo L, Garcia A, et al. Management of congenital tracheal stenosis in infancy. European Journal of Cardio-Thoracic Surgery. 2006;29(6):991-5. | Title |
|  | Aoki J, Shinozuka K, Yamagata K, Nakamura R, Sato T, Ohtani S, et al. Cephalometric analysis of the pharyngeal airway space after maxillary advancement surgery. J Oral Sci. 2019;61(4):529-33. | Title |
|  | Aoki, Y., et al. Simulation of postoperative 3D facial morphology using physics-based head model. in International Archives of the Photogrammetry, Remote Sensing and Spatial Information Sciences - ISPRS Archives. 2000. | Title |
|  | Apostolopoulos K, Shahrour R, Elshebiny T, Valiathan M. Airway Volume and Maximum Constriction Area Location in Cleft Population. Cleft Palate-Craniofacial Journal. 2022;59(4 SUPPL):84-5. | Title |
|  | Aqilah, N., et al., Computational Fluid Dynamic Study of the Pharyngeal Airway Characteristics Before and After Mandibular Setback Surgery in Patients with Mandibular Prognathism. CFD Letters, 2022. 14(3): p. 68-78. | Title |
|  | Arai, Y., et al., Sphenoid sinus development in patients with acquired middle ear cholesteatoma. Auris Nasus Larynx, 2020. 47(3): p. 391-400. | Title |
|  | Aras A, Akay MC, Çukurova I, Günbay T, Işiksal E, Aras I. Dimensional Changes of the Nasal Cavity After Transpalatal Distraction Using Bone-Borne Distractor: An Acoustic Rhinometry and Computed Tomography Evaluation. Journal of Oral and Maxillofacial Surgery. 2010;68(7):1487-97. | Title |
|  | Aras I, Dogan S. Comparative Evaluation of the Pharyngeal Airways and Related Soft Tissues of Unilateral and Bilateral Cleft Lip and Palate Patients With the Noncleft Individuals. Cleft Palate-Craniofacial Journal. 2017;54(1):53-9. | Title |
|  | Aras I, Olmez S, Dogan S. Comparative evaluation of nasopharyngeal airways of unilateral cleft lip and palate patients using three-dimensional and two-dimensional methods. Cleft Palate-Craniofacial Journal. 2012;49(6):e75-e81. | Title |
|  | Araújo PM, Carvalho ACGS, Gonçalves Filho RT, Gondim RF, Mello MJR, Nogueira RLM. Retrospective evaluation of skeletal and airway stability of patients undergoing surgery maxillomandibular advancement. International Journal of Oral and Maxillofacial Surgery. 2017;46:317-8. | Title |
|  | Arbel G, Hershkovitz I, Gross MD. Strain distribution on the skull due to occlusal loading: an anthropological perspective. Homo-Journal of Comparative Human Biology. 2000;51(1):30-55. | Title |
|  | Arcuri C, Muzzi F, Santini F, Barlattani A, Giancotti A. Five Years of Experience Using Palatal Mini-Implants for Orthodontic Anchorage. Journal of Oral and Maxillofacial Surgery. 2007;65(12):2492-7. | Title |
|  | Arens R, McDonough JM, Corbin AM, Hernandez ME, Maislin G, Schwab RJ, et al. Linear dimensions of the upper airway structure during development: Assessment by magnetic resonance imaging. American Journal of Respiratory and Critical Care Medicine. 2002;165(1):117-22. | Abstract |
|  | Arisaka T, Yagi T, Chiba S, Tonogi M, Nakajima T, Ota F. Creation of sleep apnea severity prediction equation by maxillofacial CT in non-elderly Japanese men. Sleep. 2018;41:A187-A8. | Title |
|  | Armstrong KN, Kerry LJ. Modelling the prey detection performance of Rhinonicteris aurantia (Chiroptera: Hipposideridae) in different atmospheric conditions discounts the notional role of relative humidity in adaptive evolution. Journal of Theoretical Biology. 2011;278(1):44-54. | Title |
|  | Arnold M, De Vos C, Van Zyl A, Schubert P, Zühlke A, Morkel J, et al. Mandibular desmoid fibromatosis: Balancing aggression of care for a benign disease. Pediatric Blood and Cancer. 2015;62:S352-S3. | Title |
|  | Arnold, M., et al., Experimental study on the influence of model variations on the airway occlusion of an obstructive sleep apnea patient. Journal of Biomechanics, 2021. 123. | Title |
|  | Asaumi R, Sato I, Miwa Y, Imura K, Sunohara M, Kawai T, et al. Understanding the formation of maxillary sinus in Japanese human foetuses using cone beam CT. Surgical and Radiologic Anatomy. 2010;32(8):745-51. | Title |
|  | Asgharian B, Price O, Schroeter J, Rodriguez J, Bentley T. Design and characterization of aerosol drug exposure atmospheres to aid in the development of delivery devices. Annals of Biomedical Engineering. 2016;44(12):3743-5. | Title |
|  | Ashabranner S, Albright T, Han M, Miloro M, Antonini F. Does Variation in Anatomic Landmarks Affect Volumetric Changes in the Upper Airway Following Mandibular Setback Surgery? Journal of Oral and Maxillofacial Surgery. 2020;78(10):e81-e2. | Abstract |
|  | Aslan, G., et al., Unilateral inferior turbinate bone hypertrophy: Is it compensatory or congenital? American Journal of Rhinology and Allergy, 2013. 27(4): p. 255-259. | Title |
|  | Aslıer, N.G.Y., et al., The Relationships Between Craniofacial Structure and Frontal Sinus Morphology: Evaluation With Conventional Anthropometry and CT-Based Volumetry. Ear, Nose and Throat Journal, 2020. 99(10): p. 637-647. | Full text  Dry skulls sample |
|  | Assi, A.R., et al., Differences on craniocervical posture and facial morphology among two ethnic groups. Revue de Medecine Legale, 2021. 12(1): p. 10-21. | Title |
|  | Assis, C., et al. Microchannels quality depends on workpiece microstructure and milling parameters. in Proceedings of the 16th International Conference of the European Society for Precision Engineering and Nanotechnology, EUSPEN 2016. 2016. | Title |
|  | Astani SA, Yilmaz HN, Nevzatoglu S, Demirkaya AA, Acar ZA. Evaluation of Airway Volume in Cleft Lip and Palate Following Nasoalveolar Molding. Journal of Craniofacial Surgery. 2018;29(8):2143-7. | Title |
|  | Atakan, A., et al., Does le Fort i osteotomy have an influence on nasal cavity and septum deviation? Nigerian Journal of Clinical Practice, 2020. 23(2): p. 240-245. | Title |
|  | Atterholt J, Wedel MJ. A computed tomography-based survey of paramedullary diverticula in extant Aves. Anatomical Record-Advances in Integrative Anatomy and Evolutionary Biology. | Title |
|  | Attie, K.M., et al., A single ascending-dose study of muscle regulator ace-031 in healthy volunteers. Muscle and Nerve, 2013. 47(3): p. 416-423. | Title |
|  | Aung, S.C., F.C. Liam, and L.S. Teik, Three dimensional laser scan assessment of the Oriental nose with a new classification of Oriental nasal types. British Journal of Plastic Surgery, 2000. 53(2): p. 109-116. | Title |
|  | Avci S, Ergun T, Aydin E, Kansu L. Craniofacial Measures of Chronic Otitis Media Patients by 2D Reformatted CT. Clinical Anatomy. 2010;23(4):374-85. | Title |
|  | Avci S, Ergun T, Aydin E, Kansu L. Sex differences in adult craniofacial parameters. Surgical and Radiologic Anatomy. 2015;37(9):1069-78. | Abstract |
|  | Avci S, Lakadamyali H, Lakadamyali H, Aydin E, Tekindal MA. Relationships among retropalatal airway, pharyngeal length, and craniofacial structures determined by magnetic resonance imaging in patients with obstructive sleep apnea. Sleep and Breathing. 2019;23(1):103-15. | Title |
|  | Averill LW, Kecskemethy HH, Theroux MC, Mackenzie WG, Pizarro C, Bober MB, et al. Tracheal narrowing in children and adults with mucopolysaccharidosis type IVA: evaluation with computed tomography angiography. Pediatric Radiology. 2021;51(7):1202-13. | Title |
|  | Awuapara S, Linan C, Solis G, Meneses A, Lagravere M. Evaluation of the nasal septum and depth of palatal arch in different facial vertical patterns: A Cone-Beam Computed Tomography Study. International Orthodontics. 2021;19(2):228-34. | Abstract |
|  | Azevêdo MS, Machado AW, Barbosa Ida S, Esteves LS, Rocha V, Bittencourt MA. Evaluation of upper airways after bimaxillary orthognathic surgery in patients with skeletal Class III pattern using cone-beam computed tomography. Dental Press J Orthod. 2016;21(1):34-41. | Title |
|  | Baddam P, Thereza-Bussolaro C, Flores-Mir C, Graf D. Nasal cavity structural anomalies in children and adolescents at high risk of sleep-disordered breathing: An exploratory cone-beam computed tomography study. Am J Orthod Dentofacial Orthop. 2021;160(4):533-43.e2. | Title |
|  | Badiali G, Lunari O, Bevini M, Bortolani B, Cercenelli L, Lorenzetti M, et al. Existence of a neutral-impact maxillo-mandibular displacement on upper airways morphology. Journal of Personalized Medicine. 2021;11(3):1-13. | Title |
|  | Badr FF, Jadu FM, Nasir AM, Jan AM, Wali S, Mustafa M, et al. Comparison of Airway Measurements in Rheumatoid Arthritis and Non-rheumatoid Patients using Lateral Cephalometric Radiographs. Nigerian Journal of Clinical Practice. 2021;24(8):1200-5. | Title |
|  | Badreddine FR, Fujita RR, Alves F, Cappellette M. Rapid maxillary expansion in mouth breathers: a short-term skeletal and soft-tissue effect on the nose. Brazilian Journal of Otorhinolaryngology. 2018;84(2):196-205. | Title |
|  | Badreddine FR, Yamamoto LH, Besen A, Hoppe DPMR, Fujita RR, Cappellette Junior M. Three-dimensional image study of accelerated maxillary expansion in oral breathing kids. Brazilian Journal of Otorhinolaryngology. 2022. | Title |
|  | Bae Y, Kuehn DP, Sutton BP, Conway CA, Perry JL. Three-Dimensional Magnetic Resonance Imaging of Velopharyngeal Structures. Journal of Speech Language and Hearing Research. 2011;54(6):1538-45. | Title |
|  | Baǧci, G., et al., Terminal 14q deletion with unbalanced t(Y;14)(q12;q32) translocation. Clinical Dysmorphology, 2012. 21(1): p. 37-41. | Title |
|  | Bagher, Z., et al., Will tissue-engineering strategies bring new hope for the reconstruction of nasal septal cartilage? Current Stem Cell Research and Therapy, 2019. 15(2): p. 144-154. | Title |
|  | Bai P, Udupa JK, Tong Y, Xie S, Torigian DA. Body region localization in whole-body low-dose CT images of PET/CT scans using virtual landmarks. Medical physics. 2019. 46(3): 1286-1299. | Title |
|  | Baj A, Youssef DA, Monteverdi R, Bianchi B, Combi VA, Gianni AB. Reconstruction of partial maxillary defects with the double-barrel fibula free flap. Acta Otorhinolaryngologica Italica. 2010;30(6):299-302. | Title |
|  | Baka, Z.M., et al., Cone-beam computed tomography evaluation of dentoskeletal changes after asymmetric rapid maxillary expansion. American Journal of Orthodontics and Dentofacial Orthopedics, 2015. 147(1): p. 61-71. | Title |
|  | Bakker J, Kuperus J, Kuijf H, Oner C, De Jong P, Verlaan JJ. Morphological characteristics of diffuse idiopathic skeletal hyperostosis in the cervical spine on computed tomography images. Global Spine Journal. 2017;7(2):119S-20S. | Title |
|  | Bala AK, Campbell PM, Tadlock LP, Schneiderman ED, Buschang PH. Short-term skeletal and dentoalveolar effects of overexpansion: A pilot randomized controlled trial. Angle Orthodontist. 2022;92(1):55-63. | Title |
|  | Balacey, H., et al. A new processing sequence to assess airways using 3D CT-scan. in 2013 IEEE International Conference on Image Processing, ICIP 2013 - Proceedings. 2013. | Title |
|  | Balasar Ö, Balasar B, Doǧru Ş, Örs R. Achondroplasia in twin. Journal of Perinatal Medicine. 2019;47:eA519. | Title |
|  | Balasundaram A. Hypopharyngeal lipoma causing obstructive sleep apnea: Discovery on dental cone-beam CT. Ear Nose Throat J. 2013;92(3):E1-4. | Title |
|  | Ballah D, Zhu X, Edgar JC, Cahill AM. The role of C-Arm computed tomography in the treatment of venous malformations and prediction of local skin complications. Journal of Investigative Medicine. 2011;59(3):625. | Title |
|  | Ballanti F, Lione R, Baccetti T, Franchi L, Cozza P. Treatment and posttreatment skeletal effects of rapid maxillary expansion investigated with low-dose computed tomography in growing subjects. Am J Orthod Dentofacial Orthop. 2010;138(3):311-7. | Title |
|  | Bandeira AM, Oltramari-Navarro PV, de Lima Navarro R, de Castro Ferreira Conti AC, de Almeida MR, Fernandes KB. Three-dimensional upper-airway assessment in patients with bronchial asthma. Angle Orthod. 2014;84(2):254-9. | Title |
|  | Banerjee M, Uppuluri P, Zhao XR, Carlisle PL, Vipulanandan G, Villar CC, et al. Expression of UME6, a Key Regulator of Candida albicans Hyphal Development, Enhances Biofilm Formation via Hgc1-and Sun41-Dependent Mechanisms. Eukaryotic Cell. 2013;12(2):224-32. | Title |
|  | Banjo, A.O., Levator submandibuli muscle (Banjo muscle) a rare muscle in human beings; anatomy, morphogenesis, function and incidence. African journal of medicine and medical sciences, 2009. 38(1): p. 1-8. | Title |
|  | Bank JR, Wirawan CS, Ehrlich DE, Marazita ML, Weinberg SM, Miller SF. Analyzing Facial Asymmetry in Children as a Function of BMI. FASEB Journal. 2020;34(SUPPL 1). | Title |
|  | Baratieri C, Alves M, Jr., de Souza MM, de Souza Araújo MT, Maia LC. Does rapid maxillary expansion have long-term effects on airway dimensions and breathing? Am J Orthod Dentofacial Orthop. 2011;140(2):146-56. | Title |
|  | Baratieri Cda L, Alves M, Mattos CT, Lau GW, Nojima LI, de Souza MM. Transverse effects on the nasomaxillary complex one year after rapid maxillary expansion as the only intervention: a controlled study. Dental press journal of orthodontics. 2014;19(5):79‐87. | Title |
|  | Barbero, M., et al., Tridimensional upper airway assessment in male patients with OSA using oral advancement devices modifying their vertical dimension. Journal of Clinical Sleep Medicine, 2020. 6(10): p. 1721-1729. | Title |
|  | Barbon, S., et al., Platelet-rich fibrin scaffolds for cartilage and tendon regenerative medicine: From bench to bedside. International Journal of Molecular Sciences, 2019. 20(7). | Title |
|  | Bardsley, K., et al., Repair of bone defects in vivo using tissue engineered hypertrophic cartilage grafts produced from nasal chondrocytes. Biomaterials, 2017. 112: p. 313-323. | Title |
|  | Barlaz M, Shosted R, Fu MJ, Sutton B. Oropharygneal articulation of phonemic and phonetic nasalization in Brazilian Portuguese. Journal of Phonetics. 2018;71:81-97. | Title |
|  | Barrera JE, Pau CY, Forest VI, Holbrook AB, Popelka GR. Anatomic measures of upper airway structures in obstructive sleep apnea. World Journal of Otorhinolaryngology - Head and Neck Surgery. 2017;3(2):85-91. | Title |
|  | Barrera JE. Virtual surgical planning improves surgical outcome measures in obstructive sleep apnea surgery. Laryngoscope. 2014;124(5):1259-66. | Title |
|  | Barrero C, Vu G, Humphries L, Taylor J, Nah HD. Postoperative changes of the upper airway following mandibular distraction osteogenesis in pediatric hemifacial microsomia. Cleft Palate-Craniofacial Journal. 2021;58(4 SUPPL):133-4. | Title |
|  | Barsamian JG, Cobb LW, Bremer AM, Scheffer RB, Northup HM. Radiographic, clinical, and histopathologic evaluation with surgical treatment of Forestier's disease. Oral Surg Oral Med Oral Pathol. 1985;59(2):136-41. | Title |
|  | Basciftci, F.A., et al., Does the Timing and Method of Rapid Maxillary Expansion Have an Effect on the Changes in Nasal Dimensions? Angle Orthodontist, 2002. 72(2): p. 118-123. | Title |
|  | Bass K, Boc S, Hindle M, Dodson K, Longest W. High-Efficiency Nose-to-Lung Aerosol Delivery in an Infant: Development of a Validated Computational Fluid Dynamics Method. Journal of Aerosol Medicine and Pulmonary Drug Delivery. 2019;32(3):132-48. | Title |
|  | Bass K, Farkas D, Hassan A, Bonasera S, Hindle M, Longest PW. High-efficiency dry powder aerosol delivery to children: Review and application of new technologies. Journal of Aerosol Science. 2021;153. | Title |
|  | Bassil-Nassif N, Bouserhal J, Garcia R. Facial volumes and vertical facial type: a three dimensional comparative study. Orthod Fr. 2010;81(2):127-37. | Full text  Other outcomes |
|  | Bastir M, Godoy P, Rosas A. Common features of sexual dimorphism in the cranial airways of different human populations. American Journal of Physical Anthropology. 2011;146(3):414-22. | Full text  Other outcomes |
|  | Bastir M, Higuero A, Rios L, Martinez DG. Three-Dimensional Analysis of Sexual Dimorphism in Human Thoracic Vertebrae: Implications for the Respiratory System and Spine Morphology. American Journal of Physical Anthropology. 2014;155(4):513-21. | Title |
|  | Bastir M, Megía I, Torres-Tamayo N, García-Martínez D, Piqueras FM, Burgos M. Three-dimensional analysis of sexual dimorphism in the soft tissue morphology of the upper airways in a human population. American Journal of Physical Anthropology. 2020;171(1):65-75. | Full text  Other outcomes |
|  | Bastir M, Rosas A. Cranial airways and the integration between the inner and outer facial skeleton in humans. American Journal of Physical Anthropology. 2013;152(2):287-93. | Abstract |
|  | Bastir M, Rosas A. Cranial base topology and basic trends in the facial evolution of Homo. Journal of Human Evolution. 2016;91:26-35. | Title |
|  | Bastir M, Rosas A. Nasal form and function in Mid-pleistocene human facial evolution. A first approach. American Journal of Physical Anthropology. 2011;144:83. | Title |
|  | Bastir M, Sanz-Prieto D, Burgos M. Three-dimensional form and function of the nasal cavity and nasopharynx in humans and chimpanzees. Anatomical Record. 2022;305(8):1962-73. | Title |
|  | Bates AJ, Schuh A, McConnell K, Williams BM, Lanier JM, Loew W, et al. Combining computational fluid dynamics and 3D-cine mri to determine the relationship between upper airway motion and breathing effort in pediatric obstructive sleep apnea. American Journal of Respiratory and Critical Care Medicine. 2017;195. | Title |
|  | Bauer, F.X., et al., Automated detection of alveolar arches for nasoalveolar molding in cleft lip and palate treatment. Current Directions in Biomedical Engineering, 2016. 2(1): p. 701-705. | Title |
|  | Baumann, A. and K. Sinko, Importance of soft tissue for skeletal stability in maxillary advancement in patients with cleft lip and palate. Cleft Palate-Craniofacial Journal, 2003. 40(1): p. 65-70. | Title |
|  | Bäumler, M., M. Bigorre, and J.M. Faure, Radiological evaluation of the fetal face using three-dimensional ultrasound imaging. Reports in Medical Imaging, 2012. 5(1): p. 103-113. | Title |
|  | Baykul, T., M.A. Aydin, and Y. Findik, Surgical acrylic guide for distractor positioning. International Journal of Oral and Maxillofacial Surgery, 2014. 43(6): p. 739-741. | Title |
|  | Bazak R, Ibrahim A, Hussein W, Abdelnaby M, Elwany S. Extramucosal pyriplasty without stenting for management of pyriform aperture stenosis. European Archives of Oto-Rhino-Laryngology. 2018;275(6):1469-75. | Title |
|  | Bazan-Socha S, Buregwa-Czuma S, Jakiela B, Zareba L, Zawlik I, Myszka A, et al. Airway transcriptome profile determining increased reticular basement membrane thickness in asthma patients. Allergy: European Journal of Allergy and Clinical Immunology. 2021;76(SUPPL 110):108. | Title |
|  | Bazan-Socha S, Buregwa-Czuma S, Jakiela B, Zareba L, Zawlik I, Myszka A, et al. Reticular Basement Membrane Thickness Is Associated with Growth- and Fibrosis-Promoting Airway Transcriptome Profile-Study in Asthma Patients. Int J Mol Sci. 2021;22(3): 1-21. | Title |
|  | Bazargani F, Feldmann I, Bondemark L. Three-dimensional analysis of effects of rapid maxillary expansion on facial sutures and bones. Angle Orthod. 2013;83(6):1074-82. | Title |
|  | Bazargani F, Lund H, Magnuson A, Ludwig B. Skeletal and dentoalveolar effects using tooth-borne and tooth-bone-borne RME appliances: a randomized controlled trial with 1-year follow-up. Eur J Orthod. 2021;43(3):245-53. | Title |
|  | Becker OE, Avelar RL, Goelzer JG, Dolzan AD, Haas OL, De Oliveira RB. Pharyngeal Airway Changes in Class III Patients Treated With Double Jaw Orthognathic Surgery-Maxillary Advancement and Mandibular Setback. Journal of Oral and Maxillofacial Surgery. 2012;70(11):E639-E47. | Title |
|  | Becker, D.B., et al., Functional neuroanatomy of lexical processing in children with cleft lip and palate. Plastic and Reconstructive Surgery, 2008. 122(5): p. 1371-1381. | Title |
|  | Becret A, Vialet R, Chaumoitre K, Loundou A, Lesavre N, Michel F. Upper airway modifications in head extension during development. Anaesthesia Critical Care & Pain Medicine. 2017;36(5):285-90. | Abstract |
|  | Beena, S., et al., Usefulness of fetal autopsy in the diagnosis of blomstrand chondrodysplasia: a report of three cases. Journal of Maternal-Fetal and Neonatal Medicine, 2017. 30(9): p. 1041-1044. | Title |
|  | Begnoni G, Serrao G, Musto F, Pellegrini G, Triulzi FM, Dellavia C. Craniofacial structures' development in prenatal period: An MRI study. Orthodontics & Craniofacial Research. 2018;21(2):96-103. | Title |
|  | Beidas, O.E., D.M. Thompson, and C.A. El Amm, Anthropometric effect of mucoperiosteal nostril floor reconstruction in complete cleft lip. Journal of Craniofacial Surgery, 2016. 27(1): p. 19-26. | Title |
|  | Beiriger J, Pfaff M, Owoc M, Mittal A, Erpenbeck S, Bruce M, et al. The Impact of Neonatal Mandibular Distraction on Long-Term Mandibular Growth and Morphology. Cleft Palate-Craniofacial Journal. 2022;59(4 SUPPL):46. | Title |
|  | Belfor TR. Epigenetic orthodontics: facial and airway development. N Y State Dent J. 2010;76(6):18-21. | Abstract |
|  | Bell RB, Osborn T, Dierks EJ, Potter BE, Long WB. Management of penetrating neck injuries: A new paradigm for civilian trauma. Journal of Oral and Maxillofacial Surgery. 2007;65(4):691-705. | Title |
|  | Benali, R., et al., Tubule formation by human surface respiratory epithelial cells cultured in a three-dimensional collagen lattice. American Journal of Physiology - Lung Cellular and Molecular Physiology, 1993. 264(2 8-2): p. L183-L192. | Title |
|  | Bender CA, Veneman W, Veenland JF, Mathijssen IM, Hop WC, Koudstaal MJ, et al. Orbital aspects following monobloc advancement in syndromic craniosynostosis. J Craniomaxillofac Surg. 2013;41(7):e146-53. | Title |
|  | Bendrihem, R., et al., Anatomic basis of Le Fort 1 impaction osteotomy: a radiological study. Surgical and Radiologic Anatomy, 2017. 39(11): p. 1209-1214. | Title |
|  | Bendtsen TF. Image fusion of ultrasound and MRI. Regional Anesthesia and Pain Medicine. 2013;38(5):E78-E9. | Title |
|  | Benevenuto SG, Domenico MD, Martins MAG, Costa NS, de Souza ARL, Costa JL, et al. Recreational use of marijuana during pregnancy and negative gestational and fetal outcomes: An experimental study in mice. Toxicology. 2017;376:94-101. | Title |
|  | Benninger MS, Hseu A. Laser surgical management of bilateral vocal fold immobility. Operative Techniques in Otolaryngology - Head and Neck Surgery. 2011;22(2):116-21. | Title |
|  | Bennun RD, Perandones C, Sepliarsky VA, Chantiri SN, Aguirre MIU, Dogliotti PL. Nonsurgical correction of nasal deformity in unilateral complete cleft lip: A 6-year follow-up. Plastic and Reconstructive Surgery. 1999;104(3):616-30. | Title |
|  | Berg C, Wessendorf TE, Mortsch F, Forsting M, Teschler H, Weischer T, et al. Influence of disease control with pegvisomant on sleep apnoea and tongue volume in patients with active acromegaly. European Journal of Endocrinology. 2009;161(6):829-35. | Title |
|  | Berg, C., et al., Prenatal sonographic features of Harlequin ichthyosis. Archives of Gynecology and Obstetrics, 2003. 268(1): p. 48-51. | Title |
|  | Bergmann C. 3D imaging in ENT diagnostics-experience report and update of the standard. Laryngo- Rhino- Otologie. 2018;97:S203. | Title |
|  | Berlin, F., et al., Mast cell proteases tryptase and chymase induce migratory and morphological alterations in bronchial epithelial cells. International Journal of Molecular Sciences, 2021. 22(10). | Title |
|  | Bernichi JV, Rizzo VL, Villa JF, Santos RF, Caparroz FA. Rhinogenic and sinus headache - Literature review. Am J Otolaryngol. 2021;42(6):103113. | Title |
|  | Berthommier, F., et al., Comparative anatomy of the baboon and human vocal tracts: Renewal of methods, data, and hypotheses, in Origins of Human Language: Continuities and Discontinuities with Nonhuman Primates. 2018. p. 106-144. | Title |
|  | Bertoz A, Marão AC, Barros J, Weber SA. 3D evaluation of the pharyngeal dimensions in OSA children, before and after adenotonsillectomy. European Respiratory Journal. 2015;46. | Title |
|  | Bertoz APD, Souki BQ, Lions R, Webber SAT, Bigliazzi R, Oliveira PM, et al. Three-dimensional airway changes after adenotonsillectomy in children with obstructive apnea: Do expectations meet reality? American Journal of Orthodontics and Dentofacial Orthopedics. 2019;155(6):791-800. | Title |
|  | Besio, R., et al., Lack of prolidase causes a bone phenotype both in human and in mouse. Bone, 2015. 72: p. 53-64. | Title |
|  | Bharadwaj, R., A. Ravikumar, and N.R. Krishnaswamy, Evaluation of craniofacial morphology in patients with obstructive sleep apnea using lateral cephalometry and dynamic MRI. Indian Journal of Dental Research, 2011. 22(6): p. 739-748. | Title |
|  | Bhattacharjee, A., A. Chakraborty, and P. Purkaystha, Frontoethmoidal encephalomeningocoele with colpocephaly: Case report and clinical review. Journal of Laryngology and Otology, 2008. 122(3): p. 321-323. | Title |
|  | Bhave SY. Approach to recurrent respiratory infections. Indian J Pediatr. 2001;68 Suppl 2:S26-32. | Title |
|  | Bi RY, Luo XT, Jiang N, Zhu SS, Li YF. Change in the posterior airway after mandibular distraction osteogenesis in patients with ankylosis of the temporomandibular joint: a retrospective study. British Journal of Oral & Maxillofacial Surgery. 2018;56(6):525-30. | Title |
|  | Bianchi FA, Gerbino G, Corsico M, Schellino E, Barla N, Verzè L, et al. Soft, hard-tissues and pharyngeal airway volume changes following maxillomandibular transverse osteodistraction: Computed tomography and three-dimensional laser scanner evaluation. Journal of Cranio-Maxillofacial Surgery. 2017;45(1):47-55. | Title |
|  | Bianco AS, Porras-De Agostini G, Garcia E, Sanchez M, Agostini G. Liposarcoma of nasopharynx. Otolaryngology - Head and Neck Surgery (United States). 2012;147:P165. | Title |
|  | Bielohuby M, Matsuura M, Herbach N, Kienzle E, Slawik M, Hoeflich A, et al. Short-Term Exposure to Low-Carbohydrate, High-Fat Diets Induces Low Bone Mineral Density and Reduces Bone Formation in Rats. Journal of Bone and Mineral Research. 2010;25(2):275-84. | Title |
|  | Bienstock D, Ayyala R, Eisig SB, Perrino MA. Evaluation of the Pierre robin sequence mandible using computed tomography. Journal of Oral and Maxillofacial Surgery. 2016;74(9):e52-e3. | Title |
|  | Bigoni L, Veleminska J, Bruzek J. Three-dimensional geometric morphometric analysis of cranio-facial sexual dimorphism in a Central European sample of known sex. Homo-Journal of Comparative Human Biology. 2010;61(1):16-32. | Title |
|  | Bila M, Defrancq J, Nadjmi N, Renier L, Stevens S, Vanassche B, et al. CBCT analysis of skeletal, dental and nasal changes after transpalatal distraction. International Journal of Oral and Maxillofacial Surgery. 2015;44:e39. | Title |
|  | Bilger H, Hood S, Bader K, Clarke JA. Guidelines for removal, preservation, and CT imaging of the syrinx, the avian vocal organ. Wilson Journal of Ornithology. 2020;132(3):628-38. | Title |
|  | Bilgiç F, Damlar İ, Sürmelioğlu Ö, Sözer Ö A, Tatlı U. Relationship between voice function and skeletal effects of rapid maxillary expansion. Angle Orthod. 2018;88(2):202-7. | Title |
|  | Billet G, Hautier L, de Thoisy B, Delsuc F. The hidden anatomy of paranasal sinuses reveals biogeographically distinct morphotypes in the nine-banded armadillo (Dasypus novemcinctus). Peerj. 2017;5. | Title |
|  | Bilwatsch S, Kramer M, Haeusler G, Schuster M, Wurm J, Vairaktaris E, et al. Nasolabial symmetry following Tennison-Randall lip repair: A three-dimensional approach in 10-year-old patients with unilateral clefts of lip, alveolus and palate. Journal of Cranio-Maxillofacial Surgery. 2006;34(5):253-62. | Title |
|  | Bin F, Meng Y, Meng J, Hu W. Comparison of velum morphologies using cephalometry and dental CBCT. Oral Radiology. 2016;32(1):1-8. | Title |
|  | Bin, L.R., et al., How does bimaxillary orthognathic surgery change dimensions of maxillary sinuses and pharyngeal airway space? Angle Orthodontist, 2020. 90(5): p. 715-722. | Title |
|  | Bitners AC, Sin S, Silver EJ, Shifteh K, Arens R. Lower face characteristics by linear MRI measurements in obese children with obstructive sleep apnea (OSA): Effect of age and sex. American Journal of Respiratory and Critical Care Medicine. 2020;201(1). | Title |
|  | Bjørndal, L., et al., Maxillary Sinus Impaction of a Core Carrier Causing Sustained Apical Periodontitis, Sinusitis, and Nasal Stenosis: A 3-year Follow-up. Journal of Endodontics, 2016. 42(12): p. 1851-1858. | Title |
|  | Blair A, Staub E, Dahl MJ, Yoder BA, Null DM, Albertine K. Plasma renin concentration is differentially affected by ventilation mode in preterm lambs. Journal of Investigative Medicine. 2015;63(1):213. | Title |
|  | Blanc, P., et al., A role for mesenchyme dynamics in mouse lung branching morphogenesis. PLoS ONE, 2012. 7(7). | Title |
|  | Blanco, M., et al., Hyperinsulinism in a neonate. Pediatric Annals, 2014. 43(3): p. e56-e60. | Title |
|  | Bleier, B.S. and R.J. Schlosser, Prevention and management of medial rectus injury. Otolaryngologic Clinics of North America, 2010. 43(4): p. 801-807. | Title |
|  | Block, M.S., Maxillary fixed prosthesis design Based on the preoperative physical examination. Journal of Oral and Maxillofacial Surgery, 2015. 73(5): p. 851-860. | Title |
|  | Blomström-Lundqvist, C., et al., European Heart Rhythm Association (EHRA) international consensus document on how to prevent, diagnose, and treat cardiac implantable electronic device infections-endorsed by the Heart Rhythm Society (HRS), the Asia Pacific Heart Rhythm Society (APHRS), the Latin American Heart Rhythm Society (LAHRS), International Society for Cardiovascular Infectious Diseases (ISCVID), and the European Society of Clinical Microbiology and Infectious Diseases (ESCMID) in collaboration with the European Association for Cardio-Thoracic Surgery (EACTS). European Heart Journal, 2020. 41(21): p. 2012-2032. | Title |
|  | Blomström-Lundqvist, C., et al., European Heart Rhythm Association (EHRA) international consensus document on how to prevent, diagnose, and treat cardiac implantable electronic device infections - Endorsed by the Heart Rhythm Society (HRS), the Asia Pacific Heart Rhythm Society (APHRS), the Latin American Heart Rhythm Society (LAHRS), International Society for Cardiovascular Infectious Diseases (ISCVID) and the European Society of Clinical Microbiology and Infectious Diseases (ESCMID) in collaboration with the European Association for Cardio-Thoracic Surgery (EACTS). Europace, 2020. 22(4): p. 515-516. | Title |
|  | BMEiCON 2016 - 9th Biomedical Engineering International Conference. in BMEiCON 2016 - 9th Biomedical Engineering International Conference. 2017. | Title |
|  | Boecking C, Walentek P, Zlock L, Sun DI, Wolters P, Ishikawa H, et al. A simple method to generate human bronchial organoids: Characterization and comparison with air-liquid interface cultures. Pediatric Pulmonology. 2020;55(SUPPL 2):196. | Title |
|  | Boecking C, Zlock L, Ishikawa H, Marshall W, Finkbeiner WE. Three-dimensional organoid cultures from primary and conditionally reprogramed human bronchial epithelial cells. Pediatric Pulmonology. 2015;50:260-1. | Title |
|  | Boecking CA, Walentek P, Zlock LT, Sun DI, Wolters PJ, Ishikawa H, et al. A simple method to generate human airway epithelial organoids with externally orientated apical membranes. American Journal of Physiology - Lung Cellular and Molecular Physiology. 2022;322(3):L430-L7. | Title |
|  | Bohne WH. Tarsal coalition. Curr Opin Pediatr. 2001;13(1):29-35. | Title |
|  | Boland, F.X., et al., Gingivoperiosteoplasty associated to bone graft: Radiological evaluation. Revue de Stomatologie et de Chirurgie Maxillo-Faciale, 2009. 110(4): p. 193-197. | Title |
|  | Boninsegna E, Barillari M, De Rossi S, Marchioni D, Mansueto G. Non-traumatic pneumocephalus caused by increased depth of olfactory fossa. British Journal of Oral & Maxillofacial Surgery. 2019;57(6):587-9. | Title |
|  | Bonne NX, Zago S, Hosana G, Vinchon M, Van den Abbeele T, Fayoux P. Endonasal endoscopic approach for removal of intranasal nasal glial heterotopias. Rhinology. 2012;50(2):211-7. | Title |
|  | Bora P, Agrawal P, Bagga DK, Tiwari S, Singh A. Applications of Cone Beam Computed Tomography in Orthodontics: An Overview. Bioscience Biotechnology Research Communications. 2020;13(15):168-72. | Title |
|  | Bornstein, M.M., P. Pazera, and C. Katsaros, The use of three-dimensional reconstructions in the diagnosis of impacted teeth, in Informatics in Oral Medicine: Advanced Techniques in Clinical and Diagnostic Technologies. 2010. p. 171-183. | Title |
|  | Borsky J, Veleminska J, Jurovčík M, Kozak J, Hechtova D, Tvrdek M, et al. Successful early neonatal repair of cleft lip within first 8 days of life. International Journal of Pediatric Otorhinolaryngology. 2012;76(11):1616-26. | Title |
|  | Bos, A.C., et al., Patient-specific modeling of regional antibiotic concentration levels in airways of patients with cystic fibrosis: Are we dosing high enough? PLoS ONE, 2015. 10(3). | Title |
|  | Botti, S., et al., Vomero-premaxillary joint: A marker of evolution of the species. European Annals of Otorhinolaryngology, Head and Neck Diseases, 2017. 134(2): p. 83-87. | Title |
|  | Boughner JC, Buchtova M, Fu K, Diewert V, Hallgrimsson B, Richman JM. Embryonic development of Python sebae - I: Staging criteria and macroscopic skeletal morphogenesis of the head and limbs. Zoology. 2007;110(3):212-30. | Title |
|  | Boulvain M, Irion O, Thornton JG. Induction of labour at or near term for suspected fetal macrosomia. Cochrane Database of Systematic Reviews. 2016(5). | Title |
|  | Bourassa D, Gleber SC, Vogt S, Shin CH, Fahrni CJ. MicroXRF tomographic visualization of zinc and iron in the zebrafish embryo at the onset of the hatching period. Metallomics. 2016;8(10):1122-30. | Title |
|  | Bourassa, C., et al., In-vitro comparison of different palatal sites for orthodontic miniscrew insertion: Effect of bone quality and quantity on primary stability. American Journal of Orthodontics and Dentofacial Orthopedics, 2018. 154(6): p. 809-819. | Title |
|  | Bourke JE, Li X, Foster SR, Wee E, Dagher H, Ziogas J, et al. Collagen remodelling by airway smooth muscle is resistant to steroids and beta(2)-agonists. European Respiratory Journal. 2011;37(1):173-82. | Title |
|  | Bous R, Shah P, Elnaghy R, Valiathan M. Comparison of the pharyngeal airway volume between patients with ectodermal dysplasia and non-affected controls: A CBCT study. Cleft Palate-Craniofacial Journal. 2020;57(4):7. | Title |
|  | Bouserhal J, Bassil-Nassif N, Tauk A, Will L, Limme M. Three-dimensional changes of the naso-maxillary complex following rapid maxillary expansion. Angle Orthodontist. 2014;84(1):88-95. | Title |
|  | Bovard D, Giralt A, Trivedi K, Neau L, Kanellos P, Iskandar A, et al. Comparison of the basic morphology and function of 3D lung epithelial cultures derived from several donors. Current Research in Toxicology. 2020;1:56-69. | Title |
|  | Bowe C, O'Neill M, O'Connell JE, Kearns G. The surgical and anaesthetic management of severe dento-facial infections (DFI) - A prospective study. British Journal of Oral and Maxillofacial Surgery. 2017;55(10):e109. | Title |
|  | Bowen-Wright, H.E. and J. Jonklaas, Ectopic intratracheal thyroid: An illustrative case report and literature review. Thyroid, 2005. 15(5): p. 478-484. | Title |
|  | Boyd RN, Davies PSW, Ziviani J, Trost S, Barber L, Ware R, et al. PREDICT-CP: study protocol of implementation of comprehensive surveillance to predict outcomes for school-aged children with cerebral palsy. Bmj Open. 2017;7(7). | Title |
|  | Boynuyogun E, Tuncbilek G. A Clinical Report of the Complete Nasal Agenesis: Reconstruction of Congenital Arhinia and Review of the Literature. Cleft Palate-Craniofacial Journal. 2022. | Title |
|  | Božič, M., et al., Novel method of 3-dimensional soft-tissue analysis for Class III patients. American Journal of Orthodontics and Dentofacial Orthopedics, 2010. 138(6): p. 758-769. | Title |
|  | **Bozzini MFR, Valladares-Neto J, Paiva JB, Rino-Neto J. Sex differences in pharyngeal airway morphology in adults with skeletal Class III malocclusion. Cranio. 2018;36(2):98-105.** | **Included** |
|  | Brandon BM, Stepp WH, Basu S, Kimbell JS, Senior BA, Shockley WW, et al. Nasal Airflow Changes With Bioabsorbable Implant, Butterfly, and Spreader Grafts. Laryngoscope. 2020;130(12):E817-e23. | Title |
|  | Brandstetter K, Patel K. The utility of 3D imaging and virtual planning in nasoalveolar molding appliances for cleft palate babies. Cleft Palate-Craniofacial Journal. 2019;56(1):97. | Title |
|  | **Brasil DM, Kurita LM, Groppo FC, Haiter-Neto F. Relationship of craniofacial morphology in 3-dimensional analysis of the pharynx. Am J Orthod Dentofacial Orthop. 2016;149(5):683-91.e1.** | **Included** |
|  | Bräuer, G., et al., Virtual Reconstruction and Comparative Analyses of the Middle Pleistocene Apidima 2 Cranium (Greece). Anatomical Record, 2020. 303(5): p. 1374-1392. | Title |
|  | Bravo F, Navarrete A, Niño A, Rodríguez E, Martín Ardila C, Guzmán CL. Evaluación tridimensional de la vía aérea mediante tomografía computarizada de haz de cono. Revista Archivo Médico de Camagüey. 2012;16(3):273-81. | Abstract |
|  | Bravo, B.S.F., et al., Effect of the aging process on columella-labial, naso-mental and facial angles and how to apply it in clinical practice. Journal of Cosmetic Dermatology, 2020. 19(12): p. 3383-3388. | Title |
|  | Breeze J, Carr DJ, Mabbott A, Beckett S, Clasper JC. Refrigeration and freezing of porcine tissue does not affect the retardation of fragment simulating projectiles. Journal of Forensic and Legal Medicine. 2015;32:77-83. | Title |
|  | Brennick MJ, Delikatny J, Pack AI, Pickup S, Shinde S, Zhu JX, et al. Tongue Fat Infiltration in Obese Versus Lean Zucker Rats. Sleep. 2014;37(6):1095-U198. | Title |
|  | Brighouse JR, Shepherd K, Santos R, Shivamurthy V. Sinister sinusitis. Arch Dis Child Educ Pract Ed. 2022;107(2):113-5. | Title |
|  | Bril SI, Pezier TF, Tijink BM, Janssen LM, Braunius WW, de Bree R. Preoperative low skeletal muscle mass as a risk factor for pharyngocutaneous fistula and decreased overall survival in patients undergoing total laryngectomy. Head Neck. 2019;41(6):1745-55. | Title |
|  | **Brito FC, Brunetto DP, Nojima MCG. Three-dimensional study of the upper airway in different skeletal Class II malocclusion patterns. Angle Orthod. 2019;89(1):93-101.** | **Included** |
|  | Brock J. A common rheumatology referral of vague constitutional symptoms and raised inflammatory markers. Rheumatology Advances in Practice. 2019;3:i38-i9. | Title |
|  | Broeks IJ, Hermans DJ, Dassel AC, van der Vleuten CJ, van Beynum IM. Propranolol treatment in life-threatening airway hemangiomas: a case series and review of literature. Int J Pediatr Otorhinolaryngol. 2013;77(11):1791-800. | Title |
|  | Bronfman CN. Avaliação das vias aéreas superiores por meio de tomografia computadorizada Cone-beam em pacientes Classe III submetidos à cirurgia bimaxilar. 2016. p. 104-. | Abstract |
|  | Bronoosh, P. and L. Khojastepour, Analysis of pharyngeal airway using lateral cephalogram vs CBCT images: A cross-sectional retrospective study. Open Dentistry Journal, 2015. 9: p. 263-266. | Abstract |
|  | Brons S, Meulstee JW, Loonen TGJ, Nada RM, Kuijpers MAR, Bronkhorst EM, et al. Three-dimensional facial development of children with unilateral cleft lip and palate during the first year of life in comparison with normative average faces. Peerj. 2019;7. | Title |
|  | Brons S, Meulstee JW, Nada RM, Kuijpers MAR, Bronkhorst EM, Berge SJ, et al. Uniform 3D meshes to establish normative facial averages of healthy infants during the first year of life. Plos One. 2019;14(5). | Title |
|  | Brook I. Two cases of diskitis attributable to anaerobic bacteria in children. Pediatrics. 2001;107(2):E26. | Title |
|  | Broujerdi JA, Jacobson R, Schendel S. Volumetric 3-dimensional upper airway analysis in patients with dento-facial deformity following orthognathic surgery. Journal of Oral and Maxillofacial Surgery. 2011;69(9):e-27. | Title |
|  | Broujerdi JA, Jacobson R, Schendel SA. 3D evaluation and analysis of the growth pattern of the upper airway space in normal pediatric to early adult patients. Journal of Oral and Maxillofacial Surgery. 2012;70(9):e-30. | Title |
|  | Broujerdi JA, Jacobson R, Schendel SA. Gender-based morphological variations of the upper airway space in adult non-obstructive sleep apnea patients. Journal of Oral and Maxillofacial Surgery. 2012;70(9):e-23. | Abstract |
|  | Broujerdi JA, Jacobson R, Schendel SA. Upper airway stimulation in patients with obstructive sleep apnea undergoing maxillomandibular advancement. Journal of Oral and Maxillofacial Surgery. 2012;70(9):e-24. | Title |
|  | Brown A, Mackenzie F. Fetal goitre complicating maternal Graves' disease-a case report. BJOG: An International Journal of Obstetrics and Gynaecology. 2018;125:82. | Title |
|  | Brown, A.D., et al., Mitochondrial adaptations to inactivity in diaphragm muscle fibers. Journal of Applied Physiology, 2022. 133(1): p. 191-204. | Title |
|  | Brown, L.A., et al., Differences in management outcome for first branchial cleft anomalies: A comparison of infants and toddlers to older children. International Journal of Pediatric Otorhinolaryngology, 2019. 122: p. 161-164. | Title |
|  | Bruce M, Marji F, Ma I, Lee J, Anstadt E, Chow I, et al. Changes to the cleft nose aesthetic after le fort i advancement: A three-dimensional analysis. Cleft Palate-Craniofacial Journal. 2021;58(4 SUPPL):107. | Title |
|  | Brunetto DP, Velasco L, Koerich L, Araujo MTD. Prediction of 3-dimensional pharyngeal airway changes after orthognathic surgery: A preliminary study. American Journal of Orthodontics and Dentofacial Orthopedics. 2014;146(3):299-309. | Title |
|  | Bruwier A, Poirrier R, Albert A, Maes N, Limme M, Charavet C, et al. Three-dimensional analysis of craniofacial bones and soft tissues in obstructive sleep apnea using cone beam computed tomography. Int Orthod. 2016;14(4):449-61. | Title |
|  | Bücheler, M., et al., Proliferation and differentiation of human osteoblasts from the nasal septum in a new perfusion culture system. HNO, 2008. 56(3): p. 301-305. | Title |
|  | Buck LM, Dalci O, Darendeliler MA, Papadopoulou AK. Effect of Surgically Assisted Rapid Maxillary Expansion on Upper Airway Volume: A Systematic Review. Journal of Oral and Maxillofacial Surgery. 2016;74(5):1025-43. | Title |
|  | Buck LM, Dalci O, Darendeliler MA, Papageorgiou SN, Papadopoulou AK. Volumetric upper airway changes after rapid maxillary expansion: a systematic review and meta-analysis. Eur J Orthod. 2017;39(5):463-73. | Title |
|  | Bugaighis I. et al. 3D comparison of average faces in subjects with oral clefts. European Journal of Orthodontics, 2014. 36(4): 365-372. | Title |
|  | Bui NL, Ong SH, Foong KW. Automatic segmentation of the nasal cavity and paranasal sinuses from cone-beam CT images. Int J Comput Assist Radiol Surg. 2015;10(8):1269-77. | Title |
|  | Bulut O, Liu CYJ, Gurcan S, Hekimoglu B. Prediction of nasal morphology in facial reconstruction: Validation and recalibration of the Rynn method. Legal Medicine. 2019;40:26-31. | Title |
|  | Bulycheva EA, Mamedov AA, Dybov AM, Ishchenko TA, Loktionov AA. Protocol of cone beam computed tomography analysis for patients with craniomandibular dysfunction. Stomatologiia (Mosk). 2020;99(6):94-100. | Title |
|  | Buras M, Simoncini A, Gungor A. Auto-obliteration of maxillary sinuses through osteoneogenesis in children with cystic fibrosis: A possible new way to reduce morbidity. Am J Otolaryngol. 2018;39(6):737-40. | Title |
|  | Burdelsky BA, McCarthy RC. Scaling and developmental integration in the Paranthropus head. American Journal of Physical Anthropology. 2012;147:108-9. | Title |
|  | Burger A, Roosenboom J, Hossain M, Weinberg SM, Hecht JT, Posey KL. Mutant COMP shapes growth and development of skull and facial structures in mice and humans. Molecular Genetics & Genomic Medicine. 2020;8(7). | Title |
|  | Burke, P.H. and C.A. Hughes-Lawson, Stereophotogrammetric study of growth and development of the nose. American Journal of Orthodontics and Dentofacial Orthopedics, 1989. 96(2): p. 144-151. | Title |
|  | Burke, P.H. and C.A. Hughes-Lawson, The growth and development of the soft tissues of the human face. Journal of Anatomy, 1988. 158: p. 115-120. | Title |
|  | Burkhard JPM, Dietrich AD, Jacobsen C, Roos M, Lübbers HT, Obwegeser JA. Cephalometric and three-dimensional assessment of the posterior airway space and imaging software reliability analysis before and after orthognathic surgery. Journal of Cranio-Maxillofacial Surgery. 2014;42(7):1428-36. | Title |
|  | Burns P, Meiburg E. Sediment-laden fresh water above salt water: nonlinear simulations. Journal of Fluid Mechanics.2015;762:156-95. | Title |
|  | Busayarat, S. and T. Zrimec. Detection of bronchopulmonary segments on high-resolution CT preliminary results. in Proceedings - IEEE Symposium on Computer-Based Medical Systems. 2007. | Title |
|  | Businco LD, Lauriello M, Marsico C, Corbisiero A, Cipriani O, Tirelli GC. Psychological aspects and treatment of patients with nasal septal perforation due to cocaine inhalation. Acta Otorhinolaryngologica Italica. 2008;28(5):247-51. | Title |
|  | Butaric LN, Klocke R. Internal craniofacial morphology of high-altitude Tibetans may reflect unique adaptations to hypoxic environments. American Journal of Physical Anthropology. 2017;162:133. | Title |
|  | Butaric LN, Maddux SD. Morphological Covariation between the Maxillary Sinus and Midfacial Skeleton among Sub-Saharan and Circumpolar Modern Humans. American Journal of Physical Anthropology. 2016;160(3):483-97. | Title |
|  | Butaric LN, McCarthy RC, Broadfield DC. A Preliminary 3D Computed Tomography Study of the Human Maxillary Sinus and Nasal Cavity. American Journal of Physical Anthropology. 2010;143(3):426-36. | Abstract |
|  | Butaric LN, Wadle M, Gascon J. Anatomical Variation in Maxillary Sinus Ostium Positioning: Implications for Nasal-Sinus Disease. Anatomical Record. 2019;302(6):917-30. | Title |
|  | Butaric LN. Differential Scaling Patterns in Maxillary Sinus Volume and Nasal Cavity Breadth Among Modern Humans. Anatomical Record. 2015;298(10):1710-21. | Title |
|  | Butaric, L.N., et al., Ontogenetic variation in human nasal morphology. Anatomical Record, 2022. 305(8): p. 1910-1937. | Title |
|  | Butler JP, Loring SH, Patz S, Tsuda A, Yablonskiy D, Quirk JD, et al. Lung regrowth in an adult human post pneumonectomy. American Journal of Respiratory and Critical Care Medicine. 2013;187. | Title |
|  | Butler JP, Loring SH, Patz S, Tsuda A, Yablonskiy DA, Mentzer SJ. Evidence for adult lung growth in humans. N Engl J Med. 2012;367(3):244-7. | Title |
|  | Butskiy O, Anderson DW. Upper airway obstruction due to a change in altitude: first report in fifty years. J Otolaryngol Head Neck Surg. 2016;45:9. | Title |
|  | Butterfield KJ, Marks PL, McLean L, Newton J. Linear and volumetric airway changes after maxillomandibular advancement for obstructive sleep apnea. J Oral Maxillofac Surg. 2015;73(6):1133-42. | Title |
|  | Butterfield KJ, Marks PLG, McLean L, Newton J. Pharyngeal airway morphology in healthy individuals and in obstructive sleep apnea patients treated with maxillomandibular advancement: a comparative study. Oral Surgery Oral Medicine Oral Pathology Oral Radiology. 2015;119(3):285-92. | Title |
|  | Buttery, T.A., et al., Postnatal presence of paraseptal cartilages in humans: A description of morphology and size. Acta Oto-Laryngologica, 2000. 120(1): p. 77-80. | Title |
|  | Buyuk SK, Celikoglu M, Benkli YA, Sekerci AE. Evaluation of the Transverse Craniofacial Morphology of Adolescents With Repaired Unilateral Cleft Lip and Palate Using Cone-Beam Computed Tomography.Journal of Craniofacial Surgery. 2016;27:1870-4. | Title |
|  | Buyuk SK, Simsek H, Hatal S, Sarimehmetoglu N, Odabas D. Comparison of mini-screw-assisted rapid maxillary expansion in adolescents with different body mass indices: a prospective clinical study. Australasian Orthodontic Journal. 2022;38(1):41-50. | Title |
|  | Buyukcavus MH, Findik Y, Baykul T. Evaluation of Changes in Nasal Projection After Surgically Assisted Rapid Maxillary Expansion With 3dMD Face System. Journal of Craniofacial Surgery. 2020;31(5):E462-E5. | Title |
|  | Cabezón A R, Willson E M, García-Huidobro N F, Lira R K, Solar G A. Quiste nasolabial: Reporte de dos casos. Revista de otorrinolaringología y cirugía de cabeza y cuello. 2020;80(2):178-83. | Title |
|  | **Cabral M, de Queiroz Ribeiro LR, Cardeal CM, Bittencourt MA, Crusoé-Rebello IM, Souza-Machado A. Evaluation of the oropharynx in class I and II skeletal patterns by CBCT. Oral Maxillofac Surg. 2017;21(1):27-31.** | **Included** |
|  | Cademartiri F, Luccichenti G, Laganà F, Brevi B, Sesenna E, Pavone P. Effective clinical outcome of a mandibular distraction device using three-dimensional CT with volume rendering in Pierre-Robin sequence. Acta Biomedica de l'Ateneo Parmense 2004;75:122-36. | Title |
|  | Caganova V, Borsky J, Smahel Z, Veleminska J. Facial Growth and Development in Unilateral Cleft Lip and Palate: Comparison Between Secondary Alveolar Bone Grafting and Primary Periosteoplasty. Cleft Palate-Craniofacial Journal. 2014;51(1):15-22. | Title |
|  | Cai, D., et al., A Three-Dimensional Study of the Nasolabial Soft Tissue Symmetry in Children With Unilateral Complete Cleft Lip and Palate Using Traditional and Split-Type Nasoalveolar Molding. The Journal of craniofacial surgery, 2020. 31(6): p. 1785-1789. | Title |
|  | Caiado GM, Evangelista K, Freire M, Almeida FT, Pacheco-Pereira C, Flores-Mir C, et al. Orthodontists' criteria for prescribing cone-beam computed tomography-a multi-country survey. Clin Oral Investig. 2022;26(2):1625-36. | Title |
|  | Caiulo S, Pitea M, Partenope C, Gallo D, Damia C, Adavastro M, et al. Growth hormone deficiency (GHD) in a patient with persistence of the craniopharyngeal canal with cephalocele. Hormone Research in Paediatrics. 2018;90:516. | Title |
|  | Çakli, H., et al., Use of cone beam computed tomography in otolaryngologic treatments. European Archives of Oto-Rhino-Laryngology, 2012. 269(3): p. 711-720. | Title |
|  | Calandrelli R, Pilato F, D'Apolito G, Tenore L, Onesimo R, Leoni C, et al. Airways and craniofacial assessment in children affected by achondroplasia with and without sleep-disordered breathing: quantitative magnetic resonance study. Childs Nervous System. 2022;38(6):1147-54. | Title |
|  | Caldas LD, Takeshita WM, Machado AW, Bittencourt MAV. Effect of rapid maxillary expansion on nasal cavity assessed with cone-beam computed tomography. Dental Press J Orthod. 2020;25(3):39-45. | Title |
|  | Calvert, R., Structure of rat ultimobranchial bodies after birth. The Anatomical Record, 1975. 181(3): p. 561-579. | Title |
|  | Camacho J, Heyde A, Bhullar BAS, Haelewaters D, Simmons NB, Abzhanov A. Peramorphosis, an evolutionary developmental mechanism in neotropical bat skull diversity. Developmental Dynamics. 2019;248(11):1129-43. | Title |
|  | Camargo, I.B., et al., The nasal lift technique for augmentation of the maxillary ridge: Technical note. British Journal of Oral and Maxillofacial Surgery, 2015. 53(8): p. 771-774. | Title |
|  | Camp AL, Scott B, Brainerd EL, Wilga CD. Dual function of the pectoral girdle for feeding and locomotion in white-spotted bamboo sharks. Proceedings of the Royal Society B-Biological Sciences. 2017;284(1859). | Title |
|  | Campos L, Drake A, Trindade I, Andre Pimenta L, Kimbell J, Trindade-Suedam I. Sleep apnea in cleft lip and palate and skeletal class III individuals: A polysomnographic, tomographic, and computational fluid dynamics study. Cleft Palate-Craniofacial Journal. 2018;55(1):52. | Title |
|  | Campos LD, Trindade IEK, Yatabe M, Trindade SHK, Pimenta LA, Kimbell J, et al. Reduced pharyngeal dimensions and obstructive sleep apnea in adults with cleft lip/palate and Class III malocclusion. Cranio. 2021;39(6):484-90. | Title |
|  | Camps-Perepérez I, Guijarro-Martínez R, Peiró-Guijarro MA, Hernández-Alfaro F. The value of cone beam computed tomography imaging in surgically assisted rapid palatal expansion: a systematic review of the literature. Int J Oral Maxillofac Surg 2017;46:827-38. | Title |
|  | Canellas JVdS. Estudo das alterações volumétricas faríngeas após cirurgia ortognática em pacientes classe III e sua influência no desenvolvimento da apneia obstrutiva do sono. 2015. p. 57-. | Title |
|  | Cannie M, Jani J, De Keyzer F, Roebben I, Dymarkowski S, Deprest J. Diffusion-weighted MRI in lungs of normal fetuses and those with congenital diaphragmatic hernia. Ultrasound in Obstetrics & Gynecology. 2009;34(6):678-86. | Title |
|  | Cantarella, D., et al., Changes in the midpalatal and pterygopalatine sutures induced by micro-implant-supported skeletal expander, analyzed with a novel 3D method based on CBCT imaging. Progress in Orthodontics, 2017. 18(1). | Title |
|  | Cappellette Jr M, Alves FEMM, Nagai LHY, Fujita RR, Pignatari SSN. Impact of rapid maxillary expansion on nasomaxillary complex volume in mouth-breathers. Dental Press Journal of Orthodontics. 2017;22(3):79-88. | Title |
|  | Cappellini I, Picciafuochi F, Ostento D, Danti G, De Gaudio AR, Adembri C. Recovery of muscle function after deep neuromuscular block by means of diaphragm ultrasonography and adductor of pollicis acceleromyography with comparison of neostigmine vs. sugammadex as reversal drugs: study protocol for a randomized controlled trial. Trials. 2018;19(1). | Title |
|  | Caprioglio A, Meneghel M, Fastuca R, Zecca PA, Nucera R, Nosetti L. Rapid maxillary expansion in growing patients: Correspondence between 3-dimensional airway changes and polysomnography. International Journal of Pediatric Otorhinolaryngology. 2014;78(1):23-7. | Title |
|  | Captier G, Faure JM, Baümler M, Bonnel F, Daures JP. Anatomy and growth of the fetal soft palate: a cadaveric study to improve its ultrasonographic observation. Cleft Palate-Craniofacial Journal. 2008;45(4):439-45. | Title |
|  | Captier G, Tourbach S, Bigorre M, Saguintaah M, El Ahmar J, Montoya P. Anatomical consideration of the congenital nasal pyriform aperture stenosis: Localized dysostosis without interorbital hypoplasia. Journal of Craniofacial Surgery. 2004;15(3):490-6. | Title |
|  | Carignan C. et al. The role of the pharynx and tongue in enhancement of vowel nasalization: A real-time MRI investigation of french nasal vowels. in Proceedings of the Annual Conference of the International Speech Communication Association. Interspeeech. 2013. | Title |
|  | Carlisle T, Carthy ER, Glasser M, Drivas P, McMillan A, Cowie MR, et al. Upper airway factors that protect against obstructive sleep apnoea in healthy older males. European Respiratory Journal. 2014;44(3):685-93. | Title |
|  | Carlson C, Sung J, McComb RW, Machado AW, Moon W. Microimplant-assisted rapid palatal expansion appliance to orthopedically correct transverse maxillary deficiency in an adult. Am Journal of Orthodontics and Dentofacial Orthopedics. 2016;149(5):716-28. | Title |
|  | Caron C, Pluijmers BI, Joosten KFM, Mathijssen IMJ, Dunaway DJ, Padwa PL, et al. Functional problems in patients with craniofacial microsomia. International Journal of Oral and Maxillofacial Surgery. 2019;48:8. | Title |
|  | Carrigy, N.B., et al., Pediatric in vitro and in silico models of deposition via oral and nasal inhalation. Journal of Aerosol Medicine and Pulmonary Drug Delivery, 2014. 27(3): p. 149-169. | Title |
|  | Carroll A, Amirav I, Marchand R, Olmstead D, Majaesic C, MacLean JE, et al. 3d modeled custom-made non-invasive positive pressure mask in an infant. American Journal of Respiratory and Critical Care Medicine. 2014;189. | Title |
|  | Casasayas M, García-Lorenzo J, Gómez-Ansón B, Medina V, Fernández A, Quer M, et al. Low skeletal muscle mass assessed directly from the 3rd cervical vertebra can predict pharyngocutaneous fistula risk after total laryngectomy in the male population. European Archives of Oto-Rhino-Laryngology. 2022;279(2):853-63. | Title |
|  | Cascone P, Basile E, Saccucci M, Di Carlo G, Angeletti D, Ramieri V, et al. Fast and Early Mandibular Osteodistraction: The Long-Term Follow-Up of Mandibular Distraction Osteogenesis on Teeth Position. Journal of Craniofacial Surgery. 2015;26(8):2325-8. | Title |
|  | Castaño, J.E. and D.H. Chi, Pyriform aperture stenosis repair in infants. Operative Techniques in Otolaryngology - Head and Neck Surgery, 2018. 29(2): p. 51-54. | Title |
|  | Castejón-González AC, Stefanovski D, Reiter AM. Surgical Treatment and Outcome of Acquired Midline Palate Defects in Cats. Frontiers in Veterinary Science. 2022;9. | Title |
|  | **Castro-Silva L, Monnazzi MS, Spin-Neto R, Moraes M, Miranda S, Gabrielli MFR, et al. Cone-beam evaluation of pharyngeal airway space in class I, II, and III patients. Oral Surgery Oral Medicine Oral Pathology Oral Radiology. 2015;120(6):679-83.** | **Included** |
|  | Cavalcanti, M.C., et al., Maxillary sinus floor pneumatization and alveolar ridge resorption after tooth loss: A cross-sectional study. Brazilian Oral Research, 2018. 32. | Title |
|  | Cavalcanti, M.G.P., S.S. Rocha, and M.W. Vannier, Craniofacial measurements based on 3D-CT volume rendering: Implications for clinical applications. Dentomaxillofacial Radiology, 2004. 33(3): p. 170-176. | Abstract |
|  | Cavers A, Ryu M, Abraham T, Halayko AJ, Dorscheid DR, Wadsworth SJ. Bio-airway research offering new concepts in health (bronch) partnership-developing novel scaffolds for airway engineering. American Journal of Respiratory and Critical Care Medicine. 2013;187. | Title |
|  | Cavka M, Janković I, Sikanjić PR, Ticinović N, Rados S, Ivanac G, et al. Insights into a mummy: a paleoradiological analysis. Coll Antropol. 2010;34(3):797-802. | Title |
|  | Celikoglu M, Bayram M, Sekerci AE, Buyuk SK, Toy E. Comparison of pharyngeal airway volume among different vertical skeletal patterns: a cone-beam computed tomography study. Angle Orthod. 2014;84(5):782-7. | Full text  Only Class I |
|  | Celikoglu M, Buyuk SK, Ekizer A, Sekerci AE, Sisman Y. Assessment of the soft tissue thickness at the lower anterior face in adult patients with different skeletal vertical patterns using cone-beam computed tomography. Angle Orthodontist. 2015;85(2):211-7. | Title |
|  | Celikoglu M, Buyuk SK, Ekizer A, Unal T. Pharyngeal airway effects of Herbst and skeletal anchored Forsus FRD EZ appliances. International Journal of Pediatric Otorhinolaryngology. 2016;90:23-8. | Title |
|  | Celikoglu M, Buyuk SK, Hatipoglu M, Sekerci AE, Ciftci ME. Evaluation of dehiscence and fenestration in adolescents affected by bilateral cleft lip and palate using cone-beam computed tomography. American Journal of Orthodontics and Dentofacial Orthopedics. 2017;152(4):458-64. | Title |
|  | Celikoglu M, Buyuk SK, Sekerci AE, Ersoz M, Celik S, Sisman Y. Facial soft-tissue thickness in patients affected by bilateral cleft lip and palate: A retrospective cone-beam computed tomography study. American Journal of Orthodontics and Dentofacial Orthopedics. 2014;146(5):573-8. | Title |
|  | Celikoglu M, Buyuk SK, Sekerci AE, Ucar FI, Cantekin K. Three-dimensional evaluation of the pharyngeal airway volumes in patients affected by unilateral cleft lip and palate. American Journal of Orthodontics and Dentofacial Orthopedics. 2014;145(6):780-6. | Title |
|  | Celikoglu M, Buyukcavus MH. Changes in pharyngeal airway dimensions and hyoid bone position after maxillary protraction with different alternate rapid maxillary expansion and construction protocols: A prospective clinical study. Angle Orthodontist. 2017;87(4):519-25. | Title |
|  | Celikoglu M, Ucar FI, Buyuk SK, Celik S, Sekerci AE, Akin M. Evaluation of the mandibular volume and correlating variables in patients affected by unilateral and bilateral cleft lip and palate: a cone-beam computed tomography study. Clinical Oral Investigations. 2016;20(7):1741-6. | Title |
|  | Celikoglu M, Ucar FI, Sekerci AE, Buyuk SK, Ersoz M, Sisman Y. Assessment of pharyngeal airway volume in adolescent patients affected by bilateral cleft lip and palate using cone beam computed tomography. Angle Orthod. 2014;84(6):995-1001. | Title |
|  | Cerminara, A. and N.D. Sandham. Boundary-layer receptivity and breakdown in hypersonic flow over a swept blunt wedge with three-dimensional freestream acoustic disturbances. in 8th AIAA Flow Control Conference. 2016. | Title |
|  | Cerone JB, Pinheiro JMB. Tracheal Length Measurement in Intubated Neonates to Guide the Design and Use of Endotracheal Tube Glottic Depth Markings. Children-Basel. 2022;9(2). | Title |
|  | Cevidanes LH, Styner MA, Proffit WR. Image analysis and superimposition of 3-dimensional cone-beam computed tomography models. Am J Orthod Dentofacial Orthop. 2006;129(5):611-8. | Title |
|  | Cha JY, Mah J, Sinclair P. Incidental findings in the maxillofacial area with 3-dimensional cone-beam imaging. Am J Orthod Dentofacial Orthop. 2007;132(1):7-14. | Title |
|  | Cha, J.K., et al., Core Ossification of Bone Morphogenetic Protein-2-Loaded Collagenated Bone Mineral in the Sinus. Tissue Engineering - Part A, 2021. 27(13-14): p. 905-913. | Title |
|  | Chacko R, Kumar S, Paul A. Three Dimensional Morphological Grading of the Ankylosed TMJ for Surgical Planning: A Retrospective Observational Study. Journal of Clinical and Diagnostic Research. 2022;16(2):ZC01-ZC6. | Title |
|  | Chaisooktaksin N, Chimruang J, Worasakwutiphong S, Tansalarak R. Three-dimensional Changes of Maxillary Alveolar Morphology After Using Modified Nasoalveolar Molding in Patients with Complete Unilateral Cleft lip and Palate. Cleft Palate-Craniofacial Journal. 2022. Online ahead of print. | Title |
|  | Chait P. Future directions in interventional pediatric radiology. Pediatric Clinics of North America. 1997;44(3):763-&. | Title |
|  | Chakraborty R, Park HN, Tan CC, Weiss P, Prunty MC, Pardue MT. Association of Body Length with Ocular Parameters in Mice. Optometry and Vision Science. 2017;94(3):387-94. | Title |
|  | Chakravarty MM, Aleong R, Leonard G, Perron M, Pike GB, Richer L, et al. Automated analysis of craniofacial morphology using magnetic resonance images. PLoS ONE. 2011;6(5). | Title |
|  | Chakravarty, M.M., et al., Automated analysis of craniofacial morphology using magnetic resonance images. PLoS ONE, 2011. 6(5). |  |
|  | Chalwadi UK, Swamy N, Agarwal A, Gauss CH, Greenberg SB, Lyons KA. Determining normal values for lower trachea and bronchi size in children by computed tomography (CT). Pediatric Pulmonology. 2021;56(9):2940-8. | Title |
|  | Chan HK, Phipps PR, Gonda I, Cook P, Fulton R, Young I, et al. REGIONAL DEPOSITION OF NEBULIZED HYPODENSE NONISOTONIC SOLUTIONS IN THE HUMAN RESPIRATORY-TRACT. European Respiratory Journal. 1994;7(8):1483-9. | Title |
|  | **Chan L, Kaczynski R, Kang HK. A cross-sectional retrospective study of normal changes in the pharyngeal airway volume in white children with 3 different skeletal patterns from age 9 to 15 years: Part 1. Am J Orthod Dentofacial Orthop. 2020;158(5):710-21.** | **Included** |
|  | Chan L, Tiew P, Koh M, Lee C, Ter S, Yong V, et al. A Novel 'Apical-out' Human Airway Organoid System for the Study of Host-Pathogen Interaction in COPD. American Journal of Respiratory and Critical Care Medicine. 2021;203(9). | Title |
|  | Chandiramohan A, Dabaghi M, Aguiar JA, Tiessen N, Stewart M, Cao QT, et al. Development and validation of an open-source, disposable, 3d-printed in vitro environmental exposure system for transwell culture inserts. ERJ Open Research. 2021;7(1). | Title |
|  | Chang AB. Bronchiectasis in the 21th century: diagnosis and management. Pediatric pulmonology. 2019;54:S10‐S1. | Title |
|  | Chang CS, Wallace CG, Hsiao YC, Hsieh YJ, Wang YC, Chen NH, et al. Airway Changes after Cleft Orthognathic Surgery Evaluated by Three-Dimensional Computed Tomography and Overnight Polysomnographic Study. Scientific Reports. 2017;7. | Title |
|  | Chang DT, Zhou YH, Liu WT. Evaluation of cone-beam computed tomography on upper airway changes after alternating rapid palatal expansion and constriction. Beijing da xue xue bao [Journal of Peking University Health sciences]. 2017;49(4):685‐90. | Title |
|  | Chang EH, Pezzulo AA, Potash A, Meyerholz DK, Stoltz DA, Welsh M, et al. Sinus disease in CFTR-/- pigs. Pediatric Pulmonology. 2011;46:280. | Title |
|  | Chang MK, Sears C, Huang JC, Miller AJ, Kushner HW, Lee JS. Correlation of Airway Volume With Orthognathic Surgical Movement Using Cone-Beam Computed Tomography. Journal of Oral and Maxillofacial Surgery. 2015;73(12):S67-S76. | Title |
|  | Chang Y, Koenig LJ, Pruszynski JE, Bradley TG, Bosio JA, Liu DW. Dimensional changes of upper airway after rapid maxillary expansion: A prospective cone-beam computed tomography study. Am J Orthod Dentofacial Orthoped. 2013;143(4):462-70. | Title |
|  | Chang, E.T., et al., Genial tubercle position and genioglossus advancement in obstructive sleep apnea (OSA) treatment: a systematic review. Maxillofacial Plastic and Reconstructive Surgery, 2019. 41(1). | Title |
|  | Chang, H.W., et al., Intraoperative navigation for single-splint two-jaw orthognathic surgery: From model to actual surgery. Journal of Cranio-Maxillofacial Surgery, 2015. 43(7): p. 1119-1126. | Title |
|  | Chaturvedi A, Lee Z. Three-dimensional segmentation and skeletonization to build an airway tree data structure for small animals. Physics in Medicine and Biology. 2005;50(7):1405-19. | Title |
|  | Chaudhry IG, Rushton Z, Quinney NL, Boyles SE, Cholon DM, Sears PR, et al. CFTR rescue affects secreted mucins and mucus. Pediatric Pulmonology. 2015;50:225. | Title |
|  | Chen CY, Lin CC, Ko EW. Effects of two alar base suture techniques suture techniques on nasolabial changes after bimaxillary orthognathic surgery in Taiwanese patients with class III malocclusions. International journal of oral and maxillofacial surgery. 2015;44(7):816‐22. | Title |
|  | Chen D, Zhang YM, Zhang YM, Wang YH, Jia RX. Characterization of the heteroepitaxial growth of 3C-SiC on Si during low pressure chemical vapor deposition. Chinese Science Bulletin. 2010;55(27-28):3102-6. | Title |
|  | Chen DL, Blom H, Sanchez S, Tafforeau P, Marss T, Ahlberg PE. Development of cyclic shedding teeth from semi-shedding teeth: the inner dental arcade of the stem osteichthyan Lophosteus. Royal Society Open Science. 2017;4(5). | Title |
|  | Chen H, Aarab G, De Lange J, Van Der Stelt P, Lobbezoo F, Darendeliler MA, et al. Differences in three-dimensional craniofacial anatomy between responders and non responders to mandibular advancement splint treatment in obstructive sleep apnea patients. Sleep Medicine. 2017;40:e54. | Title |
|  | Chen HC, Wang CJ, Lo YL, Hsu HC, Huang CG, Kuo IC, et al. Parapharyngeal fat pad area at the subglosso-supraglottic level is associated with corresponding lateral wall collapse and apnea-hypopnea index in patients with obstructive sleep apnea: a pilot study. Scientific Reports. 2019;9. | Title |
|  | Chen K, Xiao D, Abotaleb B, Chen HZ, Li YF, Zhu SS. Accuracy of Virtual Surgical Planning in Treatment of Temporomandibular Joint Ankylosis Using Distraction Osteogenesis: Comparison of Planned and Actual Results. Journal of Oral and Maxillofacial Surgery. 2018;76(11). | Title |
|  | Chen S, Schoen J. Air-liquid interface cell culture: From airway epithelium to the female reproductive tract. Reproduction in Domestic Animals. 2019;54:38-45. | Title |
|  | Chen SC, McDevitt H, Clement WA, Wynne DM, Mason A, Donaldson M, et al. Congenital nasal pyriform aperture stenosis and pituitary abnormalities: Case series of 20 patients and a management guideline for early identification of pituitary insufficiency. Hormone Research in Paediatrics. 2014;82:150. | Title |
|  | Chen SC, McDevitt H, Clement WA, Wynne DM, Mason A, Donaldson MDC, et al. Early identification of pituitary dysfunction in congenital nasal pyriform aperture stenosis: Recommendations based on experience in a single centre. Hormone Research in Paediatrics. 2015;83(5):302-10. | Title |
|  | Chen WE, Mou HG, Qian YF, Qian LW. Evaluation of the position and morphology of tongue and hyoid bone in skeletal Class II malocclusion based on cone beam computed tomography. Bmc Oral Health. 2021;21(1). | Abstract |
|  | Chen X, Liu D, Liu J, Wu Z, Xie Y, Li L, et al. Three-dimensional evaluation of the upper airway morphological changes in growing patients with skeletal class III malocclusion treated by protraction headgear and rapid palatal expansion: A comparative research. PLoS ONE. 2015;10(8). | Title |
|  | Chen X, Zou CC, Dong GP, Liang L, Zhao ZY. Cranio-osteoarthropathy: a rare variant of hypertrophic osteoarthropathy. Irish Journal of Medical Science. 2012;181(2):257-61. | Title |
|  | Chen YB, Jiang JH, Guo LC, Huang JA. Primary tracheal papilloma disguised as asthma:A case report. J Asthma. 2016;53(10):1090-3. | Title |
|  | Chen YF, Liao YF. Surgical-orthodontic treatment for class II asymmetry: Outcome and influencing factors. Cleft Palate-Craniofacial Journal. 2019;56(1):38. | Title |
|  | **Chen YS, Chou ST, Cheng JH, Chen SC, Pan CY, Tseng YC. Importance in the Occurrence Distribution of Minimum Oropharyngeal Cross-Sectional Area in the Different Skeletal Patterns Using Cone-Beam Computed Tomography. BioMed Research International. 2021;2021.** | **Included** |
|  | Chen, C.P., et al., Prenatal diagnosis of hydrancephaly and enlarged cerebellum and cisterna magna in a fetus with thanatophoric dysplasia type II and a review of prenatal diagnosis of brain anomalies associated with thanatophoric dysplasia. Taiwanese Journal of Obstetrics and Gynecology, 2018. 57(1): p. 119-122. | Title |
|  | Chen, H., et al., Differences in three-dimensional craniofacial anatomy between responders and nonresponders to mandibular advancement splint treatment in obstructive sleep apnoea patients. European Journal of Orthodontics, 2019. 41(3): p. 308-315. | Title |
|  | Chen, N., et al., Changes of the upper airway in children with Class Ⅱ mandibular retrusion and snoring during night before and after functional treatment by sagittal-guidance Twin-block appliance. Shanghai kou qiang yi xue = Shanghai journal of stomatology, 2021. 30(3): p. 273-277. | Title |
|  | Chen, W., Y.H. Liu, and Q. Xu, Effect of maximum anchorage extraction on upper airway in adolescent patients with bimaxillary protrusion. Shanghai kou qiang yi xue = Shanghai journal of stomatology, 2018. 27(4): p. 419-423. | Title |
|  | Chen, X. and L. Xu, Development of a surgical navigation system based on 3D slicer using augmented reality (AR) technology, in Computer-Assisted Surgery: New Developments, Applications and Potential Hazards. 2015. p. 1-25. | Title |
|  | Chen, X., Computer-assisted surgery: New developments, applications and potential hazards. Computer-Assisted Surgery: New Developments, Applications and Potential Hazards. 2015. 1-154. | Title |
|  | Chen, X., et al., Cranio-osteoarthropathy: A rare variant of hypertrophic osteoarthropathy. Irish Journal of Medical Science, 2012. 181(2): p. 257-261. | Title |
|  | Chen, Y., et al., Effect of large incisor retraction on upper airway morphology in adult bimaxillary protrusion patients: Three-dimensional multislice computed tomography registration evaluation. Angle Orthodontist, 2012. 82(6): p. 964-970. | Title |
|  | Chen, Y.J., et al., Airway increase after open bite closure with temporary anchorage devices for intrusion of the upper posteriors: Evidence from 2D cephalometric measurements and 3D magnetic resonance imaging. J Oral Rehab, 2018. 45(12): p. 939-947. | Title |
|  | Chen, Z., et al. Automatic segmentation of 3D lung airway without leakage. in ACM International Conference Proceeding Series. 2018. | Title |
|  | Cheng, S.F., et al., Xq chromosome duplication in males: Clinical, cytogenetic and array CGH characterization of a new case and review. American Journal of Medical Genetics, 2005. 135 A(3): p. 308-313. | Title |
|  | Cheng, S.L., Immunologic pathophysiology and airway remodeling mechanism in severe asthma: Focused on ige-mediated pathways. Diagnostics, 2021. 11(1). | Title |
|  | Cheung GC, Dalci O, Mustac S, Papageorgiou SN, Hammond S, Darendeliler MA, et al. The upper airway volume effects produced by Hyrax, Hybrid-Hyrax, and Keles keyless expanders: a single-centre randomized controlled trial. European journal of orthodontics. 2021;43(3):254‐64. | Title |
|  | Cheung R, Prince M. Comparison of craniofacial skeletal characteristics of infants with bilateral choanal atresia and an age-matched normative population: computed tomography analysis. J Otolaryngol. 2001;30(3):173-8. | Title |
|  | Cheung T, Oberoi S. Three Dimensional Assessment of the Pharyngeal Airway in Individuals with Non-Syndromic Cleft Lip and Palate. PLoS ONE. 2012;7(8). | Title |
|  | Cheung, L.K., et al., Three-dimensional cephalometric norms of Chinese adults in Hong Kong with balanced facial profile. Oral Surgery, Oral Medicine, Oral Pathology, Oral Radiology and Endodontology, 2011. 112(2): p. e56-e73. | Title |
|  | Chevillard M, Hinnrasky J, Pierrot D, Zahm JM, Klossek JM, Puchelle E. Differentiation of human surface upper airway epithelial cells in primary culture on a floating collagen gel. Epithelial Cell Biology. 1993;2(1):17-25. | Title |
|  | Chi L, Comyn FL, Keenan BT, Cater J, Maislin G, Pack AI, et al. Heritability of craniofacial structures in normal subjects and patients with sleep apnea. Sleep. 2014;37(10):1689-98F. | Title |
|  | Chi L, Comyn FL, Mitra N, Reilly MP, Wan F, Maislin G, et al. Identification of craniofacial risk factors for obstructive sleep apnoea using three-dimensional MRI. European Respiratory Journal. 2011;38(2):348-58. | Title |
|  | Chi L, Thorne-FitzGerald MD, Comyn F, Gislason T, Arnardottir ES, Benediktsdottir B, et al. Upper airway anatomic structures in apneic men and women, matched on age, BMI and AHI. American Journal of Respiratory and Critical Care Medicine. 2010;181(1). | Title |
|  | Chi L, Wang J, Sun Y, Wang Z, Li X, Qu W, et al. Interethnic comparison of anatomic risk factors for OSA between native chinese and icelandic caucasians. American Journal of Respiratory and Critical Care Medicine. 2013;187. | Title |
|  | Chiang CC, Jeffres MN, Miller A, Hatcher DC. Three-dimensional airway evaluation in 387 subjects from one university orthodontic clinic using cone beam computed tomography. Angle Orthod. 2012;82(6):985-92. | Title |
|  | Chiesa-Estomba, C.M., et al., Three-Dimensional Bioprinting Scaffolding for Nasal Cartilage Defects: A Systematic Review. Tissue Engineering and Regenerative Medicine, 2021. 18(3): p. 343-353. | Title |
|  | Chi-Fishman G. Quantitative lingual, pharyngeal and laryngeal ultrasonography in swallowing research: A technical review. Clinical Linguistics & Phonetics. 2005;19(6-7):589-604. | Title |
|  | Cho G, Huon L, Liu S. Maxillomandibular advancement-the name tells half the story. Sleep Medicine. 2015;16:S17-S8. | Title |
|  | **Cho, H.N., et al., Accuracy of convolutional neural networks-based automatic segmentation of pharyngeal airway sections according to craniofacial skeletal pattern. American Journal of Orthodontics and Dentofacial Orthopedics, 2022.** | **Included**  **(descriptive data)** |
|  | Cho, M.H., et al., Cluster analysis in severe emphysema subjects using phenotype and genotype data: An exploratory investigation. Respiratory Research, 2010. 11. | Title |
|  | Choi JJ, Burton CS, Danehy AR, Voss SD. Neck CT angiography examinations for pediatric oropharyngeal trauma: diagnostic yield and proposal of a new targeted technique. Pediatric Radiology. 2020;50(11):1602-9. | Title |
|  | Choi JW, Lee JY, Oh TS, Kwon SM, Yang SJ, Koh KS. Frontal soft tissue analysis using a 3 dimensional camera following two-jaw rotational orthognathic surgery in skeletal class III patients. Journal of Cranio-Maxillofacial Surgery. 2014;42(3):220-6. | Title |
|  | Choi JY, Oh SH, Kim SH, Ahn HW, Kang YG, Choi YS, et al. Effectiveness of 2D radiographs in detecting CBCT-based incidental findings in orthodontic patients. Sci Rep. 2021;11(1):9280. | Title |
|  | Choi YK, Park SB, Kim YI, Son WS. Three-dimensional evaluation of midfacial asymmetry in patients with nonsyndromic unilateral cleft lip and palate by cone-beam computed tomography. Korean Journal of Orthodontics. 2013;43(3):113-9. | Title |
|  | Choi, J., et al., The long-term changes of hard palatal bony cleft defects after palatoplasty in unilateral complete cleft lip and palate. Journal of Plastic, Reconstructive and Aesthetic Surgery, 2012. 65(11): p. 1461-1467. | Title |
|  | Chong LY, Head K, Hopkins C, Philpott C, Schilder AG, Burton MJ. Intranasal steroids versus placebo or no intervention for chronic rhinosinusitis. Cochrane Database Syst Rev. 2016;4:Cd011996. | Title |
|  | Choonthar, M.M., et al., Head injury - A maxillofacial surgeon’s perspective. Journal of Clinical and Diagnostic Research, 2016. 10(1): p. ZE01-ZE06. | Title |
|  | Chou AHK, Park JH, Shoaib AM, Lee NK, Lim HJ, Abdulwhab AA, et al. Total maxillary arch distalization with modified C-palatal plates in adolescents: A long-term study using cone-beam computed tomography.Am J Orthod Dentofacial Orthop. 2021;159(4):470-9. | Title |
|  | Chou J, Horati H, Margaroli C, Tiddens HA, Scholte BJ, Chandler JD, et al. Discovery of airway fluid proteins associated with progressive airway disease in young children with cystic fibrosis. Pediatric Pulmonology. 2019;54:328. | Title |
|  | Chou K-T, Su K-C, Hsiao Y-H, Huang S-F, Ko H-K, Tseng C-M, et al. Post-bronchodilator Reversibility of FEV1 and Eosinophilic Airway Inflammation in COPD. Arch bronconeumol (Ed impr). 2017;53(10):547-53. | Title |
|  | Chousangsuntorn K, Bhongmakapat T, Apirakkittikul N, Sungkarat W, Supakul N, Laothamatas J. Computed Tomography Characterization and Comparison With Polysomnography for Obstructive Sleep Apnea Evaluation. Journal of Oral and Maxillofacial Surgery. 2018;76(4):854-72. | Title |
|  | Chousangsuntorn K, Bhongmakapat T, Apirakkittikul N, Sungkarat W, Supakul N, Laothamatas J. Upper Airway Areas, Volumes, and Linear Measurements Determined on Computed Tomography During Different Phases of Respiration Predict the Presence of Severe Obstructive Sleep Apnea. Journal of Oral and Maxillofacial Surgery. 2018;76(7):1524-31. | Title |
|  | Christie KF, Boucher N, Chung CH. Effects of bonded rapid palatal expansion on the transverse dimensions of the maxilla: A cone-beam computed tomography study. American Journal of Orthodontics and Dentofacial Orthopedics. 2010;137(4):S79-S85. | Title |
|  | Chu G, Zhao JM, Han MQ, Mou QN, Ji LL, Zhou H, et al. Three-dimensional prediction of nose morphology in Chinese young adults: a pilot study combining cone-beam computed tomography and 3dMD photogrammetry system. International journal of legal medicine. 2020;134(5):1803‐16. | Title |
|  | Chuang YJ, Hwang SJ, Buhr KA, Miller CA, Avey GD, Story BH, et al. Anatomic development of the upper airway during the first five years of life: A three-dimensional imaging study. PLoS ONE. 2022;17(3 March). | Title |
|  | Chung JJ, Kim MJ, Kim JH, Lee JT, Yoo HS, Kim KW. Imaging findings of giant liposarcoma of the esophagus. Yonsei Med J. 2003;44(4):715-8. | Title |
|  | Chung KL, Huang YH, Lin CH, Fang JP. Novel Bitrate Saving and Fast Coding for Depth Videos in 3D-HEVC. Ieee Transactions on Circuits and Systems for Video Technology. 2016;26(10):1859-69. | Title |
|  | Chung, S. and S. Park, Correction of a long face: Simultaneous reduction of the upper lip using a modified endonasal technique and le fort i osteotomy superior impaction. Journal of Craniofacial Surgery, 2016. 27(8): p. 1937-1942. | Title |
|  | Cicenia J, Sethi S. NAVIGATION TO PERIPHERAL LUNG NODULES USING AN ARTIFICIAL INTELLIGENCE-DRIVEN AUGMENTED IMAGE FUSION PLATFORM (LUNGVISION): A PILOT STUDY. Chest. 2019;156(4):A830. | Title |
|  | Cilingir HZ. The Relationship of Oral Anatomy and Trumpet Performance: Prediction of Physical Talent [D.M.A.]. Ann Arbor: University of Cincinnati; 2012. | Title |
|  | Cillo, J.E. and P.S. Dalton, Traumatic Genial Tubercle Fractures of the Mandible: Airway Concerns and Invasive Management—A Report of 2 Cases. Journal of Oral and Maxillofacial Surgery, 2021. 79(7): p. 1529.e1-1529.e8. | Title |
|  | Ciocca, L., et al., Computer-aided design and manufacturing construction of a surgical template for craniofacial implant positioning to support a definitive nasal prosthesis. Clinical Oral Implants Research, 2011. 22(8): p. 850-856. | Title |
|  | Ciocca, L., et al., New protocol for construction of eyeglasses-supported provisional nasal prosthesis using CAD/CAM techniques. Journal of Rehabilitation Research and Development, 2010. 47(7): p. 595-604. | Title |
|  | Ciric I, Meyers MP, Mayba J, Anthonisen NR. Autopneumonectomy with compensatory lung growth. Canadian Respiratory Journal. 2003;10(5):271-3. | Title |
|  | Ciric, I., et al., Autopneumonectomy with compensatory lung growth. Canadian Respiratory Journal, 2003. 10(5): p. 271-273. |  |
|  | Citir M, Gunduz K, Kasap P. Investigation the anterior mandibular lingual concavity by using cone-beam computed tomography. Folia Morphologica. 2021;80(4):916-22. | Title |
|  | Claes, P., et al., Sexual dimorphism in multiple aspects of 3D facial symmetry and asymmetry defined by spatially dense geometric morphometrics. Journal of Anatomy, 2012. 221(2): p. 97-114. | Title |
|  | **Claudino LV, Mattos CT, Ruellas AC, Sant' Anna EF. Pharyngeal airway characterization in adolescents related to facial skeletal pattern: a preliminary study. Am J Orthod Dentofacial Orthop. 2013;143(6):799-809.** | **Included** |
|  | Claussen C, Lohkamp F, Spenneberg H, Glück E. [Computed tomography of cranio-facial injuries (author's transl)]. Laryngol Rhinol Otol (Stuttg). 1978;57(8):698-705. | Title |
|  | Clément, R., et al., Shape self-regulation in early lung morphogenesis. PLoS ONE, 2012. 7(5). | Title |
|  | Clifford AB, Witmer LM. Case studies in novel narial anatomy: 3. Structure and function of the nasal cavity of saiga (Artiodactyla : Bovidae : Saiga tatarica). Journal of Zoology. 2004;264:217-30. | Title |
|  | Coban G, Yavuz I, Karadas B, Demirbas AE. Three-dimensional assessment of nasal changes after maxillary advancement with impaction using stereophotogrammetry. Korean Journal of Orthodontics. 2020;50(4):249-57. | Title |
|  | Cohen AS, Izzy S, Figueroa SA, Hall CE, Kumar M, McDonagh DL, et al. Variation in priorities in neurocritical care education and training expressed across provider groups. Neurocritical Care. 2015;23(1):S61. | Title |
|  | Cohen O, Betito HR, Adi M, Shapira-Galitz Y, Halperin D, Lahav Y, et al. Development of the nasopharynx: a radiological study of children. Clinical anatomy (New York, NY). 2019. | Title |
|  | Cohen, K.E. and L.P. Hernandez, The complex trophic anatomy of silver carp, Hypophthalmichthys molitrix, highlighting a novel type of epibranchial organ. Journal of Morphology, 2018. 279(11): p. 1615-1628. | Title |
|  | Comyn F, Schwab R, Ratcliffe S, Beothy E, Kim C, Staley B, et al. Craniofacial structures and upper airway soft tissue differences between caucasian and african american females with obstructive sleep apnea. American Journal of Respiratory and Critical Care Medicine. 2010;181(1). | Title |
|  | Condotta I, Brown-Brandl TM, Stinn JP, Rohrer GA, Davis JD, Silva-Miranda KO. DIMENSIONS OF THE MODERN PIG. Transactions of the Asabe. 2018;61(5):1729-39. | Title |
|  | Conith AJ, Kidd MR, Kocher TD, Albertson RC. Ecomorphological divergence and habitat lability in the context of robust patterns of modularity in the cichlid feeding apparatus. Bmc Evolutionary Biology. 2020;20(1). | Title |
|  | Conley, R.S., P.M. Cattaneo, and B.S. Haskell, Characterization of the Upper Airway Morphology and Its Changes in the Apneic Patient Using Cone Beam Computed Tomography, in Cone Beam Computed Tomography in Orthodontics: Indications, Insights, and Innovations. 2014. p. 273-291. | Title |
|  | Consolaro, A., et al., Canines and inflammatory external apical resorption in healthy maxillary lateral incisors due to occlusal trauma: when to detect the position of maxillary canines, to prevent it? Dental Press Journal of Orthodontics, 2022. 27(1). | Title |
|  | Contencin, P., et al., Nasal fossae dimensions in the neonate and young infant: A computed tomographic scan study. Archives of Otolaryngology - Head and Neck Surgery, 1999. 125(7): p. 777-781. | Title |
|  | Coppadoro A, Bellani G, Bronco A, Borsa R, Lucchini A, Bramati S, et al. Measurement of endotracheal tube secretions volume by micro computed tomography (MicroCT) scan: An experimental and clinical study. BMC Anesthesiology. 2014;14. | Title |
|  | Coppadoro A, Bellani G, Bronco A, Eronia N, Barletta A, Teggia Droghi M, et al. Assessment of an endotracheal tube cleaning closed-suctioning system by micro-computed tomography: preliminary clinical data. Critical Care. 2014;18:S114. | Title |
|  | Coppadoro A, Bellani G, Bronco A, Lucchini A, Bramati S, Zambelli V, et al. The use of a novel cleaning closed suction system reduces the volume of secretions within the endotracheal tube as assessed by micro-computed tomography: a randomized clinical trial. Annals of intensive care. 2015;5(1):1‐8. | Title |
|  | Coppadoro A, Bellani G, Mauri T, Borsa R, Meroni V, Barletta A, et al. Quantitative assessment of endotracheal tube (ETT) biof lm by micro-CT scan: Evaluation of the effectiveness of ETT cleaning devices. Critical Care. 2013;17:S58. | Title |
|  | Coppola DM, Craven BA, Seeger J, Weiler E. The effects of naris occlusion on mouse nasal turbinate development. Journal of Experimental Biology. 2014;217(12):2044-52. | Title |
|  | Coquerelle M, Prados-Frutos JC, Rojo R, Drake AG, Murillo-Gonzalez JA, Mitteroecker P. The Fetal Origin of the Human Chin. Evolutionary Biology. 2017;44(3):295-311. | Title |
|  | Corda JV, Shenoy BS, Ahmad KA, Lewis L, K P, Khader SMA, et al. Nasal airflow comparison in neonates, infant and adult nasal cavities using computational fluid dynamics. Comput Methods Programs Biomed. 2022;214:106538. | Title |
|  | Córdoba Juárez S, Lagunes Torres R, Lagunes - Córdoba R, Gómez J. Problemas de anestesia en niños con hendiduras faciales complejas. Reporte de un caso. Rev argent anestesiol. 2010;68(2):183-6. | Title |
|  | Cornelis, M.A. and P.M. Cattaneo, Upper airways: tridimensional analysis and effect of treatment by functional appliances. L' Orthodontie francaise, 2019. 90(3-4): p. 337-342. | Title |
|  | Corona Ruiz MA, Lajud S, Almodovar-Mercado GJ. Patterns of pneumatization and neurovascular structures of the sphenoid sinus among puerto ricans. Otolaryngology - Head and Neck Surgery. 2020;163(1 SUPPL):P300. | Title |
|  | Cortes Dds M, Wallace-Nadolski ME. Non-surgical, upper airway remodeling for skeletal class III and malocclusion with OSA. Sleep. 2017;40:A235-A6. | Title |
|  | Cortes M, Gomez M, Park S, Singh D. A combined approach for upper airway remodeling for skeletal class iii malocclusion with complex OSA. Sleep. 2018;41:A206. | Title |
|  | Cortes M, Gomez M. Non-surgical osseous remodeling and myofunctional therapy for skeletal class iii malocclusion with severe OSA. Sleep. 2018;41:A206-A7. | Title |
|  | Cortes M, Wallace-Nadolski ME, Singh D. Non-surgical, upper airway remodeling for obstructive sleep apnea in adults with craniofacial deficiencies. Sleep. 2016;39:A142. | Title |
|  | Coscarón Blanco E, Blanco García JL, Pérez Liedo MC, Prado Sanjosé MMd, Rodríguez Tejedor A, Criado Martín DA. Quistes epiglóticos: una causa insospechada de disfagia. Estudio anatomoclínico y opciones terapéuticas. An otorrinolaringol Ibero-Am. 2006;33(4):383-9. | Title |
|  | **Costa ED, Brasil DM, Gaêta-Araujo H, Leggitt VL, Roque-Torres GD. Imaging of the upper airway: which method is best for orthodontic assessment? Gen Dent. 2022;70(1):51-5.** | **Included** |
|  | Costa HD, Camara FV, Bezerra FVF, de Moura CEB, Pereira AF, Silva AR, et al. Embryonic/Fetal Development, Placentation and Glycosaminoglycans in the Female Reproductive Tract and Placenta. Acta Scientiae Veterinariae. 2020;48. | Title |
|  | Costa SM, de Jesus AO, Silveira RL, Amaral MBF. Supernumerary nasal tooth removed with a modified maxillary vestibular approach: case report and literature review. Oral and Maxillofacial Surgery-Heidelberg. 2019;23(2):247-52. | Title |
|  | Costa TMdRL, Costa FM, Moreira CA, Rabelo LM, Boguszewski CL, Borba VZC. Sarcopenia in COPD: relationship with COPD severity and prognosis. J bras pneumol. 2015;41(5):415-21. | Title |
|  | Cota R, Goto A. Surgical treatment for dentofacial deformities: class III patient with mandibular laterodeviation and anterior open bite. International Journal of Oral and Maxillofacial Surgery. 2019;48:191. | Title |
|  | Craig JR, Palmer JN, Zhao K. Computational fluid dynamic modeling of nose-to-ceiling head positioning for sphenoid sinus irrigation. International Forum of Allergy and Rhinology. 2017;7(5):474-9. | Title |
|  | Crowe, T.P. and W.H. Hsu, Evaluation of Recent Intranasal Drug Delivery Systems to the Central Nervous System. Pharmaceutics, 2022. 14(3). | Title |
|  | Crucke J, Huysseune A. Unravelling the blood supply to the zebrafish pharyngeal jaws and teeth. Journal of Anatomy. 2013;223(4):399-409. | Title |
|  | Ctri. Evaluation of changes produced by 2 types of appliance to correct the narrow upper jaw. https://trialsearchwhoint/Trial2aspx?TrialID=CTRI/2019/10/021705. 2019. | Title |
|  | Cuccia AM, Carola C. The measurement of craniocervical posture: A simple method to evaluate head position. International Journal of Pediatric Otorhinolaryngology. 2009;73(12):1732-6. | Title |
|  | Cui DM, Han DM, Nicolas B, Hu CL, Wu J, Su MM. Three-Dimensional evaluation of nasal surgery in patients with obstructive sleep apnea. Chinese Medical Journal. 2016;129(6):651-6. | Title |
|  | Cunha L, Wolford LM, Cevidanes L, Cassano D, Mansur D, Rossi M, et al. TMJ condylar morphology variability in condylar resection/total joint prosthesis patients. Journal of Oral and Maxillofacial Surgery. 2012;70(9):e94-e5. | Title |
|  | Curic M, Janc D, Vujovic D, Vuckovic V. The effects of a river valley on an isolated cumulonimbus cloud development. Atmospheric Research. 2003;66(1-2):123-39. | Title |
|  | Curtis AA, Simmons NB. Unique Turbinal Morphology in Horseshoe Bats (Chiroptera: Rhinolophidae). Anatomical Record-Advances in Integrative Anatomy and Evolutionary Biology. 2017;300(2):309-25. | Title |
|  | Cusmano F, Pedrazzini M, Uccelli M, Ferrozzi F, Armaroli S, Mineo F, et al. [Spiral CT in maxillo-facial trauma]. Acta Biomed Ateneo Parmense. 2000;71(6):291-8. | Title |
|  | Cvrček, J., et al., Familial occurrence of skeletal developmental anomalies as a reflection of biological relationships in a genealogically documented Central European sample (19th to 20th centuries). Journal of Anatomy, 2021. 239(5): p. 1226-1238. | Title |
|  | D' Oleo-Aracena, M.F., et al., Skeletal and dentoalveolar bilateral dimensions in unilateral palatally impacted canine using cone beam computed tomography. Progress in Orthodontics, 2017. 18(1). | Title |
|  | D’agostino, A., et al., Zygomatic implants supported rehabilitations in atrophic maxilla: Sinus complications, radiological, periodontal and prosthodontic evaluations: A one year follow up retrospective longitudinal study. Journal of Biological Regulators and Homeostatic Agents, 2020. 34(6): p. 89-100. | Title |
|  | da Costa AL, Manica D, Manzini M, Bernardi A, Perez JA, Sekine L, et al. Maxillary Hypoplasia: Differential Diagnosis of Nasal Obstruction in Infants. Journal of Craniofacial Surgery. 2017;28(7):E697-E700. | Title |
|  | da Silva Machado V, Cantharino de Carvalho BA, Vedovello SAS, Valdrighi H, Santamaria Júnior M. Pharyngeal Airway Modifications in Skeletal Class III Patients Undergoing Bimaxillary Advancement Surgery. Journal of Oral and Maxillofacial Surgery. 2019;77(10):2126.e1-.e8. | Title |
|  | Da Silveira AC, Martinez O, Da Silveira D, Daw JL, Cohen M. Three-dimensional technology for documentation and record keeping for patients with facial clefts. Clinics in Plastic Surgery. 2004;31(2):141-+. | Title |
|  | Dadgar-Yeganeh A, Hatcher DC, Oberoi S. Association between degenerative temporomandibular joint disorders, vertical facial growth, and airway dimension. Journal of the World Federation of Orthodontists. 2021;10(1):20-8. | Title |
|  | Dagistan, S., et al., Retrospective morphometric analysis of the infraorbital foramen with cone beam computed tomography. Nigerian Journal of Clinical Practice, 2017. 20(9): p. 1053-1064. | Title |
|  | Dai Y, Sowjanya M, You J, Xu K. Non-Hodgkin's Lymphoma of Multiple Skeletal Muscles Involvement Seen on FDG PET/CT Scans. Medicine (Baltimore). 2015;94(18):e833. | Title |
|  | Daisne JF, Installé J, Bihin B, Laloux M, Vander Borght T, Mathieu I, et al. SPECT/CT lymphoscintigraphy for superselective nodal CTV selection in cN0 HNSCC patients: A phase I study. Radiotherapy and Oncology. 2014;111:S7. | Title |
|  | Dalla Torre D, Burtscher D, Widmann G, Rasse M, Puelacher T, Puelacher W. Long-term influence of mandibular advancement on the volume of the posterior airway in skeletal Class II-patients: a retrospective analysis. British Journal of Oral and Maxillofacial Surgery. 2017;55(8):780-6. | Title |
|  | Dalmau E, Zamora N, Tarazona B, Gandia JL, Paredes V. A comparative study of the pharyngeal airway space, measured with cone beam computed tomography, between patients with different craniofacial morphologies. Journal of Cranio-Maxillofacial Surgery. 2015;43(8):1438-46. | Full text  Other outcomes |
|  | Damade, C., et al., Anterior Cervical Idiopathic Hyperostosis and Dysphagia: The Impact of Surgical Management—Study of a Series of 11 Cases. World Neurosurgery, 2020. 138: p. e305-e310. | Title |
|  | Damian S, Gunther N, Smithhisler MR, Klarmann GJ. An air-liquid interface culture system for small airway epithelial cells. American Journal of Respiratory and Critical Care Medicine. 2011;183(1). | Title |
|  | Danahay H, Jaffe AB. High throughput culture of normal and CF primary bronchial epithelial cells. Pediatric Pulmonology. 2011;46:276. | Title |
|  | Danahay H. et al. Notch2 is required for inflammatory cytokine-driven goblet cell metaplasia in the lung. Cell Reports 2015. 10(2): p. 239-252. | Title |
|  | D'Andrea, E. and E. Barbaix, Anatomic research on the perioral muscles, functional matrix of the maxillary and mandibular bones. Surgical and Radiologic Anatomy, 2006. 28(3): p. 261-266. | Title |
|  | Dang, J., K. Honda, and H. Suzuki, Morphological and acoustical analysis of the nasal and the paranasal cavities. Journal of the Acoustical Society of America, 1994. 96(4): p. 2088-2100. | Title |
|  | Daniel, R.K., The nasal tip: Anatomy and aesthetics. Plastic and Reconstructive Surgery, 1992. 89(2): p. 216-224. | Title |
|  | Danner RM, Gulson-Castillo ER, James HF, Dzielski SA, Frank DC, Sibbald ET, et al. Habitat-specific divergence of air conditioning structures in bird bills. Auk. 2017;134(1):65-75. | Title |
|  | D'Apolito W, Dapolito I. Impacted supernumerary tooth: case report. Dental Cadmos. 2019;87(6):384-9. | Title |
|  | Daraze A, Delatte M, Liistro G, Majzoub Z. Cephalometrics of Pharyngeal Airway Space in Lebanese Adults. International Journal of Dentistry. 2017;2017. | Title |
|  | Darsey, D.M., et al., Does hyrax expansion therapy affect maxillary sinus volume? A cone-beam computed tomography report. Imaging Science in Dentistry, 2012. 42(2): p. 83-88. | Title |
|  | Darwaiz T, Pasch B, Riede T. Postnatal remodeling of the laryngeal airway removes body size dependency of spectral features for ultrasonic whistling in laboratory mice. Journal of Zoology. 2022. | Title |
|  | Das, U.M. and J.P. Beena, Effectiveness of circumoral muscle exercises in the developing dentofacial morphology in adenotonsillectomized children: An ultrasonographic evaluation. Journal of Indian Society of Pedodontics and Preventive Dentistry, 2009. 27(2): p. 94-103. | Title |
|  | Dastan F, Ghaffari H, Shishvan HH, Zareiyan M, Akhlaghian M, Shahab S. Correlation between the upper airway volume and the hyoid bone position, palatal depth, nasal septum deviation, and concha bullosa in different types of malocclusion: A retrospective cone-beam computed tomography study. Dental and Medical Problems. 2021;58(4):509-14. | Title |
|  | d'Athis, F. and J.E. de la Coussaye, Humidification of inhaled anaesthetic gases. Annales francaises d'anesthesie et de reanimation, 1988. 7(5): p. 393-400. | Title |
|  | Dave MH, Kemper M, Schmidt AR, Both CP, Weiss M. Pediatric airway dimensions-A summary and presentation of existing data. Pediatric Anesthesia. 2019;29(8):782-9. | Title |
|  | Dave MH, Schmid K, Weiss M. Airway dimensions from fetal life to adolescence. A literature overview. Pediatric Pulmonology. 2018;53(8):1140-6. | Title |
|  | David, D.J., et al., Nasendoscopy: Significant refinements of a direct-viewing technique of the velopharyngeal sphincter. Plastic and Reconstructive Surgery, 1982. 70(4): p. 423-428. | Title |
|  | Davidson, E. and A.R. Kumar, A preliminary three-dimensional analysis of nasal aesthetics following le fort i advancement in patients with cleft lip and palate. Journal of Craniofacial Surgery, 2015. 26(7): p. e629-e633. | Title |
|  | Davies JC. Invasive and non-invasive methods to assess responses to respiratory treatments in early childhood. Pediatric Pulmonology. 2017;52:S20-S2. | Title |
|  | Davy-Jow, S.L., S.J. Decker, and J.M. Ford, A simple method of nose tip shape validation for facial approximation. Forensic Science International, 2012. 214(1-3): p. 208.e1-208.e3. | Title |
|  | de Almeida AM, Ozawa TO, Alves ACD, Janson G, Lauris JRP, Ioshida MSY, et al. Slow versus rapid maxillary expansion in bilateral cleft lip and palate: a CBCT randomized clinical trial. Clinical Oral Investigations. 2017;21(5):1789-99. | Title |
|  | de Almeida IG, Jr., Kuratani DK, Gomes LM, Fiegenbaum M, Estima Correia EP, Gazzola Zen PR, et al. Nasal fistula, epidermal cyst and hypernatremia in a girl presenting holoprosencephaly due to a rare ZIC2 point mutation. Eur J Med Genet. 2020;63(2):103641. | Title |
|  | de Azevedo S, Gonzalez MF, Cintas C, Ramallo V, Quinto-Sanchez M, Marquez F, et al. Nasal airflow simulations suggest convergent adaptation in Neanderthals and modern humans. Proceedings of the National Academy of Sciences of the United States of America. 2017;114(47):12442-7. | Title |
|  | de Carvalho MF, Vieira JNM, Figueiredo R, Reher P, Chrcanovic BR, Chaves M. Validity of computed tomography in diagnosing midfacial fractures. Int J Oral Maxillofac Surg. 2021;50(4):471-6. | Title |
|  | De Felippe NLO, Bhushan N, Da Silveira AC, Viana G, Smith B. Long-term effects of orthodontic therapy on the maxillary dental arch and nasal cavity. American Journal of Orthodontics and Dentofacial Orthopedics. 2009;136(4). | Title |
|  | De Greef S, Claes P, Mollemans W, Vandermeulen D, Suetens P, Willems G. Computer-assisted facial reconstruction: recent developments and trends. Rev Belge Med Dent (1984). 2005;60(3):237-49. | Title |
|  | De Greef S, Willems G. Three-dimensional cranio-facial reconstruction in forensic identification: Latest progress and new tendencies in the 21st century. Journal of Forensic Sciences. 2005;50(1):12-7. | Title |
|  | De Greef, S. and G. Willems, Three-dimensional cranio-facial reconstruction in forensic identification: Latest progress and new tendencies in the 21st century. Journal of Forensic Sciences, 2005. 50(1): p. 12-17. | Title |
|  | De Greef, S., et al., Computer-assisted facial reconstruction: recent developments and trends. Revue belge de médecine dentaire. Belgisch tijdschrift voor tandheelkunde, 2005. 60(3): p. 237-249. | Title |
|  | de la Hoz RE, Liu X, Doucette JT, Reeves AP, Bienenfeld LA, Wisnivesky JP, et al. Increased Airway Wall Thickness is Associated with Adverse Longitudinal First–Second Forced Expiratory Volume Trajectories of Former World Trade Center workers. Lung. 2018;196(4):481-9. | Title |
|  | de Medeiros-Santana, M.N.L., et al., Predictors of Velopharyngeal Dysfunction in Individuals With Cleft Palate Following Surgical Maxillary Advancement: Clinical and Tomographic Assessments. Cleft Palate-Craniofacial Journal, 2019. 56(10): p. 1314-1321. | Title |
|  | de Menezes LM, de Oliveira RB, Weissheimer A, Avelar RL. Midfacial Protraction With Skeletal Anchorage After Pterygomaxillary Separation. J Craniofac Surg. 2016;27(6):1561-4. | Title |
|  | De Moraes, M.E.L., et al., Evaluating craniofacial asymmetry with digital cephalometric images and cone-beam computed tomography. American Journal of Orthodontics and Dentofacial Orthopedics, 2011. 139(6): p. e523-e531. | Title |
|  | De Nunzio, G., et al. An innovative lung segmentation algorithm in CT images with accurate delimitation of the hilus pulmonis. in IEEE Nuclear Science Symposium Conference Record. 2008. | Title |
|  | de Oliveira I, Costa S, Pinheiro R, Freitas B, Reher P, Rodrigues V. Oropharyngeal complex differences related to sex and occlusal factors in adolescents aged 12-17 years. Cranio-the Journal of Craniomandibular & Sleep Practice. | Full text  2D lat. cephalometry |
|  | de Paula LK, Ackerman JL, Carvalho FDR, Eidson L, Cevidanese LHS. Digital live-tracking 3-dimensional minisensors for recording head orientation during image acquisition. American Journal of Orthodontics and Dentofacial Orthopedics. 2012;141(1):116-23. | Title |
|  | De Rezende Bastos I, Torres MLCP. Supernumerary tooth in nasal fossa. Int Archives of Otorhinolaryngology.2015;19:S52-S3. | Title |
|  | De Sousa Miranda, W., et al., Three-dimensional evaluation of superior airway space after orthognathic surgery with counterclockwise rotation and advancement of the maxillomandibular complex in Class II patients. Oral Surgery, Oral Medicine, Oral Pathology and Oral Radiology, 2015. 120(4): p. 453-458. | Title |
|  | De Souza Carvalho ACG, Magro Filho O, Garcia IR, Araujo PM, Nogueira RLM. Cephalometric and three-dimensional assessment of superior posterior airway space after maxillomandibular advancement. International Journal of Oral and Maxillofacial Surgery. 2012;41(9):1102-11. | Title |
|  | De Souza Mello L, Bak R, Nizzo N, Resende LO, Machado RS. Nasolabial cyst. International Archives of Otorhinolaryngology. 2015;19:S26. | Title |
|  | de Torres HM, Evangelista K, de Torres EM, Estrela C, Figueiredo PTD, Valladares-Neto J, et al. Comparison of Dimensions of the Nasopharynx and Oropharynx Using Different Anatomical References: Is There Equivalence? Journal of Oral and Maxillofacial Surgery. 2019;77(12):2545-54. | Abstract |
|  | De Troyer, A., et al., Mechanical advantage of the human parasternal intercostal and triangularis sterni muscles. Journal of Physiology, 1998. 513(3): p. 915-925. | Title |
|  | De Water VR, Saridin JK, Bouw F, Murawska MM, Koudstaal MJ. Measuring upper airway volume: Accuracy and reliability of dolphin 3d software compared to manual segmentation in craniosynostosis patients. Journal of Oral and Maxillofacial Surgery. 2014;72(1):139-44. | Title |
|  | Deall CE, Kornmann NSS, Bella H, Wallis KL, Hardwicke JT, Su TL, et al. Facial Aesthetic Outcomes of Cleft Surgery: Assessment of Discrete Lip and Nose Images Compared with Digital Symmetry Analysis. Plast Reconstr Surg. 2016;138(4):855-62. | Title |
|  | Dearden RP, Mansuit R, Cuckovic A, Herrel A, Didier D, Tafforeau P, et al. The morphology and evolution of chondrichthyan cranial muscles: A digital dissection of the elephantfish Callorhinchus milii and the catshark Scyliorhinus canicula. Journal of Anatomy. 2021;238(5):1082-105. | Title |
|  | Dearden RP, Stockey C, Brazeau MD. The pharynx of the stem-chondrichthyan Ptomacanthus and the early evolution of the gnathostome gill skeleton. Nature Communications. 2019;10. | Title |
|  | deBruin PF, Ueki J, Watson A, Pride NB. Size and strength of the respiratory and quadriceps muscles in patients with chronic asthma. European Respiratory Journal. 1997;10(1):59-64. | Title |
|  | Deeb W, Hansen L, Hotan T, Hietschold V, Harzer W, Tausche E. Changes in nasal volume after surgically assisted bone-borne rapid maxillary expansion. American Journal of Orthodontics and Dentofacial Orthopedics. 2010;137(6):782-9. | Title |
|  | Degerliyurt K, Ueki K, Hashiba Y, Marukawa K, Nakagawa K, Yamamoto E. A comparative CT evaluation of pharyngeal airway changes in class III patients receiving bimaxillary surgery or mandibular setback surgery. Oral Surg Oral Med Oral Pathol Oral Radiol Endod. 2008;105(4):495-502. | Title |
|  | Degerliyurt K, Ueki K, Hashiba Y, Marukawa K, Simsek B, Okabe K, et al. The effect of mandibular setback or two-jaws surgery on pharyngeal airway among different genders. International Journal of Oral and Maxillofacial Surgery. 2009;38(6):647-52. | Title |
|  | del Castillo DL, Flores DA, Cappozzo HL. Ontogenetic Development and Sexual Dimorphism of Franciscana Dolphin Skull: A 3D Geometric Morphometric Approach. Journal of Morphology. 2014;275(12):1366-75. | Title |
|  | del Pilar ON, Garay MJG, Chen JC. Three-axis classification of mouse lung mesenchymal cells reveals two populations of myofibroblasts. Development. 2022;149(6). | Title |
|  | Deleon VB, Smith TD. Mapping the Nasal Airways: Using Histology to Enhance CT-Based Three-Dimensional Reconstruction in Nycticebus. Anatomical Record-Advances in Integrative Anatomy and Evolutionary Biology. 2014;297(11):2113-20. | Title |
|  | Deleu M, Dagassan D, Berg I, Bize J, Dula K, Lenoir V, et al. Establishment of national diagnostic reference levels in dental cone beam computed tomography in Switzerland. Dentomaxillofacial Radiology. 2020;49(6). | Title |
|  | Dellavia C, Catti F, Sforza C, Grandi G, Ferrario VF. Non-invasive longitudinal assessment of facial growth in children and adolescents with hypohidrotic ectodermal dysplasia. European Journal of Oral Sciences. 2008;116(4):305-11. | Title |
|  | Dellavia, C., et al., Facial anthropometry in hypohidrotic ectodermal dysplasia (HED), in Handbook of Anthropometry: Physical Measures of Human Form in Health and Disease. 2012. p. 1585-1602. | Title |
|  | Demetriades, D., et al., In Brief. Current Problems in Surgery, 2007. 44(1): p. 6-10. | Title |
|  | Demirbas, A.E., et al., Is Ultrasonic Bone Scalpel Useful in Le Fort I Osteotomy? Journal of Oral and Maxillofacial Surgery, 2020. 78(1): p. 141.e1-141.e10. | Title |
|  | Demirtas O, Kalabalik F, Dane A, Aktan AM, Ciftci E, Tarim E. Does Unilateral Cleft Lip and Palate Affect the Maxillary Sinus Volume? Cleft Palate-Craniofacial Journal. 2018;55(2):168-72. | Title |
|  | Den Dekker H, Schipper S, De Jongste J, Reiss I, Hofman A, Jaddoe V, et al. Fetal and infant growth and childhood lung function. European Respiratory Journal. 2015;46. | Title |
|  | Denadai R, Chou PY, Seo HJ, Lonic D, Lin HH, Pai BCJ, et al. Patient- and 3D morphometry-based nose outcomes after skeletofacial reconstruction. Scientific Reports. 2020;10(1). | Title |
|  | Denadai R, Chou PY, Yao CF, Chen YA, Lin YY, Huang CS, et al. Effect of Le Fort I Maxillary Repositioning on Three-Dimensional Nasal Tip Rotation: A Comparative Study with Implication for the Asian Nose. Plast Reconstr Surg. 2021;147(4):903-14. | Title |
|  | Denadai, R., et al., Type of maxillary segment mobilization affects three-dimensional nasal morphology. Journal of Plastic, Reconstructive and Aesthetic Surgery, 2021. 74(3): p. 592-604. | Title |
|  | Deng L, Jin Y, Luo M. Micro-curvature environment is a determinant of airway smooth muscle cell orientation and stiffness during early pattern formation in culture. American Journal of Respiratory and Critical Care Medicine. 2017;195. | Title |
|  | Deng, L., H. He, and N. Peter, 2-D and 3-D evaluation of upper airway changes after maxillary expansion with the Alt-RAMEC protocol. Medical Journal of Wuhan University, 2016. 37(6): p. 960-964. | Title |
|  | Dentino KM, Sierra-Vasquez D, Padwa BL. Inferior Turbinate Asymmetry Is a Feature of the Unilateral Complete Cleft Lip and Palate Nasal Deformity. Journal of Oral and Maxillofacial Surgery. 2016;74(4):797-803. | Title |
|  | Denzer W, Dondorp E, Koppetsch T, Lahaise K, Manthey U, Mostadius M, et al. Nomenclatural and morphological notes on the rare agamid lizard Pseudocophotis sumatrana (Hubrecht, 1879) ( Squamata: Agamidae: Draconinae). Raffles Bulletin of Zoology. 2021;69:448-62. | Title |
|  | Deuse T, Blankenberg F, Haddad M, Reichenspurner H, Phillips N, Robbins RC, et al. Mechanisms behind Local Immunosuppression Using Inhaled Tacrolimus in Preclinical Models of Lung Transplantation. American Journal of Respiratory Cell and Molecular Biology. 2010;43(4):403-12. | Title |
|  | Dewo E. Multiple congenital anomalies survivor. What are the problems? Pediatric Critical Care Medicine. 2014;15(4):37. | Title |
|  | Dey JK, Recker CA, Olson MD, Bowen AJ, Hamilton GS, 3rd. Predicting Nasal Soft Tissue Envelope Thickness for Rhinoplasty: A Model Based on Visual Examination of the Nose. Ann Otol Rhinol Laryngol. 2021;130(1):60-6. | Title |
|  | Dey JK, Recker CA, Olson MD, Bowen AJ, Panda A, Kostandy PM, et al. Assessing Nasal Soft-Tissue Envelope Thickness for Rhinoplasty: Normative Data and a Predictive Algorithm. JAMA Facial Plast Surg. 2019;21(6):511-7. | Title |
|  | Dhaifalah I, Curtisova V, Santavy J. Prenatal diagnosis of monocephalic bifacial tetraophthalmic diprosopus (conjoined twin). Fetal Diagnosis and Therapy. 2008;23(1):82-6. | Title |
|  | Dhasmana, A., A. Singh, and S. Rawal, Biomedical grafts for tracheal tissue repairing and regeneration “Tracheal tissue engineering: an overview”. Journal of Tissue Engineering and Regenerative Medicine, 2020. 14(5): p. 653-672. | Title |
|  | Di Carlo G, Gili T, Caldarelli G, Polimeni A, Cattaneo PM. A community detection analysis of malocclusion classes from orthodontics and upper airway data. Orthodontics & Craniofacial Research. 2021;24:172-80. | Title |
|  | Di Carlo G, Gurani SF, Pinholt EM, Cattaneo PM. A new simple three-dimensional method to characterize upper airway in orthognathic surgery patient. Dentomaxillofac Radiol. 2017;46(8):20170042. | Title |
|  | **Di Carlo G, Polimeni A, Melsen B, Cattaneo PM. The relationship between upper airways and craniofacial morphology studied in 3D. A CBCT study. Orthod Craniofac Res. 2015;18(1):1-11.** | **Included** |
|  | Di Carlo G, Saccucci M, Ierardo G, Luzzi V, Occasi F, Zicari AM, et al. Rapid Maxillary Expansion and Upper Airway Morphology: A Systematic Review on the Role of Cone Beam Computed Tomography. BioMed Research International. 2017;2017. | Title |
|  | Di Girolamo S, Di Girolamo M, Giacomini PG, Ferraro S, Izzo R. Unusual evolution of a residual dental cyst: a giant rhinolith. Cranio. 2008;26(4):294-7. | Title |
|  | Di Grezia G, Gatta G, Vecchione L, Vitiello G, D'Anna E, Dell'Aquila A. Computed tomography for the diagnosis and management of idiopathic cervical spine hyperostosis. Gazzetta Medica Italiana Archivio per le Scienze Mediche. 2017;176(11):592-5. | Title |
|  | Di Pasquale L. The use of radiographic markers for determining natural head position in virtual surgical planning. Journal of Oral and Maxillofacial Surgery. 2017;75(10):e385-e6. | Title |
|  | Diac MM, Earar K, Damian SI, Knieling A, Iov T, Shrimpton S, et al. Facial Reconstruction: Anthropometric Studies Regarding the Morphology of the Nose for Romanian Adult Population I: Nose Width. Applied Sciences-Basel. 2020;10(18). | Title |
|  | DiCosimo C, Alsulaiman AA, Shah C, Motro M, Will LA, Parsi GK. Analysis of nasal airway symmetry and upper airway changes after rapid maxillary expansion. American Journal of Orthodontics and Dentofacial Orthopedics. 2021;160(5):695-704. | Title |
|  | Dief S, Veitz-Keenan A, Amintavakoli N, McGowan R. A systematic review on incidental findings in cone beam computed tomography (CBCT) scans. Dentomaxillofac Radiol. 2019;48(7):20180396. | Title |
|  | Diehm, M., et al., Bilateral femoral capital physeal fractures in an adult cat with suspected congenital primary hypothyroidism. Tierarztliche Praxis Ausgabe K: Kleintiere - Heimtiere, 2019. 47(1): p. 48-54. | Title |
|  | Diewert, V.M., A quantitative coronal plane evaluation of craniofacial growth and spatial relations during secondary palate development in the rat. Archives of Oral Biology, 1978. 23(8): p. 607-629. | Title |
|  | Dilaver E, Ak KB, Suzen M, Altin G, Uckan S. Evaluation of Internal Nasal Valve Using Computed Tomography After Le Fort I Osteotomy: A Cross-Sectional Study from a Tertiary Center. Haseki Tip Bulteni-Medical Bulletin of Haseki. 2021;59(5):400-4. | Title |
|  | DiLuzio KG, Sittitavornwong S, Mostofi P. Can Positioning an Oral Endotracheal Tube in the Retromolar Space Allow Maxillomandibular Fixation without Occlusal Interference? Journal of Oral and Maxillofacial Surgery. 2021;79(10):e68-e9. | Title |
|  | Dindaroğlu F, Duran GS, Görgülü S. Effects of rapid maxillary expansion on facial soft tissues : deviation analysis on three-dimensional images. Fortschritte der Kieferorthopadie [Journal of orofacial orthopedics]. 2016;77(4):242‐50. | Title |
|  | Dittmar M, Spruss T, Schuierer G, Horn M. External carotid artery territory ischemia impairs outcome in the endovascular filament model of middle cerebral artery occlusion in rats. Stroke. 2003;34(9):2252-7. | Title |
|  | **Diwakar R, Kochhar AS, Gupta H, Kaur H, Sidhu MS, Skountrianos H, et al. Effect of craniofacial morphology on pharyngeal airway volume measured using cone-beam computed tomography (Cbct)—a retrospective pilot study. International Journal of Environmental Research and Public Health. 2021;18(9).** | **Included** |
|  | Diwakar R, Sidhu MS, Jain S, Grover S, Prabhakar M. Three-dimensional evaluation of pharyngeal airway in complete unilateral cleft individuals and normally growing individuals using cone beam computed tomography. Cleft Palate-Craniofacial Journal. 2015;52(3):346-51. | Title |
|  | Djordjevic J, Jadallah M, Zhurov AI, Toma AM, Richmond S. Three-dimensional analysis of facial shape and symmetry in twins using laser surface scanning. Orthodontics & Craniofacial Research. 2013;16(3):146-60. | Title |
|  | Dmitriev MO, Tikholaz VO, Shepitko KV, Shinkaruk-Dykovytska MM, Androshchuk OV, Bobruk SV, et al. SEXUAL DIMORPHISM OF NORMATIVE CEPHALOMETRIC PARAMETERS DETERMINED BY THE HOLDAWAY METHOD IN BOYS AND GIRLS OF PODILLIA. World of Medicine and Biology. 2018;64(2):39-43. | Title |
|  | Do NT, Klement KA, Denny AD. Measuring the Position of the Mandibular Foramen in Relation to the Dentoalveolar Plane in Pierre Robin Patients: Establishing Safety of the Horizontal Osteotomy. Journal of Craniofacial Surgery. 2020;31(1):150-3. | Title |
|  | Doğan MS, Callea M, Yavuz Ì, Aksoy O, Clarich G, Günay A, et al. An evaluation of clinical, radiological and three-dimensional dental tomography findings in ectodermal dysplasia cases. Med Oral Patol Oral Cir Bucal. 2015;20(3):e340-6. | Title |
|  | Dolci C, Elamin F, Gibelli DM, Barni L, Scolaro A, Sessa F, et al. Age- and Sex-Related Changes in Labial Dimensions of Sudanese Youngs of Arab Descent: A Three-Dimensional Cross-Sectional Study. Children-Basel. 2021;8(7). | Title |
|  | Doljanski F. The sculpturing role of fibroblast-like cells in morphogenesis. Perspectives in Biology and Medicine. 2004;47(3):339-56. | Title |
|  | Dong Y, Wang Z, Zhang Z, Niu B, Chen P, Zhang P, et al. Artificial Intelligence Algorithm-Based Computed Tomography Images in the Evaluation of the Curative Effect of Enteral Nutrition after Neonatal High Intestinal Obstruction Operation. J Healthc Eng. 2021;2021:7096286. | Title |
|  | Dong Y, Zhao Y, Bai S, Wu G, Wang B. Three-dimensional anthropometric analysis of the Chinese nose. J Plast Reconstr Aesthet Surg. 2010;63(11):1832-9. | Title |
|  | Dong, Y., et al., Three-dimensional anthropometric analysis of chinese faces and its application in evaluating facial deformity. Journal of Oral and Maxillofacial Surgery, 2011. 69(4): p. 1195-1206. | Title |
|  | Doring JH, Bettendorf M, Hoffmann GF, Kolker S, Ziegler A. Misdiagnosis of child abuse-fibrodysplasia ossificans progressiva as a therapy-relevant differential diagnosis in infancy. Neuropediatrics. 2018;49. | Title |
|  | Doroquez, D.B., et al., A high-resolution morphological and ultrastructural map of anterior sensory cilia and glia in Caenorhabditis elegans. eLife, 2014. 2014(3). | Title |
|  | Doruk C, Sokucu O, Bicakci AA, Yilmaz U, Tas F. Comparison of nasal volume changes during rapid maxillary expansion using acoustic rhinometry and computed tomography. European Journal of Orthodontics. 2007;29(3):251-5. | Title |
|  | Dos Inocentes RJM, Marzano-Rodrigues MN, de Espíndola GG, García-Usó M, Yatabe-Ioshida MS, Trindade IEK, et al. Adults With Unilateral Cleft Lip and Palate Present Reduced Internal Nasal Volumes: Findings of a Three-Dimensional Morphometric Assessment in Cone-Beam Computed Tomography Scans. J Craniofac Surg. 2021;32(1):e15-e9. | Title |
|  | Dossani, R.H., et al., Cranium Bifidum Occultum Associated with Hypertelorism Treated with Posterior Vault Reconstruction and Orbital Box Osteotomies: Case Report and Technical Note. World Neurosurgery, 2017. 107: p. 40-46. | Title |
|  | Dragonas, P., et al., One-year implant survival following lateral window sinus augmentation using plasma rich in growth factors (PRGF): A retrospective study. Medicina Oral Patologia Oral y Cirugia Bucal, 2020. 25(4): p. e474-e480. | Title |
|  | Drakatos P, Karkoulias K, Giannitsas K, Kalogeropoulou C, Papapanagiotou N, Lykouras D, et al. Computed tomography cephalometric and upper airway measurements in patients with OSA and erectile dysfunction. Sleep Breath. 2016;20(2):769-76. | Title |
|  | Drake, V.E., N. Rafaels, and J. Kim, Peripheral blood eosinophilia correlates with hyperplastic nasal polyp growth. International Forum of Allergy and Rhinology, 2016. 6(9): p. 926-934. | Title |
|  | Drosen C, Bock NC, von Bremen J, Pancherz H, Ruf S. Long-term effects of Class II Herbst treatment on the pharyngeal airway width. European Journal of Orthodontics. 2018;40(1):82-9. | Title |
|  | Droste, H., High-resolution seismic stratigraphy of the Shu'aiba and Natih formations in the Sultanate of Oman: Implications for Cretaceous epeiric carbonate platform ssytems, in Geological Society Special Publication. 2010. p. 145-162. | Title |
|  | Du FZ, Chen Q, Wang XJ, Guo XP, Wang ZH, Gao L, et al. Long-term facial changes and clinical correlations in patients with treated acromegaly: a cohort study. European Journal of Endocrinology. 2021;184(2):231-41. | Title |
|  | Du Tolt DF, Nortjé C. The maxillae: integrated and applied anatomy relevant to dentistry. Sadj. 2003;58(8):325-30. | Title |
|  | Du W, He DM, Wang YY, Liu HH, Liao CH, Fei W, et al. Upper Airway Changes After Mandibular Setback and/or Advancement Genioplasty in Obese Patients. Journal of Oral and Maxillofacial Surgery. 2017;75(10):2202-10. | Title |
|  | Dua KS, Aadam AA, Merrill JT, Gasparri M. Unusual case of esophageal disruption treated with self-expanding metal esophageal stents. Gastrointestinal Endoscopy. 2012;75(4):AB106-AB7. | Title |
|  | Duan, Y. and Y. Yang, Comparison of hybrid flux difference schemes. Jisuan Wuli/Chinese Journal of Computational Physics, 2006. 23(3): p. 355-360. | Title |
|  | Dubus JC, de Blic J, Mezzi K. [The role of the small airways in childhood asthma]. Rev Mal Respir. 2004;21(4 Pt 1):737-42. | Title |
|  | Ducloyer M, Wargny M, Medo C, Gourraud PA, Clement R, Levieux K, et al. The Ogival Palate: A New Risk Marker of Sudden Unexpected Death in Infancy? Frontiers in Pediatrics. 2022;10. | Title |
|  | Dufeau, D.L. and L.M. Witmer, Ontogeny of the middle-ear air-sinus system in alligator mississippiensis (archosauria: Crocodylia). PLoS ONE, 2015. 10(9). | Title |
|  | Duffy S, Noar JH, Evans RD, Sanders F. Three-dimensional analysis of the child cleft face. Cleft Palate-Craniofacial Journal. 2000;37(2):137-44. | Title |
|  | Dumont ER, Samadevam K, Grosse I, Warsi OM, Baird B, Davalos LM. SELECTION FOR MECHANICAL ADVANTAGE UNDERLIES MULTIPLE CRANIAL OPTIMA IN NEW WORLD LEAF-NOSED BATS. Evolution. 2014;68(5):1436-49. | Title |
|  | Durigan JLQ, Peviani SM, Russo TL, Silva ACD, Vieira RP, Martins MA, et al. Effects of exercise training on atrophy gene expression in skeletal muscle of mice with chronic allergic lung inflammation. Braz j med biol res. 2009;42(4):339-45. | Title |
|  | Durmaz A, Fernandez-Miranda J, Snyderman CH, Rivera-Serrano C, Tosun F. Prevertebral Corridor: Posterior Pathway for Reconstruction of the Ventral Skull Base. Journal of Craniofacial Surgery. 2011;22(3):848-53. | Title |
|  | Durmaz, C.D., et al., Bilateral choanal atresia in an adult woman with pycnodysostosis. Congenital Anomalies, 2017. 57(3): p. 91-92. | Title |
|  | Dusick A. Investigation and Management of Dysphagia. Seminars in Pediatric Neurology. 2003;10(4):255-64. | Title |
|  | Duskova M, Kotova M, Sedlackova K, Leamerova E, Horak J. Bone reconstruction of the maxillary alveolus for subsequent insertion of a dental implant in patients with cleft lip and palate. Journal of Craniofacial Surgery. 2007;18(3):630-8. | Title |
|  | Dutta D, Heo I, O'Connor R. Studying Cryptosporidium Infection in 3D Tissue-derived Human Organoid Culture Systems by Microinjection. Jove-Journal of Visualized Experiments. 2019(151). | Title |
|  | Dutta R, Spence B, Wei X, Dhapare S, Hindle M, Longest PW. CFD Guided Optimization of Nose-to-Lung Aerosol Delivery in Adults: Effects of Inhalation Waveforms and Synchronized Aerosol Delivery. Pharmaceutical Research. 2020;37(10). | Title |
|  | Dye, B.R., et al., In vitro generation of human pluripotent stem cell derived lung organoids. eLife, 2015. 2015(4): p. 1-25. | Title |
|  | Eames BF, DeLaurier A, Ullmann B, Huycke TR, Nichols JT, Dowd J, et al. FishFace: interactive atlas of zebrafish craniofacial development at cellular resolution. Bmc Developmental Biology. 2013;13. | Title |
|  | Ebenezer V, Ramalingam B. Clinical outcomes of genioplasty procedures in maxillofacial surgery: A review literature. European Journal of Molecular and Clinical Medicine. 2020;7(3):1866-70. | Title |
|  | Echarri Lobiondo P, Pérez Campoy MA, Echarri J. El paciente ortodóncico en dentición mixta y los trastornos del sueño. Rev Ateneo Argent Odontol. 2019;61(2):13-25. | Title |
|  | Edler, R.J., et al., Quantitative use of photography in orthognathic outcome assessment. British Journal of Oral and Maxillofacial Surgery, 2011. 49(2): p. 121-126. | Title |
|  | Edlich RF, Wish JR, Britt LD, Long WB. An organized approach to trauma care: legacy of R Adams Cowley. J Long Term Eff Med Implants. 2004;14(6):481-511. | Title |
|  | Edwards R, Alsufyani N, Heo G, Flores-Mir C. The frequency and nature of incidental findings in large-field cone beam computed tomography scans of an orthodontic sample. Prog Orthod. 2014;15(1):37. | Title |
|  | Edwards, R., et al., Agreement among orthodontists experienced with cone-beam computed tomography on the need for follow-up and the clinical impact of craniofacial findings from multiplanar and 3-dimensional reconstructed views. American Journal of Orthodontics and Dentofacial Orthopedics, 2015. 148(2): p. 264-273. | Title |
|  | Eggers G. Cone beam computer tomography for paranasal sinus imaging. International Journal of Computer Assisted Radiology and Surgery. 2011;6:S205-S6. | Title |
|  | Eggerstedt M, Rhee J, Buranosky M, Batra PS, Tajudeen BA, Smith RM, et al. Nasal Skin and Soft Tissue Thickness Variation Among Differing Races and Ethnicities: An Objective Radiographic Analysis. Facial Plastic Surgery & Aesthetic Medicine.2020;22(3):188-94. | Title |
|  | Eguren M, Liñán-Duran C, Quezada M, Meneses A, Lagravère M. Midpalatal suture density ratio after rapid maxillary expansion evaluated by cone-beam computed tomography. Am J Orthod Dentofacial Orthop. 2022;161(2):238-47. | Title |
|  | Eichel KA, Ackermann RR. Variation in the nasal cavity of baboon hybrids with implications for late Pleistocene hominins. J Hum Evol. 2016;94:134-45. | Title |
|  | Eiszner J. Big questions for little people: Use of peripheral nerve blocks in patients with skeletal dysplasia. Regional Anesthesia and Pain Medicine. 2015;40(5). | Title |
|  | Ekberg O. [Radiologic evaluation of the pharynx]. Radiologe. 1989;29(6):285-94. | Title |
|  | Ekşioğlu, A.S., et al., Value of strain-wave sonoelastography as an imaging modality in assessment of benign acute myositis in children. Turkish Journal of Medical Sciences, 2021. 51(6): p. 2951-2958. | Title |
|  | **Elagib T, Kyung HM, Hung BQ, Hong M. Assessment of the pharyngealmairway in Korean adolescents according to skeletal pattern, sex, and cervical vertebral maturation: A cross-sectional CBCT study. Kor J Orthod 2022; 52 (5): 345-353** | **Included** |
|  | El Fakiri MM, Lahjaouj M, Roubal M, Mahtar M. Schwannoma of the extracranial portion of the accessory nerve presenting as spinal adenopathy. BMJ Case Rep. 2019;12(5). | Title |
|  | **El H, Palomo JM. An airway study of different maxillary and mandibular sagittal positions. Eur J Orthod. 2013;35(2):262-70.** | **Included** |
|  | El H, Palomo JM. Three-dimensional evaluation of upper airway following rapid maxillary expansion: a CBCT study. Angle Orthod. 2014;84(2):265-73. | Title |
|  | El Haje, O.A., et al., Is it possible to use cross-sectional and vertical facial measurements to establish the shape of the mandibular arch? The journal of contemporary dental practice, 2014. 15(6): p. 735-739. | Title |
|  | **El, H. and J.M. Palomo, Airway volume for different dentofacial skeletal patterns. American Journal of Orthodontics and Dentofacial Orthopedics, 2011. 139(6): p. e511-e521.** | **Included** |
|  | El, H. and J.M. Palomo, Three-dimensional evaluation of upper airway following rapid maxillary expansion A CBCT study. Angle Orthodontist, 2014. 84(2): p. 265-273. | Title |
|  | El-Bialy T, Alhadlaq A. New Therapeutics in Promoting and Modulating Mandibular Growth in Cases with Mandibular Hypoplasia. Biomed Research International. 2013;2013. | Title |
|  | El-Bialy, T., Cone one-beam computed tomography an essential for proper orthodontic diagnosis and treatment planning, in Computed Tomography: New Research. 2013. p. 2412-2446. | Title |
|  | El-Boghdadly K, Onwochei DN, Millhoff B, Ahmad I. The effect of virtual endoscopy on diagnostic accuracy and airway management strategies in patients with head and neck pathology: a prospective cohort study. Can J Anaesth. 2017;64(11):1101-10. | Title |
|  | Elfeky, H.Y. and M.M.S. Fayed, Three-dimensional effects of twin block therapy on pharyngeal airway parameters in Class II malocclusion patients. Journal of the World Federation of Orthodontists, 2015. 4(3): p. 114-119. | Title |
|  | Eliason MJ, Schafer J, Archer B, Capra G. The Impact on Nasal Septal Anatomy and Physiology Following Le Fort I Osteotomy for Orthognathic Surgery. J Craniofac Surg. 2021;32(1):277-81. | Title |
|  | Ellis NA, Miller CT. Dissection and Flat-mounting of the Threespine Stickleback Branchial Skeleton. Jove-Journal of Visualized Experiments. 2016(111). | Title |
|  | Elluru RG, Thompson F, Reece A. Fibroblast Growth Factor 18 Gives Growth and Directional Cues to Airway Cartilage. Laryngoscope. 2009;119(6):1153-65. | Title |
|  | Elshal MG, Ghafar MMA, Makarem OA, Mohamed MA, Refai WM, Ibrahim ME, et al. Three dimensional evaluations of airway changes after maxillary expansion and protraction using modified tandem appliance versus facemask in cleft lip and palate patients. Medical Science. 2020;24(106):4532-40. | Title |
|  | Elshebiny T, Bous R, Withana T, Morcos S, Valiathan M. Accuracy of Three-Dimensional Upper Airway Prediction in Orthognathic Patients Using Dolphin Three-Dimensional Software. J Craniofac Surg. 2020;31(4):1098-100. | Title |
|  | Elshebiny T, Morcos S, Abdulaziz M, Quereshy F, Valiathan M. Accuracy of 3-dimensional (3D) soft tissue prediction in nonsyndromic orthognathic cases using dolphin 3D software. Cleft Palate-Craniofacial Journal. 2018;55(1):14. | Title |
|  | Elshebiny T, Morcos S, Mohammad A, Quereshy F, Valiathan M. Accuracy of Three-Dimensional Soft Tissue Prediction in Orthognathic Cases Using Dolphin Three-Dimensional Software. J Craniofac Surg. 2019;30(2):525-8. | Title |
|  | El-Torky, I.R., et al., Using ultrasound technique to quantify the effect of prosthetic palatal plate on tongue position in cleft palate infants. Life Science Journal, 2013. 10(2): p. 1322-1326. | Title |
|  | Emami E, Nguyen PT, Almeida FR, Feine JS, Karp I, Lavigne G, et al. The effect of nocturnal wear of complete dentures on sleep and oral health related quality of life: study protocol for a randomized controlled trial. Trials. 2014;15:358. | Title |
|  | Emura M, Ochiai A, Hirohashi S. In vitro reconstituted tissue as an alternative to human respiratory tract. Toxicology Letters. 1996;88(1-3):81-4. | Title |
|  | Enciso R, Shigeta Y, Nguyen M, Clark GT. Comparison of cone-beam computed tomography incidental findings between patients with moderate/severe obstructive sleep apnea and mild obstructive sleep apnea/healthy patients. Oral Surg Oral Med Oral Pathol Oral Radiol. 2012;114(3):373-81. | Title |
|  | Engboonmeskul, T., N. Leepong, and P. Chalidapongse, Effect of surgical mandibular setback on the occurrence of obstructive sleep apnea. Journal of Oral Biology and Craniofacial Research, 2020. 10(4): p. 597-602. | Title |
|  | Engelhardt JF, Schlossberg H, Yankaskas JR, Dudus L. PROGENITOR CELLS OF THE ADULT HUMAN AIRWAY INVOLVED IN SUBMUCOSAL GLAND DEVELOPMENT. Development. 1995;121(7):2031-46. | Title |
|  | Ennis, J., et al., Congenital Nemaline Myopathy: The Value of Magnetic Resonance Imaging of Muscle. Canadian Journal of Neurological Sciences, 2015. 42(5): p. 338-340. | Title |
|  | Entrenas I, Gonzalez-Chamorro E, Alvarez-Abad C, Muriel J, Menendez-Diaz I, Cobo T. Evaluation of changes in the upper airway after Twin Block treatment in patients with Class II malocclusion. Clinical and Experimental Dental Research. 2019;5(3):259-68. | Title |
|  | Erattakulangara, S. and S.G. Lingala. Airway segmentation in speech MRI using the U-net architecture. in Proceedings - International Symposium on Biomedical Imaging. 2020. | Title |
|  | Erbas B, Kocadereli I. Upper airway changes after Xbow appliance therapy evaluated with cone beam computed tomography. Angle Orthod. 2014;84(4):693-700. | Title |
|  | Ercan I, Etoz A, Guney I, Ocakoglu G, Turan-Ozdemir S, Kan I, et al. Statistical shape analysis of nose in Turkish young adults. Journal of Craniofacial Surgery. 2007;18(1):219-24. | Title |
|  | Erdogan, N., E. Unur, and M. Baykara, CT anatomy of pterygopalatine fossa and its communications: A pictorial review. Computerized Medical Imaging and Graphics, 2003. 27(6): p. 481-487. | Title |
|  | Erdur EA, Yildirim M, Karatas RMC, Akin M. Effects of symmetric and asymmetric rapid maxillary expansion treatments on pharyngeal airway and sinus volume: A cone-beam computed tomography study. Angle Orthodontist. 2020;90(3):425-31. | Title |
|  | Eriksen ES, Gulati S, Moen K, Wisth PJ, Loes S. Apnea-Hypopnea Index in Healthy Class III Patients Treated With Intraoral Vertical Ramus Osteotomy: A Prospective Cohort Study. Journal of Oral and Maxillofacial Surgery. 2019;77(3):582-90. | Title |
|  | Ertaş Ü, Ataol M. Evaluation of Nasal Airway Volume of Operated Unilateral Cleft Lip and Palate Patients Compared With Skeletal Class III Individuals. Cleft Palate Craniofac J. 2019;56(1):15-20. | Title |
|  | Ertekin T, Değermenci M, Nisari M, Unur E, Coşkun A. Age-related changes of nasal cavity and conchae volumes and volume fractions in children: a stereological study. Folia Morphol (Warsz). 2016;75(1):38-47. | Title |
|  | Etoz, M. and Y. Sisman, Evaluation of the nasopalatine canal and variations with cone-beam computed tomography. Surgical and Radiologic Anatomy, 2014. 36(8): p. 805-812. | Title |
|  | Evans CA, Scarfe WC, Ahmad M, Cevidanes LHS, Ludlow JB, Palomo JM, et al. Clinical recommendations regarding use of cone beam computed tomography in orthodontics. Position statement by the American Academy of Oral and Maxillofacial Radiology. Oral Surgery Oral Medicine Oral Pathology Oral Radiology. 2013;116(2):238-57. | Title |
|  | Evans, D.C., R. Ridgely, and L.M. Witmer, Endocranial anatomy of lambeosaurine hadrosaurids (Dinosauria: Ornithischia): A sensorineural perspective on cranial crest function. Anatomical Record, 2009. 292(9): p. 1315-1337. | Title |
|  | Evans, S.S., et al., Validation of the Maxillary Sinus Roof as a Landmark for Navigating the Pediatric Skull Base. Annals of Otology, Rhinology and Laryngology, 2020. 129(1): p. 12-17. | Title |
|  | Evteev AA, Grosheva AN. Nasal cavity and maxillary sinuses form variation among modern humans of Asian descent. American Journal of Physical Anthropology. 2019;169(3):513-25. | Title |
|  | Fairburn SC, Waite PD, Vilos G, Harding SM, Bernreuter W, Cure J, et al. Three-Dimensional Changes in Upper Airways of Patients With Obstructive Sleep Apnea Following Maxillomandibular Advancement. Journal of Oral and Maxillofacial Surgery.2007;65:6-12. | Title |
|  | Fan K, Andrews BT, Liao E, Allam K, do Amaral CAR, Bradley JP. Protection of the Temporomandibular Joint during Syndromic Neonatal Mandibular Distraction using Condylar Unloading. Plastic and Reconstructive Surgery. 2012;129(5):1151-61. | Title |
|  | Fan Y, He W, Chen G, Song GY, Matthews H, Claes P, et al. Facial asymmetry assessment in skeletal Class III patients with spatially-dense geometric morphometrics. European Journal of Orthodontics. 2022;44(2):155-62. | Title |
|  | Fang, C., X.Y. Wang, and D.D. Feng. Airway segmentation for low-contrast CT images from combined PET/CT scanners based on airway modeling and seed prediction. in IFAC Proceedings Volumes (IFAC-PapersOnline). 2009. | Abstract |
|  | Farahmand, K., R. Srinivasan, and M. Hamidi, CFD heat transfer simulation of the human upper respiratory tract for oronasal breathing condition. International Journal of Industrial Engineering Computations, 2012. 3(1 SUPPL): p. 63-70. | Title |
|  | Faria AC, Garcia LV, Santos ACd, Diniz PRB, Ribeiro HT, Mello-Filho FVd. Comparação da área da faringe na vigília e durante o sono induzido em pacientes com Síndrome da Apneia Obstrutiva do Sono (SAOS). Braz j otorhinolaryngol (Impr). 2012;78(1):103-8. | Title |
|  | Faria, A.C., et al., Volumetric analysis of the pharynx in patients with obstructive sleep apnea (OSA) treated with maxillomandibular advancement (MMA). Sleep and Breathing, 2013. 17(1): p. 395-401. | Title |
|  | Fariñas Fuentes G, Fuentes Alcalá ZM, Ortíz Arysmendys I, Sused Beirutty Alayón Y, Cedeño Martínez JA. Evaluación de la saturación arterial de oxígeno durante procedimientos de cirugía bucal en pacientes fumadores. Rev esp cir oral maxilofac. 2019;41(3):138-44. | Title |
|  | Farkas D, Bonasera S, Bass K, Hindle M, Longest PW. Advancement of a Positive-Pressure Dry Powder Inhaler for Children: Use of a Vertical Aerosolization Chamber and Three-Dimensional Rod Array Interface. Pharmaceutical Research. 2020;37(9). | Title |
|  | Farkas D, Thomas ML, Hassan A, Bonasera S, Hindle M, Longest W. Near Elimination of In Vitro Predicted Extrathoracic Aerosol Deposition in Children Using a Spray-Dried Antibiotic Formulation and Pediatric Air-Jet DPI. Pharmaceutical Research. | Title |
|  | Farouk A. Critical choices in cleft surgery: 18-year single-surgeon retrospective review of 900 cases. European Journal of Plastic Surgery. 2016;39(1):11-22. | Title |
|  | Farrell, M.R., et al., Sex-specific effects of early life stress on social interaction and prefrontal cortex dendritic morphology in young rats. Behavioural Brain Research, 2016. 310: p. 119-125. | Title |
|  | Farzal Z, Walsh J, Barbosa GLD, Zdanski CJ, Davis SD, Superfine R, et al. Volumetric Nasal Cavity Analysis in Children With Unilateral and Bilateral Cleft Lip and Palate. Laryngoscope. 2016;126(6):1475-80. | Title |
|  | Farzanegan, F., et al., Evaluation of the relationship between morphology, volume, and density of the mandible and dentofacial vertical dimension using cone beam computed tomography. Pesquisa Brasileira em Odontopediatria e Clinica Integrada, 2019. 19(1). | Title |
|  | Fastuca R, Beccarini T, Rossi O, Zecca PA, Caprioglio A. Influence of facial components in class III malocclusion esthetic perception of orthodontists, patients, and laypersons. Journal of Orofacial Orthopedics-Fortschritte Der Kieferorthopadie. 2022;83(1):48-58. | Title |
|  | Fastuca R, Campobasso A, Zecca PA, Caprioglio A. 3D facial soft tissue changes after rapid maxillary expansion on primary teeth: A randomized clinical trial. Orthodontics & Craniofacial Research. 2018;21(3):140-5. | Title |
|  | Fastuca R, Lorusso P, Lagravere MO, Michelotti A, Portelli M, Zecca PA, et al. Digital evaluation of nasal changes induced by rapid maxillary expansion with different anchorage and appliance design. Bmc Oral Health. 2017;17. | Title |
|  | Fastuca R, Meneghel M, Zecca PA, Mangano F, Antonello M, Nucera R, et al. Multimodal airway evaluation in growing patients after rapid maxillary expansion. European Journal of Paediatric Dentistry. 2015;16(2):129-34. | Title |
|  | Fastuca R, Zecca PA, Caprioglio A. Role of mandibular displacement and airway size in improving breathing after rapid maxillary expansion. Progress in Orthodontics. 2014;15(1):1-7. | Title |
|  | Fastuca, R., et al., Airway compartments volume and oxygen saturation changes after rapid maxillary expansion: A longitudinal correlation study. Angle Orthodontist, 2015. 85(6): p. 955-961. | Title |
|  | Fatouleh, R.H., et al., Reversal of functional changes in the brain associated with obstructive sleep apnoea following 6 months of CPAP. NeuroImage: Clinical, 2015. 7: p. 799-806. | Title |
|  | Fayoux P, Marciniak B, Devisme L, Storme L. Prenatal and early postnatal morphogenesis and growth of human laryngotracheal structures. Journal of Anatomy. 2008;213(2):86-92. | Title |
|  | Feghali J, Khoury E, Souccar NM, Akl R, Ghoubril J. Evaluation of preferred lip position according to different tip rotations of the nose in class I young adult subjects. International Orthodontics. 2019;17(3):478-87. | Title |
|  | Feichtinger M, Zemann W, Mossböck R, Kärcher H. Three-dimensional evaluation of secondary alveolar bone grafting using a 3D- navigation system based on computed tomography: a two-year follow-up. British Journal of Oral and Maxillofacial Surgery. 2008;46(4):278-82. | Title |
|  | Feng X, Todd T, Lintzenich CR, Ding JZ, Carr JJ, Ge YR, et al. Aging-Related Geniohyoid Muscle Atrophy Is Related to Aspiration Status in Healthy Older Adults. Journals of Gerontology Series a-Biological Sciences and Medical Sciences. 2013;68(7):853-60. | Title |
|  | Feng, K., et al., Anatomy of the petrous apex as related to the endoscopic endonasal approach. Journal of Clinical Neuroscience, 2012. 19(12): p. 1695-1698. | Title |
|  | Fenley JdC. Efeitos in vitro e in vivo dos inibidores da protease do HIV Atazanavir e Darunavir nos fatores de virulência de Candida albicans: formação de biofilme, filamentação e infecção em modelo de Galleria mellonella. 2021. p. 53-. | Title |
|  | Fernández Sanromán J, Costas López A, Fernández Ferro M, Arenaz Bua J, López De Sánchez A. Subnasal modified Le Fort I osteotomy: Indications and results. International Journal of Oral and Maxillofacial Surgery. 2013;42(10):1327. | Title |
|  | Fernandez-Ferrer L, Montiel-Company JM, Pinho T, Almerich-Silla JM, Bellot-Arcis C. Effects of mandibular setback surgery on upper airway dimensions and their influence on obstructive sleep apnoea - A systematic review. Journal of Cranio-Maxillofacial Surgery. 2015;43(2):248-53. | Title |
|  | Fernandez-Gago R, Hess M, Gensler H, Rocha F. 3D Reconstruction of the Digestive System in Octopus vulgaris Cuvier, 1797 Embryos and Paralarvae during the First Month of Life. Frontiers in Physiology. 2017;8. | Title |
|  | Ferrari D'Angelo G, Amado Ferreira De Mello A, Rodrigues Genta P, Gebrim E, Lorenzi-Filho G, Schorr F. Abdominal muscle fat infltration and sleep apnea pathogenesis. Sleep Science. 2019;12:51. | Title |
|  | Ferrario VF, Mian F, Peretta R, Rosati R, Sforza C. Three-dimensional computerized anthropometry of the nose: Landmark representation compared to surface analysis. Cleft Palate-Craniofacial Journal. 2007;44(3):278-85. | Title |
|  | Ferrario VF, Sforza C, Ciusa V, Serrao G, Tartaglia GM. Morphometry of the normal human ear: A cross-sectional study from adolescence to mid-adulthood. Journal of Craniofacial Genetics and Developmental Biology. 1999;19(4):226-33. | Title |
|  | Ferrario VF, Sforza C, Dellavia C, Galassi A, Brancaccio D. Abnormal variations in the facial soft tissues of adult uremic patients on chronic dialysis. Angle Orthodontist. 2005;75(3):320-5. | Title |
|  | Ferrario VF, Sforza C, Dellavia C, Tartaglia GM, Sozzi D, Caru A. A quantitative three-dimensional assessment of abnormal variations in facial soft tissues of adult patients with cleft lip and palate. Cleft Palate-Craniofacial Journal. 2003;40(5):544-9. | Title |
|  | Ferrario VF, Sforza C, Dellavia C, Vizzotto L, Caru A. Three-dimensional nasal morphology in cleft lip and palate operated adult patients. Annals of Plastic Surgery. 2003;51(4):390-7. | Title |
|  | Ferrario VF, Sforza C, Poggio CE, Schmitz JH. Craniofacial growth: A three-dimensional soft-tissue study from 6 years to adulthood. Journal of Craniofacial Genetics and Developmental Biology. 1998;18(3):138-49. | Title |
|  | Ferrario VF, Sforza C, Poggio CE, Schmitz JH. Facial volume changes during normal human growth and development. Anatomical Record. 1998;250(4):480-7. | Title |
|  | Ferrario VF, Sforza C, Poggio CE, Schmitz JH. Soft-tissue facial morphometry from 6 years to adulthood: A three-dimensional growth study using a new modeling. Plastic and Reconstructive Surgery. 1999;103(3):768-78. | Title |
|  | Ferrario VF, Sforza C, Poggio CE, Schmitz JH. Three-dimensional study of growth and development of the nose. Cleft Palate-Craniofacial Journal. 1997;34(4):309-17. | Title |
|  | Ferrario VF, Sforza C, Poggio CE, Tartaglia G, Farkas LG. Facial morphometry of television actresses compared with normal women. Journal of Oral and Maxillofacial Surgery. 1995;53(9):1008-15. | Title |
|  | Ferrario VF, Sforza C, Schmitz JH, Santoro F. Three-dimensional facial morphometric assessment of soft tissue changes after orthognathic surgery. Oral Surgery Oral Medicine Oral Pathology Oral Radiology and Endodontics. 1999;88(5):549-56. | Title |
|  | Ferrario VF, Sforza C, Serrao G, Colombo A, Ciusa V. Soft tissue facial growth and development as assessed by the three-dimensional computerized mesh diagram analysis. American Journal of Orthodontics and Dentofacial Orthopedics. 1999;116(2):215-26. | Title |
|  | Ferrario VF, Sforza C, Serrao G, Miani Jr A. A computerized non-invasive method for the assessment of human facial volume. Journal of Cranio-Maxillo-Facial Surgery. 1995;23(5):280-6. | Title |
|  | Ferrario VF, Sforza C, Serrao G. A three-dimensional quantitative analysis of lips in normal young adults. Cleft Palate-Craniofacial Journal. 2000;37(1):48-54. | Title |
|  | Ferreira MG, Dias DR, Cardoso L, Santos M, Sousa CA, Dourado N, et al. Dorsal Hump Reduction Based on the New Ethmoidal Point Classification: A Clinical and Radiological Study of the Keystone Area in 138 Patients. Aesthetic Surgery Journal. 2020;40(9):950-9. | Title |
|  | Ferrell S, Koons C, Mason A. Technique adaptation for transversus abdominis plane blocks in morbidly obese patients. Regional Anesthesia and Pain Medicine. 2015;40(5). | Title |
|  | Ferrier S, Hennocq Q, Leboulanger N, Couloigner V, Denoyelle F, Heuze Y, et al. Nasal cavity shape in unilateral choanal atresia and the role of fetal ventilation in facial growth. Journal of Stomatology Oral and Maxillofacial Surgery. 2020;122(2):135-40. | Title |
|  | Finkelstein Y, Wexler D, Horowitz E, Berger G, Nachmani A, Shapiro-Feinberg M, et al. Frontal and lateral cephalometry in patients with sleep-disordered breathing. Laryngoscope. 2001;111(4 I):634-41. | Title |
|  | Fiorellini, J.P., et al., Healed Edentulous Sites: Suitability for Dental Implant Placement, Need for Secondary Procedures, and Contemporary Implant Designs. International Journal of Oral and Maxillofacial Implants, 2020. 35(5): p. 924-930. | Title |
|  | Fiorentino, E., et al., Digital cineradiology in the diagnosis and surgical treatment of pharyngo-oesophageal junction motor disorders. Chirurgia italiana, 2004. 56(4): p. 495-500. | Title |
|  | **Firwana A, Wang H, Sun L, Wang J, Zhang WB. Relationship of the airway size to the mandible distance in Chinese skeletal Class I and Class II adults with normal vertical facial pattern. Indian J Dent Res. 2019;30(3):368-74.** | **Included** |
|  | Fisher DM, Lo LJ, Chen YR, Noordhoff MS. Three-dimensional computed tomographic analysis of the primary nasal deformity in 3-month-old infants with complete unilateral cleft lip and palate. Plastic and Reconstructive Surgery. 1999;103(7):1826-34. | Title |
|  | Fisher, D.M., et al., Three-dimensional computed tomographic analysis of the primary nasal deformity in 3-month-old infants with complete unilateral cleft lip and palate. Plastic and Reconstructive Surgery, 1999. 103(7): p. 1826-1834. | Title |
|  | Fishman Z, Liu J, Pope J, Fialkov JA, Whyne CM. Validating 3D face morphing towards improving pre-operative planning in facial reconstruction surgery. Computer Methods in Biomechanics and Biomedical Engineering: Imaging and Visualization. 2021;9(5):480-7. | Title |
|  | Fliegauf M, Sonnen AFP, Kremer B, Henneke P. Mucociliary Clearance Defects in a Murine In Vitro Model of Pneumococcal Airway Infection. Plos One. 2013;8(3). | Title |
|  | Flores-Mir C. Cone-beam computerized tomography and the transverse dimension. Seminars in Orthodontics. 2013;19(3):204-9. | Title |
|  | Fluger K, Warunek S, Monegro AF, Al-Jewair T. Mandibular Movement Monitoring During Sleep in Growing Patients Treated with a Functional Orthodontic Mandibular Advancement Appliance. American Journal of Respiratory and Critical Care Medicine. 2022;205(1). | Title |
|  | [Fonseca, Claudia Salvini Barbosa Martins da](https://pesquisa.bvsalud.org/portal/?lang=en&q=au:%22Fonseca,%20Claudia%20Salvini%20Barbosa%20Martins%20da%22); [March, Maria de Fátima Pombo](https://pesquisa.bvsalud.org/portal/?lang=en&q=au:%22March,%20Maria%20de%20F%C3%A1tima%20Pombo%22); [Sant'Anna, Clemax Couto](https://pesquisa.bvsalud.org/portal/?lang=en&q=au:%22Sant%27Anna,%20Clemax%20Couto%22). [Respirador bucal e alterações craniofaciais em alunos de 8 a 10 anos / Mouth breathing and craniofacial alterations in students aged 8 to 10 years](https://pesquisa.bvsalud.org/portal/resource/en/biblio-1380619). [Arq. Asma, Alerg. Imunol](http://portal.revistas.bvs.br/transf.php?xsl=xsl/titles.xsl&xml=http://catserver.bireme.br/cgi-bin/wxis1660.exe/?IsisScript=../cgi-bin/catrevistas/catrevistas.xis|database_name=TITLES|list_type=title|cat_name=ALL|from=1|count=50&lang=pt&comefrom=home&home=false&task=show_magazines&request_made_adv_search=false&lang=pt&show_adv_search=false&help_file=/help_pt.htm&connector=ET&search_exp=Arq.%20Asma,%20Alerg.%20Imunol); 2017, 1(4): 395-402. | Abstract |
|  | Fontanari M, La Greca F, Vellone V, Papoff P, Cascone P. Floating bone phenomenon in pierre robin sequence: from a failure to a new concept. International Journal of Oral and Maxillofacial Surgery. 2019;48:18-9. | Title |
|  | Fornaciari, G., et al., A great enigma of the Italian Renaissance: Paleopathological study on the death of Giovanni dalle Bande Nere (1498-1526) and historical relevance of a leg amputation. BMC Musculoskeletal Disorders, 2014. 15(1). | Title |
|  | Foshang TH, Mestan MA, Riggs LJ. Diffuse idiopathic skeletal hyperostosis: a case of dysphagia. J Manipulative Physiol Ther. 2002;25(1):71-6. | Title |
|  | Foster, A. and N. Holton, Variation in the Developmental and Morphological Interaction Between the Nasal Septum and Facial Skeleton. Anatomical Record, 2016. 299(6): p. 730-740. | Title |
|  | Fraczek M, Guzinski M, Morawska-Kochman M, Nelke KH, Krecicki T. Nasal endoscopy: an adjunct to patient selection for preoperative low-dose CT examination in chronic rhinosinusitis. Dentomaxillofac Radiol. 2016;45(8):20160173. | Title |
|  | Francoz D, Buczinski S, Belanger AM, Forte G, Labrecque O, Tremblay D, et al. Respiratory Pathogens in Quebec Dairy Calves and Their Relationship with Clinical Status, Lung Consolidation, and Average Daily Gain. Journal of Veterinary Internal Medicine. 2015;29(1):381-7. | Title |
|  | Franklin D, Freedman L, Milne N, Oxnard CE. A geometric morphometric study of sexual dimorphism in the crania of indigenous southern Africans. South African Journal of Science. 2006;102(5-6):229-38. | Title |
|  | Franzdóttir SR, Axelsson IT, Arason AJ, Baldursson Ó, Gudjonsson T, Magnusson MK. Airway branching morphogenesis in three dimensional culture. Respiratory Research. 2010;11. | Title |
|  | Fregosi RF. Influence of tongue muscle contraction and transmural pressure on nasopharyngeal geometry in the rat. Journal of Applied Physiology. 2011;111(3):766-74. | Title |
|  | Frey JW, Postigo M, Pitts LR. Endobronchial Valve Placement as Salvage Therapy in the Management of Hemoptysis, A Case Report. American Journal of Respiratory and Critical Care Medicine. 2022;205(1). | Title |
|  | Frey L, Green S, Fabbie P, Hockenbury D, Foran M, Elder K. THE ESSENTIAL ROLE OF THE COM IN THE MANAGEMENT OF SLEEP-DISORDERED BREATHING: A LITERATURE REVIEW AND DISCUSSION. Int J Orofacial Myology. 2014;40:42-55. | Title |
|  | Frey, R., et al., The remarkable vocal anatomy of the koala (Phascolarctos cinereus): insights into low-frequency sound production in a marsupial species. Journal of Anatomy, 2018. 232(4): p. 575-595. | Title |
|  | Friedl, R.M., et al., RDH10 function is necessary for spontaneous fetal mouth movement that facilitates palate shelf elevation. DMM Disease Models and Mechanisms, 2019. 12(7). | Title |
|  | Friedrich RE, Stelljes C, Hagel C, Giese M, Scheuer HA. Dysplasia of the orbit and adjacent bone associated with plexiform neurofibroma and ocular disease in 42 NF-1 patients. Anticancer Res. 2010;30(5):1751-64. | Title |
|  | Friel MT, Starbuck JM, Ghoneima AM, Murage K, Kula KS, Tholpady S, et al. Airway Obstruction and the Unilateral Cleft Lip and Palate Deformity: Contributions by the Bony Septum. Ann Plast Surg. 2015;75(1):37-43. | Title |
|  | Fu Z, Lin YF, Ma L, Li WR. Effects of maxillary protraction therapy on the pharyngeal airway in patients with repaired unilateral cleft lip and palate: A 3-dimensional computed tomographic study. American Journal of Orthodontics and Dentofacial Orthopedics. 2016;149(5):673-82. | Title |
|  | Fu, S., X. Lan, and J. Gao, An anatomical study of the nasolabial fold region. Zhonghua zheng xing shao shang wai ke za zhi = Zhonghua zheng xing shao shang waikf [i.e. waike] zazhi = Chinese journal of plastic surgery and burns / [Chung-hua cheng hsing shao shang wai k'o tsa chih pien chi wei yüan hui pien chi], 1999. 15(2): p. 145-147. | Title |
|  | Fujimoto, M., et al., Temporal correlation between wall shear stress and in-stent stenosis after Wingspan stent in swine model. American Journal of Neuroradiology, 2014. 39(5): p. 994-998. | Title |
|  | Fukase H, Ito T, Ishida H. Geographic Variation in Nasal Cavity form Among Three Human Groups from the Japanese Archipelago: Ecogeographic and Functional Implications. American Journal of Human Biology. 2016;28(3):343-51. | Title |
|  | Fukuyama, T., et al., Transient central diabetes insipidus after cranioplasty for craniosynostosis in an infant with septo-optic dysplasia. Clinical Pediatric Endocrinology, 2022. 31(1): p. 50-53. | Title |
|  | Füllekrug, B., et al., The laryngeal mask airway: Anesthetic gas leakage and fiberoptic control of positioning. Journal of Clinical Anesthesia, 1993. 5(5): p. 357-363. | Title |
|  | g9q RBR. Evaluation of 2 types of maxillary expanders: bone, dental and periodontal changes. https://trialsearchwhoint/Trial2aspx?TrialID=RBR-48g9q6. 2020. | Title |
|  | Gaboriau HP, Kreutziger KL. Penetrating injuries of the face. J La State Med Soc. 1998;150(1):6-9. | Title |
|  | Gaia, B.F., et al., Accuracy and reliability of linear measurements using 3-dimensional computed tomographic imaging software for le Fort i Osteotomy. British Journal of Oral and Maxillofacial Surgery, 2014. 52(3): p. 258-263. | Title |
|  | Galli, J., et al., Biofilm in voice prosthesis: A prospective cohort study and laboratory tests using sonication and SEM analysis. Clinical Otolaryngology, 2018. 43(5): p. 1260-1265. | Title |
|  | Galli-Tsinopoulou, A., et al., A novel variant c.97C&gt;T of the growth hormone releasing hormone receptor gene causes isolated growth hormone deficiency type Ib. JCRPE Journal of Clinical Research in Pediatric Endocrinology, 2018. 10(3): p. 284-288. | Title |
|  | Galvão CP, Tinano MM, Ramos VM, Vidigal BCL, Souki BQ, Becker HMG, et al. Prospective computed tomographic dimensional parameters and mean nasal flow in children with suspected obstructive sleep apnea. Sleep Medicine. 2019;64:S123-S4. | Title |
|  | Galvis LA, Holik AZ, Short KM, Pasquet J, Lun ATL, Blewitt ME, et al. Repression of Igf1 expression by Ezh2 prevents basal cell differentiation in the developing lung. Development. 2015;142(8):1458-69. | Title |
|  | Gandedkar NH, Chng CK, Basheer MA, Chen PY, Yeow VKL. Comparative evaluation of the pharyngeal airway space in unilateral and bilateral cleft lip and palate individuals with noncleft individuals: A cone beam computed tomography study. Cleft Palate-Craniofacial Journal. 2017;54(5):509-16. | Title |
|  | Gandevia SC, Gorman RB, McKenzie DK, Southon FCG. DYNAMIC CHANGES IN HUMAN DIAPHRAGM LENGTH - MAXIMAL INSPIRATORY AND EXPULSIVE EFFORTS STUDIED WITH SEQUENTIAL RADIOGRAPHY. Journal of Physiology-London. 1992;457:167-76. | Title |
|  | Ganjawalla K, Resnick C, Padwa B. 3D CT airway analysis in patients with syndromic craniosynostosis and obstructive sleep apnea. Cleft Palate-Craniofacial Journal. 2017;54(3):e38. | Title |
|  | Gannon AD, Darch SE. Tools for the Real-Time Assessment of a Pseudomonas aeruginosa Infection Model. Jove-Journal of Visualized Experiments. 2021(170). | Title |
|  | Ganske, I.M., et al., Does the Nostril Shape Change After Le Fort I Advancement in Patients With Unilateral Complete Cleft Lip? Journal of Oral and Maxillofacial Surgery, 2020. 78(6): p. 998-1005. | Title |
|  | Gao, X., et al., Effect of titrated mandibular advancement and jaw opening on the upper airway in nonapneic men: A magnetic resonance imaging and cephalometric study. American Journal of Orthodontics and Dentofacial Orthopedics, 2004. 125(2): p. 191-199. | Title |
|  | Garcez AS, Suzuki SS, Storto CJ, Cusmanich KG, Elkenawy I, Moon W. Effects of maxillary skeletal expansion on respiratory function and sport performance in a para-athlete – A case report. Physical Therapy in Sport. 2019;36:70-7. | Title |
|  | Garcia JM. Use of virtual endoscopy in upper airway: Present technical and clinical unknowns for a technique of uncertain future. Radiologia. 1999;41(4):239-45. | Title |
|  | García, J., et al., Calibration of a surgical microscope with automated zoom lenses using an active optical tracker. International Journal of Medical Robotics and Computer Assisted Surgery, 2008. 4(1): p. 87-93. | Title |
|  | García, M., et al., 3D kinematic mandible model to design mandibular advancement devices for the treatment of obstructive sleep apnea. Bio-Design and Manufacturing, 2021. 4(1): p. 22-32. | Title |
|  | Garcia-Martinez D, Bastir M, Villa C, Garcia-Rio F, Torres-Sanchez I, Recheis W, et al. Late subadult ontogeny and adult aging of the human thorax reveals divergent growth trajectories between sexes. Scientific Reports. 2020;10(1). | Title |
|  | García-Martínez D, Torres-Tamayo N, Torres-Sanchez I, García-Río F, Bastir M. Morphological and functional implications of sexual dimorphism in the human skeletal thorax. American Journal of Physical Anthropology. 2016;161(3):467-77. | Title |
|  | Garcia-Usó M, Lima TF, Trindade IEK, Pimenta LAF, Trindade-Suedam IK. Three-dimensional tomographic assessment of the upper airway using 2 different imaging software programs: A comparison study. Am J Orthod Dentofacial Orthop. 2021;159(2):217-23. | Title |
|  | Garib D, Miranda F, Palomo JM, Pugliese F, Bastos JCD, dos Santos AM, et al. Orthopedic outcomes of hybrid and conventional Hyrax expanders: Secondary data analysis from a randomized clinical trial. Angle Orthodontist. 2021;91(2):178-86. | Title |
|  | Garib DG, Henriques JF, Janson G, de Freitas MR, Fernandes AY. Periodontal effects of rapid maxillary expansion with tooth-tissue-borne and tooth-borne expanders: a computed tomography evaluation. Am J Orthod Dentofacial Orthop. 2006;129(6):749-58. | Title |
|  | Garib DG, Henriques JF, Janson G, Freitas MR, Coelho RA. Rapid maxillary expansion--tooth tissue-borne versus tooth-borne expanders: a computed tomography evaluation of dentoskeletal effects. Angle Orthod. 2005;75(4):548-57. | Title |
|  | Garlikova, Z., et al. Nanostructure and bioactivity of mouse lung extracellular matrix scaffolds. in NANOCON 2017 - Conference Proceedings, 9th International Conference on Nanomaterials - Research and Application. 2018. | Title |
|  | Garnier, P., et al., Surgically treated congenital cleft palate in a 4-month-old kitten: medium-term clinical and CT assessment. Journal of Feline Medicine and Surgery Open Reports, 2022. 8(1). | Title |
|  | Garrett, B.J., et al., Skeletal effects to the maxilla after rapid maxillary expansion assessed with cone-beam computed tomography. American Journal of Orthodontics and Dentofacial Orthopedics, 2008. 134(1): p. 8.e1-8.e11. | Title |
|  | Garrido MB, Jagtap R, Hansen M. Retropharyngeal internal carotid artery: a review of three cases. Oral Maxillofac Surg. 2020;24(2):255-61. | Title |
|  | Gasparini G, Di Rocco C, Saponaro G, Marianetti TM, Foresta E, Rinaldo FM, et al. Evaluation of obstructive sleep apnea in pediatric patients with facio-craniostenosis: a brief communication. Childs Nerv Syst. 2012;28(8):1135-40. | Title |
|  | Gasparini G, Tamburrini G, Di Rocco C, Pelo S. Evaluation of obstructive sleep apnea in pediatric patients with facio-craniostenosis. Child's Nervous System. 2012;28(5):757-8. | Title |
|  | Gasperini G, da Silva BG, de Sá RT, Cardoso LL. Assessment of dimensional and volumetric of upper airways in orthognáthic surgery. International Journal of Oral and Maxillofacial Surgery. 2019;48:118. | Title |
|  | Gay I, Elidan J. Dysphonia caused by Forestier's disease. Ann Otol Rhinol Laryngol. 1988;97(3 Pt 1):275-6. | Title |
|  | Gayle J.A.,et al. Anesthetic considerations for robotic-assisted surgery, in Perioperative Management in Robotic Surgery. 2017. 35-43. | Title |
|  | Geerts L, Meyer R, Nolan H, Nel L, Nel DG, Brink L, et al. Reference standards for facial measurements in early third trimester South African fetuses, and the effect of maternal and fetal characteristics. Journal of Maternal-Fetal & Neonatal Medicine. | Title |
|  | Gekas J, Li B, Kamnasaran D. Current perspectives on the etiology of agnathia-otocephaly. European Journal of Medical Genetics. 2010;53(6):358-66. | Title |
|  | Gelesko S, Bui TG, Park ES, Dierks EJ, Bobek SL, Bell RB. A protocol for computer planning and intraoperative imaging as an aid to reconstruction of gunshot wounds to the face. Journal of Oral and Maxillofacial Surgery. 2013;71(9):e7-e8. | Title |
|  | Gengler NV, Kecskemethy HH, Harcke HT, Averill LW. Multimodality Assessment of Tracheal Features in MPS IVA: A Comparison. Pediatric Radiology. 2022;52(SUPPL 1):S54. | Title |
|  | Gengler NV, Kecskemethy HH, Harcke HT, Averill LW. Utility of Various Imaging Studies for Tracheal Assessment in Mucopolysaccharidosis IVA. Pediatric Radiology. 2022;52(SUPPL 1):S55. | Title |
|  | Genkin D, Zanette B, Grzela P, Tullis DE, Ratjen FA, Santyr G, et al. Development of an Airway Segmentation Pipeline for Ultrashort Echo Time MRI of Pediatric Cystic Fibrosis Lung Disease. American Journal of Respiratory and Critical Care Medicine. 2022;205(1). | Title |
|  | George RB, Melnick AH, Rose EC, Habib AS. Case series: Combined spinal epidural anesthesia for Cesarean delivery and ex utero intrapartum treatment procedure. Canadian Journal of Anesthesia. 2007;54(3):218-22. | Title |
|  | George, C., et al., Anesthetic and surgical management of mucoid retention cyst in the vallecular region: An airway challenge. Journal of Indian Association of Pediatric Surgeons, 2018. 23(4): p. 232-233. | Title |
|  | Germec-Cakan, D., et al., Comparison of facial soft tissue measurements on three-dimensional images and models obtained with different methods. Journal of Craniofacial Surgery, 2010. 21(5): p. 1393-1399. | Title |
|  | Geyer SH, Weninger WJ. Metric characterization of the aortic arch of early mouse fetuses and of a fetus featuring a double lumen aortic arch malformation. Annals of Anatomy-Anatomischer Anzeiger. 2013;195(2):175-82. | Title |
|  | Gharravi, A.M., et al., Design and validation of perfusion bioreactor with low shear stress for tissue engineering. Journal of Medical and Biological Engineering, 2013. 33(2): p. 185-192. | Title |
|  | Ghezzi CE, Risse PA, Marelli B, Muja N, Barralet JE, Martin JG, et al. An airway smooth muscle cell niche under physiological pulsatile flow culture using a tubular dense collagen construct. Biomaterials. 2013;34(8):1954-66. | Title |
|  | Gholinia F, Habibi L, Amrollahi Boyouki M. Cephalometric Evaluation of the Upper Airway in Different Skeletal Classifications of Jaws. J Craniofac Surg. 2019;30(5):e469-e74. | Abstract |
|  | Ghoubril, J.V. and F.M. Abou Obeid, Three-dimensional assessment of facial bone cavities in Class I occlusion with normodivergent skeletal pattern. Odonto-stomatologie tropicale = Tropical dental journal, 2012. 35(140): p. 21-30. | Abstract |
|  | Ghoussoub MS, Garcia R, Sleilaty G, Rifai K. Effect of Rapid Maxillary Expansion on Condyle-fossa Relationship in Growing Patients. J Contemp Dent Pract. 2018;19(10):1189-98. | Title |
|  | Ghoussoub MS, Rifai K, Garcia R, Sleilaty G. Effect of Rapid Maxillary Expansion on Glenoid Fossa and Condyle-Fossa Relationship in Growing Patients (MEGP): Study Protocol for a Controlled Clinical Trial. Journal of International Society of Preventive and Community Dentistry. 2018;8(2):130-6. | Title |
|  | Ghoussoub MS, Sleilaty G, Garcia R, Rifai K. Correlation between Temporomandibular Joints and Nasal Cavity Width in Growing Patients after Rapid Maxillary Expansion. J Contemp Dent Pract. 2019;20(6):686-92. | Title |
|  | Giannini NP, Macrini TE, Wible JR, Rowe TB, Simmons NB. THE INTERNAL NASAL SKELETON OF THE BAT PTEROPUS LYLEI K. ANDERSEN, 1908 (CHIROPTERA: PTEROPODIDAE). Annals of Carnegie Museum. 2012;81(1):1-17. | Title |
|  | Gianoni-Capenakas S, Flores-Mir C, Vich ML, Pacheco-Pereira C. Oropharyngeal 3-dimensional changes after maxillary expansion with 2 different orthodontic approaches. American journal of orthodontics and dentofacial orthopedics. 2021;159(3):352‐9. | Title |
|  | Giap, H.V., et al., Pharyngeal Airway Morphology in Skeletal Class III With Mandibular Asymmetry is Improved After Bimaxillary Orthognathic Surgery. Journal of Oral and Maxillofacial Surgery, 2021. 79(5): p. 1107-1121. | Title |
|  | Gibelli D, Cellina M, Gibelli S, Oliva AG, Termine G, Sforza C. Three-Dimensional Assessment of Pharyngeal Volume on Computed Tomography Scans: Applications to Anesthesiology and Endoscopy. J Craniofac Surg. 2020;31(3):755-8. | Title |
|  | Gibson M, Cron RQ, Stoll ML, Kinard BE, Patterson T, Kau CH. A 3D CBCT Analysis of Airway and Cephalometric Values in Patients Diagnosed with Juvenile Idiopathic Arthritis Compared to a Control Group. Applied Sciences-Basel. 2022;12(9). | Title |
|  | Gidarakou, I.K., et al., Comparison of skeletal and dental morphology in asymptomatic volunteers and symptomatic patients with bilateral degenerative joint disease. Angle Orthodontist, 2003. 73(1): p. 71-78. | Title |
|  | Giesel FL, Mehndiratta A, von Tengg-Kobligk H, Schaeffer A, Teh K, Hoffman EA, et al. Rapid Prototyping Raw Models on the Basis of High Resolution Computed Tomography Lung Data for Respiratory Flow Dynamics. Academic Radiology. 2009;16(4):495-8. | Title |
|  | Gilhotra R, McGowan C, Htut S, Gururatsakul M, Peter-Kini G, Boyd P. Epstein-Barr virus-associated mucocutaneous ulceration: A case report and literature review. Journal of Gastroenterology and Hepatology (Australia). 2020;35(SUPPL 1):179-80. | Title |
|  | Giralt-Hernando M, Valls-Ontañón A, Haas Junior OL, Masià-Gridilla J, Hernández-Alfaro F. What are the Surgical Movements in Orthognathic Surgery That Most Affect the Upper Airways? A Three-Dimensional Analysis. Journal of Oral and Maxillofacial Surgery. 2021;79(2):450-62. | Title |
|  | Gitomer, S.A., et al., Reducing Surgical Revisions in Intracranial Complications of Pediatric Acute Sinusitis. Otolaryngology - Head and Neck Surgery (United States), 2018. 159(2): p. 359-364. | Title |
|  | Gjørup, H., et al., Morphological characteristics of frontal sinus and nasal bone focusing on bone resorption and apposition in hypophosphatemic rickets. Orthodontics and Craniofacial Research, 2013. 16(4): p. 246-255. | Title |
|  | Glade RS, Vinson K, Becton D, Bhutta S, Buckmiller LM. Management of complicated hemangiomas with vincristine/vinblastine: Quantitative response to therapy using MRI. International Journal of Pediatric Otorhinolaryngology. 2010;74(11):1221-5. | Title |
|  | Glasheen J, Hennelly D, Cusack S. Maxillofacial Injury-Not Always a Difficult Airway. Prehosp Disaster Med. 2015;30(4):421-4. | Title |
|  | Glupker L, Kula K, Parks E, Babler W, Stewart K, Ghoneima A. Three-dimensional computed tomography analysis of airway volume changes between open and closed jaw positions. Am J Orthod Dentofacial Orthop. 2015;147(4):426-34. | Title |
|  | Glushko, A.V., et al., Analysis of a change in the position of the hyoid bone when displacing the lower jaw in patients with dentofacial malformations. Vestnik rentgenologii i radiologii, 2014(6): p. 5-12. | Title |
|  | Gode S, Ozturk A, Berber V, Kısmalı E. Effect of Injectable Platelet-Rich Fibrin on Diced Cartilage's Viability in Rhinoplasty. Facial plastic surgery. 2019;35(4):393‐6. | Title |
|  | Goergen MJ, Holton NE, Grünheid T. Morphological interaction between the nasal septum and nasofacial skeleton during human ontogeny. J Anat. 2017;230(5):689-700. | Title |
|  | Gokce SM, Gorgulu S, Gokce HS, Bengi AO, Karacayli U, Ors F. Evaluation of pharyngeal airway space changes after bimaxillary orthognathic surgery with a 3-dimensional simulation and modeling program. American Journal of Orthodontics and Dentofacial Orthopedics. 2014;146(4):477-92. | Title |
|  | Gokce SM, Gorgulu S, Karacayli U, Gokce HS, Battal B. Three-dimensional evaluation of nasal and pharyngeal airway after le Fort i maxillary distraction osteogenesis. International Journal of Oral and Maxillofacial Surgery. 2015;44(4):455-61. | Title |
|  | Golchini E, Rasoolijazi H, Momeni F, Shafaat P, Ahadi R, Jafarabadi MA, et al. Investigation of the Relationship Between Mandibular Morphology and Upper Airway Dimensions. J Craniofac Surg. 2020;31(5):1353-61. | Full text  Other outcomes |
|  | Goldstein I, Reiss A, Rajamim BS, Tamir A. Nomogram of maxillary bone length in normal pregnancies. Journal of Ultrasound in Medicine. 2005;24(9):1229-33. | Title |
|  | Goldstein I, Tamir A, ItskovitzEldor J, Zimmer EZ. Growth of the fetal nose width and nostril distance in normal pregnancies. Ultrasound in Obstetrics & Gynecology. 1997;9(1):35-8. | Title |
|  | Goldstein I, Tamir A, Weiner Z, Jakobi P. Dimensions of the fetal facial profile in normal pregnancy. Ultrasound in Obstetrics & Gynecology. 2010;35(2):191-4. | Title |
|  | Gomes AF, Fontenele RC, Zanon MF, Groppo FC, Haiter Neto F, Freitas DQ. Influence of skeletal class and facial type on nose dimensions in a Brazilian subpopulation: a CBCT study. Braz Oral Res. 2021;35:e036. | Title |
|  | Gomes VL, Gonçalves LC, Costa MM, Lucas Bde L. Interalar distance to estimate the combined width of the six maxillary anterior teeth in oral rehabilitation treatment. J Esthet Restor Dent. 2009;21(1):26-35; discussion 6. | Title |
|  | Gómez Roselló, E., et al., Facial fractures: classification and highlights for a useful report. Insights into Imaging, 2020. 11(1). | Title |
|  | Gomez Y, Zamora N, Tarazona B, Bellot-Arcis C, Paredes-Gallardo V. Cross-sectional human study of soft tissue chin (STC) thickness in adult patients in relation to sex, facial pattern and skeletal class. Journal of Cranio-Maxillofacial Surgery. 2017;45(8):1205-11. | Title |
|  | Gonc¸ales E, Gonc¸ales AGB, Tieghe Neto V. Analysis of the dento-skeletal effects using cone beam computed tomography (CBCT) after surgically assisted maxillary expansion in adult patients. International Journal of Oral and Maxillofacial Surgery. 2011;40(10):1195. | Title |
|  | Gonçales ES, Assis DSFRd, Capelozza ALÁ, Alvares LC. Estudo radiográfico digital indireto do efeito da expansão de maxila cirurgicamente assistida (EMCA) sobre o septo nasal. Revista Dental Press de Ortodontia e Ortopedia Facial. 2007;12(5):85-91. | Title |
|  | Gonçales ES, Duarte MA, Palmieri C, Jr., Zakhary GM, Ghali GE. Retrospective analysis of the effects of orthognathic surgery on the pharyngeal airway space. J Oral Maxillofac Surg. 2014;72(11):2227-40. | Title |
|  | Gonçales ES, Gonçales AG, Tieghi Neto V, Palmieri CF, Ghali GE. Tomographic analysis of the volume and area of the pharyngeal airspace of patients undergoing orthognathic surgery. Journal of Oral and Maxillofacial Surgery. 2017;75(10):e349. | Abstract |
|  | Gonçales ES, Neto VT, Palmieri CF, Ghali GE. 3D volumetric analysis of pharyngeal airway of 50 individuals who underwent maxillary advancement. Journal of Oral and Maxillofacial Surgery. 2015;73(9):e22. | Abstract |
|  | Gonçalves da Silva Leite, J., et al., Morphology of nerve endings in vocal fold of human newborn. International Journal of Pediatric Otorhinolaryngology, 2016. 89: p. 55-59. | Title |
|  | Gonçalves JR, Gomes LCR, Vianna AP, Rodrigues DB, Gonçalves DAG, Wolford LM. Airway space changes after maxillomandibular counterclockwise rotation and mandibular advancement with TMJ Concepts® total joint prostheses: Three-dimensional assessment. International Journal of Oral and Maxillofacial Surgery. 2013;42(8):1014-22. | Title |
|  | **Gong X, Li W, Gao X. Effects of Craniofacial Morphology on Nasal Respiratory Function and Upper Airway Morphology. J Craniofac Surg. 2018;29(7):1717-22.** | **Included** |
|  | Gonuldas SR, Ozsoy OP, Pamukcu H, Kural F. Comparison of Dentofacial and Periodontal Effects of Rapid and Semi-rapid Maxillary Expansion. Meandros Medical and Dental Journal. 2017;18(1):33-46. | Title |
|  | Gonzalez SR, Jones JK, Golinko MS. Surgical Approach in a Patient With Agnathia-Otocephaly Complex: Three-Stage Mandibular Distraction Protocol. Journal of Craniofacial Surgery. 2020;31(1):E84-E9. | Title |
|  | Gopal R, Tripathi T, Rai P, Kanase A. Three-dimensional assessment of pharyngeal airway space by MRI in class II division 1 patients treated by twin block appliance. Journal of Clinical and Diagnostic Research. 2018;12(9):ZC20-ZC3. | Title |
|  | Gordina, G.S., et al., [Role of multislice spiral computed tomography in the evaluation of changes in upper airway volume during surgical treatment in patients with dentomaxillary abnormalities]. Vestnik rentgenologii i radiologii, 2013(2): p. 21-26. | Full text  Other outcomes |
|  | Gorgulu S, Gokce SM, Olmez H, Sagdic D, Ors F. Nasal cavity volume changes after rapid maxillary expansion in adolescents evaluated with 3-dimensional simulation and modeling programs. American Journal of Orthodontics and Dentofacial Orthopedics. 2011;140(5):633-40. | Title |
|  | Gorgulu S, Sagdic D, Akin E, Karacay S, Bulakbasi N. Tongue movements in patients with skeletal Class III malocclusions evaluated with real-time balanced turbo field echo cine magnetic resonance imaging. American Journal of Orthodontics and Dentofacial Orthopedics. 2011;139(5):E405-E14. | Title |
|  | Gottsauner-Wolf S, Laimer J, Bruckmoser E. Posterior Airway Changes Following Orthognathic Surgery in Obstructive Sleep Apnea. Journal of Oral and Maxillofacial Surgery. 2018;76(5). | Title |
|  | Goudra, B. and P.M. Singh, Airway Management During Upper GI Endoscopic Procedures: State of the Art Review. Digestive Diseases and Sciences, 2017. 62(1): p. 45-53. | Title |
|  | Goumas IK, Itri E, D'Addezzio F, Montanari E, Zanetti G. Supine percutaneous nephrolithotomy (PCNL) in a tetraparetic patient with skeletal deformity. Journal of Endourology. 2012;26:A489. | Title |
|  | Gracco A, Perri A, Siviero L, Bonetti GA, Cocilovo F, Stellini E. Multidisciplinary correction of anterior open bite relapse and upper airway obstruction. Korean Journal of Orthodontics. 2015;45(1):47-56. | Title |
|  | Grant, M.P., N.T. Iliff, and P.N. Manson, Evaluation of acute management of naso-orbito-ethmoidal fractures. Operative Techniques in Plastic and Reconstructive Surgery, 2002. 8(4): p. 230-239. | Title |
|  | **Grauer D, Cevidanes LS, Styner MA, Ackerman JL, Proffit WR. Pharyngeal airway volume and shape from cone-beam computed tomography: relationship to facial morphology. Am J Orthod Dentofacial Orthop. 2009;136(6):805-14.** | **Included** |
|  | Graviero, G., et al., The role of three-dimensional CT in the evaluation of nasal structures and anomalies. European Archives of Oto-Rhino-Laryngology, 2011. 268(8): p. 1163-1167. | Title |
|  | Grayson AK, Hearnden V, Bolt R, Jebreel A, Colley HE, Murdoch C. Use of a Rho kinase inhibitor to increase human tonsil keratinocyte longevity for three-dimensional, tissue engineered tonsil epithelium equivalents. Journal of Tissue Engineering and Regenerative Medicine. 2018;12(3):E1636-E46. | Title |
|  | Green PA, Van Valkenburgh B, Pang B, Bird D, Rowe T, Curtis A. Respiratory and olfactory turbinal size in canid and arctoid carnivorans. Journal of Anatomy. 2012;221(6):609-21. | Title |
|  | Greess H, Nömayr A, Tomandl B, Blank M, Lell M, Lenz M, et al. 2D and 3D visualisation of head and neck tumours from spiral-CT data. European Journal of Radiology. 2000;33(3):170-7. | Title |
|  | Gregory A, Zhonghui X, Lee S, Yun JH, Saferali A, Hersh CP, et al. K-means subtypes of smokers in the copdgene study have distinct patterns of COPD progression and distinct serum protein biomarker and transcriptomic profiles. American Journal of Respiratory and Critical Care Medicine. 2020;201(1). | Title |
|  | Grelard F, Baldacci F, Vialard A, Domenger JP. New methods for the geometrical analysis of tubular organs. Medical Image Analysis. 2017;42:89-101. | Title |
|  | Grélard, F., et al. Improving curve skeletons of tubular volumes. in 2016 6th International Conference on Image Processing Theory, Tools and Applications, IPTA 2016. 2017. | Title |
|  | Grelli, K.N., et al., Bronchopulmonary dysplasia precursors influence risk of white matter injury and adverse neurodevelopmental outcome in preterm infants. Pediatric Research, 2021. 90(2): p. 359-365. | Title |
|  | Gribova MN, Pluijmers BI, Resnick CM, Caron C, Borghi A, Koudstaal MJ, et al. Is There a Difference in Orbital Volume Between Affected and Unaffected Sides in Patients With Unilateral Craniofacial Microsomia? J Oral Maxillofac Surg. 2018;76(12):2625-9. | Title |
|  | Grill, F.D., et al., Facilitating CAD/CAM nasoalveolar molding therapy with a novel click-in system for nasal stents ensuring a quick and user-friendly chairside nasal stent exchange. Scientific Reports, 2018. 8(1). | Title |
|  | Grimes D, MacLeod I, Taylor T, O'Connor M, Sidebottom A. Computed tomography as an aid to planning intubation in the difficult airway. Br J Oral Maxillofac Surg. 2016;54(1):80-2. | Title |
|  | Groom P, Berwick R. The ‘Oozy Suzy’: A Part Task Trainer for Front of Neck Access (FONA). Trends in Anaesthesia and Critical Care. 2020;30:e157. | Title |
|  | Gross MD, Arbel G, Hershkovitz I. Three-dimensional finite element analysis of the facial skeleton on simulated occlusal loading. J Oral Rehabil. 2001;28(7):684-94. | Title |
|  | Grothausmann R, Kellner M, Heidrich M, Lorbeer RA, Ripken T, Meyer H, et al. Method for 3D Airway Topology Extraction. Computational and Mathematical Methods in Medicine. 2015;2015. | Title |
|  | Gruber DM, Berger UE, Sator MO, Horak F, Huber JC. Computerized assessment of facial hair growth. Fertil Steril. 1999;72(4):737-9. | Title |
|  | Grünheid T, Larson CE, Larson BE. Midpalatal suture density ratio: A novel predictor of skeletal response to rapid maxillary expansion. Am J Orthod Dentofacial Orthop. 2017;151(2):267-76. | Title |
|  | Gruszczyńska K, Likus W, Onyszczuk M, Wawruszczak R, Gołdyn K, Olczak Z, et al. How does nonsyndromic craniosynostosis affect on bone width of nasal cavity in children? – Computed tomography study. PLoS ONE. 2018;13(7). | Title |
|  | Gu M, McGrath CPJ, Wong RWK, Hagg U, Yang YQ. Cephalometric norms for the upper airway of 12-year-old Chinese children. Head & Face Medicine. 2014;10. | Title |
|  | Guha A, Pradhan K. Secondary motion in three-dimensional branching networks. Physics of Fluids. 2017;29(6). | Title |
|  | Guia S, Oleg D, Denis M, Elena B. Surgical treatment of patients with severe craniofacial injury complicated by basal liquorrhea. Brain Injury. 2012;26(4-5):398-9. | Title |
|  | Guijarro-Martínez R, Hernández-Alfaro F, Swennen G, Mareque-Bueno J. Upper airway evaluation using cone-beam computerized tomography (CBCT): Systematic review and results after mono and bimaxillary advancement. International Journal of Oral and Maxillofacial Surgery. 2011;40(10):e12. | Title |
|  | Guijarro-Martínez R, Swennen GRJ. Cone-beam computerized tomography imaging and analysis of the upper airway: A systematic review of the literature. International Journal of Oral a nd Maxillofacial Surgery. 2011;40(11):1227-37. | Title |
|  | Guijarro-Martínez R, Swennen GRJ. Three-dimensional cone beam computed tomography definition of the anatomical subregions of the upper airway: A validation study. International Journal of Oral and Maxillofacial Surgery. 2013;42(9):1140-9. | Title |
|  | Guimaraes TM, Bariani RC, Iafigliola SG, Guimaraes CM, Chaves CM, Ferraz O, et al. Cone beam computed tomography in assessment on pharynx effects of orthopedic-surgical treatment - a review of the literature. Sleep Science. 2019;12(2):106-9. | Title |
|  | Guimarães, R.E.D.S., et al., Absence of nasal air flow and maxillary sinus development. Brazilian Journal of Otorhinolaryngology, 2007. 73(2): p. 161-164. | Title |
|  | Gulisano, M., et al., The shape of the nasopharynx in youth: statistical study. Bollettino della Società italiana di biologia sperimentale, 1992. 68(11): p. 647-653. | Title |
|  | Güner, D.D., Y. Öztürk, and H.B. Sayman, Evaluation of the effects of functional orthopaedic treatment on temporomandibular joints with single-photon emission computerized tomography. European Journal of Orthodontics, 2003. 25(1): p. 9-12. | Title |
|  | Gungor AY, Turkkahraman H, Baykul T, Alkis H. Comparison of the effects of rapid maxillary expansion and surgically assisted rapid maxillary expansion in the sagittal, vertical and transverse planes. Medicina Oral Patologia Oral Y Cirugia Bucal. 2012;17(2):E311-E9. | Title |
|  | Gunhan Ket al. Does maxillary arch remodeling exist in nasal polyposis? Am Journal Rhinology and Allergy, 2010. 24(6): 428-432. | Title |
|  | Gunnar G, James FK, Kris K, Konstance K, Avik R. Intranasal Administration of ACIS KEPTIDE™ Prevents SARS-CoV2-Induced Acute Toxicity in K18-hACE2 Humanized Mouse Model of COVID-19: A Mechanistic Insight for the Prophylactic Role of KEPTIDE™ in COVID-19. 2020. | Title |
|  | Günther, L., et al., Clinical course and implications of congenital nasal pyriform stenosis and solitary median maxillary central incisor in a newborn: A case report. Journal of Medical Case Reports, 2014. 8(1). | Title |
|  | Guo J, Tan JZ, Yang YJ, Zhou H, Hu SL, Hashan A, et al. Variation and signatures of selection on the human face. Journal of Human Evolution. 2014;75:143-52. | Title |
|  | Guo X, Meng T, Huang J, Wang X, Lian W, Deng K, et al. 3D Facial Analysis in Acromegaly: Gender-Specific Features and Clinical Correlations. Frontiers in Endocrinology. 2018;9. | Title |
|  | Guo, L., et al. Three dimension reconstruction of medical images based on an improved Marching Cubes algorithm. in Proceedings of the 2013 6th International Conference on Biomedical Engineering and Informatics, BMEI 2013. 2013. | Title |
|  | Gupta A, Kumar R, Bhattacharya D, Thukral B, Suri J. Craniofacial and upper airway profile assessment in North Indian patients with obstructive sleep apnea. Lung India. 2019;36(2):94-101. | Title |
|  | Gupta A, Meaike J, Pagano A, Curcio DF, Som P, Laitman JT. Use of axial ct scans to assess early postnatal development of the internal nares: A test of reliability. FASEB Journal. 2016;30. | Title |
|  | Gupta GS, Meghana H, Shetty U, Rai D, Rao PSN, Kini R. Assessment of the Morphology of Soft Palate by Using Cone-Beam Computed Tomography. Journal of Indian Academy of Oral Medicine and Radiology. 2022;34(2):213-7. | Title |
|  | Gupta J, Chakrabarty B, Singh G, Singh S, Kumar A, Xess I, et al. A rare infective cause of stroke in an immunocompetent child. Brain Dev. 2021;43(1):152-6. | Title |
|  | **Gupta JV, Makhija PG, Gupta KC. Does a correlation exist between nasal airway volume and craniofacial morphology: A cone beam computed tomography study. Indian J Dent Res. 2016;27(4):359-63.** | **Included** |
|  | Gurani SF, Di Carlo G, Thorn JJ, Ingerslev J, Cattaneo PM, Pinholt EM. Two-Year Postoperative Upper Airway Cone-Beam Computed Tomographic Outcomes Based on a Verified Upper Airway Analysis Following Bimaxillary Orthognathic Surgery. Journal of Oral and Maxillofacial Surgery. 2019;77(7):1435-45. | Title |
|  | Gurgel JA, Tiago CM, Normando D. Transverse changes after surgically assisted rapid palatal expansion. International Journal of Oral and Maxillofacial Surgery. 2014;43(3):316-22. | Title |
|  | Gurgel M, Cevidanes L, Pereira R, Costa F, Ruellas A, Bianchi J, et al. Three-dimensional craniofacial characteristics associated with obstructive sleep apnea severity and treatment outcomes. Clinical Oral Investigations. 2022;26(1):875-87. | Title |
|  | Gurgel ML, Chaves C, Cevidanes LHS, Silva PGD, Carvalho FSR, Kurita LM, et al. Methodological parameters for upper airway assessment by cone-beam computed tomography in adults with obstructive sleep apnea: a systematic review of the literature and meta-analysis. Sleep and Breathing. 2022. | Title |
|  | Gurler G, Akar NK, Delilbasi C, Kacar I. Skeletal changes following surgically assisted rapid maxillary expansion (SARME). European Oral Research. 2018;52(2):94-8. | Title |
|  | Guyomarc'h, P., et al., Anthropological facial approximation in three dimensions (AFA3D): Computer-assisted estimation of the facial morphology using geometric morphometrics. Journal of Forensic Sciences, 2014. 59(6): p. 1502-1516. | Title |
|  | Güzeldir, O.T., et al., The effect of infrahyoid muscle sectioning on hyoid bone position and oropharyngeal air column volume. Laryngoscope, 2015. 125(6): p. 1480-1484. | Title |
|  | Ha, J.Y., et al., Feasibility study of ultra-low-dose dedicated maxillofacial computed tomography using filter-based spectral shaping in patients with craniofacial trauma: Assessment of image quality and radiation dose. Quantitative Imaging in Medicine and Surgery, 2021. 11(4): p. 1292-1302. | Title |
|  | Ha, S., et al., Magnetic resonance imaging of the levator veli palatini muscle in speakers with repaired cleft palate. Cleft Palate-Craniofacial Journal, 2007. 44(5): p. 494-505. | Title |
|  | Haarmann S, Budihardja AS, Wolff KD, Wangerin K. Changes in acoustic airway profiles and nasal airway resistance after Le Fort I osteotomy and functional rhinosurgery: A prospective study. Int Journal of Oral and Maxillofacial Surgery. 2009;38(4):321-5. | Title |
|  | **Habumugisha J, Ma SY, Mohamed AS, Cheng B, Zhao MY, Bu WQ, et al. Three-dimensional evaluation of pharyngeal airway and maxillary arch in mouth and nasal breathing children with skeletal Class I and II. BMC Oral Health. 2022;22(1):320.** | **Included**  **(nose breathers’ data)** |
|  | Haenssler A, Fang X, Perry J. Is velopharyngeal ratio really an accurate clinical determinant of velopharyngeal function? Cleft Palate-Craniofacial Journal. 2020;57(4):96-7. | Title |
|  | Haenssler A, Fang X, Perry J. Velopharyngeal ratios used as clinical determinants of velopharyngeal function. Cleft Palate-Craniofacial Journal. 2021;58(4 SUPPL):107-8. | Title |
|  | Haenssler A, Middleton S, Mason K, Kotlarek K, Mason R, Perry J. Normative adenoid development from infancy through adulthood. Cleft Palate-Craniofacial Journal. 2019;56(1):57. | Title |
|  | Haenssler AE, Fang XM, Perry JL. Effective Velopharyngeal Ratio: A More Clinically Relevant Measure of Velopharyngeal Function. Journal of Speech Language and Hearing Research. 2020;63(11):3586-93. | Title |
|  | Hafsa C, Ben Alaya T, Kriaa S, Jerbi S, Golli M, Hamdi MH, et al. [Maxillo-facial location of hydatic cyst]. Ann Otolaryngol Chir Cervicofac. 2008;125(3):160-3. | Title |
|  | Hajeer MY, Ayoub AF, Millett ET. Three-dimensional assessment of facial soft-tissue asymmetry before and after orthognathic surgery. British Journal of Oral & Maxillofacial Surgery. 2004;42(5):396-404. | Title |
|  | Halim, I.A., et al., Preliminary study: evaluating the reliability of CBCT images for tongue space measurements in the field of orthodontics. Oral Radiology, 2021. 37(2): p. 256-266. | Title |
|  | Hall G, Chen L, Ang W, Pennell C, Newnham J, Sly P. Increased fetal growth protects against early wheeze, airway hyper-responsiveness (AHR) and current asthma in early mid-childhood:Results from the Raine birth cohort. European Resp Journal 2011;38. | Title |
|  | Hall JG. Importance of Muscle Movement for Normal Craniofacial Development. Journal of Craniofacial Surgery. 2010;21(5):1336-8. | Title |
|  | Hamad, S.A. and E.N. Youhanna, Displacement of Dental Implants into the Maxillary Sinus: A Case Series Study. Bahrain Medical Bulletin, 2022. 44(1): p. 808-812. | Title |
|  | Hamarneh G, Jassi P. VascuSynth Simulating vascular trees for generating volumetric image data with ground-truth segmentation and tree analysis. Computerized Medical Imaging and Graphics. 2010;34(8):605-16. | Title |
|  | Hamilton NJ, Kanani M, Roebuck DJ, Hewitt RJ, Cetto R, Culme-Seymour EJ, et al. Tissue-Engineered Tracheal Replacement in a Child: A 4-Year Follow-Up Study. American Journal of Transplantation. 2015;15(10):2750-7. | Title |
|  | Hammond, E., et al., Impact of advanced detector technology and iterative reconstruction on low-dose quantitative assessment of lung computed tomography density in a biological lung model. Medical Physics, 2018. 45(8): p. 3657-3670. | Title |
|  | Han JJ, Hong DH, Hwang SJ. Maxillary Expansion and Mandibular Setback Surgery With and Without Mandibular Anterior Segment Osteotomy to Correct Mandibular Prognathism With Obstructive Sleep Apnea. Journal of Craniofacial Surgery. 2017;28(3):723-30. | Title |
|  | Han MD, Antonini F, Borba AM, Miloro M. Are we able to predict airway dimensional changes in isolated mandibular setback? International Journal of Oral and Maxillofacial Surgery. 2022;51(4):487-92. | Title |
|  | Han YZ, Tian Y, Zhang H, Zhao YQ, Xu M, Guo XY. Radiologic indicators for prediction of difficult laryngoscopy in patients with cervical spondylosis. Acta Anaesthesiologica Scandinavica. 2018;62(4):474-82. | Title |
|  | Han, D.S.Y., Y.S. Han, and J.H. Park, A new approach to the treatment of nasal bone fracture: Radiologic classification of nasal bone fractures and its clinical application. Journal of Oral and Maxillofacial Surgery, 2011. 69(11): p. 2841-2847. | Title |
|  | Han, X., et al., Runx2-twist1 interaction coordinates cranial neural crest guidance of soft palate myogenesis. eLife, 2021. 10: p. 1-23. | Title |
|  | Han, Y.S. and H. Lee, The Influential Bony Factors and Vectors for Predicting Soft Tissue Responses After Orthognathic Surgery in Mandibular Prognathism. Journal of Oral and Maxillofacial Surgery, 2018. 76(5): p. 1095.e1-1095.e14. | Title |
|  | Haner ST, Kanavakis G, Matthey F, Gkantidis N. Valid 3D surface superimposition references to assess facial changes during growth. Scientific Reports. 2021;11(1). | Title |
|  | Hanikeri M, Waterhouse N, Kirkpatrick N, Peterson D, Macleod I. The management of midline transcranial nasal dermoid sinus cysts. Br J Plast Surg. 2005;58(8):1043-50. | Title |
|  | Hanna, B.C., et al., Morphological consequences of lateral outfracture of the inferior turbinate. Journal of Laryngology and Otology, 2013. 127(3): p. 323-328. | Title |
|  | Hara H, Yoshida S, Toyota H, Yamashita H. Application of 3D-CT evaluation of upper airway morphology of osas patients for selection of surgical treatment candidates. Sleep and Biological Rhythms. 2011;9(4):285. | Title |
|  | Haralambidis A, Ari-Demirkaya A, Acar A, Kucukkeles N, Ates M, Ozkaya S. Morphologic changes of the nasal cavity induced by rapid maxillary expansion: A study on 3-dimensional computed tomography models. American Journal of Orthodontics and Dentofacial Orthopedics. 2009;136(6):815-21. | Title |
|  | Hariri BM, Payne SJ, Chen B, Mansfield C, Doghramji LJ, Adappa ND, et al. In vitro effects of anthocyanidins on sinonasal epithelial nitric oxide production and bacterial physiology. Am J Rhinol Allergy. 2016;30(4):261-8. | Title |
|  | Harrison LM, Hallac RR, Derderian CA. Three-Dimensional Analysis of Bilateral Cleft Lip and Palate Nasal Deformity. Cleft Palate-Craniofacial Journal. 2021;58(1):105-13. | Title |
|  | Harrison, L.M., R.R. Hallac, and C.A. Derderian, Three-Dimensional Analysis of Bilateral Cleft Lip and Palate Nasal Deformity. Cleft Palate-Craniofacial Journal, 2021. 58(1): p. 105-113. | Title |
|  | Hart PS, McIntyre BP, Kadioglu O, Currier GF, Sullivan SM, Li J, et al. Postsurgical volumetric airway changes in 2-jaw orthognathic surgery patients. Am J Orthod Dentofacial Orthop. 2015;147(5):536-46. | Title |
|  | Hartman C, Holton N, Miller S, Yokley T, Marshall S, Srinivasan S, et al. Nasal Septal Deviation and Facial Skeletal Asymmetries. Anatomical Record-Advances in Integrative Anatomy and Evolutionary Biology. 2016;299(3):295-306. | Title |
|  | Hasan Arshad S. et al. Assessing small airway function for early detection of lung function impairment. Eur Resp Journal 2020. 56(1). | Title |
|  | Hasanin M, ElNaghy R, Olson D, Al-Jewair T. Three-dimensional analysis of upper airway and craniofacial morphology in orthodontic adolescents with Attention Deficit Hyperactivity Disorder (ADHD): A comparative retrospective study. Int Orthod. 2021;19(4):622-32. | Title |
|  | Haskell BS, Voor MJ, Roberts AM. A consideration of factors affecting palliative oral appliance effectiveness for obstructive sleep apnea: a scoping review. Journal of Clinical Sleep Medicine. 2021;17(4):833-48. | Title |
|  | Haskell JA, Haskell BS, Spoon ME, Feng C. The relationship of vertical skeletofacial morphology to oropharyngeal airway shape using cone beam computed tomography: possible implications for airway restriction. Angle Orthod. 2014;84(3):548-54. | Full text  Other outcomes |
|  | Hassan R, Azmi A, Zreaqat M, Yusuf A. Measurements of Facial Morphology in Normal Malay Children Using a Stereo Photogrammetry, 3dMD System. International Medical Journal. 2011;18(1):70-3. | Title |
|  | Hassegawa CA, Garcia-Uso MA, Yatabe-Ioshida MS, Trindade IEK, Fukushiro AP, Carreira DGG, et al. Internal nasal dimensions of children with unilateral cleft lip and palate and maxillary atresia: comparison between acoustic rhinometry technique and cone-beam computed tomography. Codas. 2021;33(3). | Title |
|  | Hatab NA, Konstantinović VS, Mudrak JK. Pharyngeal airway changes after mono- and bimaxillary surgery in skeletal class III patients: Cone-beam computed tomography evaluation. J Craniomaxillofac Surg. 2015;43(4):491-6. | Title |
|  | Hatch CD, Wehby GL, Nidey NL, Uribe LMM. Effects of Objective 3-Dimensional Measures of Facial Shape and Symmetry on Perceptions of Facial Attractiveness. Journal of Oral and Maxillofacial Surgery. 2017;75(9):1958-70. | Title |
|  | Hatcher DC. Cone beam computed tomography: craniofacial and airway analysis. Dent Clin North Am. 2012;56(2):343-57. | Abstract |
|  | Hatcher, D.C. and C.L. Aboudara, Diagnosis goes digital. Am J Orthod Dentofacial Orthop 2004. 125(4): p. 512-515. | Title |
|  | Havranek T, Miladinovic B, Wadhawan R, Carver JD. Factors that affect the postnatal increase in superior mesenteric artery blood flow velocity in very low birth weight preterm infants. Journal of Perinatal Medicine. 2012;40(5):565-70. | Title |
|  | Havron AG, Aronovich S, Shelgikar AV, Kim HL, Conley RS. 3D Airway changes using CBCT in patients following mandibular setback surgery ± maxillary advancement. Orthod Craniofac Res. 2019;22 Suppl 1:30-5. | Title |
|  | Hayama, S., et al., Air trapping and arboreal locomotor adaptation in primates: a review of experiments on humans. Zeitschrift für Morphologie und Anthropologie, 2002. 83(2-3): p. 149-159. | Title |
|  | Hayes GM, Friend EJ, Jeffery ND. Relationship between pharyngeal conformation and otitis media with effusion in Cavalier King Charles spaniels. Veterinary Record. 2010;167(2):55-8. | Title |
|  | He JL, Wang YJ, Hu HT, Liao Q, Zhang WY, Xiang XR, et al. Impact on the upper airway space of different types of orthognathic surgery for the correction of skeletal class III malocclusion: A systematic review and meta-analysis. International Journal of Surgery. 2017;38:31-40. | Title |
|  | He, D., Y. Zhang, and E. Ellis Iii, Panfacial Fractures: Analysis of 33 Cases Treated Late. Journal of Oral and Maxillofacial Surgery, 2007. 65(12): p. 2459-2465. | Title |
|  | Head K, Sharp S, Chong LY, Hopkins C, Philpott C. Topical and systemic antifungal therapy for chronic rhinosinusitis. Cochrane Database of Systematic Reviews. 2018(9). | Title |
|  | Heald M, Jones E, Srivastava C, Gnanamuttu A. Anaesthesia for super obesity-a trainee's perspective. Anaesthesia. 2015;70:73. | Title |
|  | Heaton, R., H. Guscott, and M. Lau, SU‐FF‐T‐400: Supine Craniospinal Irradiation Using Electronic Portal Imaging. Medical Physics, 2006. 33(6): p. 2137-2138. | Title |
|  | Heffler E, Machetta G, Magnano M, Rolla G. When perennial rhinitis worsens: rhinolith mimicking severe allergic rhinitis. BMJ Case Rep. 2014;2014. | Title |
|  | Heidelberg SR, Heidelberg PM, Istanbul YF, Ulm PW. Examination using modern low dose Multislice-CT (MS-CT) in otorhinolaryngology in a six-week-old baby. Int J Pediatr Otorhinolaryngol. 2016;85:62-4. | Title |
|  | Heiland M, Blessmann M, Pohlenz P, Li L, Schmelzle R, Blake F. Intraoral osteotomies using piezosurgery for distraction in an infant with Pierre-Robin sequence. Clin Oral Investig. 2007;11(3):303-6. | Title |
|  | Heiss, E., et al., Oropharyngeal morphology in the basal tortoise Manouria emys emys with comments on form and function of the testudinid tongue. Journal of Morphology, 2011. 272(10): p. 1217-1229. | Title |
|  | Hellak AF, Kirsten B, Schauseil M, Davids R, Kater WM, Korbmacher-Steiner HM. Influence of maxillary advancement surgery on skeletal and soft-tissue changes in the nose - a retrospective cone-beam computed tomography study. Head Face Med. 2015;11:23. | Title |
|  | Heller M, Schmidt M, Mueller CK, Thorwarth M, Schultze-Mosgau S. Clinical-anthropometric and aesthetic analysis of nose and lip in unilateral cleft lip and palate patients. Cleft Palate-Craniofacial Journal. 2011;48(4):388-93. | Title |
|  | Henderson W, Bollinger J, Xue J, Tien YT, Gelb M. Effect of secreted phospholipase A2 group IID, IIE, and IIF-deficiency on allergen-driven asthma phenotype. European Respiratory Journal. 2013;42. | Title |
|  | Hennessy RJ, Baldwin PA, Browne DJ, Kinsella A, Waddington JL. Frontonasal dysmorphology in bipolar disorder by 3D laser surface imaging and geometric morphometrics: Comparisons with schizophrenia. Schizophrenia Research. 2010;122(1-3):63-71. | Title |
|  | Hennessy RJ, Kinsella A, Waddington JL. 3D laser surface scanning and geometric morphometric analysis of craniofacial shape as an index of cerebro-craniofacial morphogenesis: Initial application to sexual dimorphism. Biological Psychiatry. 2002;51(6):507-14. | Title |
|  | Hennessy RJ, Lane A, Kinsella A, Larkin C, O'Callaghan E, Waddington JL. 3D morphometrics of craniofacial dysmorphology reveals sex-specific asymmetries in schizophrenia. Schizophrenia Research. 2004;67(2-3):261-8. | Title |
|  | Hennessy, R.J., et al., Three-Dimensional Laser Surface Imaging and Geometric Morphometrics Resolve Frontonasal Dysmorphology in Schizophrenia. Biological Psychiatry, 2007. 61(10): p. 1187-1194. | Title |
|  | Hennig M, Schneider D, Bschorer M, Goppold K, Schön G, Bschorer R. Distraction of the mandible-a safe procedure in mild to moderate cases. Journal of Oral and Maxillofacial Surgery. 2017;75(10):e386-e7. | Title |
|  | Hens SM. Ontogeny of craniofacial sexual dimorphism in the orangutan (Pongo pygmaeus). I: Face and palate. American Journal of Primatology. 2005;65(2):149-66. | Title |
|  | Hermann N, Darvann T, Kreiborg S. Incorporating AI for semi-automatic identification of 3D facial landmarks. Cleft Palate-Craniofacial Journal. 2022;59(4 SUPPL):124-5. | Title |
|  | Hernández-Alfaro F, Guijarro-Martínez R, Mareque-Bueno J. Effect of mono- and bimaxillary advancement on pharyngeal airway volume: Cone-beam computed tomography evaluation. Journal of Oral and Maxillofacial Surgery. 2011;69(11):e395-e400. | Title |
|  | Hernández-Alfaro, F., et al., Interpositional collagenated cancellous bone blocks for nasal dorsum augmentation: A new technique for nasomaxillary hypoplasia treatment. Journal of Plastic, Reconstructive and Aesthetic Surgery, 2021. 74(1): p. 223-243. | Title |
|  | Herrera LM, Strapasson RAP, da Silva JVL, Melani RFH. Forensic facial approximation assessment: can application of different average facial tissue depth data facilitate recognition and establish acceptable level of resemblance? Forensic Science International. 2016;266:311-9. | Title |
|  | Herrera LM, Strapasson RAP, Zanin AA, da Silva JVL, Melani RFH. Comparison Among Manual Facial Approximations Conducted by Two Methodological Approaches of Face Prediction. Journal of Forensic Sciences. 2017;62(5):1279-85. | Title |
|  | Herrmann BL, Wessendorf TE, Ajaj W, Kahlke S, Teschler H, Mann K. Effects of octreotide on sleep apnoea and tongue volume (magnetic resonance imaging) in patients with acromegaly. European Journal of Endocrinology. 2004;151(3):309-15. | Title |
|  | Hewson D, Czernicki M. The anaesthetic management of a patient with severe cutaneous systemic sclerosis requiring femoral nailing. Anaesthesia. 2016;71:49. | Title |
|  | Hidaka K, Takeda T, Nakayama M, Yano H. Case of a fractured human bone fragment as an endobronchial foreign body following a traffic incident. BMJ Case Rep. 2018;2018. | Title |
|  | Hierl, T., et al., CAD-CAM-assisted esthetic facial surgery. Journal of Oral and Maxillofacial Surgery, 2013. 71(1): p. e15-e23. | Title |
|  | Hijazi DM, Addas FA, Alghanmi NM, Marzouki HZ, Merdad MA. An Enlarged Goiter Presenting with a Rare Diffuse Lipomatosis of the Thyroid Gland. Am J Case Rep. 2018;19:808-11. | Title |
|  | Hild M, Jaffe AB. Production of 3-D airway organoids from primary human airway basal cells and their use in high-throughput screening. Current Protocols in Stem Cell Biology. 2016;2016:IE.9.1-IE.9.15. | Title |
|  | Hirata RP, Schorr F, Kayamori F, Moriya HT, Romano S, Insalaco G, et al. Upper Airway Collapsibility Assessed by Negative Expiratory Pressure while Awake is Associated with Upper Airway Anatomy. J Clin Sleep Med. 2016;12(10):1339-46. | Title |
|  | Hirsh BA, Greenspon LW. Tracheobronchopathia osteochondroplastica: Rare cause of dyspnea, cough, and bronchitis. American Journal of Respiratory and Critical Care Medicine. 2018;197(MeetingAb). | Title |
|  | Ho CT, Lai HC, Lin HH, Denadai R, Lo LJ. Outcome of full digital workflow for orthognathic surgery planning in the treatment of asymmetric skeletal class III deformity. J Formos Med Assoc. 2021;120(12):2100-12. | Title |
|  | Ho M, Ho M, Julian LM, Stanford WL, Stewart DJ. Disruptive effect of lam-derived smooth muscle cells on human lung progenitors is dependent on expression of translationally controlled tumour protein. American Journal of Respiratory and Critical Care Medicine. 2019;199(9). | Title |
|  | Ho M, Park BY, Rosenblum NG, Al Mukaddam M, Kaplan FS, Kucherov V, et al. Surgical and Radiological Management of Complicated Uterine Leiomyoma Aided by 3D Models in a Patient with Fibrodysplasia Ossificans Progressiva. American Journal of Case Reports. 2021;22. | Title |
|  | Hoang, M.D., A. Saha, and B. McMillan, Chronic odontogenic nasal discharge: report of two cases. Australian Dental Journal, 2021. 66(2): p. 201-204. | Title |
|  | Hodges RJ, Atchison KA, White SC. Impact of cone-beam computed tomography on orthodontic diagnosis and treatment planning. Am J Orthod Dentofacial Orthop. 2013;143(5):665-74. | Title |
|  | Hodges SD, Eck JC, Newton D. Retrospective study of posterior cervical fusions with rhBMP-2. Orthopedics. 2012;35(6):e895-e8. | Title |
|  | Hoegger M, Fischer AJ, McMenimen JD, Ostedgaard LS, Tucker AJ, Awadalla M, et al. Impaired mucus detachment disrupts mucociliary transport in newborn pigs lacking CFTR anion channels. Pediatric Pulmonology. 2014;49:246-7. | Title |
|  | Hoffmann, J., et al., Temporary intermaxillary fixation using individualized acrylic splints permits image-data-based surgery of the lower jaw and oropharynx. Laryngoscope, 2004. 114(8 I): p. 1506-1509. | Title |
|  | Hoffmannova E, Koudelová J, Dupej J, Velemínská J. Age-progression and age-regression face modelling in Czech girls from 6 to 15 years based on three-dimensional longitudinal data. American Journal of Physical Anthropology. 2017;162:219. | Title |
|  | Hohoff, A., et al., Palatal development of preterm and low birthweight infants compared to term infants -- What do we know? Part 3: discussion and conclusion. Head &amp; face medicine, 2005. 1: p. 10. | Title |
|  | Holberg, C., et al., Inter-individual variability of the facial morphology during conscious smiling. Journal of Orofacial Orthopedics, 2006. 67(4): p. 234-243. | Title |
|  | Hollister SJ, Flanagan CL, Morrison RJ, Patel JJ, Wheeler MB, Edwards SP, et al. Integrating Image-Based Design and 3D Biomaterial Printing to Create Patient Specific Devices within a Design Control Framework for Clinical Translation. ACS Biomaterials Science and Engineering. 2016;2(10):1827-36. | Title |
|  | Holmes A.D. et al. Frontoethmoidal encephaloceles: Reconstruction and refinements. Journal Craniofac Surg 2001. 12(1): 6-18. | Title |
|  | Holmgren, E.P., et al., Facial computed tomography use in trauma patients who require a head computed tomogram. Journal of Oral and Maxillofacial Surgery, 2004. 62(8): p. 913-918. | Title |
|  | Holovacs, N.T., et al., You Can't Run, but You Can Hide: The Skeleton of the Sand-Swimmer Lizard Calyptommatus leiolepis (Squamata: Gymnophthalmidae). Anatomical Record, 2020. 303(5): p. 1305-1326. | Title |
|  | Holton N, Yokley T, Butaric L. The Morphological Interaction Between the Nasal Cavity and Maxillary Sinuses in Living Humans. Anatomical Record. 2013;296(3):414-26. | Abstract |
|  | Holton NE, Piche A, Yokley TR. Integration and modularity within the human nasal region. American Journal of Physical Anthropology. 2017;162:221. | Title |
|  | Holton, N.E., A. Piche, and T.R. Yokley, Integration of the nasal complex: Implications for developmental and evolutionary change in modern humans. American Journal of Physical Anthropology, 2018. 166(4): p. 791-802. | Title |
|  | Holton, N.E., T.R. Yokley, and A. Figueroa, Nasal septal and craniofacial form in European- and African-derived populations. Journal of Anatomy, 2012. 221(3): p. 263-274. | Title |
|  | Honda Y, Hata N. Dynamic imaging of swallowing in a seated position using open-configuration MRI. Journal of Magnetic Resonance Imaging. 2007;26(1):172-6. | Title |
|  | Honda, K. and M.K. Tiede. AN MRI STUDY ON THE RELATIONSHIP BETWEEN ORAL CAVITY SHAPE AND LARYNX POSITION. in 5th International Conference on Spoken Language Processing, ICSLP 1998. 1998. | Full text  No skeletal groups |
|  | **Hong JS, Kim DS, Oh KM, Kim YJ, Lee KH, Park YH. Three dimensional analysis of the upper airway and facial morphology in children with Class II malocclusion using cone-beam computed tomography. Korean Journal of Orthodontics. 2010;40(3):134-44.** | **Included** |
|  | **Hong JS, Oh KM, Kim BR, Kim YJ, Park YH. Three-dimensional analysis of pharyngeal airway volume in adults with anterior position of the mandible. Am J Orthod Dentofacial Orthop. 2011;140(4):e161-9.** | **Included** |
|  | Hong JS, Park YH, Kim YJ, Hong SM, Oh KM. Three-dimensional changes in pharyngeal airway in skeletal class III patients undergoing orthognathic surgery. Journal of Oral and Maxillofacial Surgery. 2011;69(11):e401-e8. | Abstract |
|  | Hong Y, Ji W, An S, Han SS, Lee SJ, Kim WJ. Sex differences of COPD phenotypes in nonsmoking patients. International Journal of COPD. 2016;11(1):1657-62. | Title |
|  | Hong Y, Niethammer M, Andruejol J, Kimbell JS, Pitkin E, Superfine R, et al. A pediatric airway atlas and its application in upper airway obstruction. American Journal of Respiratory and Critical Care Medicine. 2013;187. | Abstract |
|  | Honrado, C.P. and W.F. Larrabee Jr, Update in three-dimensional imaging in facial plastic surgery. Current Opinion in Otolaryngology and Head and Neck Surgery, 2004. 12(4): p. 327-331. | Title |
|  | Honrado, C.P., et al., Quantitative assessment of nasal changes after maxillomandibular surgery using a 3-dimensional digital imaging system. Archives of Facial Plastic Surgery, 2006. 8(1): p. 26-35. | Title |
|  | Hood CA, Hosey MT, Bock M, White J, Ray A, Ayoub AF. Facial characterization of infants with cleft lip and palate using a three-dimensional capture technique. Cleft Palate-Craniofacial Journal. 2004;41(1):27-35. | Title |
|  | Hoque T, Srinivasan D, Gnaneswar SAM, Chakravarthi S, Rajaram K. Microimplant Assisted Rapid Palatal Expansion: A Comprehensive Review. Journal of Clinical and Diagnostic Research. 2021;15(8):ZE11-ZE5. | Title |
|  | Horani S, El-Bialy T, Barmak AB, Rossouw PE, Michelogiannakis D. Changes in Airway Dimensions Following Non-extraction Clear Aligner Therapy in Adult Patients with Mild-to-moderate Crowding. J Contemp Dent Pract. 2021;22(3):224-30. | Title |
|  | Horvath L, Thurner M, Jud C, Fink A, Rothen-Rutishauser B. A new approach to engineer an air-blood tissue barrier. Respiration. 2014;87(6):535-6. | Title |
|  | Hosemann W, Gottsauner A, Leuwer A, Farmand M, Wenning W, Göde U, et al. [Fracture healing of the ethmoid bone--a contribution to rhinologic management of naso-ethmoid injuries]. Laryngorhinootologie. 1993;72(8):383-90. | Title |
|  | Hoshi K. Challenge for treatment of congenital maxillofacial anomalies using tissue engineering. Congenital Anomalies. 2019;59(6):A26. | Title |
|  | Hoskens H, Indencleef K, Li J, White JD, Ortega-Castrillon A, Wysocka J, et al. A GWAS on data-driven 3D facial phenotypes selected by matching siblings reveals 310 genetic loci. European Journal of Human Genetics. 2019;27:1158-9. | Title |
|  | Hoskens H, Li JR, Indencleef K, Gors D, Larmuseau MHD, Richmond S, et al. Spatially Dense 3D Facial Heritability and Modules of Co-heritability in a Father-Offspring Design. Frontiers in Genetics. 2018;9. | Title |
|  | Hoskens H, Liu DJ, Naqvi S, Lee MK, Eller RJ, Indencleef K, et al. 3D facial phenotyping by biometric sibling matching used in contemporary genomic methodologies. Plos Genetics. 2021;17(5). | Title |
|  | Hosono C, Matsuda R, Adryan B, Samakovlis C. Transient junction anisotropies orient annular cell polarization in the Drosophila airway tubes. Nature Cell Biology. 2015;17(12):1569-76. | Title |
|  | Hosseinian B, Almaidhan A, Shetye P, Cutting C, Grayson B. A comparative study of 3D nasal shape in unilateral cleft lip and palate noses following rotation-advancement and nam-cutting primary nasal repair. Cleft Palate-Craniofacial Journal. 2015;52(4):e120-e1. | Title |
|  | Houlton, T.M.R. and C. Wilkinson, Facial preservation following extreme mummification: Shrunken heads. Forensic Science International, 2018. 286: p. 31-41. | Title |
|  | Houlton, T.M.R., N. Jooste, and M. Steyn, Mouth Width and Cupid’s Bow Estimation in a Southern African Population. Journal of Forensic Sciences, 2020. 65(2): p. 372-379. | Title |
|  | How SC, McConnell AK, Taylor BJ, Romer LM. Acute and chronic responses of the upper airway to inspiratory loading in healthy awake humans: An MRI study. Respiratory Physiology & Neurobiology. 2007;157(2-3):270-80. | Title |
|  | Howley C, Ali N, Lee R, Cox S. Use of the alar base cinch suture in le Fort i osteotomy: Is it effective? British Journal of Oral and Maxillofacial Surgery. 2011;49(2):127-30. | Title |
|  | Hoxworth, J.M., et al., Radiation dose reduction in paranasal sinus CT using model-based iterative reconstruction. American Journal of Neuroradiology, 2014. 35(4): p. 644-649. | Title |
|  | Hren NI, Barbic U. Tongue volume in adults with skeletal Class III dentofacial deformities. Head & Face Medicine. 2016;12. | Title |
|  | Hsia, C.C.W., Quantitative morphology of compensatory lung growth. European Respiratory Review, 2006. 15(101): p. 148-156. | Title |
|  | Hsieh YJ, Chen YC, Chen YA, Liao YF, Chen YR. Effect of bimaxillary rotational setback surgery on upper airway structure in skeletal class III deformities. Plast Reconstr Surg. 2015;135(2):361e-9e. | Title |
|  | Hsieh YJ, Liao YF, Chen NH, Chen YR. Changes in the calibre of the upper airway and the surrounding structures after maxillomandibular advancement for obstructive sleep apnoea. British Journal of Oral and Maxillofacial Surgery. 2014;52(5):445-51. | Title |
|  | Hsieh, M.H., et al., Matrix Metalloproteinase-1 Polymorphism (-1607G) and Disease Severity in Non-Cystic Fibrosis Bronchiectasis in Taiwan. PLoS ONE, 2013. 8(6). | Title |
|  | Hsu CY, Li YW, Hsu JC. Congenital choanal atresia: computed tomographic and clinical findings. Acta Paediatr Taiwan. 1999;40(1):13-7. | Title |
|  | Hsu, H.A., et al., Effect of recombinant human bone morphogenetic protein-2 and ling Zhi-8 on osteogenesis: A comparative study using a rabbit sinus model. Journal of Oral and Maxillofacial Surgery, 2014. 72(9): p. 1703.e1-1703.e10. | Title |
|  | Hu CH, Wu CT, Ko EWC, Chen PKT. Monobloc Frontofacial or Le Fort III Distraction Osteogenesis in Syndromic Craniosynostosis: Three-Dimensional Evaluation of Treatment Outcome and the Need for Central Distraction. Journal of Craniofacial Surgery. 2017;28(5):1344-9. | Title |
|  | Hu XY, Dong FS, Lu HY, Ma WS, Yuan S. [Establishment of the craniofacial three-dimensional finite element models with the sutures defined alone]. Zhonghua Kou Qiang Yi Xue Za Zhi. 2013;48(10):600-5. | Title |
|  | Hu YA, Ghigliotti L, Vacchi M, Pisano E, Detrich HW, Albertson RC. Evolution in an extreme environment: developmental biases and phenotypic integration in the adaptive radiation of antarctic notothenioids. Bmc Evolutionary Biology. 2016;16. | Title |
|  | Hu YL, Duan FQ, Zhou MQ, Sun YF, Yin BC. Craniofacial reconstruction based on a hierarchical dense deformable model. Eurasip Journal on Advances in Signal Processing. 2012. | Title |
|  | Huang HY, Luo XY, Cheng X, Zhang ZH, Ma GQ, Shi B, et al. Recapitulation of Unilateral Cleft Lip Nasal Deformity on Normal Nasal Structure: A Finite Element Model Analysis. Journal of Craniofacial Surgery. 2018;29(8):2220-5. | Title |
|  | Huang Y, An Y, Wang J. In vivo evaluations of neck skin elasticity and its role in regulating upper airway patency. American Journal of Respiratory and Critical Care Medicine. 2015;191. | Title |
|  | Huang Y, Zhang X. Correlations between upper airway patency and neck fat growth: Results from a computer model. American Journal of Respiratory and Critical Care Medicine. 2012;185. | Title |
|  | Huang, J., C.Y. Li, and J.H. Jiang, Facial soft tissue changes after nonsurgical rapid maxillary expansion: A systematic review and meta-analysis. Head and Face Medicine, 2018. 14(1). | Title |
|  | Huang, J.K., et al., Congenital nasal pyriform aperture stenosis and single central maxillary incisor: Preoperative evaluation with three-dimensional computed tomography. Journal of the Formosan Medical Association, 2004. 103(1): p. 37-40. | Title |
|  | Huang, S.L., et al., Fourth branchial cleft deformity with skin orifice: a series of 10 cases. Zhonghua er bi yan hou tou jing wai ke za zhi = Chinese journal of otorhinolaryngology head and neck surgery, 2016. 51(10): p. 776-779. | Title |
|  | Huang, S.Y., et al., Sonographic diagnosis of fetal malformations associated with mycophenolate mofetil exposure in utero. American Journal of Obstetrics and Gynecology, 2008. 199(2): p. e6-e8. | Title |
|  | Huang, W., J. Ye, and X. Guan, Standard-dose versus low-dose multidetector computed tomography examinations in patients with uncontrolled chronic rhinosinusitis. Medicine (United States), 2018. 97(50). | Title |
|  | Huang, Y., et al. Automatic landmark detection and norid registration of intra-subject lung CT images. in 2009 1st International Conference on Information Science and Engineering, ICISE 2009. 2009. | Title |
|  | Hubbard AM, Harty MP. MRI for the assessment of the malformed fetus. Best Practice & Research Clinical Obstetrics & Gynaecology. 2000;14(4):629-50. | Title |
|  | Huckabee ML, Macrae P, Lamvik K. Expanding Instrumental Options for Dysphagia Diagnosis and Research: Ultrasound and Manometry. Folia Phoniatrica Et Logopaedica. 2015;67(6):269-84. | Title |
|  | Hulsey CD, Meyer A, Streelman JT. Convergent Evolution of Cichlid Fish Pharyngeal Jaw Dentitions in Mollusk-Crushing Predators: Comparative X-Ray Computed Tomography of Tooth Sizes, Numbers, and Replacement. Integrative and Comparative Biology. 2020;60(3):656-64. | Title |
|  | Hulsey CD, Roberts RJ, Lin ASP, Guldberg R, Streelman JT. Convergence in a mechanically complex phenotype: Detecting structural adaptations for crushing in cichlid fish. Evolution. 2008;62(7):1587-99. | Title |
|  | Humphries LS, Yates EK, Mhlaba JM, Collins JM, Baroody FM, Reid RR. Airway Volume Simulation in Virtual Mandibular Distraction: A Cohort Study. Plastic and Reconstructive Surgery. 2018;141(4):1003-9. | Title |
|  | Humphries S, DeBoer EM, Hunter K, Shandas R, Deterding RR. Analysis of pediatric airway morphology using statistical shape modeling. American Journal of Respiratory and Critical Care Medicine. 2013;187. | Abstract |
|  | Hun, K.D., et al., The relationship between facial asymmetry and nasal septal deviation. Journal of Craniofacial Surgery, 2015. 26(4): p. 1273-1276. | Title |
|  | Hussain SS, Bodduluri S, Kim H, Brand JD, Li Q, Foote J, et al. Progressive Airway Obstruction and Airway Loss Is Associated with Apoptosis in a Ferret Model of COPD and Chronic Mucus Stasis. Am Journal of Respiratory and Critical Care Medicine. 2021;203(9). | Title |
|  | Husson AH, Burhan AS, Hajeer MY, Nawaya FR. Three-dimensional oropharyngeal airway changes after facemask therapy using low-dose computed tomography: a clinical trial with a retrospectively collected control group. Progress in Orthodontics. 2021;22(1). | Title |
|  | Hwang H, Hwang C, West J, Singh GD. Changes in pediatric paranasal sinuses following biomimetic oral appliance therapy: 3 case reports. Cranio. 2022;40(2):160-5. | Title |
|  | Hwang, J.O., A dynamic model of technological innovation in 3D TV industry: Case of LG electronics, in Lecture Notes in Electrical Engineering. 2014. p. 375-382. | Title |
|  | Hyde, D.M., N.K. Tyler, and C.G. Plopper, Morphometry of the Respiratory Tract: Avoiding the Sampling, Size, Orientation, and Reference Traps. Toxicologic Pathology, 2007. 35(1): p. 41-48. | Title |
|  | Hyman AJ, Fastenberg JH, Stupak HD. Orientation of the premaxilla in the origin of septal deviation. Eur Arch Otorhinolaryngol. 2019;276(11):3147-51. | Title |
|  | Hyodo, M., et al., Scanning electron microscopic study of the muscle fiber arrangement in the rat cricopharyngeal muscle. Acta Oto-Laryngologica, 2005. 125(9): p. 976-980. | Title |
|  | Iannetti G, Polimeni A, Pagnoni M, Fadda MT, Ramieri V, Tecco S, et al. Upper airway volume after Le Fort III advancement in subjects with craniofacial malformation. J Craniofac Surg. 2011;22(1):351-5. | Title |
|  | Ibrahim N, Wooles NR, Elloy M, Da Forno P. A hairy situation. BMJ Case Rep. 2015;2015. | Title |
|  | Idso S, Holloway J, Patel P, Linping Z, Forbes D, Liu D. Airway changes in patients with unilateral cleft lip/palate (UCL/P) after maxillary advancement. Cleft Palate-Craniofacial Journal. 2021;58(4 SUPPL):32-3. | Title |
|  | Iglesias J, Carrasco A, Catalán G, De la Fuente P, Gutiérrez R, Paternain C, et al. Intraventricular haemorrhage by nasogastric tube insertion into the brain after transsphenoidal surgery. A case report and review of the literature. Interdisciplinary Neurosurgery: Advanced Techniques and Case Management. 2021;24. | Title |
|  | Ihemeremadu N, Geng E, Keenan B, Zang Y, Schwab RJ. Upper airway and craniofacial structural differences between male and female caucasian patients with obstructive sleep apnea using magnetic resonance imaging. American Journal of Respiratory and Critical Care Medicine. 2020;201(1). | Title |
|  | Ihemeremadu N, Lavi-Romer N, Zang Y, Keenan B, Schwab R. Evaluating differences in upper airway anatomy between diabetic and non-diabetic OSA patients. Sleep. 2020;43(SUPPL 1):A401-A2. | Title |
|  | Ikeda A, Ikeda M, Komatsuzaki A. A CT study of the course of growth of the maxillary sinus: Normal subjects and subjects with chronic sinusitis. ORL. 1998;60(3):147-52. | Title |
|  | Ikeda, A., A. Komatsuzaki, and M. Ikeda, A study of the distribution pattern and correlation between maxillary, frontal and sphenoid sinus volume. Practica Otologica, 1997. 90(11): p. 1235-1239. | Abstract |
|  | Ikeda, T. and T. Iinuma, Surgical Anatomy of the Sphenoid Sinus-Development and Intersinus Septum. Nippon Jibiinkoka Gakkai Kaiho, 1992. 95(2): p. 214-223. | Title |
|  | Imaizumi K, Taniguchi K, Ogawa Y, Matsuzaki K, Maekawa H, Nagata T, et al. Development of three-dimensional facial approximation system using head CT scans of Japanese living individuals. Journal of Forensic Radiology and Imaging. 2019;17:36-45. | Title |
|  | Imamura, M., et al., Celecoxib as a potential treatment for intractable lymphatic malformation. Pediatrics, 2019. 144(3). | Title |
|  | Inada E, Saitoh I, Kaihara Y, Murakami D, Nogami Y, Kubota N, et al. Incompetent lip seal affects the form of facial soft tissue in preschool children. Cranio-the Journal of Craniomandibular & Sleep Practice. 2021;39(5):405-11. | Title |
|  | Indencleef K, Roosenboom J, Hoskens H, White JD, Shriver MD, Richmond S, et al. Six NSCL/P Loci Show Associations With Normal-Range Craniofacial Variation. Frontiers in Genetics. 2018;9. | Title |
|  | **Indriksone I, Jakobsone G. The influence of craniofacial morphology on the upper airway dimensions. Angle Orthod. 2015;85(5):874-80.** | **Included** |
|  | Infeld MD, Brennan JA, Davis PB. HUMAN TRACHEOBRONCHIAL EPITHELIAL-CELLS DIRECT MIGRATION OF LUNG FIBROBLASTS IN 3-DIMENSIONAL COLLAGEN GELS. American Journal of Physiology. 1992;262(5):L535-L41. | Title |
|  | Inoshita, A., et al., Sex differences in the development of upper airway morphology: Is this the new kid on the block? Journal of Thoracic Disease, 2019. 11: p. S2032-S2033. | Title |
|  | Inoue K, Hosokawa S, Sato N, Sakakibara M, Ohashi K, Sugiyama T. Relationship between malocclusion and airway volume of children in the mixed dentition period. Pediatric Pulmonology. 2019;54:S116. | Abstract |
|  | Inouye J, Pelland C, Lin KYK, Borowitz K, Blemker S. Three-dimensional computer simulations demonstrate that increasing surgical overlap of the levator veli palatini improves velopharyngeal closure. Cleft Palate-Craniofacial Journal. 2014;51(3):e51-e2. | Title |
|  | Inouye JM, Pelland CM, Lin KY, Borowitz KC, Blemker SS. A Computational Model of Velopharyngeal Closure for Simulating Cleft Palate Repair. Journal of Craniofacial Surgery. 2015;26(3):658-62. | Title |
|  | Ioi, W.K., et al., Bipartition osteotomy for treating orbital hypertelorism with maxillofacial malformation. Chinese Journal of Plastic Surgery, 2022. 38(4): p. 418-424. | Title |
|  | Irani SK, Oliver DR, Movahed R, Kim YI, Thiesen G, Kim KB. Pharyngeal airway evaluation after isolated mandibular setback surgery using cone-beam computed tomography. Am J Orthod Dentofacial Orthop. 2018;153(1):46-53. | Title |
|  | Irct201204244303N. Evaluation of dental & skeletal changes following surgically assisted maxillary expansion with or without pterygomaxillary disjunction, and their long-term stability. https://trialsearchwhoint/Trial2aspx?TrialID=IRCT201204244303N2. 2012. | Title |
|  | Isaac M, ElSaieed M, ElSobky A, Aboelmaaty W. Correlation between radiographic findings of CBCT in OSA patients and their AHI value. International Journal of Computer Assisted Radiology and Surgery. 2020;15(1):S132-S3. | Title |
|  | Işeri H, Tekkaya AE, Oztan O, Bilgiç S. Biomechanical effects of rapid maxillary expansion on the craniofacial skeleton, studied by the finite element method. Eur J Orthod. 1998;20(4):347-56. | Title |
|  | Ishijima K, Sando I, Balaban C, Suzuki C, Takasaki K. Length of the eustachian tube and its postnatal development: Computer-aided three-dimensional reconstruction and measurement study. Annals of Otology Rhinology and Laryngology. 2000;109(6):542-8. | Title |
|  | Ishijima, K., et al., Functional anatomy of levator veli palatini muscle and tensor veli palatini muscle in association with eustachian tube cartilage. Annals of Otology, Rhinology and Laryngology, 2002. 111(6): p. 530-536. | Title |
|  | Ishijima, K., et al., Length of the eustachian tube and its postnatal development: Computer- aided three-dimensional reconstruction and measurement study. Annals of Otology, Rhinology and Laryngology, 2000. 109(6): p. 542-548. | Title |
|  | Ishikawa, S., K. Ishimori, and S. Ito, A 3D epithelial-mesenchymal co-culture model of human bronchial tissue recapitulates multiple features of airway tissue remodeling by TGF-β1 treatment. Respiratory Research, 2017. 18(1). | Title |
|  | Isidor S, Di Carlo G, Cornelis MA, Isidor F, Cattaneo PM. Three-dimensional evaluation of changes in upper airway volume in growing skeletal Class II patients following mandibular advancement treatment with functional orthopedic appliances. Angle Orthod. 2018;88(5):552-9. | Title |
|  | Isrctn. A prospective randomised clinical trial to assess change in nasal soft tissue dimensions in orthognathic surgery evaluated using a 3D imaging system. https://trialsearchwhoint/Trial2aspx?TrialID=ISRCTN38986023. 2007. | Title |
|  | Isrctn. NutriBrain: a controlled study to investigate the effect of a food supplement on brain development in very early born infants. https://trialsearchwhoint/Trial2aspx?TrialID=ISRCTN96620855. 2017. | Title |
|  | Ito H, Koshizuka S, Shino R, Haga A, Onoe T, Nakagawa K. Quasi-4DCT images based on physics-based lung deformation simulation. Radiotherapy and Oncology. 2011;99:S484-S5. | Title |
|  | Ito T, Nishimura TD, Ebbestad JOR, Takai M. Computed tomography examination of the face of Macaca anderssoni (Early Pleistocene, Henan, northern China): Implications for the biogeographic history of Asian macaques. Journal of Human Evolution. 2014;72:64-80. | Title |
|  | Ito T, Nishimura TD, Hamada Y, Takai M. Contribution of the maxillary sinus to the modularity and variability of nasal cavity shape in Japanese macaques. Primates. 2015;56(1):11-9. | Title |
|  | Ito T, Nishimura TD, Senut B, Koppe T, Treil J, Takai M. Reappraisal of Macaca speciosa subfossilis from the Late Pleistocene of Northern Vietnam Based on the Analysis of Cranial Anatomy. International Journal of Primatology. 2009;30(5):643-62. | Title |
|  | Ito T, Nishimura TD. Enigmatic Diversity of the Maxillary Sinus in Macaques and Its Possible Role as a Spatial Compromise in Craniofacial Modifications. Evolutionary Biology. 2016;43(3):414-26. | Title |
|  | Iwai K, Ueda H, Nagasaki T, Medina CC, Matsumura Y, Tanimoto K. Multislice computed tomography assessment of airway patency changes in the supine position following mandibular setback surgery for mandibular prognathism: A prospective 1-year follow-up study. Apos Trends in Orthodontics. 2021;11(2):106-15. | Title |
|  | Iwai T, Yajima Y, Honda K, Murata S, Omura S, Tohnai I. Computational fluid dynamic simulation of pharyngeal airway before and after mandibular setback surgery. International Journal of Oral and Maxillofacial Surgery. 2017;46:323-4. | Title |
|  | Iwasaki LR, Covell DA, Frazier-Bowers SA, Huja SS, Kapila S, Nickel JC. Preface to COAST 2018 Innovators' Workshop: Bridging the biology and technology gap in orthodontics and craniofacial care. Orthodontics & Craniofacial Research. 2019;22:5-7. | Title |
|  | **Iwasaki T, Hayasaki H, Takemoto Y, Kanomi R, Yamasaki Y. Oropharyngeal airway in children with Class III malocclusion evaluated by cone-beam computed tomography. Am J Orthod Dentofacial Orthop. 2009;136(3):318.e1-9; discussion -9.** | **Included** |
|  | Iwasaki T, Papageorgiou SN, Yamasaki Y, Ali Darendeliler M, Papadopoulou AK. Nasal ventilation and rapid maxillary expansion (RME): a randomized trial. European journal of orthodontics. 2021;43(3):283‐92. | Title |
|  | Iwasaki T, Saitoh I, Takemoto Y, Inada E, Kanomi R, Hayasaki H, et al. Evaluation of upper airway obstruction in Class II children with fluid-mechanical simulation. Am J Orthod Dentofacial Orthop. 2011;139(2):e135-45. | Full text  Only Class IIs |
|  | Iwasaki T, Sato H, Suga H, Minami A, Yamamoto Y, Takemoto Y, et al. Herbst appliance effects on pharyngeal airway ventilation evaluated using computational fluid dynamics. Angle Orthod. 2017;87(3):397-403. | Title |
|  | **Iwasaki T, Sato H, Suga H, Takemoto Y, Inada E, Saitoh I, et al. Relationships among nasal resistance, adenoids, tonsils, and tongue posture and maxillofacial form in Class II and Class III children. Am J Orthod Dentofacial Orthop. 2017;151(5):929-40.** | **Included** |
|  | Iwasaki T, Sato H, Suga H, Takemoto Y, Inada E, Saitoh I, et al. Influence of pharyngeal airway respiration pressure on Class II mandibular retrusion in children: A computational fluid dynamics study of inspiration and expiration. Orthod Craniofac Res. 2017;20(2):95-101. | Abstract |
|  | **Iwasaki T, Suga H, Yanagisawa-Minami A, Sato H, Sato-Hashiguchi M, Shirazawa Y, et al. Relationships among tongue volume, hyoid position, airway volume and maxillofacial form in paediatric patients with Class-I, Class-II and Class-III malocclusions. Orthod Craniofac Res. 2019;22(1):9-15.** | **Included** |
|  | Iwasaki T, Takemoto Y, Inada E, Sato H, Saitoh I, Kakuno E, et al. Three-dimensional cone-beam computed tomography analysis of enlargement of the pharyngeal airway by the Herbst appliance. Am J Orthod Dentofacial Orthop. 2014;146(6):776-85. | Title |
|  | Jacob A, Tward DJ, Resnick S, Smith PF, Lopez C, Rebello E, et al. Vestibular function and cortical and sub-cortical alterations in an aging population. Heliyon. 2020;6(8). | Title |
|  | **Jadhav M, Bhosale V, Patil A, Shinde S. Comparison of Volumetric Dimensions of Pharyngeal Airway for Different Dentofacial Skeletal Patterns Using Cone Beam Computed Tomography. Folia Med (Plovdiv). 2020;62(3):572-7.** | **Included** |
|  | Jadhav MA, Bhosale VI, Jadhav AB. To compare volumetric dimensions of pharyngeal airway for different dentofacial skeletal patterns - Cone beam computed tomography. Journal of the International Clinical Dental Research Organization. 2019;11(2):83-9. | Full text  No volumes |
|  | Jadhav VV, Daigavane P, Kamble R, Shrivastav S, Tiwari M. Broadening the Scope and Utility of Frontal Sinus Morphology for Predicting the Growth Pattern and Skeletal Malocclusion in Cleft Lip and Palate Cases. Journal of Pharmaceutical Research International. 2021;33(51A):173-9. | Title |
|  | Jadu, F.M., Number and nature of incidental findings in cone-beam computed tomography made for oral surgery purposes. World Journal of Dentistry, 2019. 10(5): p. 390-392. | Title |
|  | Jafari, A., K.S. Shetty, and M. Kumar, Study of stress distribution and displacement of various craniofacial structures following application of transverse orthopedic forces - A three-dimensional FEM study. Angle Orthodontist, 2003. 73(1): p. 12-20. | Title |
|  | Jafari-Pozve N, Kiani H, Aryanezhad SS. Comparing dimensions of upper airway in patients with nasal septal deviation and healthy people in CBCT. American Journal of Nuclear Medicine and Molecular Imaging. 2022;12(1):33-40. | Abstract |
|  | Jahanbin A, Mahdavishahri N, Baghayeripour M, Esmaily H, Eslami N. Evaluation of Facial Anthropometric Parameters in 11-17 Year Old Boys. Journal of Clinical Pediatric Dentistry. 2012;37(1):95-101. | Title |
|  | Jahanbin A, Poosti M, Rashed R, Sharifi V, Bozorgnia Y. Evaluation of nasomaxillary growth of adolescent boys in northeastern iran. Acta Medica Iranica. 2012;50(10):684-8. | Title |
|  | Jahanbin, A., et al., Evaluation of some facial anthropometric parameters in an Iranian population: Infancy through adolescence. Journal of Craniofacial Surgery, 2013. 24(3): p. 941-945. | Title |
|  | Jain K, Jain VK, Aggarwal K, Bansal A. Late onset isotretinoin resistant acne conglobata in a patient with acromegaly. Indian J Dermatol Venereol Leprol. 2008;74(2):139-41. | Title |
|  | Jakkalavadike Bhaktavatsalam B, Iyer D, Volikas I. Anaesthetic management for caesarean section in a pregnant woman with achondroplasia. Anaesthesia. 2020;75:73. | Title |
|  | Jakobsone G, Neimane L, Krumina G. Two- and three-dimensional evaluation of the upper airway after bimaxillary correction of Class III malocclusion. Oral Surg Oral Med Oral Pathol Oral Radiol Endod. 2010;110(2):234-42. | Title |
|  | Jandzik D, Hawkins MB, Cattell MV, Cerny R, Square TA, Medeiros DM. Roles for FGF in lamprey pharyngeal pouch formation and skeletogenesis highlight ancestral functions in the vertebrate head. Development. 2014;141(3):629-38. | Title |
|  | Jang SI, Ahn J, Paeng JY, Hong J. Three-dimensional analysis of changes in airway space after bimaxillary orthognathic surgery with maxillomandibular setback and their association with obstructive sleep apnea. Maxillofac Plastic Reconstr Surg 2018;40(1). | Title |
|  | Jang, K.S., et al., A three-dimensional photogrammetric analysis of the facial esthetics of the miss Korea pageant contestants. Korean Journal of Orthodontics, 2017. 47(2): p. 87-99. | Title |
|  | Janner SFM, Dubach P, Suter VGA, Caversaccio MD, Buser D, Bornstein MM. Sinus floor elevation or referral for further diagnosis and therapy: A comparison of maxillary sinus assessment by ENT specialists and dentists using cone beam computed tomography. Clinical Oral Implants Research. 2020;31(5):463-75. | Title |
|  | Janoff S, Marshall H, Deppe M, Billings C, Parra-Robles J, Hillis S, et al. Comparing hyperpolarised 3He MRI ventilated lung volumes with spirometry in COPD. Journal of Thoracic Imaging. 2012;27(5):W124. | Title |
|  | Janoff S, Marshall H, Deppe M, Billings C, Parra-Robles J, Hillis S, et al. Quantifying lung function in COPD with hyperpolarised 3He MRI. European Respiratory Journal. 2012;40. | Title |
|  | Jansen CE. CBCT technology for diagnosis and treatment planning: what general practitioners should consider. Compend Contin Educ Dent. 2014;35(10):749-53; quiz 54, 56. | Title |
|  | Jaw, T.S., et al., Development of adenoids: a study by measurement with MR images. The Kaohsiung journal of medical sciences, 1999. 15(1): p. 12-18. | Title |
|  | Jayakumar N, Saravanamuthu T, Balasubramaniam C, Singaravelu R. Unique mishap following transfacial pin fixated midface distraction and successful management using nasal endoscopy. BMJ Case Rep. 2019;12(12). | Title |
|  | Jayaratne YS, McGrath CP, Zwahlen RA. How accurate are the fusion of cone-beam CT and 3-D stereophotographic images? PLoS One. 2012;7(11):e49585. | Title |
|  | **Jayaratne YS, Zwahlen RA. The Oropharyngeal Airway in Young Adults with Skeletal Class II and Class III Deformities: A 3-D Morphometric Analysis. PLoS One. 2016;11(2):e0148086.** | **Included** |
|  | Jeffery N. Cranial base angulation and growth of the human fetal pharynx. Anatomical Record - Part A Discoveries in Molecular, Cellular, and Evolutionary Biology. 2005;284(1):491-9. | Title |
|  | Jeffery, N., et al., Craniofacial growth in fetal Tarsius bancanus: Brains, eyes and nasal septa. Journal of Anatomy, 2007. 210(6): p. 703-722. | Title |
|  | Jennings R, Parton C, Clark F. Tension gastrothorax-the lessons learnt. Journal of the Intensive Care Society. 2020;21(2 SUPPL):85. | Title |
|  | Jensen, O.T., et al., Trans-sinus dental implants, bone morphogenetic protein 2, and immediate function for all-on-4 treatment of severe maxillary atrophy. Journal of Oral and Maxillofacial Surgery, 2012. 70(1): p. 141-148. | Title |
|  | Jeon JY, Kim TS, Kim SY, Park CJ, Hwang KG. Does the Pharyngeal Airway Recover After Sagittal Split Ramus Osteotomy for Mandibular Prognathism? Journal of Oral and Maxillofacial Surgery. 2016;74(1):162-9. | Title |
|  | Jeong HR, Roh JY, Park SE, Baek SH, Kim SJ. Correlation between nasomaxillary complex dimensional changes and respiratory improvement after maxillary skeletal expansion treatment in pediatric obstructive sleep apnea patients. Sleep Med 2022;100:S58-S9. | Title |
|  | Jeong S, Sung J, Kim S, Kim Y, Shin S, Kim SS. Upper airway morphologic changes after mandibular setback surgery in skeletal class III malocclusion patients measured using cone beam computed tomography superimposition. International Journal of Oral and Maxillofacial Surgery. 2018;47(11):1405-10. | Title |
|  | Jeong, W.S., et al., Change in Posterior Pharyngeal Space after Counterclockwise Rotational Orthognathic Surgery for Class II Dentofacial Deformity Diagnosed with Obstructive Sleep Apnea Based on Cephalometric Analysis. Journal of Craniofacial Surgery, 2017. 28(5): p. e488-e491. | Title |
|  | Jesus AS, Oliveira CB, Murata WH, Goncales ES, Pereira VA, Santos-Pinto A. Nasomaxillary effects of miniscrew-assisted rapid palatal expansion and two surgically assisted rapid palatal expansion approaches. International Journal of Oral and Maxillofacial Surgery. 2021;50(8):1059-68. | Title |
|  | Ji, Y., et al., Effect of navigation system on removal of foreign bodies in head and neck surgery. Journal of Craniofacial Surgery, 2018. 29(7): p. e723-e726. | Title |
|  | Jia L, Qi B, Yang J, Zhang W, Lu Y, Zhang HL. Ultrasonic measurement of facial tissue depth in a Northern Chinese Han population. Forensic Science International. 2016;259:247.e1-.e6. | Title |
|  | Jiang JX, Matsugi K, Sasaki G, Yanagisawa O. Resistivity study of eutectoid decomposition kinetics of alpha-Fe2Si5 alloy. Materials Transactions. 2005;46(3):720-5. | Title |
|  | Jiang XY, Lambers M, Bunke H. Structural performance evaluation of curvilinear structure detection algorithms with application to retinal vessel segmentation. Pattern Recognition Letters. 2012;33(15):2048-56. | Title |
|  | Jiang, H., et al., Age-related changes of normal adult inferior rectus muscle: Analysis with dynamic contrast-enhanced magnetic resonance imaging. National Medical Journal of China, 2011. 91(27): p. 1899-1903. | Title |
|  | Jiang, Y.Y., Correlation between hyoid bone position and airway dimensions in Chinese adolescents by cone beam computed tomography analysis. International Journal of Oral and Maxillofacial Surgery, 2016. 45(7): p. 914-921. | Full text  Only Class I |
|  | Jin D, Iyer KS, Chen C, Hoffman EA, Saha PK. A robust and efficient curve skeletonization algorithm for tree-like objects using minimum cost paths. Pattern Recognition Letters. 2016;76:32-40. | Title |
|  | Jin YL, Jiang X. [Foreign bodies of external nose and forehead:a case report]. Lin Chung Er Bi Yan Hou Tou Jing Wai Ke Za Zhi. 2016;30(24):1973-4. | Title |
|  | Jin, D., et al. A new approach of arc skeletonization for tree-like objects using minimum cost path. in Proceedings - International Conference on Pattern Recognition. 2014. | Title |
|  | Jing XF, Yang M, Liu Y, Wang Y, Li JJ, Hu W. Associations of Trunk Muscle Mass and Muscle Quality Indicators with Self-Reported Dysphagia in Older Inpatients. Dysphagia. | Title |
|  | Jodeh DS, Ross JM, Leszczynska M, Qamar F, Dawkins RL, Cray JJ, et al. Determination of Ethnic Variation in Infant Nasolabial Anthropometry Using 3D Photographs: Implications for Bilateral Cleft Lip Nasal Correction. Cleft Palate-Craniofacial Journal. 2022;59(6):693-700. | Title |
|  | Jodeh, D.S., et al., Degree of Asymmetry Between Patients With Complete and Incomplete Cleft Lips. Cleft Palate-Craniofacial Journal, 2021. 58(5): p. 539-545. | Title |
|  | Johns J, Hill J, Kan M, Horn JL. Infraclavicular nerve block in a patient with amyotrophic lateral sclerosis. Regional Anesthesia and Pain Medicine. 2016;41(5). | Title |
|  | Johnson NJ, Maher PJ, Badulak J, Luks AM. The Transition From Emergency Medicine Resident to Critical Care Fellow: A Road Map. Aem Education and Training. 2017;1(2):116-23. | Title |
|  | Johnson, A., et al., Examination of forensic entomology evidence using computed tomography scanning: Case studies and refinement of techniques for estimating maggot mass volumes in bodies. International Journal of Legal Medicine, 2012. 126(5): p. 693-702. | Title |
|  | Jones NS. Current concepts in the management of paediatric rhinosinusitis. J Laryngol Otol. 1999;113(1):1-9. | Title |
|  | Jones, N.S., A. Strobl, and I. Holland, A study of the CT findings in 100 patients with rhinosinusitis and 100 controls. Clinical Otolaryngology and Allied Sciences, 1997. 22(1): p. 47-51. | Title |
|  | Joshi, M., et al., Sagittal lip positions in different skeletal malocclusions: a cephalometric analysis. Prog Orthod 2015. 16(1). | Title |
|  | Joy A, Park J, Chambers DW, Oh H. Airway and cephalometric changes in adult orthodontic patients after premolar extractions. Angle Orthod. 2020;90(1):39-46. | Title |
|  | Joy R, Edwards S, Gupta D, Afzali P, Conley RS, Kelly KA, et al. Evaluation of airway and obstructive sleep apnoea in cleft lip and cleft palate adolescents using cone-beam computed tomography-a retrospective study. International Journal of Oral and Maxillofacial Surgery. 2017;46:64-5. | Title |
|  | Jprn U. Anesthetic preconditioning effects of desflurane versus propofol on tourniquet induced ischemia-reperfusion injury during anterior cruciate ligament reconstruction:a single-centre randomized controlled study. https://trialsearchwhoint/Trial2aspx?TrialID=JPRN-UMIN000033217. 2018. | Title |
|  | Jprn U. Study on the volume of upper airway for orthodontic treatment patients. https://trialsearchwhoint/Trial2aspx?TrialID=JPRN-UMIN000025148. 2018. | Abstract |
|  | Jung, B.A., et al., Vertical palatal bone dimensions on lateral cephalometry and cone-beam computed tomography: Implications for palatal implant placement. Clinical Oral Implants Research, 2011. 22(6): p. 664-668. | Title |
|  | Jung, J., et al., Three dimensional evaluation of soft tissue after orthognathic surgery. Head and Face Medicine, 2018. 14(1). | Title |
|  | Kabalan O, Gordon J, Heo G, Lagravère MO. Nasal airway changes in bone-borne and tooth-borne rapid maxillary expansion treatments. Int Orthod. 2015;13(1):1-15. | Title |
|  | Kaban LB. Diagnosis and treatment of fractures of the facial bones in children 1943-1993. J Oral Maxillofac Surg. 1993;51(7):722-9. | Title |
|  | Kablan A, Mat B, Temel SG, Alanay Y. Ischiospinal Dysostosis in a boy with a novel homozygous missense mutation in the BMPER gene. European Journal of Human Genetics. 2019;27:916-7. | Title |
|  | Kachlan MO, Yang J, Balshi TJ, Wolfinger GJ, Balshi SF. Incidental Findings in Cone Beam Computed Tomography for Dental Implants in 1002 Patients. J Prosthodont. 2021;30(8):665-75. | Title |
|  | Kaczmarek P, Hermyt M, Rupik W. Embryology of the VNO and associated structures in the grass snake Natrix natrix (Squamata: Naticinae): a 3D perspective. Frontiers in Zoology. 2017;14. | Title |
|  | Kaipainen AE, Sieber KR, Nada RM, Maal TJ, Katsaros C, Fudalej PS. Regional facial asymmetries and attractiveness of the face. European Journal of Orthodontics. 2016;38(6):602-8. | Title |
|  | Kajisa E, Tohara H, Nakane A, Wakasugi Y, Hara K, Yamaguchi K, et al. The relationship between jaw-opening force and the cross-sectional area of the suprahyoid muscles in healthy elderly. Journal of Oral Rehabilitation. 2018;45(3):222-7. | Title |
|  | Kalaskar, R., S. Balasubramanian, and A. Kalaskar, Evaluation of the average nasal and nasopharyngeal volume in 10–13-year-old children: A preliminary CBCT study. International Journal of Clinical Pediatric Dentistry, 2021. 14(2): p. 187-191. | Full text  No skeletal groups |
|  | Kale, B. and M.H. Buyukcavus, Comparison of three-dimensional soft-tissue evaluations between skeletal and pseudo-class III malocclusions. Scientific Reports, 2020. 10(1). | Title |
|  | Kalmár T, Maróti Z, Zimmermann A, Sztriha L. Tremor as an early sign of hereditary spastic paraplegia due to mutations in ALDH18A1. Brain and Development. 2021;43(1):144-51. | Title |
|  | Kamano, E., et al., Three-dimensional analysis of changes in pharyngeal airway space after mandibular setback surgery. Orthodontic Waves, 2017. 76(1): p. 1-8. | Title |
|  | Kamizono, K.I., et al., Volumetric changes of transferred free anterolateral thigh flaps in head and neck lesions. Auris Nasus Larynx, 2021. 48(4): p. 751-757. | Title |
|  | Kamnasaran, D., F. Morin, and J. Gekas, Prenatal diagnosis and molecular genetic studies on a new case of agnathia-otocephaly. Fetal and Pediatric Pathology, 2010. 29(4): p. 207-211. | Title |
|  | Kan Z, Li DC, Shen T, Xiang JW, Zhang L. Aerodynamic characteristics of morphing wing with flexible leading-edge. Chinese Journal of Aeronautics. 2020;33(10):2610-9. | Title |
|  | Kane AA, DeLeon VB, Valeri C, Becker DB, Richtsmeier JT, Lo LJ. Preoperative osseous dysmorphology in unilateral complete cleft lip and palate: A quantitative analysis of computed tomography data. Plastic and Reconstructive Surgery. 2007;119(4):1295-301. | Title |
|  | Kang JH. Associations Among Temporomandibular Joint Osteoarthritis, Airway Dimensions, and Head and Neck Posture. Journal of Oral and Maxillofacial Surgery. 2020;78(12):2183.e1-.e12. | Title |
|  | Kang NE, Lee DH, In Seo J, Lee JK, Song SI. Postoperative changes in the pharyngeal airway space through computed tomography evaluation after mandibular setback surgery in skeletal class III patients: 1-year follow-up. Maxillofacial Plastic and Reconstructive Surgery. 2021;43(1). | Title |
|  | Kang NH. Current Methods for the Treatment of Alveolar Cleft. Archives of Plastic Surgery-Aps. 2017;44(3):188-93. | Title |
|  | Kang TJ, Eo SH, Cho H, Donatelli RE, Lee SJ. A sparse principal component analysis of Class III malocclusions. Angle Orthod. 2019;89(5):768-74. | Title |
|  | Kang Y, Lee S, Gong Y, Kim SH, Moon CH. Three-dimensional morphologic evaluation of the changes in the pharyngeal airway and hyoid bone after bimaxillary surgery in patients with skeletal Class III malocclusion with facial asymmetry: A preliminary study. Am J Orthod Dentofacial Orthop. 2022;162(1):42-50. | Title |
|  | Kano, S., Genomics and developmental approaches to an ascidian adenohypophysis primordium. Integrative and Comparative Biology, 2010. 50(1): p. 35-52. | Title |
|  | Kanuch P, Aghova T, Meheretu Y, Sumbera R, Bryja J. New discoveries on the ecology and echolocation of the heart-nosed bat Cardioderma cor with a contribution to the phylogeny of Megadermatidae. African Zoology. 2015;50(1):53-7. | Title |
|  | Kapila SD, Nervina JM. CBCT in orthodontics: assessment of treatment outcomes and indications for its use. Dentomaxillofac Radiol. 2015;44(1):20140282. | Abstract |
|  | Karaaslan, A., et al., Brain Abscess Caused by Eikenella corrodens in an Immunocompetent Child: Case Report and Literature Review. Journal of Pediatric Infectious Diseases, 2022. 17(3): p. 170-174. | Title |
|  | Karabiber G, Yilmaz HN. Does unilateral surgically assisted rapid maxillary expansion (SARME) lead to perinasal asymmetry? Journal of Orofacial Orthopedics-Fortschritte Der Kieferorthopadie. 2021. | Title |
|  | Karacay S, Gokce S, Yildirim E. Evaluation of hyoid bone movements in subjects with open bite: a study with real-time balanced turbo field echo cine-magnetic resonance imaging. Korean Journal of Orthodontics. 2012;42(6):318-28. | Title |
|  | Karaikovic, E.E., et al., Possible complications of anterior perforation of the vertebral body using cervical pedicle screws. Journal of Spinal Disorders and Techniques, 2002. 15(1): p. 75-78. | Title |
|  | Karaman A, Kahveci H, Tos T, Arslan R, Öztürk C. Amniotic band sequence associated with fronto-ethmoidal meningoencephalocele: Case report. Clinical Genetics. 2010;78:17. | Title |
|  | Kareem, F.A., et al., Correlation of three dimensions of palate with maxillary arch form and perimeter as predictive measures for orthodontic and orthognathic surgery. Children, 2021. 8(6). | Title |
|  | Karia H, Shrivastav S, Karia AK. Three-dimensional evaluation of the airway spaces in patients with and without cleft lip and palate: A digital volume tomographic study. Am J Orthod Dentofacial Orthop. 2017;152(3):371-81. | Title |
|  | Karimi M, Koranyi J, Franco C, Peker Y, Eder DN, Angelhed JE, et al. Increased Neck Soft Tissue Mass and Worsening of Obstructive Sleep Apnea after Growth Hormone Treatment in Men with Abdominal Obesity. Journal of Clinical Sleep Medicine. 2010;6(3):256-63. | Title |
|  | Karslioglu, H., et al., The radiological evaluation of posterior superior alveolar artery by using cbct. Current Medical Imaging, 2021. 17(3): p. 384-389. | Title |
|  | Kasai N, Kondo O, Suzuki K, Aoki Y, Ishii N, Goto M. Quantitative evaluation of maxillary bone deformation by computed tomography in patients with leprosy. PLoS Neglected Tropical Diseases. 2018;12(3). | Title |
|  | Kasemsiri P, Solares CA, Carrau RL, Prosser JD, Prevedello DM, Otto BA, et al. Endoscopic endonasal transpterygoid approaches: anatomical landmarks for planning the surgical corridor. Laryngoscope. 2013;123(4):811-5. | Title |
|  | Kashiwagi M, Nakao K, Yamanaka S, Yamauchi I, Yamashita T, Fujii T, et al. Effect of C-type natriuretic peptide on craniofacial skeletogenesis in mice during the pubertal growth spurt. Archives of Oral Biology. 2022;139. | Title |
|  | Katanacho M, Lack JC, Fehlhaber F, Krüger J. Registration of pre- and postoperative surface scans for pediatric neurosurgery. Biomedizinische Technik. 2018;63:S301. | Title |
|  | Katina S, Kelly BD, Rojas MA, Sukno FM, McDermott A, Hennessy RJ, et al. Refining the resolution of craniofacial dysmorphology in bipolar disorder as an index of brain dysmorphogenesis. Psychiatry Research. 2020;291. | Title |
|  | Katsube M, Yamada S, Miyazaki R, Yamaguchi Y, Makishima H, Takakuwa T, et al. Quantitation of nasal development in the early prenatal period using geometric morphometrics and MRI: a new insight into the critical period of Binder phenotype. Prenatal Diagnosis. 2017;37(9):907-15. | Title |
|  | Katsube, M., et al., Critical Growth Processes for the Midfacial Morphogenesis in the Early Prenatal Period. Cleft Palate-Craniofacial Journal, 2019. 56(8): p. 1026-1037. | Title |
|  | Kau CH, Kamel SG, Wilson J, Wong ME. New method for analysis of facial growth in a pediatric reconstructed mandible. Am J Orthod Dentofacial Orthop. 2011;139(4):e285-90. | Title |
|  | Kau CH, Richmond S. Three-dimensional analysis of facial morphology surface changes in untreated children from 12 to 14 years of age. American Journal of Orthodontics and Dentofacial Orthopedics. 2008;134(6):751-60. | Title |
|  | Kau C.H. et al. Facial templates: A new perspective in three dimensions. Orthodontics and Craniofacial Research, 2006. 9(1): p. 10-17. | Title |
|  | KauckaM.et al.Signals from the brain and olfactory epithelium control shaping of the mammalian nasal capsule cartilage.eLife 2018. 7. | Title |
|  | Kaur H, Uludag H, Dederich D, El-Bialy T. Dose dependent effect of low intensity pulsed ultrasound on the condylar growth during functional appliance treatment. Journal of Bone and Mineral Research. 2013;28. | Title |
|  | Kaur N, Zappa D, Maraloiu VA, Comini E. Novel Christmas Branched Like NiO/NiWO4/WO3 (p-p-n) Nanowire Heterostructures for Chemical Sensing. Advanced Functional Materials. 2021;31(38). | Title |
|  | Kaur S, Rai S, Kaur M. Comparison of reliability of lateral cephalogram and computed tomography for assessment of airway space. Niger J Clin Pract. 2014;17(5):629-36. | Title |
|  | Kavand G, Lagravère M, Kula K, Stewart K, Ghoneima A. Retrospective CBCT analysis of airway volume changes after bone-borne vs tooth-borne rapid maxillary expansion. Angle Orthod. 2019;89(4):566-74. | Title |
|  | Kawaguchi J, Aihara S, Takano E, Matsuura N, Ichinohe T. Cuff Damage Caused by a Fixation Screw during Nasotracheal Intubation. Journal of Japanese Dental Society of Anesthesiology. 2018;46(1):25-7. | Title |
|  | Kawai T, Sato I, Asaumi R, Yosue T. Cone-beam computed tomography and anatomical observations of normal variants in the mandible: variant dentists should recognize. Oral Radiology. 2018;34(3):189-98. | Title |
|  | Kawakami S, Hiura K, Yokozeki M, Seike T, Nakanishi H, Moriyama K. Prognostic implications of nasal cavity and cleft morphology in secondary bone grafting. Cleft Palate-Craniofacial Journal. 2002;39(6):575-81. | Title |
|  | Kawamata A, Fujishita M, Ariji Y, Ariji E. Three-dimensional computed tomographic evaluation of morphologic airway changes after mandibular setback osteotomy for prognathism. Oral Surg Oral Med Oral Pathol Oral Radiol Endod. 2000;89(3):278-87. | Title |
|  | Kawasaki, K. and J.T. Richtsmeier, Association of the chondrocranium and dermatocranium in early skull formation, in Building Bones: Bone Formation and Development in Anthropology. 2017. p. 52-78. | Title |
|  | Kayalar E, Schauseil M, Hellak A, Emekli U, Firatli S, Korbmacher-Steiner H. Nasal soft- and hard-tissue changes following tooth-borne and hybrid surgically assisted rapid maxillary expansion: A randomized clinical cone-beam computed tomography study. Journal of Cranio-Maxillofacial Surgery. 2019;47(8):1190-7. | Title |
|  | Kecik D. Three-dimensional analyses of palatal morphology and its relation to upper airway area in obstructive sleep apnea. Angle Orthodontist. 2017;87(2):300-6. | Title |
|  | Kelly, B. and E. Carton, Extended Indications for Extracorporeal Membrane Oxygenation in the Operating Room. Journal of Intensive Care Medicine, 2020. 35(1): p. 24-33. | Title |
|  | Kenworthy J, Marshall H, Thomas S, Parra-Robles J, Xu X, Leung G, et al. Quantifying peripheral and central lung response to bronchodilator in asthma with hyperpolarised gas MRI. European Respiratory Journal. 2013;42. | Title |
|  | Kerbrat A, Vinuesa O, Lavergne F, Aversenq E, Graml A, Kerbrat JB, et al. Clinical impact of two types of mandibular retention devices - A CAD/CAM design and a traditional design - On upper airway volume in obstructive sleep apnea patients. Journal of stomatology, oral and maxillofacial surgery. 2021;122(4):361‐6. | Title |
|  | Keskin-Yalcin, B., et al., Evaluation of skeletal changes associated with surgically rapid palatal expansion without pterygomaxillary separation. Journal of Stomatology, Oral and Maxillofacial Surgery, 2020. 121(3): p. 254-258. | Title |
|  | Kesterke MJ, Raffensperger ZD, Heike CL, Cunningham ML, Hecht JT, Kau CH, et al. Using the 3D Facial Norms Database to investigate craniofacial sexual dimorphism in healthy children, adolescents, and adults. Biology of Sex Differences. 2016;7. | Title |
|  | Kethu A. Development of a smartphone application for Early Diagnosis of Mandibular Retrusion. Sleep Medicine. 2022;100:S59. | Title |
|  | Khaghaninejad, M.S., et al., Changes in the pharyngeal airway after different orthognathic procedures for correction of class III dysplasia. Maxillofacial Plastic and Reconstructive Surgery, 2022. 44(1). | Title |
|  | Khalatbari, S., et al., Craniofacial fractures pattern in motor vehicle accidents in Tehran, Iran: Epidemiology and predictive indices. Anil Aggrawal's Internet Journal of Forensic Medicine and Toxicology, 2020. 21(2): p. 1-9. | Title |
|  | Khanna A, Londhe ND, Gupta S. Automated localized approach for airway segmentation in 3D chest CT volume. Biomedical and Pharmacology Journal. 2020;13(4):1671-82. | Title |
|  | Khatib BN, Cuddy KK, Gelesko SL, Amundson M, Cheng A, Patel AA, et al. Application of virtual surgical planning to staged management of self-inflicted gunshot wounds to the maxillofacial skeleton: The oregon protocol. Journal of Oral and Maxillofacial Surgery. 2017;75(10):e367-e8. | Title |
|  | Khatib, B., et al., Functional Anatomic Computer Engineered Surgery Protocol for the Management of Self-Inflicted Gunshot Wounds to the Maxillofacial Skeleton. Journal of Oral and Maxillofacial Surgery, 2018. 76(3): p. 580-594. | Title |
|  | Kher, U., et al., A clinical and radiographic case series of implants placed with the simplified minimally invasive antral membrane elevation technique in the posterior maxilla. Journal of Cranio-Maxillofacial Surgery, 2014. 42(8): p. 1942-1947. | Title |
|  | Khosronejad A, Kang S, Sotiropoulos F. Experimental and computational investigation of local scour around bridge piers. Advances in Water Resources. 2012;37:73-85. | Title |
|  | Khwanda MA, Burhan AS, Hajeer MY, Ajaj MA, Parker S, Nawaya FR, et al. Three-Dimensional Assessment of the Temporomandibular Joint Changes Following Reversed Twin Block Therapy of Patients With Skeletal Class III Malocclusion in Conjunction With the Photobiomodulation Therapy: A Randomized Controlled Clinical Trial. Cureus Journal of Medical Science. 2022;14(6). | Title |
|  | Kiaee B, Nucci L, Sarkarat F, Talaeipour AR, Eslami S, Amiri F, et al. Three-dimensional assessment of airway volumes in patients with unilateral cleft lip and palate. Progress in Orthodontics. 2021;22(1). | Title |
|  | Kiesler K, Gugatschka M, Sorantin E, Friedrich G. Laryngo-tracheal profile: a new method for assessing laryngo-tracheal stenoses. European Archives of Oto-Rhino-Laryngology. 2007;264(3):251-6. | Title |
|  | **Kikuchi Y. Three-dimensional relationship between pharyngeal airway and maxillo-facial morphology. Bull Tokyo Dent Coll. 2008;49(2):65-75.** | **Included** |
|  | Kilic E, Doganay S, Ulu M, Çelebi N, Yikilmaz A, Alkan A. Determination of lingual vascular canals in the interforaminal region before implant surgery to prevent life-threatening bleeding complications. Clin Oral Implants Res. 2014;25(2):e90-3. | Title |
|  | Kim BG, Chung MJ, Jeong BH, Kim H. Diagnostic performance of digital tomosynthesis to evaluate silicone airway stents and related complications. Journal of Thoracic Disease. 2021;13(10):5627-37. | Title |
|  | Kim BR, Oh KM, Cevidanes LHS, Park JE, Sim HS, Seo SK, et al. Analysis of 3D Soft Tissue Changes After 1-and 2-Jaw Orthognathic Surgery in Mandibular Prognathism Patients. Journal of Oral and Maxillofacial Surgery. 2013;71(1):151-61. | Title |
|  | Kim C, Jackson N, Marcus CL, Pien GW, Staley B, Sun Y, et al. Relationship between upper airway collapsibility (PCRIT) and pharyngeal structures. American Journal of Respiratory and Critical Care Medicine. 2012;185. | Title |
|  | Kim DH, Lee IH, Yun WS, Shim JH, Choi D, Hwang SH, et al. Long-term efficacy and safety of 3D printed implant in patients with nasal septal deformities. European Archives of Oto-Rhino-Laryngology. 2022;279(4):1943-50. | Title |
|  | Kim DH, Yun WS, Shim JH, Park KH, Choi D, Park MI, et al. Clinical Application of 3-Dimensional Printing Technology for Patients with Nasal Septal Deformities: A Multicenter Study. JAMA Otolaryngology - Head and Neck Surgery. 2018;144(12):1145-52. | Title |
|  | Kim DY, Chung SM, Park JW. Automatic navigation path generation based on two-phase adaptive region-growing algorithm for virtual angioscopy. Medical Engineering & Physics. 2006;28(4):339-47. | Title |
|  | Kim ES, Kang JW, Kim CH, Hong JM. Cervical subcutaneous emphysema and pneumomediastinum after septorhinoplasty. J Craniofac Surg. 2014;25(2):533-4. | Title |
|  | Kim H, Lee KC. Sequential Changes in Pharyngeal Airway Dimensions After Mandibular Setback Surgery and Its Correlation With Postsurgical Stability in Patients With Mandibular Prognathism. Journal of Oral and Maxillofacial Surgery. 2021;79(12):2540-7. | Title |
|  | Kim HG, Lee JH, Song JM, Sandor GK, Kim YD. Outfracture of the inferior turbinates during superior repositioning Le Fort I osteotomy with cone-beam computed tomographic analysis of the volume of the nasal cavity. British Journal of Oral & Maxillofacial Surgery. 2016;54(3):290-4. | Title |
|  | Kim HS, Kim GT, Kim S, Lee JW, Kim EC, Kwon YD. Three-dimensional evaluation of the pharyngeal airway using cone-beam computed tomography following bimaxillary orthognathic surgery in skeletal class III patients. Clin Oral Investig. 2016;20(5):915-22. | Title |
|  | Kim HY, Varner VD, Nelson CM. Apical constriction initiates new bud formation during monopodial branching of the embryonic chicken lung. Development. 2013;140(15):3146-55. | Title |
|  | Kim IS, Lee MY, Lee KI, Kim HY, Chung YJ. Analysis of the Development of the Nasal Septum according to Age and Gender Using MRI. Clinical and Experimental Otorhinolaryngology. 2008;1(1):29-34. | Title |
|  | Kim JE, Hwang KJ, Kim SW, Liu SY, Kim SJ. Correlation between craniofacial changes and respiratory improvement after nasomaxillary skeletal expansion in pediatric obstructive sleep apnea patients. Sleep Breath. 2022;26(2):585-94. | Title |
|  | Kim JE, Man PP, Jang S, Yi HK. Nasal obstruction promotes alveolar bone destruction in the juvenile rat model. Journal of Dental Sciences. 2022;17(1):176-83. | Title |
|  | Kim JW, Kwon TG. Why most patients do not exhibit obstructive sleep apnea after mandibular setback surgery? Maxillofacial Plastic and Reconstructive Surgery. 2020;42(1). | Title |
|  | Kim M, Hwang CJ, Cha JY, Lee SH, Kim YJ, Yu HS. Correlation Analysis between Three-Dimensional Changes in Pharyngeal Airway Space and Skeletal Changes in Patients with Skeletal Class II Malocclusion following Orthognathic Surgery. BioMed Research International. 2022;2022. | Title |
|  | Kim MA, Kim BR, Choi JY, Youn JK, Kim YJ, Park YH. Three-dimensional changes of the hyoid bone and airway volumes related to its relationship with horizontal anatomic planes after bimaxillary surgery in skeletal Class III patients. Angle Orthod. 2013;83(4):623-9. | Title |
|  | Kim MA, Kim BR, Youn JK, Kim YJ, Park YH. Head posture and pharyngeal airway volume changes after bimaxillary surgery for mandibular prognathism. J Craniomaxillofac Surg. 2014;42(5):531-5. | Title |
|  | Kim MA, Park YH. Does upper premolar extraction affect the changes of pharyngeal airway volume after bimaxillary surgery in skeletal class iii patients? Journal of Oral and Maxillofacial Surgery. 2014;72(1):165.e1-.e10. | Title |
|  | Kim MK, Kang SH, Kim YH, Won YJ, Roh HW, Jang HS. Computational fluid dynamic analysis of the upper airway flow after orthognathic surgery. International Journal of Computer Assisted Radiology and Surgery. 2017;12(1):S282-S3. | Title |
|  | Kim NR, Kim YI, Park SB, Hwang DS. Three dimensional cone-beam CT study of upper airway change after mandibular setback surgery for skeletal Class III malocclusion patients. Korean Journal of Orthodontics. 2010;40(3):145-55. | Title |
|  | Kim SR, Lee KM, Cho JH, Hwang HS. Three-dimensional prediction of the human eyeball and canthi for craniofacial reconstruction using cone-beam computed tomography. Forensic Science International. 2016;261:164.e1-.e8. | Title |
|  | Kim T, Baek SH, Choi JY. Effect of posterior impaction and setback of the maxilla on retropalatal airway and velopharyngeal dimensions after two-jaw surgery in skeletal Class III patients. Angle Orthodontist. 2015;85(4):625-30. | Title |
|  | Kim YI, Kim SS, Son WS, Park SB. Pharyngeal airway analysis of different craniofacial morphology using cone-beam computed tomography (CBCT). Korean Journal of Orthodontics. 2009;39(3):136-45. | Full text  No volumes |
|  | **Kim YJ, Hong JS, Hwang YI, Park YH. Three-dimensional analysis of pharyngeal airway in preadolescent children with different anteroposterior skeletal patterns. Am J Orthod Dentofacial Orthop. 2010;137(3):306.e1-11; discussion -7.** | **Included** |
|  | Kim YM, Rha KS, Weissman JD, Hwang PH, Most SP. Correlation of Asymmetric Facial Growth with Deviated Nasal Septum. Laryngoscope. 2011;121(6):1144-8. | Title |
|  | Kim, J.H., et al., Histologic changes in transplanted expanded polytetrafluoroethylene in an animal model. Laryngoscope, 2012. 122(1): p. 17-22. | Title |
|  | Kim, K.A., S.J. Kim, and Y.G. Park, TAD-assisted naso-maxillo-pharyngeal expansion, in Temporary Anchorage Devices in Clinical Orthodontics. 2020. p. 243-257. | Title |
|  | Kim, K.B., et al., Computational airflow analysis before and after maxillomandibular advancement surgery. Journal of the World Federation of Orthodontists, 2016. 5(1): p. 2-8. | Title |
|  | Kim, M., et al., Three-dimensional evaluation of soft tissue changes after mandibular setback surgery in class III malocclusion patients according to extent of mandibular setback, vertical skeletal pattern, and genioplasty. Oral Surgery, Oral Medicine, Oral Pathology, Oral Radiology and Endodontology, 2010. 109(5): p. e20-e32. | Title |
|  | Kim, M.S., et al., Low-dose recombinant human bone morphogenetic protein-2 to enhance the osteogenic potential of the schneiderian membrane in the early healing phase: In vitro and in vivo studies. Journal of Oral and Maxillofacial Surgery, 2014. 72(8): p. 1480-1494. | Title |
|  | Kim O.K.et al.A mixture of humulus japonicus increases longitudinal bone growth rate in sprague dawley rats. Nutrients 2020.12:1-10. | Title |
|  | Kim O.K. et al. Effects of a Mixture of Humulus japonicus on Longitudinal Bone Growth in Hypophysectomized Rats. Journal of Medicinal Food, 2021. 24(5): p. 497-504. | Title |
|  | Kim, S.H. and S.K. Choi, Changes in the hyoid bone, tongue, and oropharyngeal airway space after mandibular setback surgery evaluated by cone-beam computed tomography. Maxillofacial Plastic and Reconstructive Surgery, 2020. 42(1). | Title |
|  | Kim, S.M., T.M. McCulloch, and K. Rim, Pharyngeal pressure analysis by the finite element method during liquid bolus swallow. Annals of Otology, Rhinology and Laryngology, 2000. 109(6): p. 585-589. | Title |
|  | Kim, Y.J.R., et al., 3D Assessment of Orthognathic Surgical Outcomes, in Cone Beam Computed Tomography in Orthodontics: Indications, Insights, and Innovations. 2014. p. 463-483. | Title |
|  | Kimbell JS, Ferkol TW, Ranganathan SC, Davis SD. Ventilation distribution in early cystic fibrosis. American Journal of Respiratory and Critical Care Medicine. 2015;191. | Title |
|  | Kimes KR, Mooney MP, Siegel MI, Todhunter JS, Langdon HL. Growth rate of the vomer in normal and cleft lip and palate human fetal specimens. Cleft Palate-Craniofacial Journal. 1992;29(1):38-43. | Title |
|  | Kimes KR, Mooney MP, Siegel MI, Todhunter JS. Size and growth rate of the tongue in normal and cleft lip and palate human fetal specimens. Cleft Palate-Craniofacial Journal. 1991;28(2):212-6. | Title |
|  | Kimura E, Obata T, Kitai S, Ishii T, Sakamoto T, Watanabe M, et al. Three-dimensional measurements of pharyngeal airway in patients with unilateral cleft lip and palate. Bulletin of Tokyo Dental College. 2020;61(4):213‐9. | Title |
|  | Kinoshita, Y., et al., Gene Therapy Using Adeno-Associated Virus Serotype 8 Encoding TNAP-D10 Improves the Skeletal and Dentoalveolar Phenotypes in Alpl−/− Mice. Journal of Bone and Mineral Research, 2021. 36(9): p. 1835-1849. | Title |
|  | Kinzinger G, Czapka K, Ludwig B, Glasl B, Gross U, Lisson J. Effects of fixed appliances in correcting Angle Class II on the depth of the posterior airway space. Journal of Orofacial Orthopedics-Fortschritte Der Kieferorthopadie. 2011;72(4):301-20. | Title |
|  | Kiraly AP, Higgins WE, McLennan G, Hoffman EA, Reinhardt JM. Three-dimensional human airway segmentation methods for clinical virtual bronchoscopy. Acad Radiol. 2002;9(10):1153-68. | Title |
|  | Kirk, E.C., et al., Cranial anatomy of the duchesnean primate Rooneyia viejaensis: New insights from high resolution computed tomography. Journal of Human Evolution, 2014. 74: p. 82-95. | Title |
|  | Kishimoto, K. and M. Morimoto, Mammalian tracheal development and reconstruction: Insights from in vivo and in vitro studies. Development (Cambridge), 2021. 148(13). | Title |
|  | Kita S, Oshima M, Shimazaki K, Iwai T, Omura S, Ono T. Computational fluid dynamic study of nasal respiratory function before and after bimaxillary orthognathic surgery with bone trimming at the inferior edge of the pyriform aperture. Journal of oral and maxillofacial surgery. 2016;74(11):2241‐51. | Title |
|  | Kitai N, Iguchi Y, Takashima M, Murakami S, Kreiborg S, Kamiji T, et al. Craniofacial Morphology in an Unusual Case with Nasal Aplasia Studied by Roentgencephalometry and Three-Dimensional CT Scanning. Cleft Palate-Craniofacial Journal. 2004;41(2):208-13. | Title |
|  | Kjaerulff, A.M.G., M. Rusan, and T.E. Klug, Clinical evaluation of intravenous ampicillin as empirical antimicrobial treatment of acute epiglottitis. Acta Oto-Laryngologica, 2018. 138(1): p. 60-65. | Title |
|  | Klazen YP, Caron C, Schaal SC, Borghi A, Van der Schroeff MP, Dunaway DJ, et al. What Are the Characteristics of the Upper Airway in Patients With Craniofacial Microsomia? J Oral Maxillofac Surg. 2019;77(9):1869-81. | Title |
|  | Klepacek I, Mala PZ. "Bochdalek's" skull: morphology report and reconstruction of face. Forensic Science Medicine and Pathology. 2012;8(4):451-9. | Title |
|  | Kling DE, Tsvang I, Murphy MP, Newburg DS. Group B Streptococcus induces a caspase-dependent apoptosis in fetal rat lung interstitium. Microbial Pathogenesis. 2013;61-62:1-10. | Title |
|  | Klotch DW. Frontal sinus fractures: anterior skull base. Facial Plast Surg. 2000;16(2):127-34. | Title |
|  | Knabe, C., et al., Effect of sex-hormone levels, sex, body mass index and other host factors on human craniofacial bone regeneration with bioactive tricalcium phosphate grafts. Biomaterials, 2017. 123: p. 48-62. | Title |
|  | Knauthe P, Beutel RG, Hornschemeyer T, Pohl H. Serial block-face scanning electron microscopy sheds new light on the head anatomy of an extremely miniaturized insect larva (Strepsiptera). Arthropod Systematics & Phylogeny. 2016;74(2):107-26. | Title |
|  | Knight, Z.L., et al., The Changing Nasolabial Dimensions following Repair of Unilateral Cleft Lip: An Anthropometric Study in Late Childhood. Plastic and Reconstructive Surgery, 2016. 138(5): p. 879e-886e. | Title |
|  | Knoops PGM, Borghi A, Ruggiero F, Badiali G, Bianchi A, Marchetti C, et al. A novel soft tissue prediction methodology for orthognathic surgery based on probabilistic finite element modelling. PLoS ONE. 2018;13(5). | Title |
|  | Knudsen TB, Laulund AS, Ingerslev J, Homoe P, Pinholt EM. Improved Apnea-Hypopnea Index and Lowest Oxygen Saturation After Maxillomandibular Advancement With or Without Counterclockwise Rotation in Patients With Obstructive Sleep Apnea: A Meta-Analysis. Journal of Oral and Maxillofacial Surgery. 2015;73(4):719-26. | Title |
|  | Ko, H.S., et al., Nasolabial dimensions of the facial profile at 20 to 37 weeks' gestation on 2- and 3-dimensional sonography in normal Korean fetuses. Journal of Ultrasound in Medicine, 2013. 32(4): p. 617-624. | Title |
|  | Kocabalkanli C, Aalamifar F, Fenton R, Gaetani S, Linguraru M, Seifabadi R. Measuring deformational plagiocephaly and brachycephaly using a smartphone in a prospective study. Cleft Palate-Craniofacial Journal. 2022;59(4 SUPPL):51. | Title |
|  | Kocakara G, Buyukcavus MH, Orhan H. Evaluation of pharyngeal airway dimensions and hyoid bone position according to craniofacial growth pattern. Cranio-the Journal of Craniomandibular & Sleep Practice. 2022;40(4):313-23. | Full text  2D lat. cephalometry |
|  | Kocandrlova K, Dupej J, Hoffmannova E, Veleminska J. Three-dimensional mixed longitudinal study of facial growth changes and variability of facial form in preschool children using stereophotogrammetry. Orthodontics & Craniofacial Research. 2021;24(4):511-9. | Title |
|  | Kochar GD, Chakranarayan A, Kohli S, Kohli VS, Khanna V, Jayan B, et al. Effect of surgical mandibular advancement on pharyngeal airway dimensions: a three-dimensional computed tomography study. International Journal of Oral and Maxillofacial Surgery. 2016;45(5):553-9. | Title |
|  | Kochar GD, Sharma M, Chowdhury SKR, Londhe SM, Kumar P, Jain A, et al. Pharyngeal airway evaluation following isolated surgical mandibular advancement: A 1-year follow-up. American Journal of Orthodontics and Dentofacial Orthopedics. 2019;155(2):207-15. | Title |
|  | Kochel J, Meyer-Marcotty P, Sickel F, Lindorf H, Stellzig-Eisenhauer A. Short-term pharyngeal airway changes after mandibular advancement surgery in adult Class II-Patients-a three-dimensional retrospective study. Journal of Orofacial Orthopedics-Fortschritte Der Kieferorthopadie. 2013;74(2):137-52. | Title |
|  | **Kochhar AS, Sidhu MS, Bhasin R, Kochhar GK, Dadlani H, Sandhu J, et al. Cone beam computed tomographic evaluation of pharyngeal airway in North Indian children with different skeletal patterns. World Journal of Radiology. 2021;13(2):40-52.** | **Included** |
|  | Köhler, C., et al., Sonographic examination of the soft palate in dogs. Ultraschall in der Medizin, 2011. 32(SUPPL. 2): p. E191-E201. | Title |
|  | Kohn J.C. et al. Infratemporal fossa fat enlargement in chronic maxillary atelectasis. British J Ophthalmology 2013. 97(8): 1005-1009. | Title |
|  | Kolewe EL, Stillman Z, Woodward IR, Fromen CA. Check the gap: Facemask performance and exhaled aerosol distributions around the wearer. PLoS ONE. 2020;15(12 December). | Title |
|  | Kollara L, Perry JL, Hudson S. Racial Variations in Velopharyngeal and Craniometric Morphology in Children: An Imaging Study. Journal of Speech Language and Hearing Research. 2016;59(1):27-38. | Abstract |
|  | Kolodziej J, Al Basri S, Azizi M, Rombach A, Kirchhoff F, Wright P. Causes, diagnosis and treatment of neurogenic dysphagia. Nervenheilkunde. 2021;40(11):912-8. | Title |
|  | Kong, M., et al., A new application of modified Nishida muscle transposition procedure for medial rectus muscle transection following endoscopic sinus surgery without tenotomy or splitting muscles. Journal of AAPOS, 2019. 23(5): p. 287-289. | Title |
|  | Kongsong W, Waite PD, Sittitavornwong S, Schibler M, Alshahrani F. The correlation of maxillomandibular advancement and airway volume change in obstructive sleep apnea using cone beam computed tomography. International Journal of Oral and Maxillofacial Surgery. 2021;50(7):940-7. | Title |
|  | Kooiman TD, Calabrese CE, Didier R, Estroff JA, Padwa BL, Koudstaal MJ, et al. Micrognathia and Oropharyngeal Space in Patients With Robin Sequence: Prenatal MRI Measurements. Journal of Oral and Maxillofacial Surgery. 2018;76(2):408-15. | Title |
|  | Korayem MM, Witmans M, MacLean J, Heo G, El-Hakim H, Flores-Mir C, et al. Craniofacial morphology in pediatric patients with persistent obstructive sleep apnea with or without positive airway pressure therapy: a cross-sectional cephalometric comparison with controls. Am J Orthod Dentofacial Orthop. 2013;144(1):78-85. | Title |
|  | Kortes J, Dehnad H, Kotte ANT, Fennis WMM, Rosenberg A. A novel digital workflow to manufacture personalized three-dimensional-printed hollow surgical obturators after maxillectomy. Int J Oral Maxillofac Surg. 2018;47(9):1214-8. | Title |
|  | Kosztyła-Hojna B,Popko M.[Intranasal meningocele presenting as a nasal polyp--case report].Pol Merkur Lekarski. 2008;24(139):27-9. | Title |
|  | Kotlarek K, Jaskolka M, Fang X, Ellis C, Sutton B, Blemker S, et al. A preliminary study of anatomical changes following the use of a pedicled buccal fat pad graft during primary palatoplasty. Cleft Palate-Craniofacial Journal. 2020;57(4):46. | Title |
|  | Kotlarek K, Jaskolka M, Sutton B, Blemker S, Horswell B, Kloostra P, et al. 5-year maintenance of the pedicled buccal fat pad graft during primary palatoplasty: An MRI case study. Cleft Palate-Craniofacial Journal. 2020;57(4):59. | Title |
|  | Kotlarek K, Medeiros-Santana MN, Yamashita R, Perry J. Can visual inspection be utilized to reliably classify velar length? Cleft Palate-Craniofacial Journal. 2019;56(1):56-7. | Title |
|  | Kotlarek K, Sitzman T, Williams J, Perry J. Successful Completion of Non-sedated MRI for Visualization of the Velopharynx in Children. Cleft Palate-Craniofacial Journal. 2022;59(4 SUPPL):19. | Title |
|  | Kotrikova B, Hassfeld S, Steiner HH, Hahnel S, Krempien R, Muhling J. Operative correction and follow-up of craniofacial duplication. Plastic and Reconstructive Surgery. 2007;119(3):985-91. | Title |
|  | Kouamo J, Saague AMN, Zoli AP. Determination of age and weight of bovine fetus (Bos indicus) by biometry. Journal of Livestock Science. 2018;9:9-15. | Title |
|  | Koudelova J, Bruzek J, Caganova V, Krajicek V, Veleminska J. Development of facial sexual dimorphism in children aged between 12 and 15years: a three-dimensional longitudinal study. Orthodontics & Craniofacial Research. 2015;18(3):175-84. | Abstract |
|  | Koudelová J, Dupej J, Brůžek J, Sedlak P, Velemínská J. Modelling of facial growth in Czech children based on longitudinal data: Age progression from 12 to 15 years using 3D surface models. Forensic Science International. 2015;248:33-40. | Abstract |
|  | Kovacevic, M. and J. Wurm, New aspects in surgery of the nasal tip. HNO, 2015. 63(1): p. 34-45. | Title |
|  | Kowalczyk P, Kleiber M. MODELING AND NUMERICAL-ANALYSIS OF STRESSES AND STRAINS IN THE HUMAN LUNG INCLUDING TISSUE-GAS INTERACTION. European Journal of Mechanics a-Solids. 1994;13(3):367-93. | Title |
|  | Krall, M., et al., Biallelic sequence variants in INTS1 in patients with developmental delays, cataracts, and craniofacial anomalies. European Journal of Human Genetics, 2019. 27(4): p. 582-593. | Title |
|  | Kramer, B., K. Molema, and E.F. Hutchinson, An osteological assessment of cyclopia by micro-CT scanning. Surgical and Radiologic Anatomy, 2019. 41(9): p. 1053-1063. | Title |
|  | Krarup JF, Nielsen HL, Danstrup CS. Severe deep neck space infection caused by Eggerthia catenaformis. BMJ Case Rep. 2021;14(4). | Title |
|  | Krasny M, Wysocki J, Prus M, Niemczyk K. Location of the narrowest area of the pharynx regarding body mass index and obstructive sleep apnoea severity. Folia Morphol (Warsz). 2017;76(3):491-500. | Title |
|  | Krastinova-Lolov, D., Mask lift and facial aesthetic sculpturing. Plastic and Reconstructive Surgery, 1995. 95(1): p. 21-36. | Title |
|  | Kravanja SL, Hocevar-Boltezar I, Music MM, Jarc A, Verdenik I, Ovsenik M. Three-dimensional ultrasound evaluation of tongue posture and its impact on articulation disorders in preschool children with anterior open bite. Radiology and Oncology. 2018;52(3):250-6. | Title |
|  | Kreiborg S, Dahl E. Cranial base and face in mandibulofacial dysostosis. Am J Med Genet. 1993;47(5):753-60. | Title |
|  | Kreissl, M.E., et al., Zygoma implant-supported prosthetic rehabilitation after partial maxillectomy using surgical navigation: A clinical report. Journal of Prosthetic Dentistry, 2007. 97(3): p. 121-128. | Title |
|  | Krespi YP, Husain S, Levine TM, Reede DL. Sublabial transseptal repair of choanal atresia or stenosis. Laryngoscope. 1987;97(12):1402-6. | Title |
|  | Krimmel M, Breidt M, Bacher M, Muller-Hagedorn S, Dietz K, Bulthoff H, et al. Three-Dimensional Normal Facial Growth from Birth to the Age of 7 Years. Plastic and Reconstructive Surgery. 2015;136(4):490E-501E. | Title |
|  | Krimmel M, Kluba S, Bacher M, Dietz K, Reinert S. Digital surface photogrammetry for anthropometric analysis of the cleft infant face. Cleft Palate-Craniofacial Journal. 2006;43(3):350-5. | Title |
|  | Krimmel M, Kluba S, Breidt M, Bacher M, Dietz K, Buelthoff H, et al. Three-Dimensional Assessment of Facial Development in Children With Pierre Robin Sequence. Journal of Craniofacial Surgery. 2009;20(6):2055-60. | Title |
|  | Krimmel M, Schuck N, Bacher M, Reinert S. Facial surface changes after cleft alveolar bone grafting. Journal of Oral and Maxillofacial Surgery. 2011;69(1):80-3. | Title |
|  | Krimmel, M., et al., Three-dimensional assessment of facial development in children with unilateral cleft lip with and without alveolar cleft. Journal of Craniofacial Surgery, 2013. 24(1): p. 313-316. | Title |
|  | Krmpotić-Nemanić, J., et al., Variations of the ethmoid labyrinth and sphenoid sinus and CT imaging. Oto-Rhino-Laryngology, 1993. 250(4): p. 209-212. | Title |
|  | Krneta, B., et al., Diagnosis of Class III malocclusion in 7- to 8-year-old children - A 3D evaluation. European Journal of Orthodontics, 2015. 37(4): p. 379-385. | Title |
|  | Krolewski RC, Schwob JE. Expansion, engraftment and multi-lineage potency of mouse neonatal olfactory neurospheres. Chemical Senses. 2009;34(7):A86. | Title |
|  | Krüsi, M., T. Eliades, and S.N. Papageorgiou, Are there benefits from using bone-borne maxillary expansion instead of tooth-borne maxillary expansion? A systematic review with meta-analysis. Progress in Orthodontics, 2019. 20(1). | Title |
|  | Kuan CH, Lin CY, Hsaio JK, Chen JS, Han YY. Prognostic Factors of Survival From Intractable Oronasal Bleeding After Successful Transarterial Embolization. J Oral Maxillofac Surg. 2015;73(9):1790-4. | Title |
|  | Kuang W, Zheng J, Li S, Yuan S, He H, Yuan W. Three-Dimensional Analysis of the Pharyngeal Airway Volume and Craniofacial Morphology in Patients With Bilateral Cleft Lip and Palate. Cleft Palate-Craniofacial Journal. 2021;58(3):332-9. | Title |
|  | Kuang, W., et al., Three-Dimensional Analysis of the Pharyngeal Airway Morphology of Children with Complete Left CLP. Medical Journal of Wuhan University, 2017. 38(4): p. 618-621 and 650. | Title |
|  | Kubiak M, Beckmann F, Friedrich F. The adult head of the annulipalpian caddisfly Philopotamus ludificatus McLachlan, 1878 (Philopotamidae), mouthpart homologies, and implications on the ground plan of Trichoptera. Arthropod Systematics & Phylogeny. 2015;73(3):351-84. | Title |
|  | Kuijpers MAR, Chiu YT, Nada RM, Carels CEL, Fudalej PS. Three-dimensional Imaging Methods for Quantitative Analysis of Facial Soft Tissues and Skeletal Morphology in Patients with Orofacial Clefts: A Systematic Review. Plos One. 2014;9(4). | Title |
|  | Kulubya ES, Tejas K, Thaci B, Duong H. Endoscopic transnasal transodontoid treatment of a ruptured anterior spinal artery aneurysm. Journal of Neurological Surgery Part B: Skull Base. 2021;82(SUPPL 2). | Title |
|  | Kumamaru KK, Sisk GC, Mitsouras D, Schultz K, Steigner ML, George E, et al. Vascular communications between donor and recipient tissues after successful full face transplantation. American Journal of Transplantation. 2014;14(3):711-9. | Title |
|  | Kumar, A., T.G. Davies, and N. Itasaki, Developmental abnormalities of the otic capsule and inner ear following application of prolyl-hydroxylase inhibitors in chick embryos. Birth Defects Research, 2018. 110(15): p. 1194-1204. | Title |
|  | Kumjan N, Manosudprasit A, Pisek A, Winaikosol K, Manosudprasit M, Pisek P, et al. A Three-Dimensional Comparison of Nasolabial Soft Tissue between Children with Repaired Unilateral Complete Cleft Lip and Palate and Unaffected Children in Khon Kaen, Thailand. Journal of Clinical Pediatric Dentistry. 2020;44(6):442-50. | Title |
|  | Kunjur J, Messiah A, Manisali M. Obstructive sleep apneoa after orthognathic surgery. International Journal of Oral and Maxillofacial Surgery. 2015;44:e250-e1. | Title |
|  | Kunkel M, Hochban W. ACOUSTIC RHINOMETRY - RATIONALE AND PERSPECTIVES. Journal of Cranio-Maxillofacial Surgery. 1994;22(4):244-9. | Title |
|  | Kunkel M, Wahlmann U, Wagner W. Acoustic airway profiles in unilateral cleft palate patients. Cleft Palate-Craniofacial Journal. 1999;36(5):434-40. | Title |
|  | Kuramoto E, Nishiuma T, Kobayashi K, Yamamoto M, Kono Y, Funada Y, et al. Inhalation of urokinase-type plasminogen activator reduces airway remodeling in a murine asthma model. American Journal of Physiology - Lung Cellular and Molecular Physiology. 2009;296(3):L337-L46. | Title |
|  | Kurnik NM, Calis M, Sobol DL, Kapadia H, Mercan E, Tse RW. A Comparative Assessment of Nasal Appearance following Nasoalveolar Molding and Primary Surgical Repair for Treatment of Unilateral Cleft Lip and Palate. Plastic and Reconstructive Surgery. 2021;148(5):1075-84. | Title |
|  | Kustár A, Forró L, Kalina I, Fazekas F, Honti S, Makra S, et al. FACE-R--a 3D database of 400 living individuals' full head CT- and face scans and preliminary GMM analysis for craniofacial reconstruction. J Forensic Sci. 2013;58(6):1420-8. | Title |
|  | Kuttenberger JJ, Hardt N, Schlegel C. Diagnosis and initial management of laryngotracheal injuries associated with facial fractures. J Craniomaxillofac Surg. 2004;32(2):80-4. | Title |
|  | Kwon, T.G., S.M. Kang, and H.D. Hwang, Three-dimensional soft tissue change after paranasal augmentation with porous polyethylene. International Journal of Oral and Maxillofacial Surgery, 2014. 43(7): p. 816-823. | Title |
|  | Kyllar M, Stembirek J, Putnova I, Stehlik L, Odehnalova S, Buchtova M. Radiography, Computed Tomography and Magnetic Resonance Imaging of Craniofacial Structures in Pig. Anatomia Histologia Embryologia. 2014;43(6):435-52. | Title |
|  | Lacquaniti F, Maioli C. COORDINATE TRANSFORMATIONS IN THE CONTROL OF CAT POSTURE. Journal of Neurophysiology. 1994;72(4):1496-515. | Title |
|  | Lacquaniti, F. and C. Maioli, Coordinate transformations in the control of cat posture. Journal of Neurophysiology, 1994. 72(4): p. 1496-1515. | Title |
|  | Lagarde M, Knuijt S, Groothuis J, De Groot I, Van Den Engel-Hoek L. Longitudinal changes in oral and masticatory muscles in duchenne muscular dystrophy: A disturbed balance. Dysphagia. 2017;32(1):142. | Title |
|  | Lagvilava, G., et al., INTRA-ARTERIAL INFUSIONS AND DOPLEROGRAPIC CONTROL FOR COMPLEX TREATMENT OF UPPER AND MIDDLE FACIAL ZONES, CONCOMITANT WITH TRAUMATIC CRANIOCEREBRAL INJURIES. Georgian medical news, 2016(251): p. 16-21. | Title |
|  | Lahey Iii ET, Lee SH, Kaban LB. Skeletal stability of patients undergoing maxillomandibular advancement for treatment of obstructive sleep apnea. Journal of Oral and Maxillofacial Surgery. 2014;72(9):e25-e6. | Title |
|  | Lam B, Ooi CGC, Peh WCG, Lauder I, Tsang KWT, Lam WK, et al. Computed tomographic evaluation of the role of craniofacial and upper airway morphology in obstructive sleep apnea in Chinese. Respiratory Medicine. 2004;98(4):301-7. | Title |
|  | Lam SY, Bux SI, Kumar G, Ng KH, Hussain AF. A comparison between low-dose and standard-dose noncontrasted multidetector CT scanning of the paranasal sinuses. Biomedical imaging and intervention journal. 2009;5(3). | Title |
|  | Lamb CR, Ciasca TC, Mantis P, Forcada Y, Potter M, Church DB, et al. Computed tomographic signs of acromegaly in 68 diabetic cats with hypersomatotropism. Journal of Feline Medicine and Surgery. 2014;16(2):99-108. | Title |
|  | Lambodaran, G., et al., A digital cephalometric study to evaluate the effect of age in relating the level of ala tragal line to the occlusal plane. International Journal of Current Research and Review, 2020. 12(19): p. 122-127. | Title |
|  | Lamichhane, A.P., Osteoporosis - An update. Journal of the Nepal Medical Association, 2005. 44(158): p. 60-66. | Title |
|  | Lana, M.V.D.C., et al., Wry nose in an adult equine. Acta Scientiae Veterinariae, 2012. 40(3). | Title |
|  | Landi F, Profico A, O'Higgins P. Maxillary sinus growth and development in Neanderthals and H. Sapiens. American Journal of Physical Anthropology. 2019;168:135-6. | Title |
|  | Landi, F., et al., The role of the nasal region in craniofacial growth: An investigation using path analysis. Anatomical Record, 2022. 305(8): p. 1892-1909. | Title |
|  | Lane JA, Maisey JG. THE VISCERAL SKELETON AND JAW SUSPENSION IN THE DUROPHAGOUS HYBODONTID SHARK TRIBODUS LIMAE FROM THE LOWER CRETACEOUS OF BRAZIL. Journal of Paleontology. 2012;86(5):886-905. | Title |
|  | Lanteri V, Farronato M, Ugolini A, Cossellu G, Gaffuri F, Parisi FMR, et al. Volumetric Changes in the Upper Airways after Rapid and Slow Maxillary Expansion in Growing Patients: A Case-Control Study. Materials. 2020;13(10). | Title |
|  | Larici AR, Glaudemans AW, Del Ciello A, Slart RH, Calandriello L, Gheysens O. Radiological and nuclear medicine imaging of sarcoidosis. Q J Nucl Med Mol Imaging. 2018;62(1):14-33. | Title |
|  | Lashkarinia, S.S., et al., Spatiotemporal remodeling of embryonic aortic arch: stress distribution, microstructure, and vascular growth in silico. Biomechanics and Modeling in Mechanobiology, 2020. 19(5): p. 1897-1915. | Title |
|  | Lata S, Mohanty SK, Vinay S, Das AC, Das S, Choudhury P. "Is Cone Beam Computed Tomography (CBCT) a Potential Imaging Tool in ENT Practice?: A Cross-Sectional Survey Among ENT Surgeons in the State of Odisha, India. Indian Journal of Otolaryngology and Head & Neck Surgery. 2018;70(1):130-6. | Title |
|  | Lautner, N., et al., Three-dimensional evaluation of the effect of nasoalveolar molding on the volume of the alveolar gap in unilateral clefts. Journal of Cranio-Maxillofacial Surgery, 2020. 48(2): p. 141-147. | Title |
|  | Law CJ, Dorgan KM, Rouse GW. Relating Divergence in Polychaete Musculature to Different Burrowing Behaviors: A Study Using Opheliidae (Annelida). Journal of Morphology. 2014;275(5):548-71. | Title |
|  | Laxmi, N.V., et al., Importance of cephalographs in diagnosis of patients with sleep apnea. Contemporary Clinical Dentistry, 2015. 6: p. S221-S226. | Title |
|  | Lazarus, A., et al., A perfusion-independent role of blood vessels in determining branching stereotypy of lung airways. Development, 2011. 138(11): p. 2359-2368. | Title |
|  | Learreta JA. Síndrome de Binder. Ortodoncia. 2015;79(158):48-50. | Title |
|  | Leary RP, Manuel CT, Shamouelian D, Protsenko DE, Wong BJF. Finite Element Model Analysis of Cephalic Trim on Nasal Tip Stability. Jama Facial Plastic Surgery. 2015;17(6):413-20. | Title |
|  | Lee DH, Jin KS. Effect of Nasal Septal Deviation on Pneumatization of the Mastoid Air Cell System: 3D Morphometric Analysis of Computed Tomographic Images in a Pediatric Population. Journal of International Advanced Otology. 2014;10(3):251-5. | Title |
|  | Lee J, Ku B, Combs PD, Da Silveira AC, Markey MK. Quantitative Anthropometric Measures of Facial Appearance of Healthy Hispanic/Latino White Children: Establishing Reference Data for Care of Cleft Lip With or Without Cleft Palate. 3d Research. 2017;8(2). | Title |
|  | **Lee JH, Park SB, Jeon EY, Park JT. Three-dimensional measurements of pharynx structures in malocclusion. Medico-Legal Update. 2019;19(1):429-33.** | **Included** |
|  | Lee JS, Kim JB, Lee JW, Yang JD, Chung HY, Cho BC, et al. Factors prognostic for phonetic development after cleft palate repair. Journal of Cranio-Maxillofacial Surgery. 2015;43(8):1602-7. | Title |
|  | Lee JY, Kim YI, Hwang DS, Park SB. Effect of maxillary setback movement on upper airway in patients with class III skeletal deformities: cone beam computed tomographic evaluation. J Craniofac Surg. 2013;24(2):387-91. | Title |
|  | Lee K, Hwang SJ. Change of the upper airway after mandibular setback surgery in patients with mandibular prognathism and anterior open bite. Maxillofacial Plastic and Reconstructive Surgery. 2019;41(1). | Title |
|  | Lee K, Yamada K, Tsuneda R, Kishimoto M, Shimizu J, Kobayashi Y, et al. Clinical experience of using multicletector-row CT for the diagnosis of disorders in cattle. Veterinary Record. 2009;165(19):559-62. | Title |
|  | Lee RWW, Sutherland K, Chan ASL, Zeng BA, Grunstein RR, Darendeliler MA, et al. Relationship Between Surface Facial Dimensions and Upper Airway Structures in Obstructive Sleep Apnea. Sleep. 2010;33(9):1249-54. | Title |
|  | Lee RWW. Three-dimensional facial phenotyping in obstructive sleep apnoea. Respirology. 2018;23(6):560-1. | Title |
|  | Lee SJ, Tse KM, Lee HP. Similar Fracture Patterns in Human Nose and Gothic Cathedral. Facial Plast Surg. 2015;31(5):553-60. | Title |
|  | Lee SJ, Yoo SK, Moon DJ, Kim JA, Yoo JY, Choi MH, et al. Modified Point- and Surface-based Registration and their Accuracy Evaluation Methods for Computer Assisted Maxillofacial Surgical System. International Journal of Computer Assisted Radiology and Surgery. 2019;14:S188-S9. | Title |
|  | Lee SR, Lee JW, Chung DH, Lee SM. Short-term impact of microimplant-assisted rapid palatal expansion on the nasal soft tissues in adults: A three-dimensional stereophotogrammetry study. Korean Journal of Orthodontics. 2020;50(2):75-85. | Title |
|  | Lee ST, Park JH, Kwon TG. Influence of mandibular setback surgery on three-dimensional pharyngeal airway changes. International Journal of Oral and Maxillofacial Surgery. 2019;48(8):1057-65. | Title |
|  | Lee UL, Oh H, Min SK, Shin JH, Kang YS, Lee WW, et al. The structural changes of upper airway and newly developed sleep breathing disorders after surgical treatment in class III malocclusion subjects. Medicine (United States). 2017;96(22). | Title |
|  | Lee UY, Kim H, Song JK, Kim DH, Ahn KJ, Kim YS. Assessment of nasal profiles for forensic facial approximation in a modern Korean population of known age and sex. Leg Med (Tokyo). 2020;42:101646. | Title |
|  | Lee Y, Chun YS, Kang N, Kim M. Volumetric changes in the upper airway after bimaxillary surgery for skeletal class iii malocclusions: A case series study using 3-dimensional cone-beam computed tomography. Journal of Oral and Maxillofacial Surgery. 2012;70(12):2867-75. | Title |
|  | Lee YS, Baik HS, Lee KJ, Yu HS. The structural change in the hyoid bone and upper airway after orthognathic surgery for skeletal class III anterior open bite patients using 3-dimensional computed tomography. Korean Journal of Orthodontics. 2009;39(2):72-82. | Title |
|  | Lee, D., et al., Disruption of tracheobronchial airway growth following postnatal exposure to ozone and ultrafine particles. Inhalation Toxicology, 2011. 23(9): p. 520-531. | Title |
|  | Lee JY et al. Changes in the maxillary sinus volume and the surgical outcome after the canine fossa puncture approach in pediatric patients with an antrochoanal polyp:Results of a minimum 3-year follow-up.Am Journal of Rhinology and Allergy2009.23(5):531-534. | Title |
|  | Lee, K., et al., Clinical experience of using multidetector- Row CT for the diagnosis of disorders in cattle. Veterinary Record, 2009. 165(19): p. 559-562. | Title |
|  | Lee, U.Y., et al., Morphological characteristics of the infraorbital foramen and infraorbital canal using three-dimensional models. Surgical and Radiologic Anatomy, 2006. 28(2): p. 115-120. | Title |
|  | Lee, W.Y., et al., Change of the airway space in mandibular prognathism after bimaxillary surgery involving maxillary posterior impaction. Maxillofacial Plastic and Reconstructive Surgery, 2016. 38(1). | Title |
|  | Lee, Y.S., et al., Using 3D-CT to analyze structural changes in the hyoid bone and upper airway after orthognathic surgery in patients with skeletal Class III anterior open bite, in Computed Tomography: New Research. 2013. p. 165-179. | Title |
|  | Lei, J., et al., Effect of extraction on condylar process position and upper airway in 12-14 years old females with skeletal Class Ⅱ high angle. Shanghai kou qiang yi xue = Shanghai journal of stomatology, 2020. 29(3): p. 281-286. | Title |
|  | Leibel SL, McVicar RN, Winquist AM, Snyder EY. Generation of 3D Whole Lung Organoids from Induced Pluripotent Stem Cells for Modeling Lung Developmental Biology and Disease. Jove-Journal of Visualized Experiments. 2021(170). | Title |
|  | Lenza MG, Lenza MM, Dalstra M, Melsen B, Cattaneo PM. An analysis of different approaches to the assessment of upper airway morphology: a CBCT study. Orthod Craniofac Res. 2010;13(2):96-105. | Title |
|  | Levi, G., Y. Gitton, and N. Narboux-Nême, Transitory expression of Dlx5 and Dlx6 in maxillary arch epithelial precursors is essential for upper jaw morphogenesis. F1000Research, 2014. 2. | Title |
|  | Levine JP, Bradley JP, Shahinian HK, Longaker MT. Nasal expansion in the fetal lamb: A first step toward management of cleft nasal deformity in utero. Plastic and Reconstructive Surgery. 1999;103(3):761-7. | Title |
|  | Levy Vehel J. 3-D fractal model for lung morphogenesis.Proceedings of SPIE The International Society for Optical Engineering. 1990. | Title |
|  | Lezhnev, D.A., D.V. Davydov, and M.O. Dutova, The possibilities of multislice computed tomography in nasal anthropometry. Acta Biomedica Scientifica, 2019. 3(6): p. 121-125. | Title |
|  | Li H, Lu X, Shi J, Shi H. Measurements of normal upper airway assessed by 3-dimensional computed tomography in Chinese children and adolescents. International Journal of Pediatric Otorhinolaryngology. 2011;75(10):1240-6. | Full text  No skeletal assessment |
|  | Li H, Sun C, Chen Y, Sun Z, Gao X. Quantitative changes of upper airway in class III patients undergoing bimaxillary surgery after one-year follow-up: a retrospective study. Head and Face Medicine. 2022;18(1). | Title |
|  | Li J, Ge XL, Guan HB, Zhang SL, Qiao X, Chang WW, et al. Three-dimensional changes of the upper airway in patients with Class II malocclusion treated with functional appliances: a systematic review and meta-analysis. Eur J Orthod 2021;43(4):415-23. | Title |
|  | Li J, Liu Y, Yuan X, Ding X, Guo Y, Qiu L. Dynamical Changes of Mandible and Upper Airway After Mandibular Distraction Osteogenesis in Pierre Robin Sequence. J Craniofac Surg. 2020;31(2):513-6. | Title |
|  | Li J, Zhao Z, Zheng L, Daraqel B, Liu J, Hu Y. Effects of mouth breathing on maxillofacial and airway development in children and adolescents with different cervical vertebral maturation stages: a cross-sectional study. BMC Oral Health. 2022;22(1):197. | Abstract |
|  | Li K, Guilleminault C, Amat P. [Maxillomandibular Advancement for OSA: A 25-year perspective]. Orthod Fr. 2022;93(1):79-92. | Title |
|  | Li K, Guilleminault C, Amat P. [Treatment of OSA by maxillomandibular advancement: Serious Complications and Failures]. Orthod Fr. 2022;93(2):155-68. | Title |
|  | Li K, Iwasaki T, Quo S, Li C, Young K, Leary E, et al. [Persistent pediatric obstructive sleep apnea treated with skeletally anchored transpalatal distraction]. Orthod Fr. 2022;93(2):139-53. | Title |
|  | Li K, Li ZJ, Zhang SJ, Gao S, Wang X, Sun H, et al. Titanium plate placement for internal fixation of slopes in children aged 1-6 years: Digital morphological analysis of mechanical support. Chinese Journal of Tissue Engineering Research. 2021;25(27):4289-93. | Title |
|  | Li L, Liu H, Cheng H, Han Y, Wang C, Chen Y, et al. CBCT Evaluation of the upper airway morphological changes in growing patients of class ii division 1 malocclusion with mandibular retrusion using twin block appliance: A comparative research. PLoS ONE. 2014;9(4). | Title |
|  | Li L, Wu W, Yan G, Liu L, Liu H, Li G, et al. Analogue simulation of pharyngeal airflow response to Twin Block treatment in growing patients with Class II(1) and mandibular retrognathia. Sci Rep. 2016;6:26012. | Title |
|  | Li Q, Tang H, Liu X, Luo Q, Jiang Z, Martin D, et al. Comparison of dimensions and volume of upper airway before and after mini-implant assisted rapid maxillary expansion. Angle Orthod. 2020;90(3):432-41. | Title |
|  | Li SJ, Zhang PY, Chen YJ, Fang L, Bai YJ, Zhao ZM. Influence of Bone-Borne Trans-Sutural Distraction Osteogenesis Therapy on the Hard Palate of Growing Children With Cleft Lip and Palate. Journal of Craniofacial Surgery. 2022;33(2):390-4. | Title |
|  | Li WB, Zankl M, Schlattl H, Petoussi-Henss N, Eckerman KF, Bolch WE, et al. Impact on 141Ce, 144Ce, 95Zr, and 90Sr beta emitter dose coefficients of photon and electron SAFs calculated with ICRP/ICRU reference adult voxel computational phantoms. Health Phys. 2010;99(4):503-10. | Title |
|  | Li YM, Liu JL, Zhao JL, Dai J, Wang L, Chen JW. Morphological changes in the pharyngeal airway of female skeletal class III patients following bimaxillary surgery: A cone beam computed tomography evaluation. International Journal of Oral and Maxillofacial Surgery. 2014;43(7):862-7. | Title |
|  | Li Z, Zhang F, Duan Y, Xu Y, Yuan Y, Lei Y. [Three dimensional measurement of craniofacial hard tissues for the Han nationality with normal occlusion in Changsha area]. Zhong Nan Da Xue Xue Bao Yi Xue Ban. 2018;43(1):58-67. | Title |
|  | **Li ZM, Wu J, Men HY, Li HF. Cone-beam CT study for the oropharyngeal airway volume and hyoid position of adults Class III skeletal malocclusion. Shanghai kou qiang yi xue [Shanghai journal of stomatology]. 2015;24(3):351‐5.** | **Included** |
|  | Li, C., et al., Wnt5a participates in distal lung morphogenesis. Developmental Biology, 2002. 248(1): p. 68-81. | Title |
|  | Li, F. and C.W. Chen, 3D warping and registration from lung images. Proceedings of SPIE - The International Society for Optical Engineering, 1999. 3660: p. 459-470. | Title |
|  | Li, L., et al., Analysis of nasal cavity morphology and nasolabial development of the normal Han ethnic people under age of 12. European Review for Medical and Pharmacological Sciences, 2016. 20(10): p. 1923-1933. | Title |
|  | Li, P., et al., Improved vacuum-assisted closure therapy for diabetic wounds that were difficult to heal and accompanied by chronic narrow sinus: A case series of five patients. International Journal of Clinical and Experimental Medicine 2017. 10(11): p. 15229-15236. | Title |
|  | Li, Q.Y., et al., The pre-styloid compartment of the parapharyngeal space: A three-dimensional digitized model based on the Chinese Visible Human. Surgical and Radiologic Anatomy, 2004. 26(5): p. 411-416. | Title |
|  | Li, S., M. Wan, and S. Wang, The effects of the false vocal fold gaps in a model of the larynx on pressures distributions And Flows, in Lecture Notes in Computer Science (including subseries Lecture Notes in Artificial Intelligence and Lecture Notes in Bioinformatics). 2007. p. 147-156. | Title |
|  | Li, W. and Y. Wang, Long wooden stick penetrating across the retromandibular, nasopharyngeal and contralateral orbital region in a child. International Journal of Pediatric Otorhinolaryngology Extra, 2009. 4(2): p. 88-91. | Title |
|  | Li, X., et al., Effect of functional appliance on upper airway in adolescent patients with skeletal Class II malocclusion. Shanghai kou qiang yi xue = Shanghai journal of stomatology, 2017. 26(2): p. 222-227. | Title |
|  | Li, X.D., X.M. Gao, and X.L. Zeng, A magnetic resonance imaging research of upper airway and surrounding tissues of 83 non-snoring males. Beijing da xue xue bao. Yi xue ban = Journal of Peking University. Health sciences, 2005. 37(2): p. 190-194. | Full text  No skeletal groups |
|  | Li, Z., et al., Angiogenic potential of human bone marrow-derived mesenchymal stem cells in chondrocyte brick-enriched constructs promoted stable regeneration of craniofacial cartilage. Stem Cells Translational Medicine, 2017. 6(2): p. 601-612. | Title |
|  | Liang X, Chen P, Chen C, Che W, Yang Y, Tan Z, et al. Comprehensive risk assessments and anesthetic management for children with osteogenesis imperfecta: A retrospective review of 252 orthopedic procedures over 5 years. Paediatric Anaesthesia. 2022;32(7):851-61. | Title |
|  | Liang, T.K., et al. Segmentation of airway trees from multislice CT using fuzzy logic. in Conference Record - Asilomar Conference on Signals, Systems and Computers. 2009. | Title |
|  | Liberton DK, Claes P, McEvoy B, Beleza S, Barsh G, Tang H, et al. Using automated high density quasi-landmarks to test for associations between normal facial feature variation, genetic ancestry and candidate gene variation in Cape Verdeans. American Journal of Physical Anthropology. 2011;144:198. | Title |
|  | Liegeois F, Albert A, Limme M. Comparison between tongue volume from magnetic resonance images and tongue area from profile cephalograms. European Journal of Orthodontics. 2010;32(4):381-6. | Title |
|  | Likus W, Bajor G, Gruszczyńska K, Baron J, Markowski J. Nasal region dimensions in children: A CT study and clinical implications. BioMed Research International. 2014;2014. | Abstract |
|  | Lim HM, Park YC, Lee KJ, Kim KH, Choi YJ. Stability of dental, alveolar, and skeletal changes after miniscrew-assisted rapid palatal expansion. Korean Journal of Orthodontics. 2017;47(5):313-22. | Title |
|  | Lim S. Two separate pathologies (Coeliac disease and Central precocious puberty) associated with catch-up growth in the case of a child born small for gestational age (SGA). Hormone Research in Paediatrics. 2019;91:593. | Title |
|  | Lim TC, Mokal NJ, Tan WT. Management of concomitant maxillofacial, cervical spine and laryngeal trauma--case reports. Ann Acad Med Singap. 1997;26(1):108-12. | Title |
|  | Lim, H., et al., Sputum plug selection under inverted microscopy improves microbial identification during exacerbations of airway diseases. Respiratory Medicine, 2018. 134: p. 92-94. | Title |
|  | Lim, H.C., et al., Application of a collagenated biphasic calcium phosphate loaded with fibroblast growth factor-2 in the rabbit sinus: A pilot study. International Journal of Oral and Maxillofacial Implants, 2015. 30(5): p. 1197-1204. | Title |
|  | Lima, Y.K., et al., Three-dimensional evaluation of soft tissue change gradients after mandibular setback surgery in skeletal Class III malocclusion. Angle Orthodontist, 2010. 80(5): p. 896-903. | Title |
|  | Lin C, Wiemken AS, Leinwand SE, Wang SH, Keenan BT, Wang J, et al. Interethnic comparison of inter-mandibular and soft tissue volumes among native chinese, icelandic caucasian and African-American apneics. Sleep. 2017;40:A162. | Title |
|  | Lin CH, Liao YF, Chen NH, Lo LJ, Chen YR. Three-dimensional computed tomography in obstructive sleep apneics treated by maxillomandibular advancement. Laryngoscope. 2011;121(6):1336-47. | Title |
|  | Lin SL, Ji C, Xu L, Zhao ZY. Assessment of facial growth for children based on three-dimensional face database. Chinese Journal of Biomedical Engineering. 2014;33(5):546-55. | Title |
|  | Lin XZ. Correlation study of increase of pharyngeal airway space after mandibular advancement, taking natural head position into consideration. British Journal of Oral & Maxillofacial Surgery. 2019;57(8):760-4. | Title |
|  | Lin ZC, CS Jian, and RY Wang. A study of simulation of three-dimensional quasi-steady molecular statics nanoscale orthogonal cutting of single-crystal silicon material having rows of atoms with vacancy defect. Journal of the Chinese Society of Mechanical Engineers, Transactions Chinese Institute of Engineers, Series C/Chung-Kuo Chi Hsueh Kung Ch'eng Hsuebo Pao,2014. 35(2):93-100. | Title |
|  | Lindbaek M, Melby KK, Schøyen R, Hjortdahl P. Bacteriological findings in nasopharynx specimens from patients with a clinical diagnosis of acute sinusitis. Scand J Prim Health Care. 2001;19(2):126-30. | Title |
|  | Lindsay, K.E., F.J. Rühli, and V.B. Deleon, Revealing the Face of an Ancient Egyptian: Synthesis of Current and Traditional Approaches to Evidence-Based Facial Approximation. Anatomical Record, 2015. 298(6): p. 1144-1161. | Title |
|  | Lindsey SE, Butcher JT, Vignon-Clementel IE. Cohort-based multiscale analysis of hemodynamic-driven growth and remodeling of the embryonic pharyngeal arch arteries. Development. 2018;145(20). | Title |
|  | Lindsey SE, Menon PG, Kowalski WJ, Shekhar A, Yalcin HC, Nishimura N, et al. Growth and hemodynamics after early embryonic aortic arch occlusion. Biomechanics and Modeling in Mechanobiology. 2015;14(4):735-51. | Title |
|  | Lindsey SE, Vignon-Clementel IE, Butcher JT. Assessing Early Cardiac Outflow Tract Adaptive Responses Through Combined Experimental-Computational Manipulations. Annals of Biomedical Engineering. 2021;49(12):3227-42. | Title |
|  | Linnau KF, Stanley Jr RB, Hallam DK, Gross JA, Mann FA. Imaging of high-energy midfacial trauma: What the surgeon needs to know. European Journal of Radiology. 2003;48(1):17-32. | Title |
|  | Lintermann A, Schroder W. A Hierarchical Numerical Journey Through the Nasal Cavity: from Nose-Like Models to Real Anatomies. Flow Turbulence and Combustion. 2019;102(1):89-116. | Title |
|  | Lintner M, Weissenbacher A, Heiss E. The Oropharyngeal Morphology in the Semiaquatic Giant Asian Pond Turtle, Heosemys grandis, and Its Evolutionary Implications. Plos One. 2012;7(9). | Title |
|  | Linton S, Vinoo A, Cadden F, Mani N. Skull base chondroblastoma presenting as a deep lobe parotid tumour. BMJ Case Rep. 2021;14(2). | Title |
|  | Lione R, Franchi L, Ghislanzoni LTH, Primozic J, Buongiomo M, Cozza P. Palatal surface and volume in mouth-breathing subjects evaluated with three-dimensional analysis of digital dental casts-a controlled study. European Journal of Orthodontics.2015;37:101-4. | Title |
|  | Lione, R., et al., Evaluation of maxillary arch dimensions and palatal morphology in mouth-breathing children by using digital dental casts. International Journal of Pediatric Otorhinolaryngology, 2014. 78(1): p. 91-95. | Title |
|  | Liu CN, Kang KT, Yao CCJ, Chen YJ, Lee PL, Weng WC, et al. Changes in Cone-Beam Computed Tomography Pediatric Airway Measurements After Adenotonsillectomy in Patients With OSA. Jama Otolaryngology-Head & Neck Surgery. | Title |
|  | Liu D, Alhazmi N, Hecht JT, Wehby GL, Moreno LM, Heike CL, et al. Exome-wide low-frequency genetic variants contribute to human craniofacial morphology. Genetic Epidemiology. 2019;43(7):893-4. | Title |
|  | Liu F, Zhang ZF, Skovsted CB. Advances in the soft anatomy and skeletal microstructures of Cambrian hyoliths in China and their implications for lophotrochozoan evolution. Chinese Science Bulletin-Chinese. 2021;66(27):3631-44. | Title |
|  | Liu J, Chen Y, Li F, Wu W, Hao J, Luo D, et al. Condylar positions before and after bilateral mandibular distraction osteogenesis in children with Pierre Robin sequence. International Journal of Oral and Maxillofacial Surgery. 2018;47(1):57-63. | Title |
|  | Liu JP, Yao XH, Wang ZW, Ye J, Luan CC, He Y, et al. A flexible porous chiral auxetic tracheal stent with ciliated epithelium. Acta Biomaterialia. 2021;124:153-65. | Title |
|  | Liu L, Li J, Ji H, Zhang N, Wang Y, Zheng G, et al. Cone-beam computed tomography evaluation of the maxillofacial features of patients with unilateral temporomandibular joint ankylosis undergoing condylar reconstruction with an autogenous coronoid process graft. PLoS ONE. 2017;12(3). | Title |
|  | Liu S, Gao X. Nasopharyngeal changes during healthy children of 8-13 years old in China: A longitudinal study. Sleep Medicine. 2017;40:e197. | Title |
|  | Liu S, Zhou Y, Gao X. Growth and development of upper airway and surrounding tissues in non-snoring children. Sleep Medicine. 2015;16:S47. | Title |
|  | Liu SYC, Huon LK, Lo MT, Chang YC, Capasso R, Chen YJ, et al. Static craniofacial measurements and dynamic airway collapse patterns associated with severe obstructive sleep apnoea: a sleep MRI study. Clinical Otolaryngology. 2016;41(6):700-6. | Title |
|  | Liu XD, Skold CM, Umino T, Spurzem JR, Romberger DJ, Rennard SI. Sodium nitroprusside augments human lung fibroblast collagen gel contraction independently of NO-cGMP pathway. American Journal of Physiology-Lung Cellular and Molecular Physiology. 2000;278(5):L1032-L8. | Title |
|  | Liu Y, Yang K. Three-dimensional changes in the upper airway and craniomaxillofacial morphology of patients with Angle Class III malocclusion treated with a Frankel III appliance. BMC Oral Health. 2021;21(1):634. | Title |
|  | Liu Y. Unusual Illustration of Richter Transformation in Chronic Lymphocytic Leukemia on FDG PET/CT. Clin Nucl Med. 2022;47(8):746-7. | Title |
|  | Liu Z, Anderson JD, Deng L, Mackay S, Zhao R, Liu ZL, et al. Fully differentiated nasal epithelial organoids for the study of CF. Pediatric Pulmonology. 2018;53:261-2. | Title |
|  | Liu, S.S., et al., Nasopharyngeal changes in 8-13 years old healthy children in China: a longitudinal study. Zhonghua er bi yan hou tou jing wai ke za zhi = Chinese journal of otorhinolaryngology head and neck surgery, 2016. 51(10): p. 733-739. | Abstract |
|  | Lkhagvadorj K, Zeng ZJ, Song J, Reinders-Luinge M, Kooistra W, Song SS, et al. Prenatal smoke exposure dysregulates lung epithelial cell differentiation in mouse offspring: role for AREG-induced EGFR signaling. American Journal of Physiology-Lung Cellular and Molecular Physiology. 2020;319(4):L742-L51. | Title |
|  | Lloyd G, Howard D, Lund VJ, Savy L. Imaging for juvenile angiofibroma. J Laryngol Otol. 2000;114(9):727-30. | Title |
|  | Lo Giudice A, Ronsivalle V, Gastaldi G, Leonardi R. Assessment of the accuracy of imaging software for 3D rendering of the upper airway, usable in orthodontic and craniofacial clinical settings. Progress in Orthodontics. 2022;23(1). | Title |
|  | Lo Giudice A, Spinuzza P, Rustico L, Messina G, Nucera R. Short-term treatment effects produced by rapid maxillary expansion evaluated with computed tomography: A systematic review with meta-analysis. Korean Journal of Orthodontics. 2020;50(5):314-23. | Title |
|  | Lobbezoo, F., et al., Lack of Associations between Occlusal and Cephalometric Measures, Side Imbalance in Striatal D2 Receptor Binding, and Sleep-Related Oromotor Activities. Journal of Orofacial Pain, 2001. 15(1): p. 64-71. | Title |
|  | Longaker MT, Stern M, Lorenz HP, Whitby DJ, Dodson TB, Harrison MR, et al. A MODEL FOR FETAL CLEFT-LIP REPAIR IN LAMBS. Plastic and Reconstructive Surgery. 1992;90(5):750-6. | Title |
|  | Longest PW, Golshahi L, Farkas D, Tian G, Behara SRB, Hindle M. Simultaneous administration of low flow nasal cannula oxygen support and pharmaceutical aerosols. American Journal of Respiratory and Critical Care Medicine. 2015;191. | Title |
|  | Longest, P.W., et al., Aerodynamic factors responsible for the deaggregation of carrier-free drug powders to form micrometer and submicrometer aerosols. Pharmaceutical Research, 2013. 30(6): p. 1608-1627. | Title |
|  | Looby JF, Schendel SA, Lorenz HP, Hopkins EM, Aizenbud D. Airway Analysis: With Bilateral Distraction of the Infant Mandible. Journal of Craniofacial Surgery. 2009;20(5):1341-6. | Title |
|  | Lopes IA, Tucunduva RMA, Handem RH, Capelozza ALA. Study of the frequency and location of incidental findings of the maxillofacial region in different fields of view in CBCT scans. Dentomaxillofacial Radiology. 2016;46(1). | Title |
|  | Lopes L, Afonso J, Graça A, Teixeira S, Moura F. An inusual case in obstetrics-smart and safe management. Anesthesia and Analgesia. 2021;133(3 SUPPL 2):1021. | Title |
|  | Lopez JI, Perez A. Pharyngeal adenocarcinoma with intestinal features. Journal of Laryngology and Otology. 1990;104(11):900-2. | Title |
|  | Lopez-Mateos MLM, Carreno-Carreno J, Palma JC, Alarcon JA, Lopez-Mateos CM, Menendez-Nunez M. Three-dimensional photographic analysis of the face in European adults from southern Spain with normal occlusion: reference anthropometric measurements. Bmc Oral Health. 2019;19(1). | Title |
|  | Lorente C, Hernandez-Alfaro F, Perez-Vela M, Lorente P, Lorente T. Surgical-orthodontic approach for facial rejuvenation based on a reverse facelift. Progress in Orthodontics. 2019;20(1). | Title |
|  | Lotfi V, Ghoneima A, Lagravere M, Kula K, Stewart K. Three-dimensional evaluation of airway volume changes in two expansion activation protocols. International Orthodontics. 2018;16(1):144-57. | Title |
|  | Lovasova K. et al. Three-dimensional CAD/CAM imaging of the maxillary sinus in ageing process. Annals Anat 2018. 218: 69-82. | Title |
|  | Lovisi, C.B., et al., Immediate three-dimensional changes in the oropharynx after different mandibular advancements in counterclockwise rotation orthognathic planning. Journal of Clinical and Experimental Dentistry, 2021. 13(4): p. e334-e341. | Title |
|  | Lowe AA, Fleetham JA, Adachi S, Ryan CF. Cephalometric and computed tomographic predictors of obstructive sleep apnea severity. Am J Orthod Dentofacial Orthop. 1995;107(6):589-95. | Title |
|  | Lowth A, Juge L, Knapman F, Burke P, Brown E, Butler J, et al. Dynamic mri tongue deformation patterns during mandibular advancement and associations with craniofacial anatomy in OSA. Journal of Sleep Research. 2018;27. | Title |
|  | Lowth A, Sutherland K, Antic N, Carney S, Catcheside PG, Chai-Coetzer CL, et al. MRI analysis of tissue and airway volumes following upper airway surgery for Obstructive Sleep Apnoea. Journal of sleep research. 2019;28(SUPPL 1). | Title |
|  | Lu A, Hargett J, Gillis J. eP179: Expanding the phenotype of CLCN6-associated early-onset neurodegeneration. Genetics in Medicine. 2022;24(3):S108-S11. | Title |
|  | Lu-Emerson, C., et al., Management of ischemic stroke: Part 1. Emergency room management. Journal of Hospital Medicine, 2010. 5(1): p. 33-40. | Title |
|  | Luengen AE, Cheremkhina M, Gonzalez-Rubio J, Weckauf J, Kniebs C, Uebner H, et al. Bone Marrow Derived Mesenchymal Stromal Cells Promote Vascularization and Ciliation in Airway Mucosa Tri-Culture Models in Vitro. Frontiers in Bioengineering and Biotechnology. 2022;10. | Title |
|  | Lundeen IK, Kirk EC. Internal nasal morphology of the Eocene primate Rooneyia viejaensis and extant Euarchonta: Using mu CT scan data to understand and infer patterns of nasal fossa evolution in primates. Journal of Human Evolution. 2019;132:137-73. | Title |
|  | Luo CH, Zheng ZJ, Xu WH, Wang QY. Three-dimensional reconstruction of the nasomaxillary complex and upper airway following rapid maxillary expansion by cone-beam CT. Chinese Journal of Tissue Engineering Research. 2017;21(36):5781-6. | Title |
|  | Luo S, Yan Y. Implementation of a virtual laryngoscope system using efficient reconstruction algorithms. Med Sci Monit. 2009;15(8):Mt95-100. | Title |
|  | Luo WW, Kao MT, Liu JN. Echolocation precursor calls of Kerivoula furva pups may contain individual signatures. Acta Chiropterologica. 2020;22(2):337-48. | Title |
|  | Luscan, R., et al., Developmental changes of upper airway dimensions in children. Paediatric Anaesthesia, 2020. 30(4): p. 435-445. | Abstract |
|  | Luu, B.L., et al., Tongue acceleration in humans evoked with intramuscular electrical stimulation of genioglossus. Respiratory Physiology and Neurobiology, 2022. 295. | Title |
|  | Lypka M, Goldstein J, Hendricks H. Long-term assessment of pierre robin sequence patients treated with a vertically-oriented mandibular distraction vector. Cleft Palate-Craniofacial Journal. 2019;56(1):46. | Title |
|  | M. Harandi, N., et al., Variability in muscle activation of simple speech motions: A biomechanical modeling approach. Journal of the Acoustical Society of America, 2017. 141(4): p. 2579-2590. | Title |
|  | Ma, Y., et al., Novel compound heterozygous mutations of PCNT gene in MOPD type II with central precocious puberty. Gynecological Endocrinology, 2021. 37(2): p. 190-192. | Title |
|  | Ma, Y., M. Yu, and X. Gao, The effect of gradually increased mandibular advancement on the efficacy of an oral appliance in the treatment of obstructive sleep apnea. Journal of Clinical Sleep Medicine, 2020. 16(8): p. 1369-1376. | Title |
|  | Maal T, Kau CH, Borstlap W, Berge S. Facial morphology of adult dutch, egyptian and texan white population using 3D stereophotogrammetry. International Journal of Computer Assisted Radiology and Surgery. 2011;6:S204-S5. | Title |
|  | Macari AT, Haddad RV. The case for environmental etiology of malocclusion in modern civilizations-Airway morphology and facial growth. Seminars in Orthodontics. 2016;22(3):223-33. | Title |
|  | Machado GL. CBCT imaging - A boon to orthodontics. Saudi Dental Journal. 2015;27(1):12-21. | Title |
|  | Machado, M.C., K.M. Tarquinio, and T.J. Webster. Bacterial colonization of nanomodified endotracheal tubes in a bench top airway model. in AIChE Annual Meeting, Conference Proceedings. 2011. | Title |
|  | Macrini TE. COMPARATIVE MORPHOLOGY OF THE INTERNAL NASAL SKELETON OF ADULT MARSUPIALS BASED ON X-RAY COMPUTED TOMOGRAPHY. Bulletin of the American Museum of Natural History. 2012(365):1-+. | Title |
|  | Macrini TE. Development of the Ethmoid in Caluromys philander (Didelphidae, Marsupialia) With a Discussion on the Homology of the Turbinal Elements in Marsupials. Anatomical Record-Advances in Integrative Anatomy and Evolutionary Biology. 2014;297(11):2007-17. | Title |
|  | MacRini, T.E., Comparative morphology of the internal nasal skeleton of adult marsupials based on X-ray computed tomography, in Bulletin of the American Museum of Natural History. 2012. p. 1-91. | Title |
|  | Maddux, S.D. and L.N. Butaric, Zygomaticomaxillary Morphology and Maxillary Sinus Form and Function: How Spatial Constraints Influence Pneumatization Patterns among Modern Humans. Anatomical Record, 2017. 300(1): p. 209-225. | Title |
|  | Mafee MF, Tran BH, Chapa AR. Imaging of rhinosinusitis and its complications: plain film, CT, and MRI. Clin Rev Allergy Immunol. 2006;30(3):165-86. | Title |
|  | Mafee MF, Valvassori GE. Radiology of the craniofacial anomalies. Otolaryngologic Clinics of North America 1981. 14(4): 939-988. | Title |
|  | Magnusson A, Bjerklin K, Kim H, Nilsson P, Marcusson A. Three-dimensional computed tomographic analysis of changes to the external features of the nose after surgically assisted rapid maxillary expansion and orthodontic treatment: A prospective longitudinal study. American Journal of Orthodontics and Dentofacial Orthopedics. 2013;144(3):404-13. | Title |
|  | Magnusson A. Evaluation of surgically assisted rapid maxillary expansion and orthodontic treatment. Effects on dental, skeletal and nasal structures and rhinological findings. Swed Dent J Suppl. 2013(229):1-104. | Title |
|  | Magro I, Pastel D, Hilton J, Miller M, Saunders J, Noonan K. Developmental Anatomy of the Eustachian Tube: implications for Balloon Dilation. Otolaryngology--head and neck surgery. 2021. | Title |
|  | Mah JK, Huang JC, Choo H. Practical applications of cone-beam computed tomography in orthodontics. J Am Dent Assoc. 2010;141 Suppl 3:7s-13s. | Abstract |
|  | Mah, J.K., et al., Advanced Applications of Cone Beam Computed Tomography in Orthodontics. Seminars in Orthodontics, 2011. 17(1): p. 57-71. | Title |
|  | Mahapatra AK, Dev EJ, Krishnan A, Sharma RR. Craniofacial surgery for leaking encephalocele in a newborn baby. Childs Nerv Syst. 2001;17(10):626-8. | Title |
|  | Mahdy MAA, Zayed M. Computed tomography and cross-sectional anatomy of the head in the red fox (Vulpes vulpes). Anatomia Histologia Embryologia. 2020;49(6):708-17. | Title |
|  | Mahrous Mohamed A, Al Bishri A, Haroun Mohamed A. Distraction osteogenesis as followed by CT scan in Pierre Robin sequence. Journal of Cranio-Maxillofacial Surgery. 2011;39(6):412-9. | Title |
|  | Maina JN, Madan AK, Alison B. Expression of fibroblast growth factor-2 (FGF-2) in early stages (days 3-11) of the development of the avian lung, Gallus gallus variant domesticus: an immunocytochemical study. Journal of Anatomy. 2003;203(5):505-12. | Title |
|  | Maisano, J.A., M. Kearney, and T. Rowe, Cranial anatomy of the spade-headed amphisbaenian Diplometopon zarudnyi (Squamata, Amphisbaenia) based on high-resolution X-ray computed tomography. Journal of Morphology, 2006. 267(1): p. 70-102. | Title |
|  | Majewski, S., et al., Second-trimester prenatal diagnosis of total arhinia. Journal of Ultrasound in Medicine, 2007. 26(3): p. 391-395. | Title |
|  | Major MP, Saltaji H, El-Hakim H, Witmans M, Major P, Flores-Mir C. The accuracy of diagnostic tests for adenoid hypertrophy A systematic review. Journal of the American Dental Association. 2014;145(3):247-54. | Title |
|  | Major MP, Witmans M, El-Hakim H, Major PW, Flores-Mir C. Agreement between cone-beam computed tomography and nasoendoscopy evaluations of adenoid hypertrophy. Am J Orthod Dentofacial Orthop. 2014;146(4):451-9. | Title |
|  | Makris, N., et al., MRI-based anatomical model of the human head for specific absorption rate mapping. Medical and Biological Engineering and Computing, 2008. 46(12): p. 1239-1251. | Title |
|  | Malm A, Chudzik B, Piersiak T, Gawron A. Glass surface as potential in vitro substratum for Candida Famata Biofilm. Annals of Agricultural and Environmental Medicine. 2010;17(1):115-8. | Title |
|  | Manansala M, Kannan J, Patel P, Mehta A, Utset M, Pytel P, et al. Hydroxychloroquine-related neuromyopathic and cardiac toxicity. Journal of Investigative Medicine. 2020;68(5):1068-70. | Title |
|  | Mannelli G, Arcuri F, Spacca B, Genitori L, Spinelli G. Respiratory and volumetric changes of the upper airways in craniofacial synostosis patients. Journal of Cranio-Maxillofacial Surgery. 2019;47(4):548-55. | Title |
|  | Manyama M, Larson JR, Liberton DK, Rolian C, Smith FJ, Kimwaga E, et al. Facial morphometrics of children with NON-syndromic orofacial clefts in Tanzania. Bmc Oral Health. 2014;14. | Title |
|  | Mao Z, Zhang N, Cui Y. A clinical prediction rule to identify difficult intubation in children with Robin sequence requiring mandibular distraction osteogenesis based on craniofacial CT measures. BMC Anesthesiology. 2019;19(1). | Title |
|  | Mao Z, Zhang N, Cui Y. Three-dimensional printing of surgical guides for mandibular distraction osteogenesis in infancy. Medicine (Baltimore). 2019;98(10):e14754. | Title |
|  | Mao, X.Y., et al., Evaluation of the upper airway and maxillary and mandibular characters in adult skeletal Class Ⅱ malocclusion. Shanghai kou qiang yi xue = Shanghai journal of stomatology, 2021. 30(4): p. 419-423. | Full text  Other outcomes |
|  | Marcussen L, Henriksen JE, Thygesen T. Do mandibular advancement devices influence patients' snoring and obstructive sleep apnea? a cone-beam computed tomography analysis of the upper airway volume. Journal of Oral and Maxillofacial Surgery.2015;73:1816-26. | Title |
|  | Marcussen L, Stokbro K, Aagaard E, Torkov P, Thygesen T. Changes in Upper Airway Volume Following Orthognathic Surgery. J Craniofac Surg. 2017;28(1):66-70. | Title |
|  | Marečková, K., et al., Testosterone-mediated sex differences in the face shape during adolescence: Subjective impressions and objective features. Hormones and Behavior, 2011. 60(5): p. 681-690. | Title |
|  | Maresky HS, Klar MM, Tepper J, Gavriel H, Baran TZ, Shapiro CM, et al. Mandibular width as a novel anthropometric measure for assessing obstructive sleep apnea risk. Medicine (United States). 2019;98(4). | Title |
|  | Maret, D., et al., Recent advances in cone-beam CT in oral medicine. Current Medical Imaging Reviews, 2020. 16(5): p. 553-564. | Title |
|  | Margetis, K., P.J. Christos, and M. Souweidane, Endoscopic resection of incidental colloid cysts: Clinical article. Journal of Neurosurgery, 2014. 120(6): p. 1259-1267. | Title |
|  | Mǎrginean, C., et al., The three-dimensional ultrasonography of the fetal face--history and progress. Revista medico-chirurgicalǎ̌ a Societǎ̌ţii de Medici ş̧i Naturaliş̧ti din Iaş̧i, 2010. 114(4): p. 1058-1063. | Title |
|  | Marianetti TM, Gasparini G, Moro A, Alimonti V, Cervelli D, Boniello R, et al. Nasal and Ethmoidal Alterations in Anterior Synostotic Plagiocephaly. Journal of Craniofacial Surgery. 2011;22(2):509-13. | Title |
|  | Marino, J. and A. Kaufman, Planar Visualization of Treelike Structures. IEEE Transactions on Visualization and Computer Graphics, 2016. 22(1): p. 906-915. | Title |
|  | Marks TN, Maddux SD, Butaric LN, Franciscus RG. Climatic adaptation in human inferior nasal turbinate morphology: Evidence from Arctic and equatorial populations. American Journal of Physical Anthropology. 2019;169(3):498-512. | Title |
|  | Marks, B., E. Schober, and H. Swoboda, Diffuse idiopathic skeletal hyperostosis causing obstructing laryngeal edema. European Archives of Oto-Rhino-Laryngology, 1998. 255(5): p. 256-258. | Title |
|  | Maroldi R, Battaglia G, Farina D, Maculotti P, Chiesa A. Tumours of the oropharynx and oral cavity: Perineural spread and bone invasion. Journal Belge de Radiologie. 1999;82(6):294-300. | Title |
|  | Márquez S, Laitman JT. Climatic effects on the nasal complex: A CT imaging, comparative anatomical, and morphometric investigation of Macaca mulatta and Macaca fascicularis. Anatomical Record. 2008;291(11):1420-45. | Title |
|  | Márquez S, Lawson W, Mowbray K, Delman BN, Laitman JT. CT Examination of Nose and Paranasal Sinuses of Egyptian Mummies and Three Distinct Human Population Groups: Anthropological and Clinical Implications. Anatomical Record. 2015;298(6):1072-84. | Title |
|  | Martin DP, Bhalla T, Tobias JD. Ultrasound for pediatric peripheral arterial and venous cannulation. Journal of clinical monitoring and computing. 2014;28:461‐2. | Title |
|  | Martinez H, Martinez A, Gonzalez C. Uva clinic obstructive sleep apnea: volumetric, structural and ahi changes after counter clockwise surgery. International Journal of Oral and Maxillofacial Surgery. 2019;48:165. | Title |
|  | Martino F, Di Mauro R, Paciaroni K, Gaziev J, Alfieri C, Greco L, et al. Pathogenesis of chronic rhinosinusitis in patients affected by β-thalassemia major and sickle cell anaemia post allogenic bone marrow transplant. International Journal of Pediatric Otorhinolaryngology. 2018;106:35-40. | Title |
|  | Martins LF, Vigorito JW. Cone beam tomographic study of facial structures characteristics at rest and wide smile, and their correlation with the facial types. Dental Press J Orthod. 2013;18(6):38-44. | Title |
|  | Martins, A., et al., Picking up the threads: Comparative osteology and associated cartilaginous elements for members of the genus Trilepida Hedges, 2011 (Serpentes, Leptotyphlopidae) with new insights on the Epictinae systematics. Anatomical Record, 2021. 304(10): p. 2149-2182. | Title |
|  | Martins, L.S., et al., Important queries for the airway analysis in cone-beam computed tomography scans: Threshold tool and voxel size protocol. Imaging Science in Dentistry, 2018. 6(2): p. 26-30. | Title |
|  | Maruani A, Brown S, Lorette G, Pondaven-Letourmy S, Herbreteau D, Eisenbaum A. Lack of Effect of Propranolol in the Treatment of Lymphangioma in Two Children. Pediatric Dermatology. 2013;30(3):383-5. | Title |
|  | Masiero S, Marchese Ragona R, Bottin R, Volante D, Ortolani M. An unusual cause of aspiration pneumonia. Aging Clin Exp Res. 2006;18(1):78-82. | Title |
|  | Mason K, Jordan M, Perry J. Volumetric and 3-dimensional assessment of growth of the nasopharyngeal structures from childhood to adolescence. Cleft Palate-Craniofacial Journal. 2018;55(1):56-7. | Title |
|  | Mason K, Perry J, Riski J, Fang X. Do age related changes exist between the level of velopharyngeal closure and the cervical spine? Cleft Palate-Craniofacial Journal. 2016;53(4):e125-e6. | Title |
|  | Mason K, Riski J, Perry J. Do external factors impact final pharyngoplasty tissue location and speech postsurgically? Cleft Palate-Craniofacial Journal. 2019;56(1):88. | Title |
|  | Mason K, Riski J, Perry J. Effect of scar contracture and gravity on postoperative tissue changes and speech outcomes following sphincter pharyngoplasties. Cleft Palate-Craniofacial Journal. 2018;55(1):116-7. | Title |
|  | Mason KN, Perry JL. Relationship Between Age and Diagnosis on Volumetric and Linear Velopharyngeal Measures in the Cleft and Noncleft Populations. Journal of Craniofacial Surgery. 2016;27(5):1340-5. | Title |
|  | Mason KN, Riski JE, Williams JK, Jones RA, Perry JL. Utilization of 3D MRI for the Evaluation of Sphincter Pharyngoplasty Insertion Site in Patients With Velopharyngeal Dysfunction. Cleft Palate-Craniofacial Journal. | Title |
|  | Masoud AI, Alwadei FH. Two-dimensional upper airway normative values in children aged 7 to 17 years. Cranio 2021. June 26; 1-8. Online ahead of print. | Title |
|  | Maspero C, Galbiati G, Del Rosso E, Farronato M, Giannini L. RME: effects on the nasal septum. A CBCT evaluation. European Journal of Paediatric Dentistry. 2019;20(2):123-6. | Title |
|  | Maspero, C., et al., Orthopaedic vs surgical palatal expansion. Dental Cadmos, 2008. 76(4): p. 57-79. | Title |
|  | Massaro C, Garib D, Cevidanes L, Janson G, Yatabe M, Lauris JRP, et al. Maxillary dentoskeletal outcomes of the expander with differential opening and the fan-type expander: a randomized controlled trial. Clinical oral investigations. 2021;25(9):5247‐56. | Title |
|  | Massenburg B, Mercan E, Ettinger R, Tse R. The Yin and Yang of Primary Unilateral Cleft Lip and Nose Repair: Opposing cleft and non-cleft side changes and the importance of balance in achieving harmony. Cleft Palate-Craniofacial Journal.2022;59(4 SUPPL):43-4. | Title |
|  | Massie JP, Bruckman K, Rifkin WJ, Runyan CM, Shetye PR, Grayson B, et al. The Effect of Nasoalveolar Molding on Nasal Airway Anatomy: A 9-Year Follow-up of Patients With Unilateral Cleft Lip and Palate. Cleft Palate Craniofac J. 2018;55(4):596-601. | Title |
|  | Massie JP, Runyan CM, Stern MJ, Alperovich M, Rickert SM, Shetye PR, et al. Nasal Septal Anatomy in Skeletally Mature Patients With Cleft Lip and Palate. JAMA Facial Plast Surg. 2016;18(5):347-53. | Title |
|  | Mastej EJ, DeBoer EM, Humphries SM, Cook MC, Hunter KS, Liptzin DR, et al. Lung and airway shape in neuroendocrine cell hyperplasia of infancy. Pediatric Radiology. 2018;48(12):1745-54. | Title |
|  | Mat Q, Mehta R, Lejeune D, D'Adesky C, Duterme JP. Nasal polyps with osseous metaplasia: A case report. B-ENT. 2017; 13:53. | Title |
|  | Mata M, Milián Medina L, Oliver Ferrándiz M, Sancho-Tello Valls M, Monleón Prada M, Martinez Ramos C, et al. 3d porous polycaprolactone scaffolds for cartilage regeneration. Artificial Organs. 2017;41(9):A79. | Title |
|  | Matharu, P., et al., Feeding Outcomes for Infants with Bronchopulmonary Dysplasia Discharged on Nasogastric Feeds. American Journal of Perinatology, 2021. 38(9): p. 897-900. | Title |
|  | Matos I, Sousa S, Coelho P. An unusual tongue base cyst: a case-report. International Journal of Oral and Maxillofacial Surgery. 2019;48:237. | Title |
|  | Matsumoto K, Nozoe E, Okawachi T, Ishihata K, Nishinara K, Nakamura N. Preliminary Analysis of the 3-Dimensional Morphology of the Upper Lip Configuration at the Completion of Facial Expressions in Healthy Japanese Young Adults and Patients With Cleft Lip. Journal of Oral and Maxillofacial Surgery. 2016;74(9):1834-46. | Title |
|  | Matsuo M, Mine Y, Kawahara K, Murayama T. Accuracy Evaluation of a Three-Dimensional Model Generated from Patient-Specific Monocular Video Data for Maxillofacial Prosthetic Rehabilitation: A Pilot Study. Journal of Prosthodontics-Implant Esthetic and Reconstructive Dentistry. 2020;29(8):712-7. | Title |
|  | Matsuoka, A., et al., Development of three-dimensional facial expression models using morphing methods for fabricating facial prostheses. Journal of Prosthodontic Research, 2019. 63(1): p. 66-72. | Title |
|  | Matthews H, Penington T, Saey I, Halliday J, Muggli E, Claes P. Spatially dense morphometrics of craniofacial sexual dimorphism in 1-year-olds. Journal of Anatomy. 2016;229(4):549-59. | Title |
|  | Matthews HS, Penington AJ, Hardiman R, Fan Y, Clement JG, Kilpatrick NM, et al. Modelling 3D craniofacial growth trajectories for population comparison and classification illustrated using sex-differences. Scientific Reports. 2018;8. | Title |
|  | Maurer J, Wiese-Rischke C, Walles T. Generation, optimization and characterization of a 3d-human airway model. European Surgical Research. 2021;62(SUPPL 1):18-9. | Title |
|  | Maurer JE, Sullivan SM, Currier GF, Kadioglu O, Li J. The airway implications in treatment planning two-jaw orthognathic surgery: The impact on minimum cross-sectional area. Seminars in Orthodontics. 2016;22(1):18-26. | Title |
|  | Maxwell MJ, Goldie RG, Henry PJ. Ca2+ signalling by endothelin receptors in rat and human cultured airway smooth muscle cells. Br J Pharmacol. 1998;125(8):1768-78. | Title |
|  | Mayer P, Pépin JL, Bettega G, Veale D, Ferretti G, Deschaux C, et al. Relationship between body mass index, age and upper airway measurements in snorers and sleep apnoea patients. European Respiratory Journal. 1996;9(9):1801-9. | Title |
|  | Mayorga J, Castellón L, Alister JP, Martinovic G. Mandibular reconstruction in pediatric patients using stereolitographic models: Report of cases. International Journal of Oral and Maxillofacial Surgery. 2011;40(10):e27. | Title |
|  | McCance, A.M., et al., Three-dimensional analysis techniques - Part 1: Three-dimensional soft- tissue analysis of 24 adult cleft palate patients following Le Fort I maxillary advancement: A preliminary report. Cleft Palate-Craniofacial Journal, 1997. 34(1): p. 36-45. | Title |
|  | McCarthy RC, Butaric LN. Changes to the face and basicranium in a habitually bipedal Japanese macaque (Macaca fuscata). American Journal of Physical Anthropology. 2015;156:220. | Title |
|  | McColley, S.A., Predicting the course of nutrition and lung disease in infants and children with cystic fibrosis. Journal of Cystic Fibrosis, 2020. 19(6): p. 847-849. | Title |
|  | McDonough JE, Verleden S, Vercauteren I, Verschakelen JA, Verbeken E, Decramer M, et al. 3-D reconstruction of small airways in IPF. American Journal of Respiratory and Critical Care Medicine. 2015;191. | Title |
|  | McGillick EV, te Pas AB, van den Akker T, Keus JMH, Thio M, Hooper SB. Evaluating Clinical Outcomes and Physiological Perspectives in Studies Investigating Respiratory Support for Babies Born at Term With or at Risk of Transient Tachypnea: A Narrative Review. Frontiers in Pediatrics. 2022;10. | Title |
|  | McInerney PL, Lee MSY, Clement AM, Worthy TH. The phylogenetic significance of the morphology of the syrinx, hyoid and larynx, of the southern cassowary, Casuarius casuarius (Aves, Palaeognathae). Bmc Evolutionary Biology. 2019;19(1). | Title |
|  | McKinney, K.A., et al., Transpalatal greater palatine canal injection: Radioanatomic analysis of where to bend the needle for pediatric sinus surgery. American Journal of Rhinology and Allergy, 2010. 24(5): p. 385-388. | Title |
|  | McNamara JA, Lione R, Franchi L, Angelieri F, Cevidanes LHS, Darendeliler MA, et al. The role of rapid maxillary expansion in the promotion of oral and general health. Progress in Orthodontics. 2015;16. | Title |
|  | Medal RM, Im AM, Yamamoto Y, Lakhdari O, Blackwell TS, Hoffman HM, et al. The innate immune response in fetal lung mesenchymal cells targets VEGFR2 expression and activity. American Journal of Physiology-Lung Cellular and Molecular Physiology. 2017;312(6):L861-L72. | Title |
|  | Medeiros MN, Yamashita R, Guo Y, Yaedu RYF, Perry J. Three-dimensional changes in the pharyngeal volume of skeletal class III patients after orthognathic surgery: impact on speech. Cleft Palate-Craniofacial Journal. 2018;55(1):120. | Title |
|  | Meer M, Siddiqi A, Morkel JA, van Rensburg PJ, Zafar S. Knife inflicted penetrating injuries of the maxillofacial region: A descriptive, record-based study. Injury-International Journal of the Care of the Injured. 2010;41(1):77-81. | Title |
|  | Mehta S, Gandhi V, Vich ML, Allareddy V, Tadinada A, Yadav S. Long-term assessment of conventional and mini-screw-assisted rapid palatal expansion on the nasal cavity. Angle Orthodontist. 2022;92(3):315-23. | Title |
|  | **Mei D, Han L, Yan Z, Huang H. Significance of comparison in upper airway morphology and hyoid position between skeletal class III malocclusion of high-angle and normal occlusion of adults by cone beam CT. Journal of Jilin University Medicine Edition. 2019;45(4):899-904.** | **Included** |
|  | Mellia J, Keenan B, Leinwand SE, Wiemken A, Hoge C, Schwab RJ. Classical heritability of upper airway anatomy in twins. American Journal of Respiratory and Critical Care Medicine. 2017;195. | Title |
|  | Mellick, L.B., Open airway, neutral cervical spine. Pediatric Emergency Care, 2016. 32(3): p. e7-e8. | Title |
|  | Mello Junior CF, Guimarães Filho HA, Gomes CA, Paiva CC. Radiological findings in patients with obstructive sleep apnea. J Bras Pneumol. 2013;39(1):98-101. | Title |
|  | **Mello PAS, Barreto BCT, Claudino LV, Mattos CT, Marañón-Vásquez GA, Araújo MTS, et al. Analysis of the middle region of the pharynx in adolescents with different anteroposterior craniofacial skeletal patterns. Dental Press J Orthod. 2019;24(5):60-8.** | **Included** |
|  | Meloni, V.A., et al., Novel homozygous ALX4 mutation causing frontonasal dysplasia-2 in a patient with meningoencephalocele. Clinical Genetics, 2015. 88(6): p. 593-596. | Title |
|  | Menéndez López-Mateos, M.L., et al., Three-dimensional photographic analysis of the face in European adults from southern Spain with normal occlusion: Reference anthropometric measurements. BMC Oral Health, 2019. 19(1). | Title |
|  | Meng K, Sun J, Li YL, Liu YS, Chen C, Xu ZX, et al. [The effect of orthognathic surgery on speech function in patients with skeletal Class Ⅲ malocclusion]. Shanghai Kou Qiang Yi Xue. 2021;30(4):394-401. | Title |
|  | Meng, N., J. Jiao, and L. Zhang, Culture of human nasal polyp epithelial cells at an air-liquid surface. Zhonghua er bi yan hou tou jing wai ke za zhi = Chinese journal of otorhinolaryngology head and neck surgery, 2014. 49(1): p. 49-53. | Title |
|  | Meoded A, Turan S, Harman C, Poretti A, Zinreich J, Huisman TA. Pre- and postnatal ultrasound and magnetic resonance imaging of intracranial extra-axial glioneuronal heterotopia. Fetal Diagn Ther. 2011;30(4):314-6. | Title |
|  | Mercadante VRG, Fontes PLP, Ciriaco FM, Henry DD, Moriel P, Ealy AD, et al. Effects of recombinant bovine somatotropin administration at breeding on cow, conceptus, and subsequent offspring performance of beef cattle. Journal of Animal Science. 2016;94(5):2128-38. | Title |
|  | Mercan E, Morrison CS, Stuhaug E, Shapiro LG, Tse RW. Novel computer vision analysis of nasal shape in children with unilateral cleft lip. Journal of Cranio-Maxillofacial Surgery. 2018;46(1):35-43. | Title |
|  | Merz E, Pashaj S. Anomalies of the fetal face. Donald School Journal of Ultrasound in Obstetrics and Gynecology. 2019;13(1):34-40. | Title |
|  | Mesanovic N, Huseinagic H, Mujagic S. 3D tracheobronchial airway tree segmentation from thorax CT images. Biomedical Engineering - Applications, Basis and Communications. 2013;25(1). | Title |
|  | Metes A, Hoffstein V, Direnfeld V, Chapnik JS, Zamel N. Three-dimensional CT reconstruction and volume measurements of the pharyngeal airway before and after maxillofacial surgery in obstructive sleep apnea. Journal of Otolaryngology. 1993;22(4):261-4. | Title |
|  | Metzler, P., et al., Surgically assisted maxillary expansion imparts three-dimensional nasal change. Journal of Oral and Maxillofacial Surgery, 2014. 72(10): p. 2005-2014. | Title |
|  | Meulstee, J.W., et al., The normal evolution of the cranium in three dimensions. International Journal of Oral and Maxillofacial Surgery, 2020. 49(6): p. 739-749. | Title |
|  | Meyerholz DK. CF in pigs - Modeling CF lung disease. Pediatric Pulmonology. 2011;46:106-7. | Title |
|  | Meyvaci SS, Kosif R, Bamac B, Hizal M, Ankarali H. Evaluation of apertura piriformis and related cranial anatomical structures through computed tomography: golden ratio. Folia Morphologica. 2019;78(4):839-46. | Title |
|  | Mietto, C., et al., Tracheal tube obstruction in mechanically ventilated patients assessed by high-resolution computed tomography. Anesthesiology, 2014. 121(6): p. 1226-1235. | Title |
|  | Millar, K., et al., Psychological status as a function of residual scarring and facial asymmetry after surgical repair of cleft lip and palate. Cleft Palate-Craniofacial Journal, 2013. 50(2): p. 150-157. | Title |
|  | Miller JL, Macedonia C, Sonies BC. Sex differences in prenatal oral-motor function and development. Developmental Medicine and Child Neurology. 2006;48(6):465-70. | Title |
|  | Miller NA, Gregory JS, Semple SIK, Aspden RM, Stollery PJ, Gilbert FJ. Relationships between vocal structures, the airway, and craniocervical posture investigated using magnetic resonance imaging. Journal of Voice. 2012;26(1):102-9. | Title |
|  | Minarik M, Stundl J, Fabian P, Jandzik D, Metscher BD, Psenicka M, et al. Pre-oral gut contributes to facial structures in non-teleost fishes. Nature. 2017;547(7662): p. 209-212. | Title |
|  | Miranda F, Garib D, Pugliese F, da Cunha Bastos JC, Janson G, Palomo JM. Upper airway changes in Class III patients using miniscrew-anchored maxillary protraction with hybrid and hyrax expanders: a randomized controlled trial. Clinical oral investigations. 2022;26(1):183‐95. | Title |
|  | Miranda GE, Wilkinson C, Roughley M, Beaini TL, Melani RFH. Assessment of accuracy and recognition of three-dimensional computerized forensic craniofacial reconstruction. PLoS ONE. 2018;13(5). | Title |
|  | Miranda, J.A. and A. Ernst, Complications in interventional pulmonology. Minerva Pneumologica, 2007. 46(2): p. 109-121. | Title |
|  | **Miranda-Viana M, Freitas DQ, Machado AH, Gomes AF, Nejaim Y. Do the dimensions of the hard palate have a relationship with the volumes of the upper airways and maxillary sinuses? A CBCT study. BMC Oral Health. 2021;21(1):356.** | **Included** |
|  | Mirhashemi SA, Arab S, Bahrami R. Effect of Bone Borne Expansion and Tooth Borne Palatal Expansion on Airway Volume: A Review Article. Journal of Mazandaran University of Medical Sciences. 2022;32(208):179-88. | Title |
|  | Mishima K, Sugahara T, Mori Y, Sakuda M. Three-dimensional comparison between the palatal forms in complete unilateral cleft lip and palate with and without Hotz plate from cheiloplasty to palatoplasty. Cleft palate-craniofacial journal. 1996;33(4). | Title |
|  | Mishima, K., et al., Comparison between the palatal configurations in complete and incomplete unilateral cleft lip and palate infants under 18 months of age. Cleft Palate-Craniofacial Journal, 2001. 38(1): p. 49-54. | Title |
|  | Mishra AK, Tramacere F, Guarino R, Pugno NM, Mazzolai B. A study on plant root apex morphology as a model for soft robots moving in soil. Plos One. 2018;13(6). | Title |
|  | Mistry N, Coulson C, George A. endoscope-i: an innovation in mobile endoscopic technology transforming the delivery of patient care in otolaryngology. Expert Rev Med Devices. 2017;14(11):913-8. | Title |
|  | Mitra, S., Towards statistically rigorous biometric authentication using facial images, in Statistical Methods in Counterterrorism: Game Theory, Modeling, Syndromic Surveillance, and Biometric Authentication. 2006. p. 47-79. | Title |
|  | Mittal N, El-Said HG, Ratnayaka K, Rao A, Friesen TL, Nigro JJ, et al. Bronchial stenting in infants with severe bronchomalacia: Technique and outcomes. Int J Pediatr Otorhinolaryngol. 2021;145:110703. | Title |
|  | Mittal R, Lee LA, Lin CH, Hsin LJ, Bhusri N, Li HY. Prediction of tongue obstruction observed from drug induced sleep computed tomography by cephalometric parameters. Auris Nasus Larynx. 2019;46(3):384-9. | Title |
|  | Miyamoto J, Miyamoto S, Nagasao T, Nakajima T, Kishi K. Anthropometric evaluation of bilateral cleft lip nose with cone beam computed tomography in early childhood: Estimation of nasal tip collapse. Journal of Plastic Reconstructive and Aesthetic Surgery. 2012;65(2):169-74. | Title |
|  | Miyamoto, J. and T. Nakajima, Anthropometric evaluation of complete unilateral cleft lip nose with cone beam CT in early childhood. Journal of Plastic, Reconstructive and Aesthetic Surgery, 2010. 63(1): p. 9-14. | Title |
|  | Miyamoto, S., et al., A boy with biallelic frameshift variants in TTC5 and brain malformation resembling tubulinopathies. Journal of Human Genetics, 2021. 66(12): p. 1189-1192. | Title |
|  | Miyashita, T., et al., The internal cranial morphology of an armoured dinosaur Euoplocephalus corroborated by X-ray computed tomographic reconstruction. Journal of Anatomy, 2011. 219(6): p. 661-675. | Title |
|  | Miyata, I., N. Sasaki, and Y. Eto, Congenital Deficiency of Growth Hormone and Prolactin. Clinical Pediatric Endocrinology, 1995. 4(1): p. 17-23. | Title |
|  | Miyata, K., et al., 15-hydroxy eicosadienoic acid is an exacerbating factor for nasal congestion in mice. FASEB Journal, 2022. 36(1). | Title |
|  | Miyawaki S, Hoffman EA, Wenzel SE, Lin CL. Aerosol deposition predictions in computed tomography-derived skeletons from severe asthmatics: A feasibility study. Clinical Biomechanics. 2019;66:81-7. | Title |
|  | Miyawaki S, Tawhai MH, Hoffman EA, Wenzel SE, Lin CL. Automatic construction of subject-specific human airway geometry including trifurcations based on a CT-segmented airway skeleton and surface. Biomech Model Mechanobiol. 2017;16(2):583-96. | Abstract |
|  | Mobasher WA, Asiri BM, Asiri LA, Saad LM, Meshawi EA, Assiri HA, et al. CLEFT LIP AND PALATE SURGICAL MANAGEMENT IN PEDIATRIC: SYSTEMATIC LITERATURE REVIEW. Indo American Journal of Pharmaceutical Sciences. 2019;6(1):5-10. | Title |
|  | Moerenhout, B.A.M.M.L., et al., Accuracy and repeatability of cone-beam computed tomography (CBCT) measurements used in the determination of facial indices in the laboratory setup. Journal of Cranio-Maxillofacial Surgery, 2009. 37(1): p. 18-23. | Title |
|  | Mohamed AM, Al Bishri A, Mohamed AH. Distraction osteogenesis as followed by CT scan in Pierre Robin sequence. Journal of Cranio-Maxillofacial Surgery. 2011;39(6):412-9. | Title |
|  | Mohamed AS, Habumugisha J, Cheng B, Zhao MY, Guo YC, Zou R, et al. Three-dimensional evaluation of hyoid bone position in nasal and mouth breathing subjects with skeletal Class I, and Class II. Bmc Oral Health. 2022;22(1). | Abstract |
|  | Mohammadieh A, Sutherland K, Cistulli P. Influence of maxillary width and obesity measures on OSA severity. Sleep and Biological Rhythms. 2014;12:31-2. | Title |
|  | Mohassel, P., et al., Cross-sectional Neuromuscular Phenotyping Study of Patients with Arhinia with SMCHD1 Variants. Neurology, 2022. 98(13): p. E1384-E1396. | Title |
|  | Moin Anwer, H.M., et al., The role of the dentist in the diagnosis and management of pediatric obstructive sleep apnea. Saudi Dental Journal, 2021. 33(7): p. 424-433. | Title |
|  | Molaei S, Esmaeili F, Sadrhaghighi A, Khatoonabad MJ, Oskoei DS, Esmaeilijah N. Determine and Compare the Volume and Length of the Upper Airway Using Cone‑beam Computed Tomography Images in Patients with Obstructive Sleep Apnea. Asian Journal of Pharmaceutics. 2018;12(2):S484-S9. | Title |
|  | Molteni, G., et al., Voluminous frontoethmoidal mucocele with epidural involvement. Surgical treatment by coronal approach. Acta otorhinolaryngologica Italica : organo ufficiale della Società italiana di otorinolaringologia e chirurgia cervico-facciale, 2003. 23(3): p. 185-190. | Title |
|  | Momany SM, AlJamal G, Shugaa-Addin B, Khader YS. Cone Beam Computed Tomography Analysis of Upper Airway Measurements in Patients With Obstructive Sleep Apnea. American Journal of the Medical Sciences. 2016;352(4):376-84. | Title |
|  | Mondello C, Baldino G, Bottari A, Sapienza D, Perri F, Argo A, et al. The role of PMCT for the assessment of the cause of death in natural disaster (landslide and flood): a Sicilian experience. Int J Legal Med. 2022;136(1):237-44. | Title |
|  | Monk, C.S., et al., Adolescent immaturity in attention-related brain engagement to emotional facial expressions. NeuroImage, 2003. 20(1): p. 420-428. | Title |
|  | Montaudon M, Berger P, De Dietrich G, Braquelaire A, Marthan R, Tunon-De-Lara JM, et al. Assessment of airways with three-dimensional quantitative thin-section CT: In vitro and in vivo validation. Radiology. 2007;242(2):563-72. | Title |
|  | Montgomery-Downs H, Lewis JW, Brefczynski-Lewis J, Clawges H, Carey ME, Hou B, et al. Infant feeding method and pediatric sleep-disordered breathing. Sleep. 2015;38:A355. | Title |
|  | Moradi E, Sheikh M, Vaezi T. Cone beam computed tomography (CBCT) evaluation of age-related upper airway changes. Bangladesh Journal of Medical Science. 2016;15(1):78-83. | Abstract |
|  | Morawska-Kochman M, Nelke K, Nienartowicz J, Pawlak W, Bochnia M. Technical aspects of nasal cavity surgery through the Le Fort I down-fracture approach: An otolaryngologist’s point of view based on 90 patients’ experience. Advances in Clinical and Experimental Medicine. 2019;28(2):203-10. | Title |
|  | Moreddu E, Le Treut-Gay C, Triglia JM, Nicollas R. Congenital nasal pyriform aperture stenosis: Elaboration of a management algorithm from 25 years of experience. International Journal of Pediatric Otorhinolaryngology. 2016;83:7-11. | Title |
|  | Moreddu, E., et al., Morphometric measurements and sexual dimorphism of the piriform aperture in adults. Surgical and Radiologic Anatomy, 2013. 35(10): p. 917-924. | Title |
|  | Morelli DL, Jackson N, Gislason T, Arnardottir ES, Benediktsdottir B, Juliusson S, et al. The effect of edentulism on AHI, ODI, and tongue morphology in Iceland Sleep Apnea Cohort (ISAC). American Journal of Respiratory and Critical Care Medicine. 2011;183(1). | Title |
|  | Mori T, Nishino E, Jitsukawa T, Hoshino E, Hirakawa S, Kuroiwa Y, et al. Chiari type 1 malformation associated with central sleep apnea after high dose growth hormone (GH) therapy in a 12-year-old boy: A case report. Clinical Pediatric Endocrinology. 2018;27(1):45-51. | Title |
|  | Mori Y, Hoshi K, Takato T, Takahashi M, Hirano Y, Kanno Y, et al. Submucous cleft palate: variations in bony defects of the hard palate. Br J Oral Maxillofac Surg. 2013;51(8):e220-3. | Title |
|  | Mori, A., et al., Analysis of 109 Japanese children's lip and nose shapes using 3-dimensional digitizer. British Journal of Plastic Surgery, 2005. 58(3): p. 318-329. | Title |
|  | Morina Q, Bunjaku D, Matoshi D, Katanolli F, Morina A, Kelmendi F. Management of difficult airway in neonate with giant anterior encephalocele. European Journal of Anaesthesiology. 2014;31:281. | Title |
|  | Morioka D, Mandrano N, Fujimoto H, Koga Y, Sato N, Tosa Y, et al. Longitudinal Follow-up of Individuals With Cleft Lip Using Three-Dimensional Stereophotogrammetry. Journal of Craniofacial Surgery. 2018;29(5):1261-5. | Title |
|  | Moritz L, Wesener T, Koch M. An apparently non-swinging tentorium in the Diplopoda (Myriapoda): comparative morphology of the tentorial complex in giant pill-millipedes (Sphaerotheriida). Zookeys. 2018(741):77-91. | Title |
|  | Moroi, A., et al., Evaluation of soft tissue morphologic changes after using the alar base cinch suture in le Fort i osteotomy in mandibular prognathism with and without asymmetry. Journal of Cranio-Maxillofacial Surgery, 2014. 42(6): p. 718-724. | Title |
|  | Morra A, Calgaro A, Cioffi V, Pravato M, Cova M, Pozzi Mucelli R. [Virtual endoscopy of the nasal cavity and the paranasal sinuses with computerized tomography. Anatomical study]. Radiol Med. 1998;96(1-2):29-34. | Title |
|  | Morris GE, Bridge JC, Eltboli OMI, Knox AJ, Aylott JW, Brightling CE, et al. Human airway smooth muscle maintain in situ cell orientation and phenotype when cultured on aligned electrospun scaffolds. American Journal of Physiology-Lung Cellular and Molecular Physiology. 2014;307(1):L38-L47. | Title |
|  | Morris JM, Lane JI, Witte RJ, Thompson DM. Giant cell reparative granuloma of the nasal cavity. AJNR Am J Neuroradiol. 2004;25(7):1263-5. | Title |
|  | Morrison, L.J., et al., Part 8: Advanced life support: 2010 International Consensus on Cardiopulmonary Resuscitation and Emergency Cardiovascular Care Science with Treatment Recommendations. Circulation, 2010. 122(16 SUPPL. 2): p. S345-S421. | Title |
|  | Morrison, S.L., C.K. Campbell, and G.M. Wright, Chondrogenesis of the branchial skeleton in embryonic sea lamprey, Petromyzon marinus. Anatomical Record, 2000. 260(3): p. 252-267. | Title |
|  | Morrow KL, Park RD, Spurgeon TL, Stashak TS, Arceneaux B. Computed tomographic imaging of the equine head. Veterinary Radiology & Ultrasound. 2000;41(6):491-7. | Title |
|  | Morsing, E., P. Gustafsson, and J. Brodszki, Lung function in children born after foetal growth restriction and very preterm birth. Acta Paediatrica, International Journal of Paediatrics, 2012. 101(1): p. 48-54. | Title |
|  | Moscarino S, Kotter F, Brandt M, Modabber A, Kniha K, Holzle F, et al. Influence of different surgical concepts for moderate skeletal class II and III treatment on the nasopharyngeal airway space. Journal of Cranio-Maxillofacial Surgery. 2019;47(10):1489-97. | Title |
|  | Moses, J.J., C.R. Lange, and A. Arredondo, Endoscopic treatment of sinonasal disease in patients who have had orthognathic surgery. British Journal of Oral and Maxillofacial Surgery, 2000. 38(3): p. 177-184. | Title |
|  | **Moshajari A, Irannezhad A, Kajan ZD, Nasab NK, Rafiei E, Kiani P. Correlation of palatal volume with nasopharyngeal volume on computed tomography scans of an Iranian subpopulation. Orthodontic Waves. 2020;79(1):31-8.** | **Included** |
|  | Moshfeghi M, Abedian B, Ahsaie MG, Tajdini F. Prevalence of Nasal Septum Deviation Using Cone-Beam Computed Tomography: A Cross-Sectional Study. Contemporary Clinical Dentistry. 2020;11(3):223-8. | Title |
|  | Mosleh, M.I., et al., Comparison of transverse changes during maxillary expansion with 4-point bone-borne and tooth-borne maxillary expanders. American Journal of Orthodontics and Dentofacial Orthopedics, 2015. 148(4): p. 599-607. | Title |
|  | Mostafiz W, Viana G, Kusnoto B, Ma S, Darendeliler M, Carley DW, et al. Airway analysis and mandibular advancement treatment in obstructive sleep apnea. Sleep. 2015;38:A193. | Title |
|  | Mostafiz WR, Carley DW, Viana MGC, Ma S, Dalci O, Darendeliler MA, et al. Changes in sleep and airway variables in patients with obstructive sleep apnea after mandibular advancement splint treatment. Am J Orthod Dentofacial Orthop. 2019;155(4):498-508. | Title |
|  | Motro M, Schauseil M, Ludwig B, Zorkun B, Mainusch S, Ateş M, et al. Rapid-maxillary-expansion induced rhinological effects: a retrospective multicenter study. European Archives of Oto-Rhino-Laryngology. 2016;273(3):679-87. | Title |
|  | Mouhanna-Fattal C, Papadopoulos M, Bouserhal J, Tauk A, Bassil-Nassif N, Athanasiou A. Evaluation of upper airway volume and craniofacial volumetric structures in obstructive sleep apnoea adults: A descriptive CBCT study. Int Orthod. 2019;17(4):678-86. | Title |
|  | Mousavi SE, Patil JG. Stages of embryonic development in the live-bearing fish, Gambusia holbrooki. Developmental Dynamics. 2022;251(2):287-320. | Title |
|  | Moustafa MA, Arida EA, Zanaty OM, El-Tamboly SF. Endotracheal intubation: ultrasound-guided versus fiberscope in patients with cervical spine immobilization. Journal of anesthesia. 2017;31(6):846‐51. | Title |
|  | Mu L, Sanders I. Sihler's whole mount nerve staining technique: a review. Biotechnic & Histochemistry. 2010;85(1):19-42. | Title |
|  | Mu, L. and I. Sanders, Neuromuscular organization of the canine tongue. Anatomical Record, 1999. 256(4): p. 412-424. | Title |
|  | Mueller AA, Paysan P, Schumacher R, Zeilhofer HF, Berg-Boerner BI, Maurer J, et al. Missing facial parts computed by a morphable model and transferred directly to a polyamide laser-sintered prosthesis: an innovation study. British Journal of Oral & Maxillofacial Surgery. 2011;49(8):E67-E71. | Title |
|  | Mukai T, Shirai M, Aoyama A, Ishibazawa E, Nii M, Yoshida Y, et al. Interstitial deletion of the long arm of chromosome 2 in IgA deficiency and Graves disease. Hormone Research in Paediatrics. 2013;80:353. | Title |
|  | Mulliken JB, Burvin R, Farkas LG. Repair of bilateral complete cleft lip: Intraoperative nasolabial anthropometry. Plastic and Reconstructive Surgery. 2001;107(2):307-14. | Title |
|  | Mulliken JB, Martinez-Perez D. The principle of rotation advancement for repair of unilateral complete cleft lip and nasal deformity: Technical variations and analysis of results. Plastic and Reconstructive Surgery. 1999;104(5):1247-60. | Title |
|  | Mulliken JB, Trier WC. Bilateral complete cleft lip and nasal deformity: An anthropometric analysis of staged to synchronous repair. Plastic and Reconstructive Surgery. 1995;96(1):9-25. | Title |
|  | Mulliken JB, Wu JK, Padwa BL. Repair of bilateral cleft lip: Review, revisions, and reflections. Journal of Craniofacial Surgery. 2003;14(5):609-20. | Title |
|  | Mulliken JB. Bilateral cleft lip. Clinics in Plastic Surgery. 2004;31(2):209-+. | Title |
|  | Mulliken, J.B. and R.A. Labrie, Fourth-dimensional changes in nasolabial dimensions following rotation-advancement repair of unilateral cleft lip. Plastic and Reconstructive Surgery, 2012. 129(2): p. 491-498. | Title |
|  | Mummy, D.G., et al., Asthma, in Medical Radiology. 2018. p. 223-253. | Title |
|  | Muñoz-Guerra, M.F., L. Naval-Gías, and A. Capote-Moreno, Le Fort I Osteotomy, Bilateral Sinus Lift, and Inlay Bone-Grafting for Reconstruction in the Severely Atrophic Maxilla: A New Vision of the Sandwich Technique, Using Bone Scrapers and Piezosurgery. Journal of Oral and Maxillofacial Surgery, 2009. 67(3): p. 613-618. | Title |
|  | Münster T, Hoffmann M, Schlaffer S, Ihmsen H, Schmitt H, Tzabazis A. Anatomical location of the vocal cords in relation to cervical vertebrae. European Journal of Anaesthesiology. 2016;33(4):257-62. | Title |
|  | Murad H, Ghabrah T, Rafeeq M, Ali S. Subdiuretic dose of furosemide enhances albuterol effects in asthmatic mice rather than bumetanide. Allergol immunopatol. 2018;46(6):585-93. | Title |
|  | Murakami D, Inada E, Saitoh I, Takemoto Y, Morizono K, Kubota N, et al. Morphological differences of facial soft tissue contours from child to adult of Japanese males: A three-dimensional cross-sectional study. Archives of Oral Biology. 2014;59(12):1391-9. | Title |
|  | Murakami N, Sato N, Morioka D, Tosa Y, Ohkubo F, Kadomatsu K. Association between three-dimensional measurements of the unilateral cleft lip nasal deformity and maxillary alveolar morphology: A retrospective study. Journal of Plastic Reconstructive and Aesthetic Surgery. 2019;72(8):1411-7. | Title |
|  | Murga A, Kuga K, Yoo SJ, Ito K. Can the inhalation exposure of a specific worker in a cross-ventilated factory be evaluated by time- and spatial-averaged contaminant concentration? Environmental Pollution. 2019;252:1388-98. | Title |
|  | Musa, N., et al., A novel POU1F1 pathogenic variant: Two familial case reports with phenotype expansion. Clinical Genetics, 2021. 100(5): p. 641-642. | Title |
|  | Musilová, B., et al., Exocranial surfaces for sex assessment of the human cranium. Forensic Science International, 2016. 269: p. 70-77. | Title |
|  | Mutalik S, Rengasamy K, Tadinada A. Incidental findings based on anatomical location and clinical significance in CBCT scans of dental implant patients. Quintessence Int. 2018;49(5):419-26. | Title |
|  | Mydlova M, Dupej J, Koudelova J, Veleminska J. Sexual dimorphism of facial appearance in ageing human adults: A cross-sectional study. Forensic Science International. 2015;257. | Title |
|  | Myga-Porosiło J, Skrzelewski S, Surga W, Borowiak H, Jackowska Z, Kluczewska E. CT imaging of facial trauma. the role of different types of reconstruction. part II - Soft tissues. Polish Journal of Radiology. 2011;76(1):52-8. | Title |
|  | Mylavarapu G, Fleck R, Subramanyam R, Frimpong-Manso M, Amin R, Mahmoud MA. Effect of a combination of dexmedetomidine and ketamine anesthesia on the upper airway caliber in normal children. American Journal of Respiratory and Critical Care Medicine. 2017;195. | Title |
|  | Mylavarapu G, Fleck RJ, Ok MS, Ding L, Kandil A, Amin RS, et al. Effects on the upper airway morphology with intravenous addition of ketamine after dexmedetomidine administration in normal children. Journal of Clinical Medicine. 2020;9(11):1-14. | Title |
|  | Na, J.S., et al., Computational analysis of airflow dynamics for predicting collapsible sites in the upper airways: A preliminary study. Journal of Applied Physiology, 2019. 126(2): p. 330-340. | Title |
|  | Nackos JS, Wiggins RH, 3rd, Harnsberger HR. CT and MR imaging of giant cell granuloma of the craniofacial bones. AJNR Am J Neuroradiol. 2006;27(8):1651-3. | Title |
|  | Nada, R.M., et al., Volumetric changes of the nose and nasal airway 2 years after tooth-borne and bone-borne surgically assisted rapid maxillary expansion. European Journal of Oral Sciences, 2013. 121(5): p. 450-456. | Title |
|  | Nadazdyova A, Samohyl M, Stefankova E, Pintesova S, Stanko P. Human race as indicator of 3D planning of soft tissue of face and multidisciplinary approach. Bratislava Medical Journal-Bratislavske Lekarske Listy. 2017;118(7):431-6. | Title |
|  | Nádaždyová, A., E. Štefánková, and M. Samohýl, The impact of body mass index on craniofacial parameters. Kontakt, 2016. 18(4): p. e253-e257. | Title |
|  | Nadeem S, Jin D, Hoffman EA, Saha PK. A fully automated airway segmentation algorithm from chest CT images at total lung capacity. American Journal of Respiratory and Critical Care Medicine. 2017;195. | Title |
|  | Nadeem, S.A., et al., A novel iterative method for airway tree segmentation from CT imaging using multiscale leakage detection, in Lecture Notes in Computer Science (including subseries Lecture Notes in Artificial Intelligence and Lecture Notes in Bioinformatics). 2017. p. 46-60. | Title |
|  | Nadjmi, N., et al., Comparison of soft-tissue profiles in le Fort i osteotomy patients with Dolphin and Maxilim softwares. American Journal of Orthodontics and Dentofacial Orthopedics, 2013. 144(5): p. 654-662. | Title |
|  | Nagasao T, Miyamoto J, Hikosaka M, Yoshikawa K, Ishii N, Nakajima T. A new method to quantify subtle morphological deformities in nasal profile curvatures and its application for analysis of unilateral cleft lip noses. Journal of Cranio-Maxillofacial Surgery. 2008;36(6):321-34. | Title |
|  | Nagasao, T., et al., An anatomical study of the three-dimensional structure of the nasal septum in patients with alveolar clefts and alveolar-palatal clefts. Plastic and Reconstructive Surgery, 2008. 121(6): p. 2074-2083. | Title |
|  | Nagata H, Kamo H, Kato T. Tremor-like involuntary movements of the head due to midbrain infarction. Clinical Neurology. 1982;22(6):521-5. | Title |
|  | Naguib M, Malabarey T, AlSatli RA, Al Damegh S, Samarkandi AH. Predictive models for difficult laryngoscopy and intubation. A clinical, radiologic and three-dimensional computer imaging study. Canadian Journal of Anaesthesia-Journal Canadien D Anesthesie. 1999;46(8):748-59. | Title |
|  | Nagy K, Mommaerts MY. Analysis of the cleft-lip nose in submental-vertical view, Part I - Reliability of a new measurement instrument. Journal of Cranio-Maxillofacial Surgery. 2007;35(6-7):265-77. | Title |
|  | Nair, A., Computed tomography of the neck. BMJ, 2011. 342(7805). | Title |
|  | NakagawaT et al. Interaction between fibronectin and eosinophils in the growth of nasal polyps. Laryngoscope, 1999. 109(4): 557-561. | Title |
|  | Nakamura M, Wada S, Miki T, Shimada Y, Suda Y, Tamura G. Automated segmentation and morphometric analysis of the human airway tree from multidetector CT images. J Physiol Sci. 2008;58(7):493-8. | Title |
|  | Nakamura N, Okawachi T, Nishihara K, Hirahara N, Nozoe E. Surgical Technique for Secondary Correction of Unilateral Cleft Lip-Nose Deformity: Clinical and 3-Dimensional Observations of Preoperative and Postoperative Nasal Forms. Journal of Oral and Maxillofacial Surgery. 2010;68(9):2248-57. | Title |
|  | Nakamura N, Okawachi T, Nozoe E, Nishihara K, Matsunaga K. Three-dimensional analyses of nasal forms after secondary treatment of bilateral cleft lipnose deformity in comparison to those of healthy young adults. Journal of Oral and Maxillofacial Surgery. 2011;69(11):e469-e81. | Title |
|  | Nakamura N, Suzuki A, Takahashi H, Honda Y, Sasaguri M, Ohishi M. A longitudinal study on influence of primary facial deformities on maxillofacial growth in patients with cleft lip and palate. Cleft Palate-Craniofacial Journal. 2005;42(6):633-40. | Title |
|  | Nakamura, N., et al., Secondary correction of bilateral cleft lip nose deformity - Clinical and three-dimensional observations on pre- and postoperative outcome. Journal of Cranio-Maxillofacial Surgery, 2011. 39(5): p. 305-312. | Title |
|  | Nakano H, Mishima K, Matsushita A, Suga H, Matsumura M, Miyawaki Y, et al. Relationship between airway volume and sleep-disordered breathing. Sleep and Biological Rhythms. 2011;9(4):281. | Title |
|  | Nakao T, Katayama M, Lee Y, Kino H, Mitsuno D, Kawai S, et al. 17-year follow-up after distraction osteogenesis performed in a 4-month old infant with robin sequence. Congenital Anomalies. 2019;59(6):A67. | Title |
|  | Nakao, K., et al., The effects of C-type natriuretic peptide on craniofacial skeletogenesis. Journal of Dental Research, 2013. 92(1): p. 58-64. | Title |
|  | Nalabothu P, Verna C, Benitez BK, Dalstra M, Mueller AA. Load Transfer during Magnetic Mucoperiosteal Distraction in Newborns with Complete Unilateral and Bilateral Orofacial Clefts: A Three-Dimensional Finite Element Analysis. Applied Sciences-Basel. 2020;10(21). | Title |
|  | Nandalike K, Strauss T, Stakofsky A, Bent J, Bassila M, Parikh S, et al. Volumetric analysis of the upper airway before and after adenotonsillectomy in obese children with obstructive sleep apnea. American Journal of Respiratory and Critical Care Medicine. 2011;183(1). | Title |
|  | Naran S, Kinsella C, MacIsaac Z, Katzel E, Bykowski M, Shakir S, et al. Pediatric facial fracture patterns: Trajectories and ramifications in 151 patients. Cleft Palate-Craniofacial Journal. 2014;51(3):e36-e7. | Title |
|  | Narang K, Rust L, Wick M. eP460: Diagnostic dilemma in a case of suspected fetal skeletal dysplasia. Genetics in Medicine. 2022;24(3):S289. | Title |
|  | Nardelli P, Khan KA, Corvò A, Moore N, Murphy MJ, Twomey M, et al. Optimizing parameters of an open-source airway segmentation algorithm using different CT images. Biomed Eng Online. 2015;14:62. | Title |
|  | Narimatsu, K., A. Iida, and T. Kobayashi, Palatoplasty for the Patient With Campomelic Dysplasia—Report of a Case and Review of the Literature. Cleft Palate-Craniofacial Journal, 2022. 59(1): p. 132-136. | Title |
|  | Naseh R, Azami N, Tofangchiha M, Sabzevaripour P, Shirazi M. Cephalometric analysis of upper airways in class I malocclusion in adults in Qazvin. Journal of Mazandaran University of Medical Sciences. 2016;26(138):55-65. | Abstract |
|  | **Nath M, Ahmed J, Ongole R, Denny C, Shenoy N. CBCT analysis of pharyngeal airway volume and comparison of airway volume among patients with skeletal Class I, Class II, and Class III malocclusion: A retrospective study. Cranio-the Journal of Craniomandibular & Sleep Practice. 2021;39(5):379-90.** | **Included** |
|  | Navales I, Paredes P, Cols M, Perissinotti A, Vancells M, Pons F. Utilidad de la tomografía por emisión de positrones con 18F-FDG en un caso de papilomatosis pulmonar recurrente juvenil. Rev esp med nucl imagen mol (Ed impr). 2013;32(6):387-9. | Title |
|  | Navasumrit S, Chen YA, Hsieh YJ, Yao CF, Chang CS, Chen NH, et al. Skeletal and upper airway stability following modified maxillomandibular advancement for treatment of obstructive sleep apnea in skeletal class I or II deformity. Clin Oral Investig. 2022;26(3):3239-50. | Title |
|  | Nct. Botulinum Toxin to Improve Results in Cleft Lip Repair. https://clinicaltrialsgov/show/NCT01429402. 2011. | Title |
|  | Nct. Comparison Between the Subglotic Diameter and the Epiphyseal Diameter of the Radius in Children for Prediction of Appropriate Endotracheal Tube Sizes. https://clinicaltrialsgov/show/NCT03713385. 2018. | Title |
|  | Nct. Comparison of Treatment Effects of PowerScope2 and Forsus Using CBCT. https://clinicaltrialsgov/show/NCT03296644. 2017. | Title |
|  | Nct. Dentoskeletal Effects of the Expander With Differential Opening and the Fan-type Expander. https://clinicaltrialsgov/show/NCT03705871. 2018. | Title |
|  | Nct. Effect of Coffee and Tea Consumption on Adolescent Weight Control. https://clinicaltrialsgov/show/NCT05181176. 2022. | Title |
|  | Nct. Heat Therapy to Prevent Deconditioning During Immobilization. https://clinicaltrialsgov/show/NCT05021523. 2021. | Title |
|  | Nct. Mesenchymal Stemcells for Radiation Induced Xerostomia. https://clinicaltrialsgov/show/NCT02513238. 2015. | Title |
[truncated: 228,993 more chars]
